# Development and validation of a model based on immunogenic cell death related genes to predict the prognosis and immune response to bladder urothelial carcinoma

**DOI:** 10.3389/fonc.2023.1291720

**Published:** 2023-11-10

**Authors:** Lizhu Chen, Jiexiang Lin, Yaoming Wen, Yu Chen, Chuan-ben Chen

**Affiliations:** ^1^Department of Medical Oncology, Clinical Oncology School of Fujian Medical University, Fujian Cancer Hospital, Fuzhou, Fujian, China; ^2^Cancer Bio-immunotherapy Center, Clinical Oncology School of Fujian Medical University, Fujian Cancer Hospital, Fuzhou, Fujian, China; ^3^Fujian Provincial Key Laboratory of Translational Cancer Medicine, Fuzhou, Fujian, China; ^4^The Shengli Clinical Medical College, Fujian Medical University, Fuzhou, Fujian, China; ^5^Drug Development, Fujian Institute of Microbiology, Fuzhou, Fujian, China; ^6^Department of Radiation Oncology, Clinical Oncology School of Fujian Medical University, Fujian Cancer Hospital, Fuzhou, Fujian, China

**Keywords:** bladder urothelial carcinoma, immunogenic cell death, bioinformatics, immune, prognostic model

## Abstract

**Background:**

Immunogenic cell death (ICD) has been categorized as a variant of regulated cell death that is capable of inducing an adaptive immune response. A growing body of evidence has indicated that ICD can modify the tumor immune microenvironment by releasing danger signals or damage-associated molecular patterns (DAMPs), potentially enhancing the efficacy of immunotherapy. Consequently, the identification of biomarkers associated with ICD that can classify patients based on their potential response to ICD immunotherapy would be highly advantageous. Therefore the goal of the study is to better understand and identify what patients with bladder urothelial carcinoma (BLCA) will respond to immunotherapy by analyzing ICD signatures and investigate ICD-related prognostic factors in the context of BLCA.

**Methods:**

The data obtained from The Cancer Genome Atlas (TCGA) and Gene Expression Omnibus (GEO) databases regarding BLCA and normal samples was categorized based on ICD-related genes (IRGs). Specifically, we conducted an immunohistochemical (IHC) experiment to validate the expression levels of Calreticulin (CALR) in both tumor and adjacent tissues, and evaluated its prognostic significance using the Kaplan-Meier (KM) curve. Subsequently, the samples from TCGA were divided into two subtypes using consensus clustering. To obtain a more comprehensive comprehension of the biological functions, we utilized Gene Ontology (GO), Kyoto Encyclopedia of Genes and Genomes (KEGG), and Gene Set Enrichment Analysis (GSEA). The calculation of immune landscape between two subtypes was performed through ESTIMATE and CIBERSORT. Risk models were constructed using Cox and Lasso regression and their prognosis predictive ability was evaluated using nomogram, receiver operating characteristic (ROC), and calibration curves. Finally, Tumor Immune Dysfunction and Exclusion (TIDE) algorithms was utilized to predict the response to immunotherapy.

**Results:**

A total of 34 IRGs were identified, with most of them exhibiting upregulation in BLCA samples. The expression of CALR was notably higher in BLCA compared to the adjacent tissue, and this increase was associated with an unfavorable prognosis. The differentially expressed genes (DEGs) associated with ICD were linked to various immune-related pathways. The ICD-high subtypes exhibited an immune-activated tumor microenvironment (TME) compared to the ICD-low subtypes. Utilizing three IRGs including *CALR*, *IFNB1*, and *IFNG*, a risk model was developed to categorize BLCA patients into high- and low-risk groups. The overall survival (OS) was considerably greater in the low-risk group compared to the high-risk group, as evidenced by both the TCGA and GEO cohorts. The risk score was identified as an independent prognostic parameter (all *p* < 0.001). Our model demonstrated good predictive ability (The area under the ROC curve (AUC), AUC_1_-year= 0.632, AUC_3_-year= 0.637, and AUC_5_-year =0.653). Ultimately, the lower risk score was associated with a more responsive immunotherapy group.

**Conclusion:**

The potential of the ICD-based risk signature to function as a marker for evaluating the prognosis and immune landscape in BLCA suggests its usefulness in identifying the suitable population for effective immunotherapy against BLCA.

## Introduction

Urothelial carcinoma originates from the transitional epithelium of the bladder. In 2023, 82,290 new urinary bladder cases and 16,710 cancer deaths are projected to occur in the United States (1). Over the years, immunotherapy has completely changed the treatment patterns for advanced urothelial cancer which was dominated by chemotherapy (2, 3). But the bladder urothelial carcinoma (BLCA) still has a high risk for recurrence (4) and a low 5-year overall survival (OS) rate (5). Hence, it is imperative to employ sensitive methods for the precise assessment of clinical prognosis outcomes in patients with BLCA, thereby fostering advancements in the field of precision medicine.

The tumor microenvironment (TME) contains various cellular components interacting with cytokines, chemokines, and growth factors (6). Based on its distinctive features, tumors can be categorized as either immuno-cold or immuno-hot types, which could offer a base for identifying the efficacy of immunotherapies (7). Immuno-cold tumors are characterized by an immunosuppressive TME and exhibit limited responsiveness to immunotherapies. Conversely, immuno-hot tumors are associated with a heightened response to immunotherapy and accompanied by robust infiltration of active T-cells (8). Therefore, it is crucial to utilize practical biomarkers to distinguish tumor type and its response to immunotherapy.

Immune checkpoint inhibitors (ICIs) used for treating advanced BLCA have impressive response rates and toxicity characteristics (9, 10). However, urological cancers have different response rates to immunotherapies because of immunogenic cancer cells (11). And the efficacy of these treatments is limited to a minority of patients (12), with a substantial proportion experiencing either a restricted or non-existent response, particularly in advanced BLCA cases. Therefore, additional research is warranted to explore the correlation between immunity and BLCA with the aim of exploring the possible prognostic significance of immune and immune-related indicators.

Immunogenic cell death (ICD), as a modality of immunostimulatory cell death, was initially discovered and developed by the laboratories of Guido Kroemer and Laurence Zitvogel (13, 14). ICD can be delineated by its elicitation of three primary damage associated molecular patterns (DAMPs), namely the translocation of calreticulin, active secretion of adenosine triphosphate, and release of the high mobility group box 1 protein (13, 15, 16). ICD is an exceptional form of cellular demise induced by diverse modalities for cancer treatment, including radiotherapy and chemotherapeutic agents. By inducing ICD, the non-immunogenicity of tumor cells can be transformed into immunogenicity, leading to the initiation of an antitumor immune response and the elimination of tumor cells (17). Therefore, the activation of immune cells and the eradication of tumors are significantly influenced by the presence of ICD (18). The activation of ICD in cancer cells has the potential to address the existing limitations of immunotherapy employed in tumor treatment (19). However, there is a dearth of research on the potential applications and underlying mechanisms of utilizing ICD for the treatment of BLCA.

Researchers have developed a model based on ICD codes for predicting prognosis and immunotherapy response in other carcinomas (20). The association between genes associated with ICD and the prognosis of BLCA remains uncertain at present. In order to enhance the ability to forecast the effectiveness of immunotherapy in BLCA patients and provide guidance for clinical treatments, it is necessary to identify novel biomarkers. Therefore, we developed an ICD-related genes (IRGs) risk model to excavate its relationships with tumor immune environment, prognosis, and immune treatment response of BLCA patients. This may help to provide a molecular-level basis for screening populations with effective immunotherapy in BLCA.

## Materials and methods

### Analysis of differentially expressed genes

For our study, transcriptome profiling data for 429 BLCA patients came from The Cancer Genome Atlas (TCGA) (https://portal.gdc.cancer.gov/repository) database (**Supplementary Table 1**). The ICD parameters were assessed in this study through an extensive review of relevant research investigations conducted *in vivo* using mice and/or *in vitro* using primary human immune cells, utilizing databases such as Web of Knowledge, Scopus, and PubMed (21). Garg et al. have identified the metagene signatures derived from ICD that are associated with enhanced patient survival and provided confirmation that the ICD can function as a valuable tool for the identification of prognostic metagenes (21). The expression of IRGs in BLCA tumors and normal tissues was calculated using the limma R package from Bioconductor (www.bioconductor.org), which employed a linear model for microarray data. The ratio of all IRGs in the samples was counted to identify the fold-change FC of ICD-high group and ICD-low group. The |log2FC| was set at >1 and false discovery rate (FDR) was set at <0.05. The protein-protein interaction (PPI) network of DEGs was performed using the STRING database (https://string-db.org/).

### Immunohistochemistry

Furthermore, immunohistochemical (IHC) experiments were conducted to determine the expression level of Calreticulin (CALR) in BLCA tissues and adjacent tissues. The BLCA sample tissue chip (HBlaU079Su01) was from Outdo Biotechnology (Shanghai, China). The detailed clinical information was downloaded from the companys website (**Supplementary Table 2**). The experiment steps were as follows. First, we placed the tissue chips into an oven, set temperature to 63 degree, and waxed it for one hour. Second, dewaxing was performed in a fully automatic dying machine. Third, we put the slides into the performing antigen repair apparatus and started the repair after the selection procedure. After repair, the slides were subjected to natural cooling in distilled water at ambient temperature. Fourth, we washed the slides with PBS buffer and added the diluted primary antibody working solution (CALR Rabbit pAb, 1:2000, ABclonal, A1066) in refrigerator with 4 degree overnight. Fifth, we removed the slides from the refrigerator and washed them with PBS buffer after rewarming for 45 min at room temperature. Sixth, we put the slides into the DAKO IHC instrument, and selected the corresponding procedure. Seventh, we counterstained the slides for 1 min with hematoxylin. Eighth, the slides were then immersed in 0.25% hydrochloric alcohol for about 10s. Finally, we dried the slides and sealed them. At last, the prepared sections were scanned as high-resolution digital images at 5.4 using a Pannoramic MIDI II scanner (3DHISTECH Ltd., Budapest, Hungary). The staining intensity and staining positive rate of CALR in the cytoplasm of cancer and adjacent tissues were read separately. The frequency of positive staining was assessed using a scoring system ranging from 0 to 100, where 0 indicated the absence of positively stained cells and 1-100 represented the percentage of cells stained. The staining intensity was evaluated on a scale of 0 to 3, with 0 indicating no staining, 1 indicating weak staining, 2 showing moderate staining, and 3 indicating strong staining. The final IHC scores, ranging from 0 to 300, were calculated by multiplying the frequency and intensity scores. Afterwards, the Kaplan-Meier (KM) curve was used to assess the disparity in survival between the low- and high-expression group.

### Consensus clustering analysis

In order to discern distinct molecular subtypes, consensus clustering was conducted using the “ConsensusClusterPlus” package, utilizing the expression of IRGs. The analysis was iterated 1000 times to guarantee precise and consistent clustering outcomes (22). The KM curve was utilized to visually represent disparities in OS between the ICD-high and -low group, utilizing the “survival” and “survminer” (version 4.2.0) R packages.

### Differential analysis among the ICD subgroups

Differentially expressed IRGs between the two ICD subgroups were calculated using the “limma” package, which was adjusted for *P* < 0.05 and |Log2(FC) |> 1 (23). The Gene Ontology (GO) and Kyoto Encyclopedia of Genes and Genomes (KEGG) enrichment analyses were performed using the “clusterProfiler” R package (Version 4.2.0) (24). In order to gain insight into the biological functions within the ICD-high and -low groups, we conducted the Gene Set Enrichment Analysis (GSEA) (25).

### Immune environment analysis between two ICD subgroups

To assess the proportion of immune and stromal elements in every tumor specimen, the ESTIMATE algorithm was utilized to evaluate an immune score. Subsequently, the Immune, Stromal, ESTIMATE, and TumorPurity scores were computed. Following this, CIBERSORT (26, 27) analysis, which calculated the association between the status of immune infiltration and different groups, was performed. The expression data of both subgroups of the ICD were computed utilizing CIBERSORT (HTTPS://cibersort.stanford.edu/) in order to ascertain the relative proportion of 22 immune cell types (28).

Then, we investigated the relationship between different ICD subgroups and human leukocyte antigen (HLA) genes expression levels using “limma, plyr, shape2, ggplot2, ggpubr” packages in R software. The examination of the relationship between two ICD subgroups and the levels of expression of immune checkpoint genes was conducted utilizing the R software.

### Development and validation of an ICD score prognostic model

Clinical data from 410 BLCA samples was acquired using the TCGA. The series Matrix Files and Platforms of 256 BLCA samples of GSE13507 (GPL6102) were taken from the Gene Expression Omnibus (GEO) database (https://www.ncbi.nlm.nih.gov/geo/) (**Supplementary Table 3**). The training cohort consisted of BLCA samples obtained from the TCGA dataset, while the validation cohort comprised BLCA samples obtained from the GEO dataset.

The DEGs with significant effects on the prognosis was obtained using a univariate cox regression with *P* < 0.01.* A* LASSO Cox regression analysis was conducted using the “glmnet” R package (Version 4.2.0) to determine the extent of gene selection. A formula was employed to compute the IRG score: IRG score = ∑ (Expi ∗ Coefi). Patients with BLCA were categorized into high- and low-risk groups according to their median risk scores. The KM plots were utilized to assess the survival of the two risk score groups. The GSE13507 cohort was considered as an external validation dataset. The risk curve was conducted to assess the prognostic prediction of patients with BLCA. Furthermore, univariate and multivariate Cox regression analyses were conducted to examine the risk score as a standalone prognostic indicator.

### Independence evaluation of the risk model

The R package ‘rms’ was utilized to create a nomograph model that integrates clinicopathological characteristics and risk score. The Cox regression analysis was used to compute factors and predict the chances of patient survival at intervals of 1, 3, and 5 years. The precision of the nomogram was assessed by means of a calibration graph and consistency index (C index). The C index serves as a measure of accuracy for the nomogram, indicating a positive correlation. The predictive ability of the risk model, built on the risk score, was confirmed through time-dependent receiver operating characteristic (ROC) analysis utilizing the R software package ‘timeROC’.

### Response to immunotherapy

To further evaluate the tumor-immune microenvironment in the different subgroups, We used CIBERSORT to identify the tumor-infiltrating immune cells (TIICs) and the Tumor Immune Dysfunction and Exclusion (TIDE) tool (29) to predict the response of BLCA patients to immunotherapy.

### Statistical analysis

All statistical analyses were performed using the R (version 4.2.0) software and related R packages in the bioinformatic analysis section. An adjusted P value <0.05 indicated a statistically significant difference. The OS between the low and high ICD risk cohort was compared using KM analysis, employing the survminer and survival packages in the R programming language. The prospective prognostic indicators were identified through Univariate Cox analysis, while the assessment of the risk score as an independent risk factor for OS in BLCA was conducted using multivariate Cox analysis. In the IHC analysis section, a two-tailed unpaired t-test was used to examine the differences between the two variables. The log-rank (Mantel–Cox) test was used to compare the survival curves. A value of *p* < 0.05 was considered statistically significant. GraphPad Prism software (V. 8.0) was used for data management and statistical analyses.

## Results

### Different expression of IRGs in the TCGA cohort

**Figure 1:**
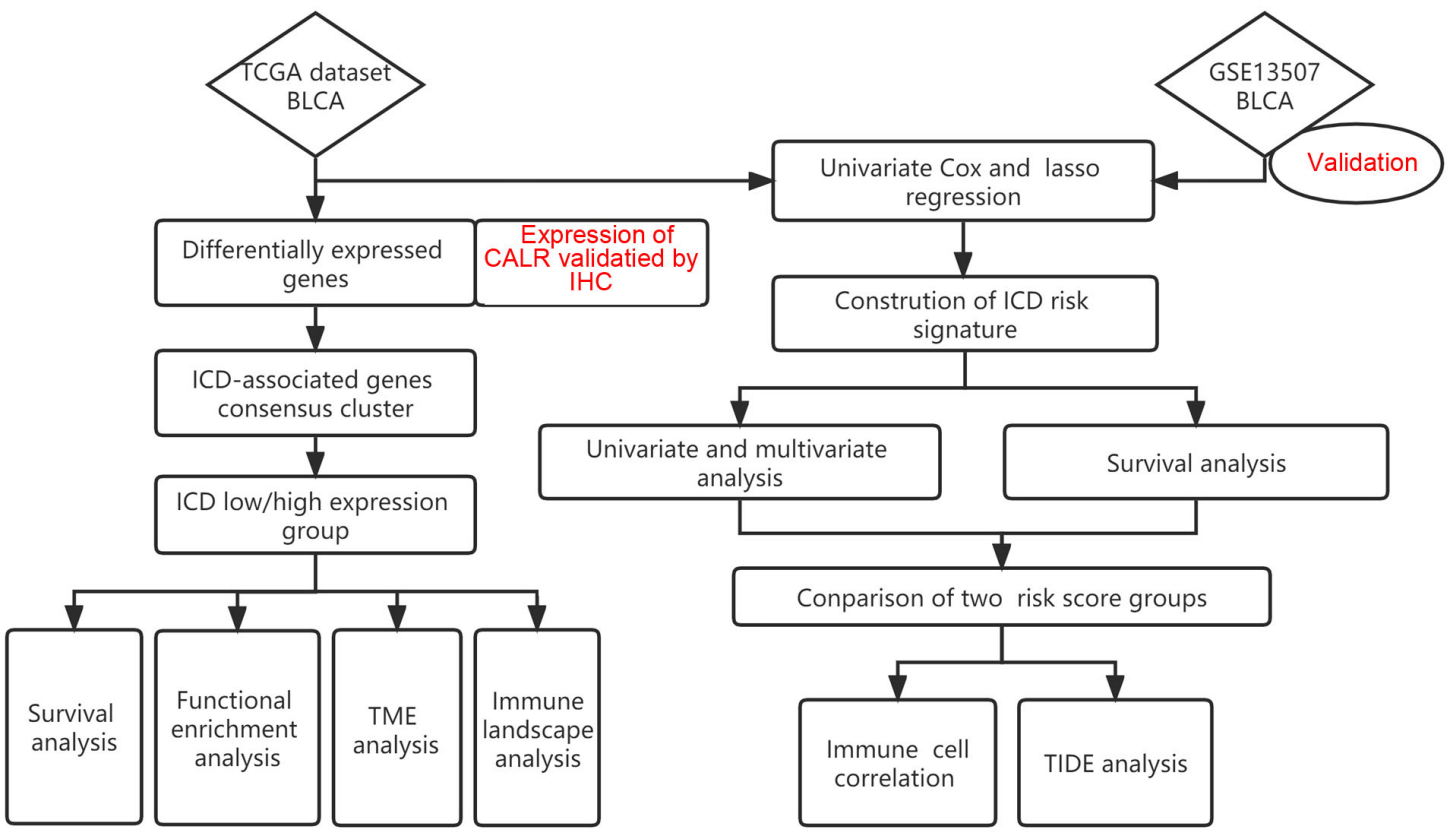
Shows a flow chart of the study.

**Figure 2:**
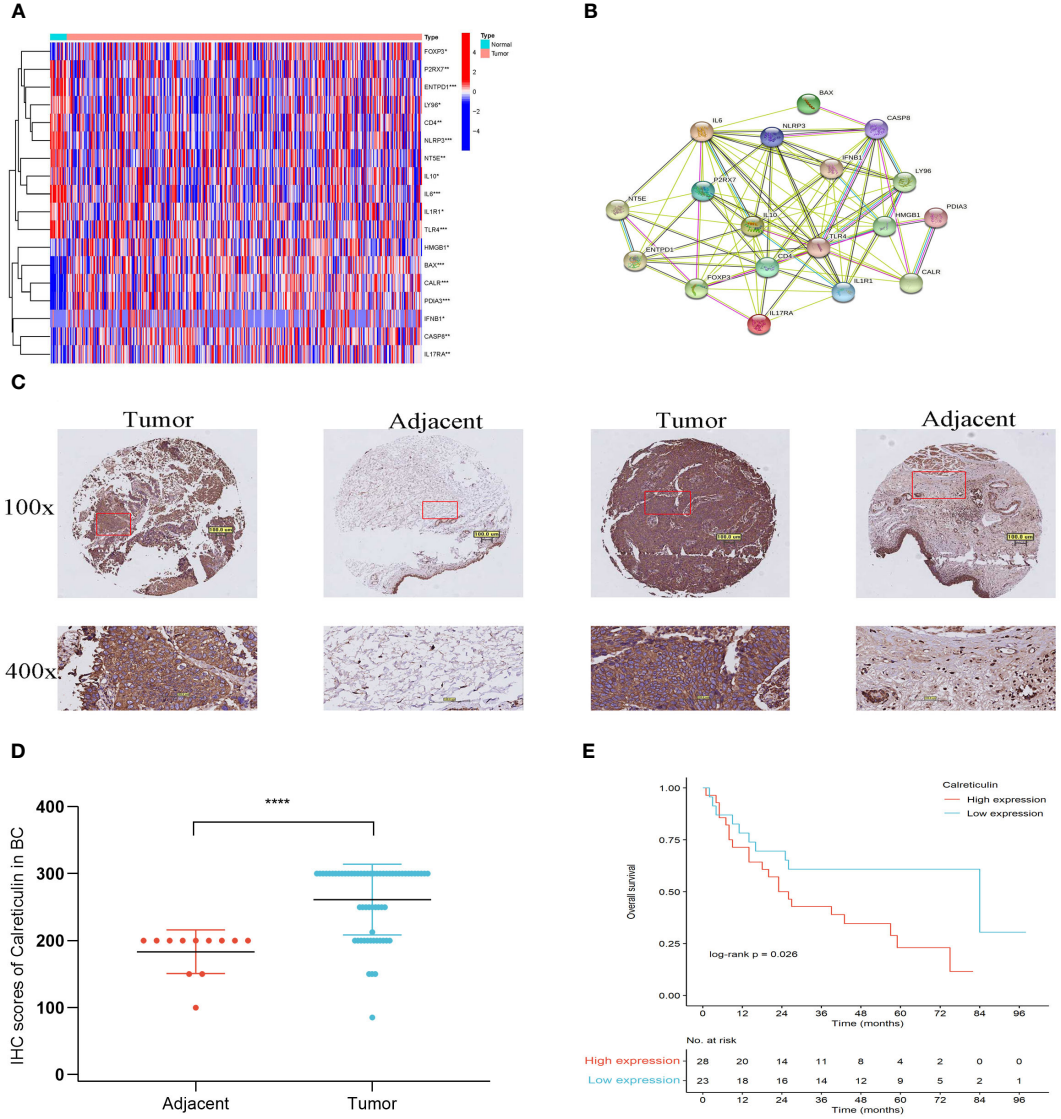
Different expressions of IRGs in the TCGA cohort. **(A)** Different expression of IRGs in tumor and normal tissues in BLCA. **(B)** PPI of the different expressions of IRGs. **(C)** IHC staining of Calreticulin expression from a tissue microarray of BLCA patients (100×: scale bar, 100 μm; 400×: scale bar, 50 μm). **(D)** IHC scores of Calreticulin expression in unpaired BLCA tissues and adjacent tissues from the tissue microarray. BLCA tissues (num = 63) and adjacent tissues (num = 16). **(E)** The overall survival of patients with BLCA in low-expression Calreticulin group and in high-expression Calreticulin group. *P<0.05, **P<0.01, ***P<0.001, ****P<0.0001.

**Figure 1** illustrated the flow of our research. Most of the 34 IRGs (**Supplementary Table 4**) (21) were upregulated in tumor samples (**Figure 2A**). The PPI network showed the interrelated relationships between these IRGs (**Figure 2B**). *CALR* was identified as one of the ICD genes. Calreticulin (CALR) is an endoplasmic reticulum -resident protein and exerts influence on numerous essential physiological processes, such as protein folding, calcium homeostasis, cellular adhesion, motility, antigen presentation, and the transmission of danger signals (30). The intracellular roles of CALR, serving as a crucial controller of Ca2+ homeostasis and integrin-dependent signaling, may be imperative for advancing certain tumors (30). Research has indicated that elevated CALR levels detected in diagnostic biopsies have been linked to unfavorable prognostic outcomes in certain groups of patients (31–34). The adverse prognostic effect of strong CALR expression in certain oncological contexts may stem from the compensatory upregulation of CD47 (35). In our study, we observed CALR was mainly expressed in the cytoplasmin both BLCA tissues and adjacent tissues (**Figure 2C**). Its staining intensity in BLCA tissues was significantly higher than that in adjacent tissues (**Figure 2C**). In the unpaired BLCA samples, compared to adjacent tissues, carcinoma tissues expressed higher levels of CALR (**Figure 2D**). And the high-expression CALR group had significantly worse OS than the low-expression group (**Figure 2E**). Patient clinical information used in the TCGA, HBlaU079Su01, and GEO cohorts was presented in **Supplementary Tables 1**–**3**, respectively.

### Typing and grouping of IRGs

**Figure 3:**
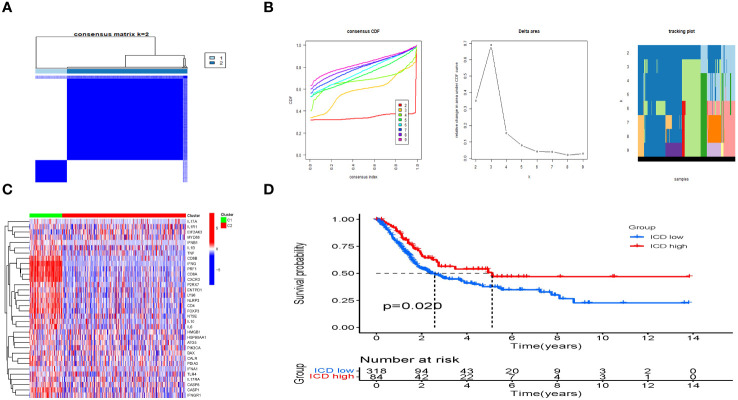
Consensus clustering to identify ICD-associated subtypes. **(A)** Heatmap of 34 genes in BLCA clustered by consensus clustering (k = 2). **(B)** Consensus clustering delta area curves show changes in area under cumulative distribution function curve for k = 2 to 10. **(C)** The heatmap shows the expression of 34 IRGs in different subtypes, with red representing high expression and blue representing low expression. **(D)** The KM curves of OS for ICD-highs and -lows.

Based on the levels of IRGs expression, the samples were categorized into two isoform categories (C1 and C2) using consensus clustering (**Figures 3A, B**; **Supplementary Table 5**). Most IRGs were upregulated in the C1 isoform (**Figure 3C**). Afterwards, the IRGs in the C1 and C2 subtypes were divided into the ICD-high and ICD-low categories, respectively. Significantly, the ICD-high group demonstrated a notable rise in survival probability when compared to the ICD-low group, as illustrated in **Figure 3D**.

### The functional and pathway analyses in two subgroups

**Figure 4:**
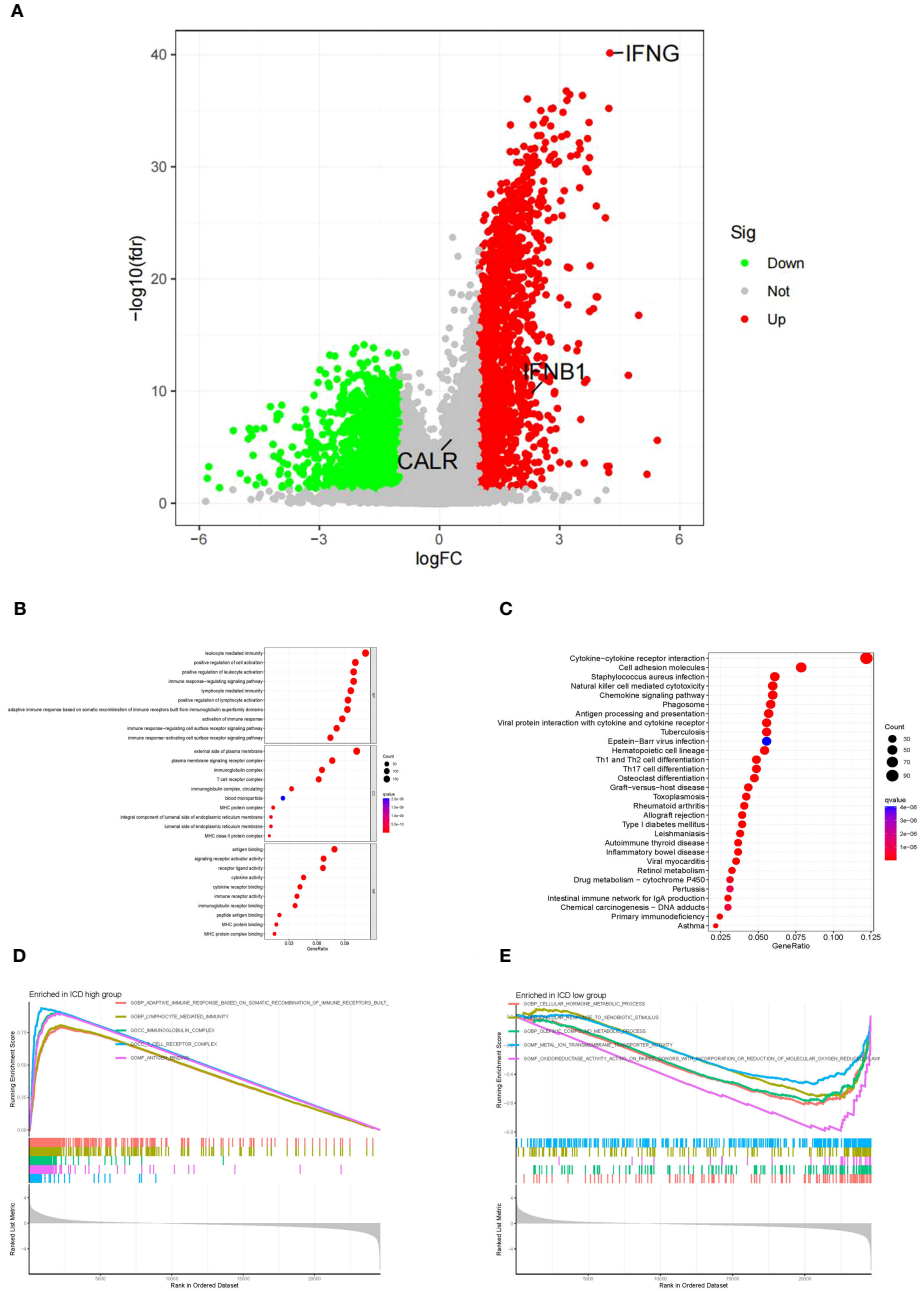
The functional and pathway analysis in two subgroups. **(A)** Volcano plot of all DEGs between two subgroups. Red = upregulated DEGs; Black = non-significant genes; Green = downregulated DEGs. **(B)** GO enrichment analysis, including biological process analysis, cellular component analysis, and molecular function analysis. **(C)** KEGG pathway analysis. **(D)** The terms enriched in the ICD high group. **(E)** The terms enriched in the ICD low group.

To further explore the mechanisms causing the prognostic differences between the two subgroups, we conducted functional and pathway analyses in 3305 DEGs (**Supplementary Table 6**) using the “limma” package. The ICD-high group showed upregulation of fifty genes (**Supplementary Table 7**), such as *IFNG*, and *IFNB1*, as displayed in the volcano map (**Figure 4A**). However, no significant difference was observed in *CALR* expression level between the ICD-high and -low groups (**Figure 4A**). In the GO analysis, it was found that leukocyte-mediated immunity, T-cell receptor complex, and antigen binding were enriched (**Figure 4B**), whereas the KEGG enrichment analysis primarily indicated involvement of cytokine-cytokine receptor interaction, natural killer cell-mediated cytotoxicity, and chemokine signaling pathway (**Figure 4C**). This showed that the IRGs regulated the tumor immune microenvironment. Next, we delved deeper into the biological functionalities within both risk score categories by employing GSEA. In the ICD-high group, we discovered a greater enrichment of immune-related pathways, such as lymphocyte-mediated defense, immunoglobulin complex, and T-cell receptor complex (**Figures 4D**). **Figures 4E** showed that the ICD-low group engaged in cellular hormone metabolic process, cellular response to xenobiotic stimulus, and olefinic compound metabolic process.

### Comparison of the immune landscape between both subgroups

**Figure 5:**
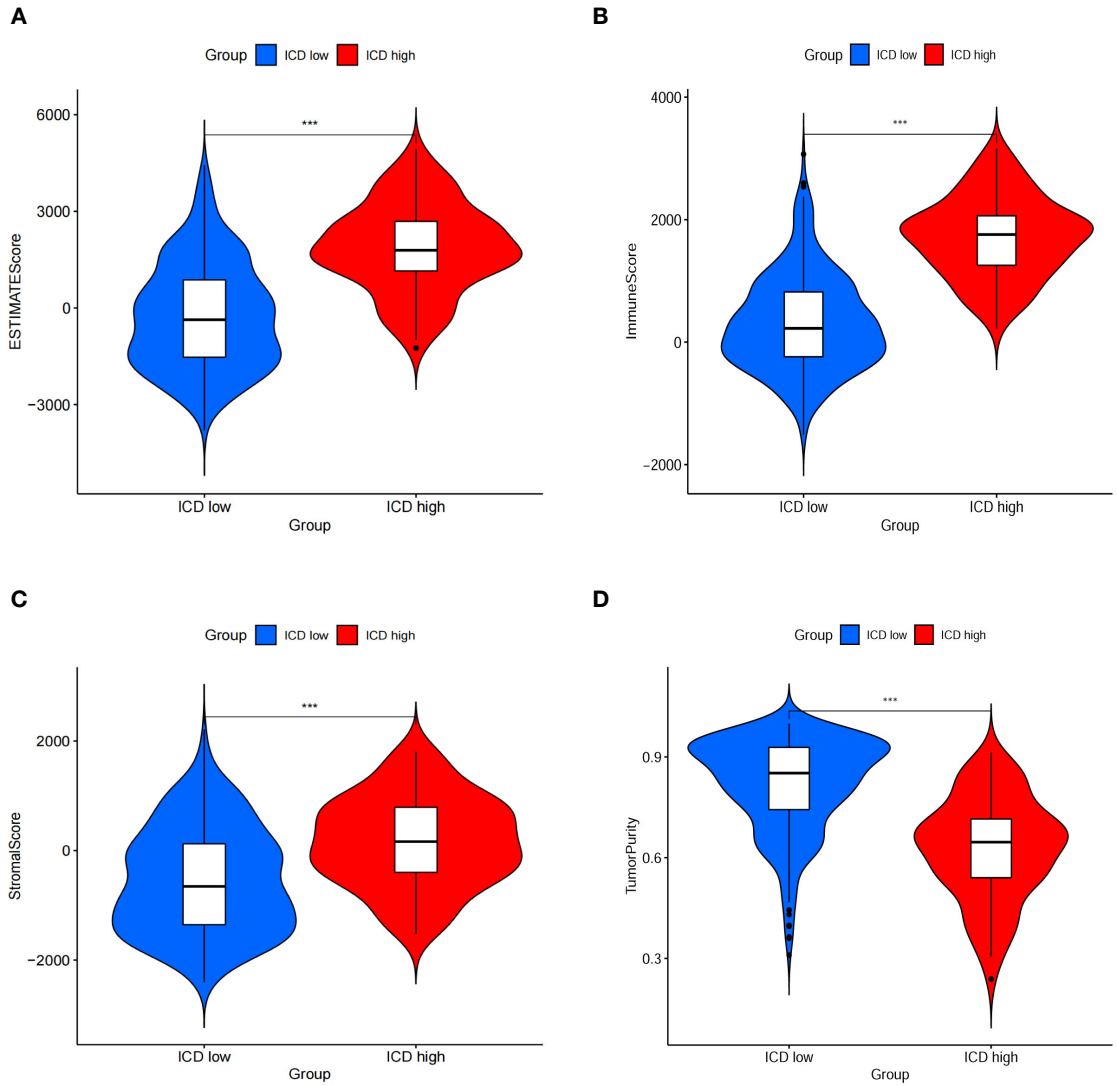
Immune scores between ICD-low group and ICD-high gorup. **(A–D)** Stromal, immune, ESTIMATE, and purity scores in the subgroup.

**Figure 6:**
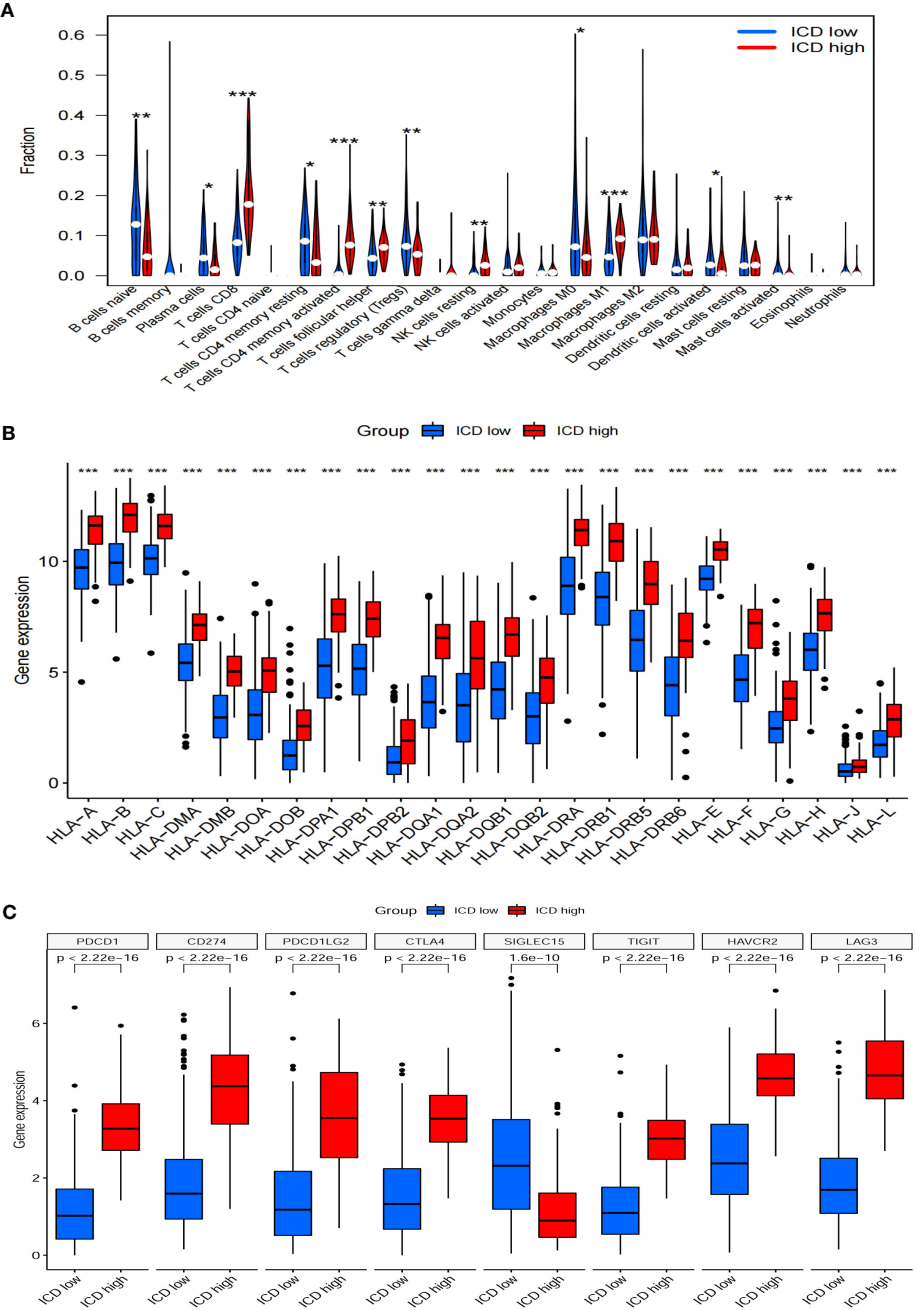
Immune landscapes between ICD-low group and ICD-high gorup. Bar plots illustrating the proportion of 22 different types of TIICs **(A)**, HLA genes expression levels **(B)**, immune checkpoint gene expression levels **(C)** between ICD-high group and ICD-low group in the TCGA cohort. *P<0.05, **P<0.01, ***P<0.001

The ICD-high group exhibited increased stromal, immune, and ESTIMATE scores, while the tumor purity score decreased (**Figures 5A–D**). Consequently, these patients may exhibit heightened immune activity and robust anti-tumor immunity. Hence, the CIBERSORT algorithm was used to determine the infiltration patterns of 22 immune cells. The results showed that people in the ICD-high category exhibited higher percentages of CD8 T-lymphocytes, activated CD4 memory T-lymphocytes, T-helper cells in the follicles, inactive natural killer cells, and M1 macrophages (**Figures 6A**). Moreover, the ICD-high group demonstrated a notable rise in the expression levels of all HLA genes (**Supplementary Table 8**), as depicted in **Figure 6B**. The expression levels of eight immune checkpoint genes, including *CD274*, *CTLA4*, *HAVCR2*, *LAG3*, *PDCD1*, *PDCD1LG2*, *TIGIT*, and *SIGLEC15*, were calculated in different ICD subgroups. The ICD-high group demonstrated a notable rise in the expression levels of *CD274*, *CTLA4*, *HAVCR2*, *LAG3*, *PDCD1*, *PDCD1LG2*, and *TIGIT* (**Figure 6C**). However, the ICD-low group demonstrated a notable rise in the expression level of *SIGLEC15* (**Figure 6C**).

### Construction and validation of an ICD-related prognostic gene model

**Figure 7:**
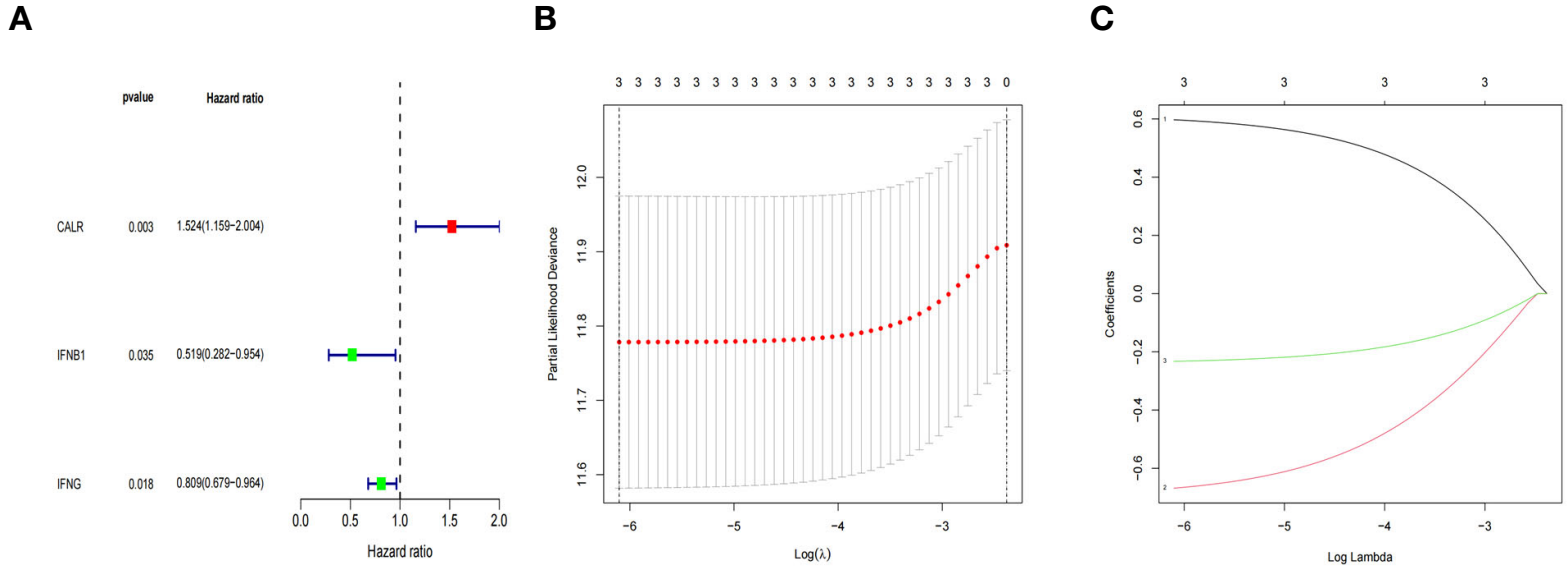
ICD-related prognostic gene model. **(A)** The IRGs with prognostic values were identified using a univariate Cox proportional hazards regression model in the TCGA cohort. **(B, C)** The selection of three genes for the risk model using the LASSO analysis.

In this study, we develop a prognostic model by evaluating the prognostic characteristics of BLCA patients based on 34 IRGs. 3 ICD-related genes, including *CALR*, *IFNB1, and IFNG*, were found to be considerably linked to the OS of patients in the Cox univariate analysis (**Figure 7A**). Further, the LASSO model was applied to those three IRGs to calculate the optimal coefficient, and they were selected for follow-up research (**Figures 7B, C**). The algorithm provided below was used to determine the risk score signature: Risk score = (0.597009118354826) * *CALR* + (-0.668324769314062) * *IFNB1* + (-0.232629267444094) * *IFNG*.

**Figure 8:**
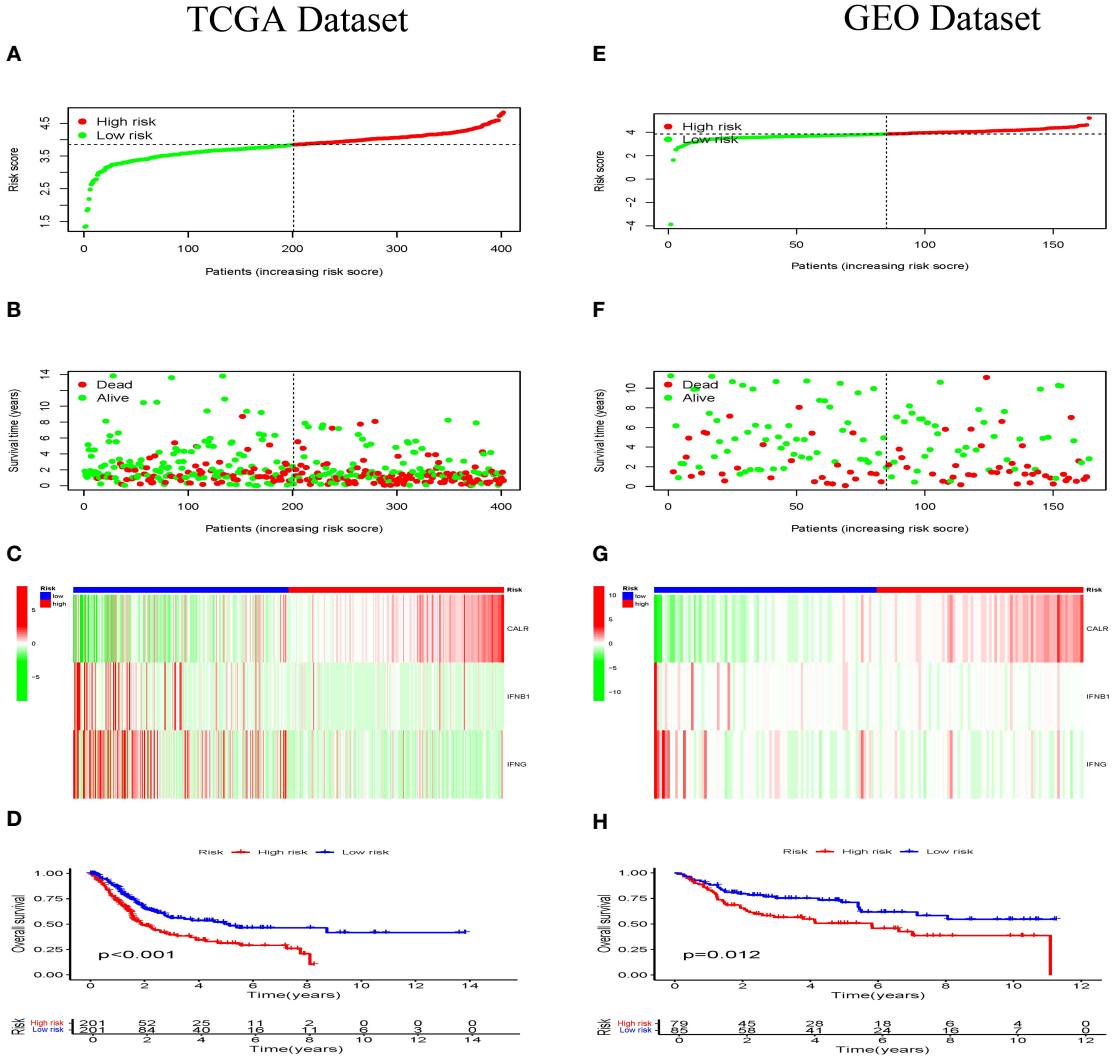
Prognosis of the risk model in TCGA dataset and GEO dataset. The distribution of risk scores in different groupings **(A, E)**, risk score and survival status **(B, F)**, heat maps for *CALR*, *IFNB1 and IFNG* expressions **(C, G)**, KM curves of BLCA patients **(D, H)** between low- and high-risk groups in the TCGA, and GEO datasets, respectively.

In addition, we investigated the relationship between survival status and risk score. In order to validate the precision and dependability of the prognostic significance associated with the IRGs-based risk score, we constructed risk models utilizing the TCGA and GEO datasets, respectively. According to their median risk scores, BLCA patients were divided into high- and low-risk categories (**Figures 8A, E**). **Figures 8B, F** showed an inverse relationship between the risk score and BLCA survival time. **Figures 8C, G** displayed distinct expressions of three IRGs in both low-risk and high-risk groups, as shown by thermographic visualization. Patients classified as low-risk in the TCGA dataset exhibited a considerably extended overall survival compared to high-risk patients, as illustrated in **Figure 8D**. The GEO cohort (**Figure 8H**) confirmed the validity of this outcome.

### Risk model based on the IRGs as an independent prognostic factor

**Figure 9:**
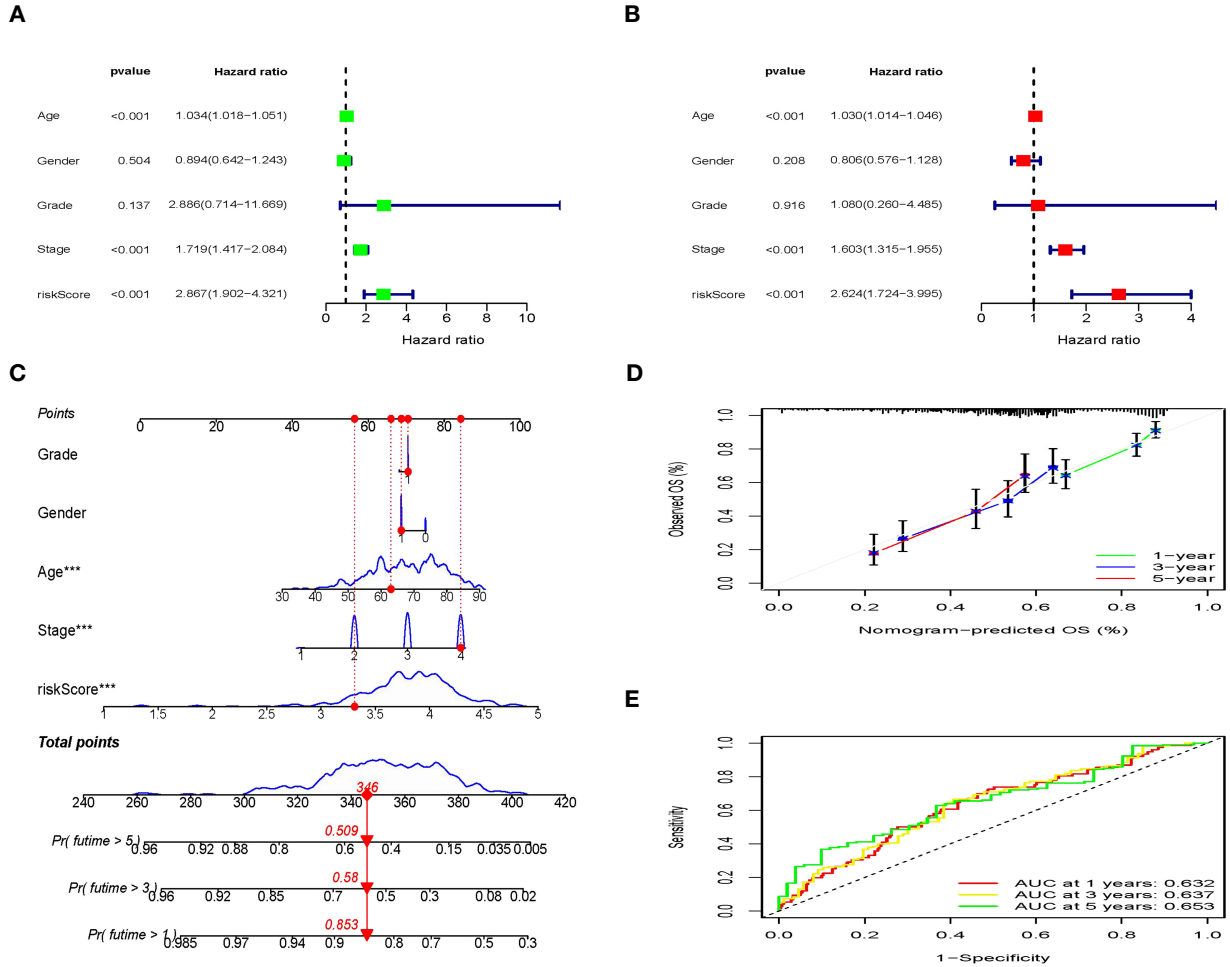
Independent prognostic analysis of clinical characteristics and risk score based on the TCGA set. **(A, B)** Screening the independent predictors for OS in BLCA using the univariate and multivariate Cox proportional hazards regression models. **(C)** Nomogram construction of risk score and linicopathological characteristics to predict the 1-, 3-, 5-years OS rate of BLCA patients. **(D)** Calibration curve shows the accuracy between predictive capacity and actual OS rate of 1-, 3-, and 5-years. **(E)** Time-dependent ROC curves indicate the area under the ROC curve (AUC) at 1-, 3-, and 5-years.

Next, risk score was examined as an independent prognostic factor of the model. Our study showed that the risk ratio (HR) of the risk score in univariate COX regression analysis was 2.867 (95% CI 1.902-4.321) (*P<*0.001) (**Figure 9A**), while the HR of risk score in multivariate COX regression analysis was 2.624 (95% CI 1.724-3.995) (*P<*0.001) (**Figure 9B**). Then we constructed a nomograph model to accurately predict the OS of BLCA patents in 1-, 3-, and 5- year according to the risk score and clinicopathological characteristics (**Figure 9C**). In addition, the calibration curve showed that the OS of 1-, 3-, and 5- year predicted by the nomogram was in satisfactory agreement with the actual OS of patients with BLCA (**Figure 9D**). The ROC values of 1-, 3-, and 5-year survival rates in the TCGA dataset were 0.632, 0.637, and 0.653 respectively (**Figure 9E**). To summarize, the prognosis model of ICD-related risk scoring evaluated results with higher accuracy and stability and demonstrated a commendable ability to accurately assess the prognosis of patients diagnosed with BLCA.

### Association between risk scores with TIICs, and the response to immunotherapy

**Figure 10:**
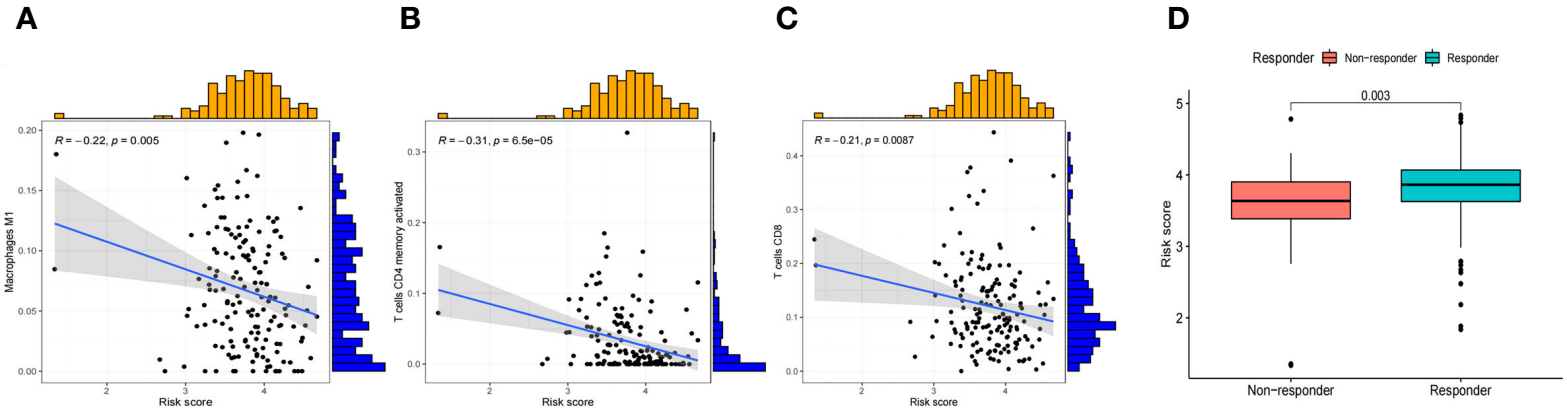
Relationship between the ICD-related signature and immune cell infiltration. **(A–C)** The infiltration levels of immune cell types in the two risk groups. **(D)** Comparisons of the response of the risk score to immunotherapy.

In the low-risk group, there was a higher presence of infiltrating activated memory CD4+ T-cells, CD8+ T-cells, and macrophages M1 (**Figures 10A–C**). Patients who had high-risk scores demonstrated markedly elevated TIDE scores (*P*=0.003) and a less favorable reaction to immunotherapy (**Figure 10D**). Consequently, this could offer groundbreaking perspectives for tailoring personalized and accurate medical treatments for BLCA patients belonging to diverse risk groups in upcoming clinical environments.

## Discussion

Bladder cancer is responsible for being the 10th leading cause of cancer-related deaths (1). ICIs have changed the natural history of genitourinary cancer treatment (36). Implementing ICIs yielded a significantly longer OS in patients diagnosed with advanced renal-cell carcinoma (37). The latest findings from the KEYNOTE-564 trial also provided further evidence of endorsing pembrolizumab monotherapy as the adjuvant treatment protocol for individuals with renal cell carcinoma who had an increased risk of recurrence after nephrectomy (38). ICD is a controlled mechanism of cellular demise that stimulates innate and adaptive immune reactions via the liberation of DAMPs. Furthermore, when combined with immunotherapy, particularly ICIs, it has the potential to counteract the immunosuppressive milieu within tumors (39). Timely identification and categorization of risk factors can enhance the immunotherapeutic regimens outcome of BLCA (40). Given the observed impact of ICD on survival rates in various tumor types, such as lung (41), ovarian malignancies (21), and head and neck squamous cell carcinoma (20), as well as its relevance to cancer therapy (16, 42, 43), it is imperative to investigate ICD-related prognostic factors in the context of BLCA. It could be advantageous to identify ICD-related biomarkers that help distinguish BLCA patients who will benefit from immunotherapy.

In light of the heterogeneity of BLCA, we undertook a consensus cluster analysis focusing on IRGs. Consequently, patients from TCGA dataset were categorized into distinct subgroups (C1 and C2) according to variations in the expression levels of IRGs. In BLCA tumors, the majority of IRGs exhibited notably elevated expression levels in comparison to normal tissues. Additionally, a more favorable prognosis was observed in the group with high-ICD. The group classified as ICD-high exhibited elevated levels of immunescore, TIICs, HLA, and immune checkpoint genes. The findings of our study indicated that gene sets linked to the high group of the ICD were notably enriched in immune active signaling pathways. We infer that the ICD-high group belongs to the immuno-hot type, while the ICD-low group belongs to the immuno-cold type. The findings indicated that the ICD-high group may be more responsive to immunotherapy than the ICD-low group, which could help us in achieving a better prognosis.

As TIICs are also an important part of TME (44), their changes can affect the biological properties of tumors. Higher rates of infiltration of immune cells into tumor tissues are essential for effective immunotherapy (45). The findings of extensive clinical studies have revealed that immune infiltration, such as CD8+ cytotoxic T-lymphocytes, Th1 and Th17 CD4+ T-cells, and M1 macrophages possess autonomous prognostic value in various cancer types (46–48). On the other hand, elevated levels of intratumoral CD4+CD25+FOXP3+ regulatory T-cells, Th2 CD4+ T-cells, and M0 macrophages have consistently been linked to an unfavorable prognosis (49). Sharma P et al. indicated that the existence of CD8 T cells infiltrating in muscle-invasive urothelial tumor, potentially indicating a reaction to particular tumor antigens, was associated with a more favorable prognosis for patients (50). Prior studies have also looked at the impact of the TME on clinical outcomes and treatment response in patients with bladder cancer, particularly the role of TIICs, which significantly affected tumor progression and treatment efficacy (51, 52). Hence, we further investigated the extensive attributes of the TIICs in BLCA. Our study revealed that individuals in the ICD-high category displayed increased levels of CD8+ T-cells, T-cells CD4 memory-activated cells, and macrophage M1 cell infiltration in BLCA. Furthermore, the infiltration of these aforementioned cells demonstrated a negative correlation with the risk score derived from our model. Conversely, the ICD-low group displayed increased levels of T-cells CD4 memory resting cells and mast cell infiltration. Previous research has provided evidence supporting the association between the presence of resting memory CD4+ T-cells and the prognostic outcomes of bladder cancer (53). Memory CD4+ T-cells exhibit elevated quantities and accelerated effector function upon reinfection, in contrast to naive T-cells (54). Mast cells, recognized as tissue-resident sentinel cells, have garnered prominence for their ability to stimulate angiogenesis and inflammation, thereby assuming a crucial role in modifying the TME. The promoting or inhibitory effect of mast cells in tumors depends on local stromal conditions (55). Crivellato et al. reviewed on the ability of tumor-infiltrating mast cells to produce and release highly potent angiogenic factors, such as vascular endothelial growth factor (56). Multiple studies have demonstrated that tumor-infiltrating mast cells in urologic malignancies exhibit a potential correlation with tumor microvessel density and play a role in facilitating tumor angiogenesis (57, 58). Consistent with their proinflammatory role, mast cells have been documented to possess a robust ability to attract additional immune cells to the TME, including neutrophils, macrophages, and eosinophils (59). The capacity of mast cells to influence the TME has garnered attention for investigating its potential prognostic and predictive significance in bladder cancer (60).

HLA genes are the most polymorphic in the human genome and are essential for regulating specific immunity (61). HLA molecules play a vital role in initiating and controlling immune responses by aiding in the presentation of peptides derived from mutated neoantigens or tumor-associated antigens to cytotoxic T-cells. According to the reported findings, HLA has demonstrated its potential as a prognostic marker for HNSCC by not only indicating enhanced tumor antigen presentation but also accurately predicting improved survival rates (62). HLA can be used as an independent biomarker for immune checkpoint blocker therapy. Chowell et al. reported a positive association between class I HLA allele diversity and clinical benefits in an ICB-treated melanoma cohort (63). Our study found that the levels of HLA in the BLCA ICD-high group were considerably higher than those in the ICD-low group. Additionally, the former group exhibited a more favorable prognosis. This illustrated that stratified management of IRGs could be a better strategy to select the BLCA immunotherapy population. Additionally, we discovered that the ICD-associated characteristics of the genes exhibited strong performance in predicting the effectiveness of immune checkpoint therapy. Tumor cells activate immune checkpoints by releasing several substances. This will prevent the antigens from being submitted to the T-cells and lead to no immunological response from the T-cells (64). In the group with high levels of ICD, the majority of immune checkpoints showed a significant increase in expression levels.Therefore, ICIs targeting these checkpoints may be better treatments for these patients.

In our analysis, 3 of the 34 ICD-related genes were considerably linked to the prognosis of BLCA patients, including *CALR*, *IFNB1*, and *IFNG*. Following this, we developed a risk model utilizing these three IRGs to assess its efficacy in prognosticating patient outcomes in BLCA. Furthermore, we explored the correlation between the prognosis model and the immune microenvironment, along with its potential implications for immunotherapy. The three genes (*CALR*, *IFNB1*, and *IFNG*) were involved in anti-tumor immunity, which improved the predictive performance of this signature. Studies conducted previously have discovered that individuals with BLCA exhibit a greater expression of CALR in both tumor tissues and urine compared to healthy individuals (65, 66). The findings from our experiment align with this observation. CALR is a highly conserved chaperone protein and plays a crucial role in various physiological and pathological processes (67). Notably, it exerts an influence on transcriptional activity and the modulation of gene expression (68). Therefore, the multifunctionality of CALR renders it a significant factor in the pathogenesis of diverse diseases, encompassing cancer and autoimmune disorders (30, 69, 70). The findings of our study indicated a significant correlation between elevated CALR expression in BLCA and unfavorable prognosis. In **Figure 4A**, there was no significant difference observed in *CALR* expression level between the ICD-high and -low groups. However, our study revealed that the ICD-high group exhibited improved response to immunotherapy. This finding suggests that the elevated *CALR* expression in BLCA tissues may contribute to the unresponsiveness of BLCA patients to immunotherapy and their overall poor prognosis. However, additional investigation into the fundamental molecular mechanisms is necessary. As *IFNB1* has both direct antiangiogenic and anti-tumor effects, it can stimulate immune production (71). It was recently reported that an IFNB1-expressing blister stomatitis virus was able to create a “comfortable” TME for immune checkpoint suppression, which promotes the antitumor immune response (72). It is a pleiotropic cytokine with a more pronounced immunomodulatory effect than its antiviral activity (73). *IFNG* is the secreted cytokine and is an important modulator of immunity and inflammation (74). IFNG signaling is vital in the immune response to tumors, and its activation is linked to the effectiveness of checkpoint-blocking therapy (75). In **Figure 4A**, the ICD-high group showed upregulation of *IFNG* and *IFNB1*. This finding aligns with our subsequent analysis, which demonstrates a positive correlation between these two genes and a favorable prognosis in patients with BLCA (**Figure 7A**). Additionally, we observed that the responders to immunotherapy exhibited lower-risk scores compared to the non-responders, indicating that our patients had a more favorable response to immunotherapy.

To guarantee comprehensive validation and broad applicability of the prognostic signature, two cohorts were chosen for analysis. These cohorts include the development cohort obtained from TCGA and the external validation cohort acquired from GEO. The three-gene pattern played a vital part in differentiating patients into subgroups with low and high risks. Remarkably, our signature consistently demonstrated satisfactory performance in both cohorts, as evidenced by the clear discrimination between risk subgroups, the unfavorable prognosis for patients in the high-risk category, and the superior predictive significance of genes related to the immune system.

In this study, we have substantiated the reliability and independence of risk score as a prognostic biomarker for BLCA. The robustness and precision of the risk model were demonstrated by the favorable ROC values for 1-, 3-, and 5-year survival rates. Furthermore, the incorporation of the risk score alongside other clinicopathological parameters in the development of a nomogram yielded significantly higher AUC values in the ROC curves compared to single-factor variables such as age, risk score, age, and stage. The calibration graph showed that the estimated curve closely resembled the perfect curve, indicating that the created nomogram had the ability to improve the predictive capability and precision for individuals with BLCA. Consequently, the 3-gene signature panel established in our investigation exhibits robust prognostic capabilities for BLCA. In the end, our investigation uncovered notable statistical differences in the tumor immune microenvironments linked to signatures and their capacity to forecast the response to immunotherapy in subgroups classified as low- and high-risk.

Nevertheless, there were certain constraints in our research. Given the retrospective nature of this study, it is crucial to carry out future prospective studies with larger sample sizes to confirm these findings. Our study has experimentally validated the *CALR* signature. However, further augmentation of relevant data in the immunotherapy cohort is necessary in subsequent investigations.

## Conclusions

By analyzing IRGs, this study effectively distinguished two distinct phenotypes for BLCA and provided a comprehensive understanding of the tumor immune microenvironment differences between them. Additionally, we also developed prognostic models of risk features that were closely related to the BLCA immune response. Therefore, we are of the opinion that our findings may offer valuable perspectives on the personalized treatment of patients with BLCA, aiding in the selection of suitable individuals for better immunotherapy response.

## Data availability statement

The datasets presented in this study can be found in online repositories. The names of the repository/repositories and accession number(s) can be found in the article/**Supplementary Material**.

## Ethics statement

Ethical approval and participants' written informed consent were not required for this study as the datasets used can be found in online repositories. The study was conducted in accordance with the local legislation and institutional requirements.

## Author contributions

LC: Conceptualization, Data curation, Formal Analysis, Funding acquisition, Investigation, Validation, Writing – original draft. JL: Data curation, Visualization, Writing – original draft. YW: Data curation, Software, Visualization, Writing – review & editing. YC: Conceptualization, Funding acquisition, Project administration, Writing – review & editing. CC: Conceptualization, Formal Analysis, Funding acquisition, Project administration, Writing – review & editing.
