# Supplementary material for: Development and validation of a model based on immunogenic cell death related genes to predict the prognosis and immune response to bladder urothelial carcinoma
Source: Front Oncol. 2023 Nov 10;13:1291720. doi: 10.3389/fonc.2023.1291720 (PMC10676223; doi:10.3389/fonc.2023.1291720)
Supplement: Supplementary file 1 [file Table_1.docx]

**Supplementary Table 1 Clinical features of all BLCA patients in TCGA cohort**

| Id | Survival day | Overall survival | Age | Gender | Grade | Stage | T | M | N |
| --- | --- | --- | --- | --- | --- | --- | --- | --- | --- |
| TCGA-GU-AATP | 1003 | Survival | 74 | Male | High Grade | Stage IV | T2 | MX | N2 |
| TCGA-CF-A1HR | 389 | Survival | 62 | Male | High Grade | Stage III | T3 | M0 | N0 |
| TCGA-GV-A3QK | 832 | Survival | 56 | Female | High Grade | Stage IV | T4a | M0 | N2 |
| TCGA-XF-A9SJ | 98 | Death | 83 | Male | High Grade | Stage IV | T3b | MX | N2 |
| TCGA-FD-A6TB | 572 | Survival | 82 | Male | High Grade | Stage III | T3a | MX | N0 |
| TCGA-FD-A5C0 | 550 | Death | 61 | Male | High Grade | Stage IV | T3a | MX | N2 |
| TCGA-XF-AAMX | 206 | Death | 87 | Female | High Grade | Stage III | T3b | MX | N0 |
| TCGA-E7-A6ME | 731 | Survival | 75 | Male | High Grade | Stage II | T2b | M0 | NX |
| TCGA-FD-A3SS | 391 | Death | 66 | Male | High Grade | Stage IV | T4 | MX | N3 |
| TCGA-DK-A3WW | 633 | Survival | 57 | Male | High Grade | Stage III | T3 | M0 | N0 |
| TCGA-FD-A3SM | 547 | Death | 70 | Male | High Grade | Stage IV | T3a | M1 | N2 |
| TCGA-UY-A78K | 536 | Death | 60 | Male | High Grade | Stage IV | unknow | MX | N2 |
| TCGA-XF-AAMQ | 2177 | Survival | 59 | Female | High Grade | Stage II | T2a | MX | N0 |
| TCGA-UY-A9PD | 542 | Survival | 80 | Male | High Grade | Stage III | T3a | MX | N0 |
| TCGA-MV-A51V | 410 | Survival | 75 | Male | High Grade | Stage III | T3a | M0 | N0 |
| TCGA-GV-A6ZA | 691 | Survival | 54 | Male | High Grade | Stage II | T2b | MX | N0 |
| TCGA-R3-A69X | 433 | Survival | 70 | Male | High Grade | Stage III | T3a | M0 | N0 |
| TCGA-FJ-A3Z7 | 945 | Survival | 76 | Male | High Grade | Stage IV | T4a | MX | N2 |
| TCGA-E7-A7XN | 428 | Survival | 66 | Male | High Grade | Stage III | T3 | M0 | N0 |
| TCGA-UY-A9PB | 899 | Survival | 49 | Male | High Grade | Stage III | T3a | MX | N0 |
| TCGA-GU-AATO | 324 | Death | 75 | Male | High Grade | Stage IV | T4a | MX | N2 |
| TCGA-ZF-A9R2 | 642 | Survival | 75 | Male | High Grade | Stage II | T2b | M0 | N0 |
| TCGA-DK-AA6U | 578 | Survival | 64 | Male | unknow | Stage II | unknow | M0 | N0 |
| TCGA-KQ-A41O | 1538 | Survival | 84 | Male | High Grade | Stage IV | T3 | M0 | N1 |
| TCGA-ZF-A9R9 | 864 | Death | 58 | Male | High Grade | Stage IV | T3b | MX | N2 |
| TCGA-FD-A62S | 406 | Death | 60 | Female | High Grade | Stage III | T3b | MX | N0 |
| TCGA-XF-AAN2 | 1869 | Death | 73 | Male | High Grade | Stage II | T2b | MX | N0 |
| TCGA-GV-A3JW | 649 | Survival | 74 | Male | High Grade | Stage II | T2 | MX | NX |
| TCGA-CF-A47X | 384 | Survival | 60 | Male | Low Grade | Stage II | T2 | M0 | N0 |
| TCGA-FD-A43Y | 474 | Death | 65 | Male | High Grade | Stage III | T4 | MX | N0 |
| TCGA-BT-A20V | 154 | Death | 59 | Female | High Grade | Stage IV | T4a | M0 | N2 |
| TCGA-DK-A2HX | 1420 | Death | 80 | Female | High Grade | Stage IV | T3 | M0 | N2 |
| TCGA-S5-A6DX | 56 | Death | 83 | Male | High Grade | Stage IV | T4a | MX | N2 |
| TCGA-GC-A3YS | 758 | Survival | 61 | Male | High Grade | Stage IV | T3a | MX | N1 |
| TCGA-LT-A5Z6 | 474 | Survival | 56 | Male | High Grade | Stage II | unknow | MX | NX |
| TCGA-CU-A3KJ | 562 | Survival | 75 | Male | High Grade | Stage III | T3b | M0 | N0 |
| TCGA-CU-A72E | 413 | Death | 76 | Male | High Grade | Stage IV | T3b | M0 | N2 |
| TCGA-FD-A5BY | 251 | Survival | 63 | Female | High Grade | Stage III | T3a | MX | N0 |
| TCGA-CF-A8HY | 345 | Survival | 59 | Male | High Grade | Stage II | T2 | M0 | N0 |
| TCGA-C4-A0F1 | 89 | Survival | 71 | Male | High Grade | Stage III | T3b | M0 | N0 |
| TCGA-BL-A0C8 | 1219 | Survival | 73 | Male | High Grade | Stage I | T1 | M0 | NX |
| TCGA-SY-A9G0 | 1008 | Death | 82 | Male | High Grade | Stage IV | T4 | M0 | N1 |
| TCGA-DK-A3IL | 413 | Death | 79 | Female | High Grade | Stage IV | T3 | M0 | N2 |
| TCGA-FD-A3B6 | 1005 | Death | 75 | Male | High Grade | Stage II | T2b | MX | N0 |
| TCGA-UY-A9PA | 1072 | Survival | 48 | Male | High Grade | Stage III | T3a | MX | N0 |
| TCGA-XF-A9SU | 182 | Death | 74 | Female | High Grade | Stage IV | T3b | MX | N1 |
| TCGA-E7-A85H | 394 | Survival | 64 | Male | High Grade | Stage III | T3 | M0 | N0 |
| TCGA-K4-A4AB | 76 | Survival | 76 | Male | High Grade | Stage III | T4a | MX | N0 |
| TCGA-E7-A677 | 820 | Survival | 81 | Male | Low Grade | Stage II | T2b | M0 | N0 |
| TCGA-XF-A9SK | 486 | Death | 65 | Male | High Grade | Stage IV | T3b | MX | N1 |
| TCGA-CF-A47W | 368 | Survival | 42 | Male | Low Grade | Stage II | T2 | M0 | N0 |
| TCGA-CF-A47Y | 373 | Survival | 55 | Male | Low Grade | Stage II | T2 | M0 | N0 |
| TCGA-5N-A9KI | 76 | Death | 76 | Female | High Grade | Stage III | T4 | MX | NX |
| TCGA-GV-A3JX | 581 | Survival | 59 | Male | High Grade | Stage III | T3b | MX | N0 |
| TCGA-ZF-A9RG | 1786 | Survival | 70 | Female | High Grade | Stage III | T3b | MX | N0 |
| TCGA-FD-A5BT | 328 | Death | 84 | Male | High Grade | Stage III | T3b | MX | N0 |
| TCGA-CF-A9FM | 398 | Survival | 50 | Male | High Grade | Stage I | T1 | M0 | N0 |
| TCGA-H4-A2HQ | 590 | Survival | 64 | Female | High Grade | Stage IV | unknow | M1 | NX |
| TCGA-ZF-AA5N | 168 | Death | 62 | Female | High Grade | Stage IV | T2 | M1 | NX |
| TCGA-ZF-A9RC | 2868 | Survival | 77 | Male | High Grade | Stage III | T3a | MX | N0 |
| TCGA-BL-A3JM | 205 | Death | 62 | Male | High Grade | Stage III | T3 | M0 | N0 |
| TCGA-FD-A5BR | 813 | Survival | 57 | Male | High Grade | Stage II | T2b | MX | N0 |
| TCGA-4Z-AA7R | 522 | Death | 73 | Male | High Grade | Stage IV | T4b | M0 | N0 |
| TCGA-YF-AA3M | 415 | Survival | 57 | Male | High Grade | Stage II | T2a | MX | NX |
| TCGA-ZF-A9RE | 106 | Death | 78 | Female | High Grade | Stage II | unknow | MX | unknow |
| TCGA-DK-A3IQ | 539 | Death | 74 | Male | High Grade | Stage III | T3 | M0 | N0 |
| TCGA-E7-A6MF | 750 | Survival | 37 | Male | Low Grade | Stage II | T2a | M0 | NX |
| TCGA-K4-A3WU | 105 | Survival | 87 | Male | High Grade | Stage III | T4a | MX | N0 |
| TCGA-4Z-AA7N | 1367 | Death | 65 | Male | High Grade | Stage III | T3a | M0 | N0 |
| TCGA-FD-A3SN | 887 | Survival | 79 | Male | High Grade | Stage III | T3b | MX | N0 |
| TCGA-DK-A1A5 | 65 | Death | 79 | Male | High Grade | Stage II | T2b | M0 | N0 |
| TCGA-FD-A5BS | 1639 | Survival | 68 | Male | High Grade | Stage III | T3b | MX | N0 |
| TCGA-2F-A9KQ | 2886 | Survival | 69 | Male | High Grade | Stage III | T3a | M0 | N0 |
| TCGA-E7-A3X6 | 904 | Death | 70 | Male | Low Grade | Stage II | T2 | M0 | NX |
| TCGA-K4-A54R | 842 | Survival | 59 | Male | High Grade | Stage II | T2b | MX | N0 |
| TCGA-FD-A6TE | 376 | Survival | 54 | Male | High Grade | Stage IV | T3a | MX | N2 |
| TCGA-BT-A3PH | 142 | Death | 76 | Male | High Grade | Stage IV | T3b | MX | N2 |
| TCGA-DK-AA6M | 1582 | Survival | 64 | Female | High Grade | Stage II | T2 | M0 | N0 |
| TCGA-GV-A3QF | 617 | Death | 79 | Male | High Grade | Stage IV | T3b | MX | N2 |
| TCGA-DK-A6AV | 1952 | Survival | 82 | Female | High Grade | Stage II | T2a | M0 | N0 |
| TCGA-ZF-AA4R | 1036 | Death | 67 | Male | High Grade | Stage IV | T3a | MX | N1 |
| TCGA-DK-AA6T | 572 | Survival | 63 | Male | High Grade | Stage II | unknow | M0 | N0 |
| TCGA-4Z-AA7M | 495 | Survival | 65 | Male | High Grade | Stage III | T3a | M0 | N0 |
| TCGA-BT-A20Q | 593 | Death | 73 | Male | High Grade | Stage IV | T3b | M0 | N2 |
| TCGA-G2-A2EO | 1804 | Death | 69 | Male | High Grade | Stage III | T3a | M0 | N0 |
| TCGA-DK-A1AF | 536 | Survival | 84 | Female | High Grade | Stage IV | T3 | M0 | N2 |
| TCGA-E7-A6MD | 129 | Survival | 66 | Male | High Grade | Stage IV | T4a | M0 | N1 |
| TCGA-FD-A5BX | 173 | Death | 82 | Male | High Grade | Stage IV | T3b | MX | N1 |
| TCGA-ZF-A9R4 | 921 | Survival | 83 | Male | High Grade | Stage II | T2 | M0 | NX |
| TCGA-DK-A1A7 | 560 | Survival | 67 | Female | High Grade | Stage IV | T3 | M0 | N2 |
| TCGA-XF-A9T2 | 575 | Death | 54 | Male | High Grade | Stage III | T3b | MX | N0 |
| TCGA-K4-A5RJ | 539 | Survival | 75 | Male | High Grade | Stage II | T2b | MX | N0 |
| TCGA-K4-A5RH | 276 | Survival | 69 | Male | High Grade | Stage III | T3a | MX | N0 |
| TCGA-4Z-AA7S | 1064 | Death | 66 | Male | High Grade | Stage III | T4a | M0 | N0 |
| TCGA-GU-A42P | 332 | Death | 72 | Male | High Grade | Stage IV | T3a | M0 | N1 |
| TCGA-BT-A20N | 795 | Death | 72 | Male | High Grade | Stage III | T3a | MX | N0 |
| TCGA-G2-A3IB | 220 | Death | 66 | Male | High Grade | Stage II | unknow | MX | NX |
| TCGA-XF-AAMY | 3011 | Survival | 78 | Male | High Grade | Stage III | T3b | MX | N0 |
| TCGA-XF-A9T8 | 418 | Death | 64 | Male | High Grade | Stage III | T3b | MX | N0 |
| TCGA-FD-A3B4 | 510 | Death | 55 | Female | High Grade | Stage III | T4a | MX | N0 |
| TCGA-BT-A42C | 873 | Survival | 64 | Male | High Grade | Stage II | unknow | M0 | N0 |
| TCGA-ZF-A9RF | 1949 | Survival | 74 | Male | High Grade | Stage II | T1 | M0 | NX |
| TCGA-BL-A5ZZ | 377 | Survival | 80 | Female | High Grade | Stage III | T4a | MX | N0 |
| TCGA-4Z-AA7Y | 1522 | Survival | 60 | Male | High Grade | Stage II | T2a | M0 | N0 |
| TCGA-GV-A3QI | 1110 | Survival | 47 | Male | High Grade | Stage III | T3b | MX | N0 |
| TCGA-E5-A4U1 | 1181 | Survival | 72 | Male | High Grade | Stage II | T2b | M0 | N0 |
| TCGA-DK-AA74 | 1708 | Survival | 75 | Male | High Grade | Stage III | T3 | M0 | N0 |
| TCGA-4Z-AA7W | 840 | Survival | 55 | Male | High Grade | Stage II | T2a | M0 | N0 |
| TCGA-CU-A5W6 | 56 | Death | 70 | Male | High Grade | Stage III | T4a | M0 | N0 |
| TCGA-FD-A5BU | 588 | Survival | 76 | Female | High Grade | Stage II | T2b | MX | N0 |
| TCGA-K4-AAQO | 359 | Survival | 56 | Male | High Grade | Stage III | T3a | MX | N0 |
| TCGA-4Z-AA89 | 1029 | Survival | 60 | Male | High Grade | Stage IV | T4b | M0 | N0 |
| TCGA-UY-A78M | 690 | Death | 81 | Female | High Grade | Stage IV | T2 | MX | N2 |
| TCGA-DK-A2I2 | 237 | Death | 63 | Female | High Grade | Stage IV | T3 | M0 | N3 |
| TCGA-XF-A9ST | 128 | Death | 68 | Male | High Grade | Stage III | T3b | MX | N0 |
| TCGA-4Z-AA81 | 1270 | Death | 80 | Male | High Grade | Stage II | T2b | M0 | N0 |
| TCGA-YC-A9TC | 20 | Death | 71 | Male | High Grade | Stage IV | unknow | M1 | unknow |
| TCGA-KQ-A41N | 1604 | Survival | 73 | Male | High Grade | Stage III | T3b | M0 | N0 |
| TCGA-E5-A2PC | 1326 | Survival | 61 | Female | High Grade | Stage IV | T2b | MX | N1 |
| TCGA-DK-A1A3 | 665 | Death | 60 | Male | High Grade | Stage IV | T3 | M0 | N2 |
| TCGA-XF-A8HG | 467 | Death | 69 | Male | High Grade | Stage III | T3b | MX | N0 |
| TCGA-FD-A3SL | 712 | Death | 60 | Male | High Grade | Stage IV | T4a | M1 | N2 |
| TCGA-G2-A2EJ | 1460 | Survival | 56 | Female | High Grade | Stage II | unknow | M0 | N0 |
| TCGA-FD-A3SO | 168 | Death | 68 | Male | High Grade | Stage IV | T3a | MX | N1 |
| TCGA-FD-A62P | 191 | Death | 76 | Male | High Grade | Stage II | T2b | MX | N0 |
| TCGA-FD-A43X | 110 | Survival | 84 | Male | High Grade | Stage II | T2a | MX | N0 |
| TCGA-DK-A6B6 | 1115 | Survival | 57 | Male | High Grade | Stage II | unknow | MX | NX |
| TCGA-ZF-A9R7 | 665 | Survival | 76 | Female | High Grade | Stage II | unknow | M0 | NX |
| TCGA-XF-A8HI | 544 | Death | 57 | Female | High Grade | Stage IV | T2b | MX | N2 |
| TCGA-BT-A20X | 251 | Death | 56 | Male | High Grade | Stage IV | T4a | M0 | N2 |
| TCGA-GV-A3QG |  | Death | 65 | Male | High Grade | Stage IV | T3a | MX | N3 |
| TCGA-G2-A2ES | 1004 | Death | 85 | Male | High Grade | Stage II | T3b | M0 | N0 |
| TCGA-ZF-AA4N | 88 | Death | 74 | Male | High Grade | Stage II | unknow | Mx | unknow |
| TCGA-GC-A3BM | 651 | Death | 70 | Male | High Grade | Stage II | T2b | M0 | N0 |
| TCGA-DK-AA6W | 415 | Death | 78 | Male | High Grade | Stage II | T2 | M0 | N0 |
| TCGA-E7-A5KE | 17 | Survival | 78 | Female | High Grade | Stage II | T2a | M0 | N0 |
| TCGA-UY-A78L | 1127 | Survival | 62 | Male | High Grade | Stage IV | unknow | MX | N1 |
| TCGA-E7-A97P | 437 | Death | 73 | Male | High Grade | Stage II | T2 | M0 | NX |
| TCGA-XF-A9SH | 1971 | Death | 65 | Female | High Grade | Stage II | T2b | M0 | N0 |
| TCGA-FT-A61P | 337 | Survival | 76 | Male | High Grade | Stage IV | T3b | MX | N2 |
| TCGA-CU-A0YR | 460 | Death | 83 | Male | High Grade | Stage IV | T2 | M0 | N2 |
| TCGA-DK-AA77 | 618 | Survival | 61 | Male | High Grade | Stage II | T2a | M0 | N0 |
| TCGA-CF-A9FH | -64 | Survival | 85 | Male | Low Grade | Stage II | T2 | M0 | N0 |
| TCGA-E7-A5KF | 20 | Survival | 67 | Male | Low Grade | Stage II | T2a | M0 | N0 |
| TCGA-DK-A2I6 | 2656 | Survival | 81 | Male | High Grade | Stage IV | T2b | M0 | N1 |
| TCGA-CF-A5UA | 365 | Survival | 67 | Male | Low Grade | Stage II | T2 | M0 | N0 |
| TCGA-DK-AA6X | 467 | Survival | 63 | Male | High Grade | Stage II | unknow | M0 | N0 |
| TCGA-GU-A762 | 232 | Death | 87 | Male | High Grade | Stage IV | T4a | MX | N2 |
| TCGA-E7-A541 | 778 | Death | 66 | Male | High Grade | Stage II | T2b | MX | N0 |
| TCGA-BT-A2LB | 492 | Death | 73 | Female | High Grade | Stage III | T3a | M0 | N0 |
| TCGA-XF-A9T6 | 64 | Survival | 88 | Female | High Grade | Stage III | T3b | MX | N0 |
| TCGA-DK-A3IV | 294 | Death | 60 | Male | High Grade | Stage II | unknow | M0 | NX |
| TCGA-GD-A3OP | 64 | Survival | 84 | Female | High Grade | Stage IV | T4a | MX | N2 |
| TCGA-C4-A0F7 | 62 | Death | 77 | Male | High Grade | Stage IV | T4b | M0 | N2 |
| TCGA-FD-A3B5 | 272 | Death | 86 | Male | High Grade | Stage IV | T2b | MX | N1 |
| TCGA-FD-A3SJ | 739 | Death | 59 | Male | High Grade | Stage IV | T2b | MX | N2 |
| TCGA-FD-A3SQ | 1423 | Death | 62 | Male | High Grade | Stage IV | T3a | MX | N2 |
| TCGA-FD-A43U | 636 | Survival | 70 | Male | High Grade | Stage IV | T4a | MX | N2 |
| TCGA-GV-A40E | 261 | Death | 75 | Male | High Grade | Stage II | unknow | MX | NX |
| TCGA-KQ-A41P | 1094 | Survival | 76 | Male | High Grade | Stage IV | T3b | M1 | N3 |
| TCGA-XF-AAN1 | 941 | Death | 75 | Female | High Grade | Stage III | T4a | M0 | N0 |
| TCGA-XF-A8HH | 57 | Death | 61 | Female | High Grade | Stage IV | T3b | M0 | N2 |
| TCGA-XF-A8HC | 200 | Death | 79 | Male | High Grade | Stage IV | T3a | MX | N2 |
| TCGA-C4-A0EZ | 273 | Death | 69 | Female | High Grade | Stage IV | T3a | M1 | N1 |
| TCGA-ZF-AA56 | 259 | Death | 79 | Female | High Grade | Stage III | T4a | MX | N0 |
| TCGA-UY-A78N | 2641 | Death | 80 | Male | High Grade | Stage IV | T2 | MX | N1 |
| TCGA-PQ-A6FI | 372 | Survival | 70 | Male | High Grade | Stage II | T2a | MX | N0 |
| TCGA-CU-A0YN | 393 | Death | 60 | Male | High Grade | Stage III | T3a | M0 | N0 |
| TCGA-GD-A2C5 | 812 | Survival | 53 | Female | High Grade | Stage IV | T3a | MX | N2 |
| TCGA-E5-A4TZ | 467 | Death | 64 | Male | High Grade | Stage IV | T4b | MX | N2 |
| TCGA-XF-A8HF | 2954 | Death | 80 | Male | High Grade | Stage III | T3a | M0 | N0 |
| TCGA-GU-AATQ | 213 | Death | 68 | Male | High Grade | Stage III | T3b | MX | N0 |
| TCGA-DK-A3WX | 321 | Death | 67 | Female | High Grade | Stage III | T3 | M0 | N0 |
| TCGA-G2-AA3F | 893 | Survival | 77 | Male | High Grade | Stage IV | T3 | M0 | N1 |
| TCGA-XF-AAMR | 2790 | Survival | 48 | Male | High Grade | Stage III | T3b | MX | N0 |
| TCGA-BT-A3PJ | 789 | Survival | 76 | Male | High Grade | Stage III | T3b | M0 | N0 |
| TCGA-XF-AAMH | 344 | Death | 80 | Male | High Grade | Stage IV | T3b | MX | N1 |
| TCGA-XF-AAMT | 90 | Death | 75 | Female | High Grade | Stage IV | T3b | MX | N2 |
| TCGA-CF-A47T | 385 | Death | 58 | Female | Low Grade | Stage II | T2 | M0 | N0 |
| TCGA-XF-A9SP | 454 | Death | 59 | Male | High Grade | Stage III | T3b | MX | N0 |
| TCGA-ZF-AA58 | 1649 | Survival | 61 | Female | High Grade | Stage IV | T3a | MX | N2 |
| TCGA-CF-A7I0 | 368 | Survival | 54 | Male | Low Grade | Stage II | T2 | M0 | N0 |
| TCGA-CF-A47V | 379 | Survival | 52 | Male | High Grade | Stage II | T2 | M0 | N0 |
| TCGA-XF-A9T0 | 799 | Survival | 68 | Male | High Grade | Stage III | T3b | MX | N0 |
| TCGA-G2-A3VY | 536 | Survival | 66 | Male | High Grade | Stage II | unknow | unknow | unknow |
| TCGA-BT-A20U | 455 | Death | 70 | Female | High Grade | Stage III | T3a | M0 | N0 |
| TCGA-XF-AAN5 | 2293 | Survival | 61 | Female | High Grade | Stage III | T3b | MX | N0 |
| TCGA-E7-A4IJ | 674 | Death | 56 | Male | High Grade | Stage II | T2b | M0 | NX |
| TCGA-ZF-AA4W | 1830 | Survival | 56 | Male | High Grade | Stage III | T3b | MX | N0 |
| TCGA-YC-A8S6 | 293 | Survival | 71 | Male | High Grade | Stage II | T2a | MX | N0 |
| TCGA-FD-A6TA | 1912 | Survival | 58 | Male | High Grade | Stage IV | T3b | MX | N2 |
| TCGA-UY-A8OD | 3432 | Survival | 68 | Female | High Grade | Stage II | T2b | MX | N0 |
| TCGA-GC-A3RC | 484 | Survival | 59 | Male | High Grade | Stage II | T2b | M0 | N0 |
| TCGA-4Z-AA83 | 2024 | Survival | 52 | Male | High Grade | Stage II | T2a | M0 | N0 |
| TCGA-4Z-AA7Q | 510 | Death | 79 | Male | High Grade | Stage III | T3a | M0 | NX |
| TCGA-BT-A2LA | 522 | Survival | 54 | Male | High Grade | Stage III | T3a | M0 | N0 |
| TCGA-GV-A3QH | 258 | Death | 67 | Male | High Grade | Stage II | unknow | MX | NX |
| TCGA-DK-AA6S | 5050 | Survival | 60 | Male | High Grade | Stage III | T3b | M0 | N0 |
| TCGA-SY-A9G5 | 1186 | Survival | 66 | Male | High Grade | Stage III | T4a | M0 | N0 |
| TCGA-FD-A43S | 455 | Survival | 71 | Female | High Grade | Stage III | T3b | MX | N0 |
| TCGA-BT-A2LD | 623 | Death | 78 | Female | High Grade | Stage IV | T3a | M0 | N1 |
| TCGA-GD-A3OS | 638 | Survival | 54 | Female | High Grade | Stage II | unknow | MX | NX |
| TCGA-DK-AA71 | 415 | Survival | 71 | Male | High Grade | Stage II | T2b | M0 | N0 |
| TCGA-XF-AAMF | 329 | Death | 68 | Male | High Grade | Stage IV | T4 | MX | N2 |
| TCGA-LC-A66R | 466 | Survival | 78 | Male | High Grade | Stage IV | T4a | MX | N2 |
| TCGA-2F-A9KR | 3183 | Death | 59 | Female | High Grade | Stage III | T3a | M0 | N0 |
| TCGA-CF-A27C | 425 | Survival | 52 | Male | High Grade | Stage III | T3 | M0 | N0 |
| TCGA-FD-A3SP | 783 | Survival | 60 | Male | High Grade | Stage III | T3b | MX | N0 |
| TCGA-FD-A3SR | 602 | Death | 68 | Male | High Grade | Stage IV | T4a | MX | N2 |
| TCGA-DK-AA6Q | 413 | Death | 61 | Female | High Grade | Stage IV | T3 | M0 | N1 |
| TCGA-BL-A13I | 223 | Death | 57 | Female | High Grade | Stage III | T3 | M0 | N0 |
| TCGA-BT-A42E | 1108 | Survival | 74 | Male | High Grade | Stage III | T3a | M0 | N0 |
| TCGA-E7-A519 | 508 | Survival | 72 | Male | High Grade | Stage II | T2b | M0 | NX |
| TCGA-4Z-AA7O | 512 | Survival | 64 | Male | High Grade | Stage II | T2a | M0 | N0 |
| TCGA-XF-AAN7 | 565 | Death | 60 | Male | High Grade | Stage IV | T3a | MX | N1 |
| TCGA-4Z-AA87 | 1454 | Survival | 72 | Male | High Grade | Stage III | T4a | M0 | N0 |
| TCGA-DK-A1A6 | 2020 | Survival | 53 | Male | High Grade | Stage IV | T2a | M0 | N1 |
| TCGA-XF-A9SW | 362 | Death | 85 | Male | High Grade | Stage IV | T3b | MX | N2 |
| TCGA-FD-A6TI | 294 | Death | 73 | Male | High Grade | Stage IV | T4b | MX | N1 |
| TCGA-GC-A6I1 | 0 | Survival | 90 | Female | High Grade | Stage II | T2b | MX | N0 |
| TCGA-DK-A1AA | 578 | Survival | 57 | Male | High Grade | Stage III | T3 | M0 | N0 |
| TCGA-BT-A20P | 544 | Death | 81 | Female | High Grade | Stage III | T3a | M0 | N0 |
| TCGA-XF-AAML | 232 | Death | 75 | Male | High Grade | Stage II | T2b | MX | N0 |
| TCGA-G2-AA3D | 2139 | Survival | 60 | Male | High Grade | Stage IV | T3 | M0 | N2 |
| TCGA-K4-A3WV | 646 | Survival | 77 | Female | High Grade | Stage II | T2b | MX | N0 |
| TCGA-4Z-AA84 | 460 | Survival | 61 | Male | High Grade | Stage IV | T3a | M1 | N2 |
| TCGA-ZF-A9R3 | 949 | Death | 53 | Female | High Grade | Stage II | T2 | M0 | NX |
| TCGA-DK-A1AB | 508 | Death | 74 | Female | High Grade | Stage IV | T4a | M0 | N2 |
| TCGA-XF-A8HB | 1370 | Survival | 50 | Female | High Grade | Stage II | T2b | MX | N0 |
| TCGA-K4-A4AC | 278 | Death | 83 | Male | High Grade | Stage II | T2b | MX | N0 |
| TCGA-DK-A3IS | 1529 | Survival | 68 | Male | High Grade | Stage II | T2a | M0 | N0 |
| TCGA-CF-A5U8 | 399 | Survival | 59 | Male | Low Grade | Stage II | T2 | M0 | N0 |
| TCGA-UY-A9PF | 117 | Survival | 77 | Male | High Grade | Stage IV | T3a | MX | N2 |
| TCGA-BT-A3PK | 303 | Death | 80 | Male | High Grade | Stage II | T2b | MX | N0 |
| TCGA-KQ-A41R | 1350 | Survival | 80 | Female | High Grade | Stage II | T2 | MX | N0 |
| TCGA-XF-A9SG | 1479 | Survival | 81 | Male | High Grade | Stage II | T2a | MX | N0 |
| TCGA-FD-A43N | 699 | Survival | 76 | Male | High Grade | Stage III | T3a | MX | N0 |
| TCGA-GV-A40G | 580 | Survival | 77 | Male | High Grade | Stage II | T2a | MX | N0 |
| TCGA-ZF-A9RM | 1455 | Survival | 70 | Male | High Grade | Stage II | T0 | MX | N0 |
| TCGA-BT-A42F | 864 | Survival | 64 | Male | High Grade | Stage IV | T4a | MX | N1 |
| TCGA-4Z-AA86 | 311 | Death | 66 | Male | High Grade | Stage IV | T3a | M0 | N1 |
| TCGA-ZF-A9R0 | 680 | Death | 82 | Male | High Grade | Stage III | T3b | M0 | NX |
| TCGA-C4-A0F6 | 700 | Survival | 82 | Female | High Grade | Stage III | T3b | M0 | N0 |
| TCGA-XF-A9SX | 719 | Death | 63 | Female | High Grade | Stage IV | T3b | MX | N2 |
| TCGA-XF-AAMW | 253 | Death | 79 | Female | High Grade | Stage IV | T2b | unknow | N0 |
| TCGA-GU-A764 | 610 | Survival | 66 | Male | High Grade | Stage II | T2b | MX | N0 |
| TCGA-DK-AA6R | 5041 | Survival | 68 | Male | High Grade | Stage IV | T4 | M0 | N1 |
| TCGA-CF-A47S | 333 | Survival | 41 | Male | Low Grade | Stage II | T2 | M0 | N0 |
| TCGA-4Z-AA80 | 19 | Death | 73 | Male | High Grade | Stage II | T2a | M0 | N0 |
| TCGA-ZF-AA5H | 897 | Survival | 60 | Female | High Grade | Stage IV | T3b | M0 | N2 |
| TCGA-GU-A766 | 480 | Survival | 62 | Male | High Grade | Stage II | T2a | MX | N0 |
| TCGA-XF-A9T5 | 2027 | Survival | 78 | Female | High Grade | Stage IV | T3a | MX | N1 |
| TCGA-DK-A1AE | 491 | Survival | 84 | Male | High Grade | Stage III | T3 | M0 | N0 |
| TCGA-ZF-A9RD | 408 | Death | 75 | Female | High Grade | Stage IV | T3a | MX | N2 |
| TCGA-ZF-AA52 | 1077 | Death | 70 | Male | High Grade | Stage III | T3a | MX | NX |
| TCGA-HQ-A5NE | 370 | Death | 57 | Male | High Grade | Stage III | T3 | M0 | N0 |
| TCGA-CF-A9FF | 361 | Survival | 52 | Male | High Grade | Stage II | T2 | M0 | N0 |
| TCGA-UY-A78P | 2380 | Survival | 78 | Female | High Grade | Stage II | T2 | MX | N0 |
| TCGA-DK-A6B2 | 477 | Survival | 70 | Male | High Grade | Stage IV | T3 | M0 | N1 |
| TCGA-G2-AA3B | 2008 | Survival | 75 | Female | High Grade | Stage II | T2 | M0 | N0 |
| TCGA-XF-A9T4 | 495 | Death | 48 | Male | High Grade | Stage IV | T2b | MX | N1 |
| TCGA-BT-A20W | 254 | Death | 71 | Male | High Grade | Stage II | T2b | M0 | N0 |
| TCGA-G2-A2EC | 696 | Death | 58 | Female | High Grade | Stage II | unknow | M0 | N0 |
| TCGA-GU-A763 | 997 | Survival | 72 | Male | High Grade | Stage III | T4 | M0 | N0 |
| TCGA-DK-A1AC | 3981 | Survival | 72 | Male | High Grade | Stage III | T3b | M0 | N0 |
| TCGA-CF-A3MG | 369 | Survival | 48 | Male | Low Grade | Stage II | T2 | M0 | N0 |
| TCGA-ZF-A9RL | 2703 | Survival | 61 | Male | High Grade | Stage II | unknow | MX | unknow |
| TCGA-BT-A42B | 534 | Death | 59 | Male | High Grade | Stage IV | T3a | MX | N1 |
| TCGA-G2-A2EL | 819 | Death | 77 | Male | High Grade | Stage II | unknow | M0 | N0 |
| TCGA-FJ-A871 | 272 | Death | 49 | Male | High Grade | Stage III | T3b | MX | NX |
| TCGA-XF-AAMG | 3364 | Survival | 49 | Male | High Grade | Stage III | T4a | MX | N0 |
| TCGA-GC-A3I6 | 630 | Death | 45 | Male | High Grade | Stage III | T3a | M0 | N0 |
| TCGA-DK-A2I4 | 3835 | Survival | 79 | Male | High Grade | Stage III | T3b | M0 | N0 |
| TCGA-UY-A8OC | 0 | Death | 90 | Male | High Grade | Stage IV | T4 | MX | N1 |
| TCGA-BT-A20O | 370 | Death | 75 | Male | High Grade | Stage III | T3a | MX | N0 |
| TCGA-DK-A6AW | 1621 | Survival | 70 | Male | High Grade | Stage II | T2a | M0 | N0 |
| TCGA-BT-A20R | 154 | Death | 79 | Female | High Grade | Stage IV | T3b | M0 | N1 |
| TCGA-BT-A0S7 | 200 | Death | 75 | Male | High Grade | Stage III | T4a | MX | N0 |
| TCGA-CF-A3MI | 370 | Survival | 62 | Male | Low Grade | Stage II | T2 | M0 | N0 |
| TCGA-FJ-A3ZE | 324 | Death | 65 | Male | High Grade | Stage IV | unknow | M1 | N3 |
| TCGA-GD-A6C6 | 67 | Survival | 64 | Male | High Grade | Stage III | T3a | MX | N0 |
| TCGA-FD-A6TG | 93 | Death | 73 | Male | High Grade | Stage IV | T3a | MX | N2 |
| TCGA-DK-A3IM | 248 | Death | 76 | Male | High Grade | Stage III | T3 | M0 | N0 |
| TCGA-KQ-A41S | 35 | Survival | 87 | Female | High Grade | Stage III | T4a | MX | NX |
| TCGA-XF-AAN4 | 823 | Death | 77 | Female | High Grade | Stage III | T3b | MX | N0 |
| TCGA-ZF-AA53 | 1761 | Survival | 60 | Male | High Grade | Stage II | T2 | M0 | NX |
| TCGA-GU-A767 | 144 | Death | 81 | Male | High Grade | Stage IV | T3b | MX | N2 |
| TCGA-E7-A7DU | 28 | Survival | 73 | Male | Low Grade | Stage III | T3 | M0 | N0 |
| TCGA-ZF-AA51 | 1714 | Survival | 69 | Female | High Grade | Stage II | T2 | M0 | NX |
| TCGA-FD-A6TF | 69 | Death | 80 | Female | High Grade | Stage IV | T3b | M1 | N2 |
| TCGA-FD-A43P | 832 | Survival | 74 | Male | High Grade | Stage II | T2a | MX | N0 |
| TCGA-GC-A3WC | 540 | Survival | 80 | Female | High Grade | Stage III | T3 | MX | N0 |
| TCGA-FD-A3NA | 1845 | Survival | 60 | Male | High Grade | Stage II | T2b | MX | N0 |
| TCGA-DK-A6B1 | 2049 | Survival | 67 | Male | High Grade | Stage II | T2a | M0 | N0 |
| TCGA-ZF-A9RN | 615 | Death | 67 | Female | High Grade | Stage III | T3b | M0 | NX |
| TCGA-ZF-AA4T | 599 | Death | 65 | Male | High Grade | Stage IV | T4 | MX | N2 |
| TCGA-DK-AA76 | 366 | Survival | 64 | Female | High Grade | Stage II | unknow | M0 | N0 |
| TCGA-GC-A4ZW | 15 | Survival | 71 | Male | High Grade | Stage III | T3a | M0 | N0 |
| TCGA-ZF-A9R5 | 1090 | Survival | 59 | Male | High Grade | Stage III | T3 | M0 | N0 |
| TCGA-GU-A42R | 577 | Death | 68 | Male | High Grade | Stage IV | T4a | MX | N2 |
| TCGA-BL-A13J | 81 | Death | 65 | Male | High Grade | Stage IV | T4 | M0 | N2 |
| TCGA-DK-AA6L | 1163 | Death | 48 | Male | High Grade | Stage IV | T3 | MX | N1 |
| TCGA-DK-A1AD | 3420 | Survival | 69 | Male | High Grade | Stage IV | T3b | M0 | N2 |
| TCGA-4Z-AA82 | 1556 | Death | 59 | Male | High Grade | Stage IV | T2a | M0 | N1 |
| TCGA-G2-A3IE | 612 | Death | 51 | Male | High Grade | Stage II | unknow | MX | NX |
| TCGA-DK-AA6P | 457 | Survival | 72 | Female | High Grade | Stage II | unknow | M0 | N0 |
| TCGA-G2-A2EF | 1884 | Survival | 50 | Male | High Grade | Stage II | unknow | M0 | N0 |
| TCGA-XF-A8HE | 3817 | Survival | 47 | Male | High Grade | Stage III | T3b | MX | N0 |
| TCGA-XF-A9SL | 2020 | Death | 69 | Male | High Grade | Stage IV | T3a | MX | N2 |
| TCGA-XF-A9SZ | 859 | Death | 79 | Male | High Grade | Stage IV | T3b | MX | N2 |
| TCGA-YF-AA3L | 364 | Survival | 47 | Female | High Grade | Stage II | T2b | MX | N0 |
| TCGA-S5-AA26 | 503 | Survival | 46 | Male | High Grade | Stage III | T3a | MX | N0 |
| TCGA-C4-A0F0 | 59 | Survival | 60 | Male | High Grade | Stage II | T2b | M0 | N0 |
| TCGA-DK-A3IT | 648 | Survival | 62 | Male | High Grade | Stage III | T3 | M0 | N0 |
| TCGA-ZF-AA54 | 590 | Death | 71 | Male | High Grade | Stage III | T3 | MX | NX |
| TCGA-E7-A97Q | 246 | Death | 60 | Female | High Grade | Stage IV | T4a | MX | N3 |
| TCGA-HQ-A5ND | 274 | Death | 78 | Male | High Grade | Stage IV | T3b | M0 | N1 |
| TCGA-CF-A9FL | 565 | Death | 85 | Male | High Grade | Stage III | T3b | M0 | N0 |
| TCGA-G2-AA3C | 211 | Death | 66 | Male | High Grade | Stage IV | T3b | M0 | N1 |
| TCGA-FD-A6TC | 187 | Survival | 79 | Female | High Grade | Stage III | T4a | MX | N0 |
| TCGA-FD-A6TK | 330 | Survival | 60 | Male | High Grade | Stage III | T3a | MX | N0 |
| TCGA-XF-A8HD | 2964 | Survival | 77 | Male | High Grade | Stage III | T3a | MX | N0 |
| TCGA-DK-A3X2 | 547 | Death | 85 | Male | High Grade | Stage IV | T3 | M0 | N2 |
| TCGA-GC-A6I3 | 0 | Survival | 90 | Female | High Grade | Stage IV | T3a | MX | N1 |
| TCGA-FD-A3N5 | 685 | Death | 69 | Male | High Grade | Stage II | T2b | MX | N0 |
| TCGA-XF-A9SI | 2423 | Survival | 73 | Female | High Grade | Stage II | T2b | MX | N0 |
| TCGA-FT-A3EE | 99 | Death | 80 | Female | High Grade | Stage III | T4a | MX | N0 |
| TCGA-CU-A3YL | 906 | Survival | 67 | Male | High Grade | Stage III | T4a | M0 | N0 |
| TCGA-UY-A8OB | 2109 | Survival | 63 | Male | High Grade | Stage IV | T3a | MX | N1 |
| TCGA-5N-A9KM | 530 | Death | 73 | Female | High Grade | Stage III | T4a | MX | N0 |
| TCGA-2F-A9KT | 2352 | Survival | 83 | Male | High Grade | Stage II | T2b | M0 | N0 |
| TCGA-FD-A5BV | 163 | Death | 47 | Female | High Grade | Stage III | T3b | MX | N0 |
| TCGA-GC-A3RB | 582 | Survival | 54 | Male | High Grade | Stage III | T3b | M0 | N0 |
| TCGA-DK-A2I1 | 546 | Survival | 73 | Female | High Grade | Stage II | T2b | M0 | N0 |
| TCGA-FD-A3B7 | 122 | Death | 66 | Male | High Grade | Stage III | T3a | MX | N0 |
| TCGA-FD-A62N | 82 | Survival | 69 | Male | High Grade | Stage III | T3b | MX | N0 |
| TCGA-E7-A3Y1 | 163 | Survival | 57 | Male | Low Grade | Stage II | unknow | M0 | N0 |
| TCGA-DK-A3X1 | 2009 | Survival | 78 | Female | High Grade | Stage III | T3 | M0 | N0 |
| TCGA-KQ-A41Q | 361 | Survival | 89 | Male | High Grade | Stage III | T3b | MX | N0 |
| TCGA-E7-A4XJ | 68 | Death | 66 | Male | High Grade | Stage II | T2 | M0 | N0 |
| TCGA-K4-A83P | 495 | Survival | 77 | Male | unknow | Stage IV | T4a | MX | N1 |
| TCGA-K4-A5RI | 356 | Death | 67 | Female | High Grade | Stage III | T3a | MX | N0 |
| TCGA-DK-A1AG | 475 | Survival | 65 | Male | High Grade | Stage III | T3 | M0 | N0 |
| TCGA-ZF-AA4X | 2044 | Survival | 56 | Male | High Grade | Stage II | T2 | M0 | NX |
| TCGA-2F-A9KO | 734 | Death | 63 | Male | High Grade | Stage IV | T3 | M0 | N1 |
| TCGA-GU-A42Q | 344 | Death | 67 | Male | High Grade | Stage III | T3b | M0 | N0 |
| TCGA-YC-A89H | 573 | Survival | 78 | Female | High Grade | Stage II | unknow | MX | unknow |
| TCGA-K4-A3WS | 761 | Survival | 66 | Male | High Grade | Stage III | T3a | MX | N0 |
| TCGA-GC-A3OO | 481 | Survival | 79 | Male | High Grade | Stage II | T2b | M0 | N0 |
| TCGA-XF-A9SY | 640 | Survival | 60 | Female | High Grade | Stage IV | T3b | MX | N2 |
| TCGA-CF-A3MF | 383 | Survival | 34 | Male | Low Grade | Stage III | T3 | M0 | N0 |
| TCGA-FD-A6TH | 131 | Death | 63 | Male | High Grade | Stage IV | T3 | MX | N2 |
| TCGA-DK-A6B0 | 2330 | Survival | 61 | Male | High Grade | Stage II | T2b | M0 | N0 |
| TCGA-LT-A8JT | 641 | Survival | 69 | Female | High Grade | Stage II | T2a | M0 | N0 |
| TCGA-XF-AAN8 | 118 | Death | 74 | Female | High Grade | Stage III | T3b | MX | N0 |
| TCGA-XF-A9T3 | 68 | Survival | 69 | Female | High Grade | Stage IV | T3b | MX | N2 |
| TCGA-DK-A3IU | 706 | Death | 58 | Male | High Grade | Stage II | T2b | M0 | N0 |
| TCGA-ZF-AA4V | 1806 | Survival | 66 | Male | High Grade | Stage III | T3b | M0 | N0 |
| TCGA-XF-AAMZ | 1348 | Death | 81 | Female | High Grade | Stage IV | T3a | MX | N2 |
| TCGA-E7-A8O8 | 13 | Survival | 59 | Male | High Grade | Stage II | T2 | M0 | N0 |
| TCGA-FD-A62O | 216 | Death | 74 | Male | High Grade | Stage IV | T3a | MX | N2 |
| TCGA-XF-A9SV | 388 | Death | 82 | Male | High Grade | Stage IV | T4a | M1 | N2 |
| TCGA-ZF-AA5P | 372 | Survival | 65 | Male | High Grade | Stage IV | T2b | M0 | N2 |
| TCGA-PQ-A6FN | 507 | Survival | 78 | Female | High Grade | Stage III | T3a | MX | N0 |
| TCGA-E7-A7PW | 416 | Survival | 63 | Male | High Grade | Stage III | T3a | M0 | NX |
| TCGA-E7-A678 | 798 | Survival | 55 | Male | Low Grade | Stage III | T3 | M0 | N0 |
| TCGA-2F-A9KW | 254 | Death | 67 | Female | High Grade | Stage III | T3b | MX | N0 |
| TCGA-UY-A78O | 2312 | Survival | 75 | Female | High Grade | Stage II | T2 | MX | N0 |
| TCGA-DK-A3IN | 250 | Death | 72 | Male | High Grade | Stage III | T4a | M0 | N0 |
| TCGA-FD-A5C1 | 1792 | Survival | 61 | Female | High Grade | Stage III | T3b | MX | N0 |
| TCGA-XF-AAN0 | 1718 | Death | 68 | Male | High Grade | Stage IV | T4a | M0 | N2 |
| TCGA-CF-A8HX | 345 | Survival | 55 | Female | High Grade | Stage II | T2 | M0 | N0 |
| TCGA-GD-A3OQ | 95 | Survival | 48 | Male | High Grade | Stage IV | T4a | MX | N1 |
| TCGA-FJ-A3Z9 | 385 | Death | 72 | Male | High Grade | unknow | TX | M0 | N0 |
| TCGA-GC-A3RD | 428 | Survival | 83 | Female | High Grade | Stage III | T3a | M0 | N0 |
| TCGA-ZF-AA4U | 262 | Death | 70 | Male | High Grade | Stage III | T4a | MX | NX |
| TCGA-FJ-A3ZF | 524 | Survival | 73 | Male | High Grade | Stage III | unknow | M0 | N0 |
| TCGA-E7-A7DV | 37 | Survival | 44 | Male | High Grade | Stage IV | T4 | MX | N3 |
| TCGA-XF-AAME | 2828 | Death | 64 | Female | High Grade | Stage II | T2b | MX | N0 |
| TCGA-CU-A0YO | 149 | Death | 84 | Male | High Grade | Stage IV | T3a | MX | N2 |
| TCGA-G2-A2EK | 485 | Survival | 57 | Male | High Grade | Stage II | unknow | M0 | N0 |
| TCGA-DK-A3IK | 146 | Death | 87 | Male | High Grade | Stage IV | T3 | M0 | N2 |
| TCGA-UY-A9PH | 1561 | Survival | 73 | Male | High Grade | Stage II | T2b | MX | N0 |
| TCGA-E7-A8O7 | 466 | Survival | 52 | Male | Low Grade | Stage II | T2 | M0 | N0 |
| TCGA-CF-A3MH | 398 | Survival | 75 | Male | Low Grade | Stage II | T2 | M0 | N0 |
| TCGA-FD-A5BZ | 835 | Death | 77 | Female | High Grade | Stage IV | T3a | MX | N1 |
| TCGA-FD-A3B3 | 974 | Death | 74 | Female | High Grade | Stage III | T3 | MX | N0 |
| TCGA-GV-A3JZ | 603 | Survival | 55 | Male | High Grade | Stage IV | T4a | MX | N3 |
| TCGA-HQ-A2OE | 1174 | Survival | 69 | Male | unknow | unknow | T2a | MX | N2 |
| TCGA-BT-A0YX | 400 | Death | 70 | Female | High Grade | Stage III | T3b | M0 | N0 |
| TCGA-XF-AAMJ | 1670 | Death | 70 | Male | High Grade | Stage III | T3b | M0 | N0 |
| TCGA-CU-A3QU | 158 | Survival | 58 | Male | High Grade | Stage IV | T2b | M0 | N1 |
| TCGA-XF-A9SM | 1048 | Survival | 76 | Male | High Grade | Stage III | T3b | MX | N0 |
| TCGA-K4-A6FZ | 55 | Survival | 75 | Female | High Grade | Stage III | T3a | MX | N0 |
| TCGA-BT-A20T | 453 | Death | 63 | Male | High Grade | Stage IV | T3b | M0 | N1 |
| TCGA-ZF-A9R1 | 773 | Survival | 81 | Male | High Grade | Stage IV | T3b | M0 | N1 |
| TCGA-DK-A3WY | 4967 | Survival | 67 | Female | High Grade | Stage III | T3 | M0 | N0 |
| TCGA-2F-A9KP | 364 | Death | 66 | Male | High Grade | Stage IV | T3a | MX | N2 |
| TCGA-K4-A6MB | 469 | Survival | 64 | Male | High Grade | Stage IV | T3b | MX | N1 |
| TCGA-DK-A6B5 | 1542 | Survival | 45 | Male | High Grade | Stage IV | T4a | M0 | N2 |
| TCGA-XF-AAN3 | 2625 | Survival | 76 | Male | High Grade | Stage IV | T3b | MX | N2 |
| TCGA-FD-A3B8 | 384 | Survival | 56 | Male | High Grade | Stage II | T2b | MX | N0 |
| TCGA-BT-A20J | 579 | Death | 75 | Male | High Grade | Stage II | T2b | MX | N0 |
| TCGA-FD-A6TD | 386 | Death | 77 | Male | High Grade | Stage III | T3a | MX | N0 |
| TCGA-H4-A2HO | 46 | Survival | 53 | Male | High Grade | Stage III | T4a | MX | N0 |
| TCGA-GV-A3JV | 434 | Death | 66 | Male | High Grade | Stage IV | T3b | MX | N1 |
| TCGA-UY-A9PE | 189 | Survival | 86 | Male | High Grade | Stage IV | T2b | MX | N2 |
| TCGA-FD-A3N6 | 851 | Survival | 43 | Female | High Grade | Stage II | T2b | MX | N0 |
| TCGA-GD-A76B | 224 | Survival | 86 | Female | High Grade | Stage II | T2b | MX | N0 |
| TCGA-DK-AA75 | 340 | Death | 82 | Male | High Grade | Stage III | T3 | M0 | N0 |
|  |  |  |  |  |  |  |  |  |  |
