# Supplementary material for: Development and validation of a model based on immunogenic cell death related genes to predict the prognosis and immune response to bladder urothelial carcinoma
Source: Front Oncol. 2023 Nov 10;13:1291720. doi: 10.3389/fonc.2023.1291720 (PMC10676223; doi:10.3389/fonc.2023.1291720)
Supplement: Supplementary file 2 [file Table_2.docx]

**Supplementary Table 2 Clinical features of all BLCA patients in HBlaU079Su01 cohort**

| **Id** | **Overall Survival** | **Survival month** | **Gender** | **Age** | **Stage** |
| --- | --- | --- | --- | --- | --- |
| F03A0036 | Survival | unknow | Male | 64 | II |
| F03A0039 | Death | 26 | Male | 76 | IV |
| F03A0051 | Survival | unknow | Male | 67 | Tis |
| F03A0074 | Death | 20 | Male | 82 | III |
| F03A0083 | Survival | 65 | Male | 82 | II |
| F03A0084 | Survival | 64 | Male | 62 | II |
| F03A0091 | Survival | 63 | Male | 75 | III |
| F03A0095 | Survival | 62 | Male | 80 | II |
| F03A0105 | Death | 7 | Male | 50 | III |
| F03A0110 | Death | 5 | Male | 59 | III |
| F03A0200 | Survival | 52 | Male | 66 | II |
| F03A0112 | Death | 4 | Male | 79 | III |
| F03A0121 | Survival | 51 | Male | 76 | III |
| F03A0220 | Survival | 39 | Male | 67 | II |
| F03A0224 | Survival | unknow | Male | 83 | unknow |
| F03A0373 | Death | 2 | Male | 81 | II |
| F03A0026 | Survival | unknow | Male | 75 | IV |
| F03A0027 | Death | 1 | Male | 71 | IV |
| F03A0037 | Survival | 82 | Male | 75 | III |
| F03A0038 | Death | 27 | Female | 72 | II |
| F03A0040 | Death | 11 | Female | 66 | II |
| F03A0044 | Death | 4 | Male | 67 | IV |
| F03A0043 | Death | 23 | Male | 58 | unknow |
| F03A0045 | Death | 8 | Male | 77 | unknow |
| F03A0058 | Death | 3 | Male | 68 | III |
| F03A0060 | Death | 57 | Male | 61 | I |
| F03A0061 | Death | 16 | Female | 58 | III |
| F03A0068 | Death | 18 | Male | 73 | unknow |
| F03A0070 | Survival | unknow | Female | 42 | III |
| F03A0071 | Survival | 70 | Male | 57 | II |
| F03A0076 | Death | 9 | Male | 55 | III |
| F03A0079 | Survival | unknow | Male | 75 | I |
| F03A0088 | Survival | 63 | Male | 73 | III |
| F03A0090 | Death | 8 | Male | 77 | IV |
| F03A0097 | Survival | unknow | Male | 57 | Tis |
| F03A0102 | Death | 14 | Male | 78 | unknow |
| F03A0103 | Death | 59 | Male | 74 | I |
| F03A0196 | Survival | 57 | Female | 72 | unknow |
| F03A0108 | Survival | 55 | Female | 65 | III |

| F03A0197 | Death | 25 | Male | 59 | III-VI |
| --- | --- | --- | --- | --- | --- |
| F03A0120 | Death | 43 | Male | 75 | III |
| F03A0122 | Survival | unknow | Male | 55 | III |
| F03A0123 | Survival | 51 | Male | 57 | III |
| F03A0124 | Death | 5 | Male | 61 | IV |
| F03A0126 | Death | 26 | Male | 79 | III |
| F03A0127 | Survival | 50 | Male | 77 | I |
| F03A0137 | Survival | unknow | Male | 48 | Tis |
| F03A0201 | Death | 14 | Female | 56 | unknow |
| F03A0131 | Death | 14 | Female | 85 | III-VI |
| F03A0133 | Survival | 49 | Male | 76 | I |
| F03A0134 | Death | 39 | Male | 61 | I |
| F03A0141 | Survival | unknow | Male | 75 | Tis |
| F03A0202 | Survival | 44 | Female | 66 | unknow |
| F03A0216 | Death | 23 | Male | 84 | III |
| F03A0218 | Survival | 40 | Male | 75 | IV |
| F03A0219 | Survival | 39 | Male | 78 | Tis |
| F03A0221 | Survival | unknow | Male | 44 | III |
| F03A0222 | Death | 9 | Female | 77 | III |
| F03A0231 | Death | 9 | Male | 71 | III |
| F03A0347 | Survival | 27 | Male | 62 | II |
| F03A0374 | Survival | 25 | Male | 59 | III-VI |
| F03A0372 | Survival | 24 | Male | 64 | IV |
| F03A0375 | Survival | 24 | Male | 67 | unknow |
