# Supplementary material for: Development and validation of a model based on immunogenic cell death related genes to predict the prognosis and immune response to bladder urothelial carcinoma
Source: Front Oncol. 2023 Nov 10;13:1291720. doi: 10.3389/fonc.2023.1291720 (PMC10676223; doi:10.3389/fonc.2023.1291720)
Supplement: Supplementary file 3 [file Table_3.docx]

**Supplementary Table 3 Clinical features of all BLCA patients in GEO cohort**

| Id | Gender | Age | Stage | Grade | Overall survival | Survival month |
| --- | --- | --- | --- | --- | --- | --- |
| GSM340605 | Male | 78 | T1N0M0 | Low | Death | 18.17 |
| GSM340606 | Female | 54 | TaN0M0 | Low | Survival | 136.97 |
| GSM340607 | Male | 37 | TaN0M0 | Low | Survival | 136.37 |
| GSM340608 | Male | 72 | T1N0M0 | Low | Death | 26.9 |
| GSM340609 | Male | 68 | T1N0M0 | Low | Death | 87.07 |
| GSM340610 | Male | 80 | T1N0M0 | Low | Death | 15.3 |
| GSM340611 | Male | 67 | T1N0M0 | Low | Death | 18.53 |
| GSM340612 | Male | 58 | T2N0M0 | Low | Survival | 129.93 |
| GSM340613 | Female | 77 | T3bN0M0 | High | Death | 12.57 |
| GSM340614 | Male | 64 | T3aN0M0 | Low | Survival | 128.97 |
| GSM340615 | Male | 63 | TaN0M0 | Low | Survival | 125.03 |
| GSM340616 | Female | 56 | TaN0M0 | Low | Survival | 125.27 |
| GSM340617 | Male | 69 | T1N0M0 | Low | Survival | 130.6 |
| GSM340618 | Male | 75 | T4N0M0 | Low | Death | 1.03 |
| GSM340619 | Male | 76 | T1N0M0 | Low | Survival | 129.67 |
| GSM340620 | Male | 61 | T3aN2M1 | Low | Death | 16.67 |
| GSM340621 | Male | 45 | T1N0M0 | Low | Survival | 127.63 |
| GSM340622 | Male | 58 | TaN0M0 | Low | Survival | 124.53 |
| GSM340623 | Female | 71 | T4N2M1 | High | Death | 3.13 |
| GSM340624 | Male | 79 | T1N0M0 | Low | Death | 65.93 |
| GSM340625 | Male | 75 | T1N0M0 | Low | Death | 3.23 |
| GSM340626 | Male | 63 | T1N0M0 | Low | Survival | 120.6 |
| GSM340627 | Female | 68 | T1N0M0 | Low | Death | 22.67 |
| GSM340628 | Male | 56 | T3aN0M0 | High | Survival | 120.5 |
| GSM340629 | Male | 82 | T1N0M0 | Low | Death | 50.43 |
| GSM340630 | Male | 38 | T2N0M0 | Low | Death | 66.3 |
| GSM340631 | Male | 55 | TaN0M0 | Low | Survival | 120.17 |
| GSM340632 | Male | 53 | T1N0M0 | Low | Survival | 120.6 |
| GSM340633 | Male | 64 | T3bN0M0 | High | Death | 14.57 |
| GSM340634 | Male | 68 | T3aN0M0 | High | Survival | 115.83 |
| GSM340635 | Male | 65 | TaN0M0 | Low | Survival | 106.6 |
| GSM340636 | Female | 65 | T2N0M0 | High | Survival | 121.2 |
| GSM340637 | Male | 74 | T1N0M0 | Low | Death | 98 |
| GSM340638 | Male | 67 | T1N0M0 | Low | Death | 67.03 |
| GSM340639 | Male | 61 | TaN0M0 | Low | Survival | 99.73 |
| GSM340640 | Male | 78 | TaN0M0 | Low | Death | 50.3 |
| GSM340641 | Male | 52 | T1N0M0 | High | Survival | 98.23 |
| GSM340642 | Male | 65 | T1N0M0 | Low | Survival | 106.03 |
| GSM340643 | Male | 69 | T1N0M0 | Low | Survival | 95.3 |
| GSM340644 | Male | 64 | TaN0M0 | Low | Death | 80.43 |
| GSM340645 | Male | 69 | T1N0M0 | High | Death | 35.7 |
| GSM340646 | Female | 75 | T1N0M0 | High | Death | 51.77 |
| GSM340647 | Male | 53 | T1N0M0 | Low | Survival | 92.83 |
| GSM340648 | Male | 58 | T2N0M0 | Low | Survival | 90.73 |
| GSM340649 | Male | 35 | T1N0M0 | Low | Survival | 90.47 |
| GSM340650 | Male | 43 | T1N0M0 | Low | Survival | 86.07 |
| GSM340651 | Male | 66 | T1N0M0 | Low | Death | 134.97 |
| GSM340652 | Male | 83 | T1N0M0 | Low | Death | 65.93 |
| GSM340653 | Male | 73 | T3bN0M0 | High | Death | 25.83 |
| GSM340654 | Male | 51 | T3bN2M0 | High | Death | 10.27 |
| GSM340655 | Female | 88 | T1N0M0 | High | Death | 85.43 |
| GSM340656 | Male | 77 | T1N0M0 | High | Death | 3.17 |
| GSM340657 | Male | 79 | T1N0M0 | Low | Survival | 83.63 |
| GSM340658 | Male | 79 | T1N0M0 | Low | Survival | 83.63 |
| GSM340659 | Male | 63 | T1N0M0 | Low | Death | 70.93 |
| GSM340660 | Male | 62 | T2N0M0 | Low | Survival | 81.63 |
| GSM340661 | Male | 67 | T1N0M0 | Low | Survival | 80.2 |
| GSM340662 | Male | 73 | T1N0M0 | High | Survival | 78.83 |
| GSM340663 | Male | 79 | T1N0M0 | High | Survival | 78.73 |
| GSM340664 | Male | 59 | TaN0M0 | Low | Death | 70.73 |
| GSM340665 | Male | 78 | T2N1M1 | High | Death | 8.7 |
| GSM340666 | Male | 59 | T1N0M0 | Low | Survival | 75.33 |
| GSM340667 | Female | 38 | T1N0M0 | Low | Survival | 75.7 |
| GSM340668 | Male | 49 | TaN0M0 | Low | Survival | 75.13 |
| GSM340669 | Male | 62 | T1N0M0 | Low | Survival | 75.13 |
| GSM340670 | Male | 61 | TaN0M0 | Low | Survival | 74.17 |
| GSM340671 | Male | 74 | T1N0M0 | High | Death | 16.23 |
| GSM340672 | Male | 72 | T3bN0M0 | High | Death | 15.1 |
| GSM340673 | Female | 60 | T2N0M0 | Low | Death | 11.23 |
| GSM340674 | Male | 60 | T2N0M0 | High | Survival | 69.43 |
| GSM340675 | Male | 82 | T1N0M0 | High | Death | 25.03 |
| GSM340676 | Male | 67 | TaN0M0 | Low | Survival | 67.07 |
| GSM340677 | Male | 64 | T2N0M0 | High | Survival | 65.23 |
| GSM340678 | Male | 73 | T4N0M0 | High | Death | 11.97 |
| GSM340679 | Male | 67 | T1N0M0 | Low | Survival | 62 |
| GSM340680 | Male | 77 | T1N0M0 | Low | Survival | 61.33 |
| GSM340681 | Male | 66 | T1N0M0 | High | Survival | 60.9 |
| GSM340682 | Male | 79 | T1N0M0 | Low | Survival | 60.2 |
| GSM340683 | Male | 48 | T1N0M0 | Low | Survival | 59.6 |
| GSM340684 | Male | 76 | T4N0M0 | High | Death | 10.4 |
| GSM340685 | Male | 66 | T1N0M0 | Low | Death | 59.87 |
| GSM340686 | Male | 67 | T1N0M0 | Low | Survival | 59 |
| GSM340687 | Female | 68 | T1N0M0 | Low | Survival | 58.73 |
| GSM340688 | Male | 67 | T1N0M0 | Low | Death | 46.17 |
| GSM340689 | Female | 80 | T1N0M0 | Low | Survival | 58.43 |
| GSM340690 | Male | 68 | T1N0M0 | Low | Survival | 58.07 |
| GSM340691 | Female | 32 | T1N0M0 | Low | Survival | 57.63 |
| GSM340692 | Male | 63 | T1N0M0 | Low | Death | 36.3 |
| GSM340693 | Female | 80 | T2N0M0 | High | Survival | 56.47 |
| GSM340694 | Male | 67 | T2N0M0 | High | Survival | 55.5 |
| GSM340695 | Male | 37 | T1N0M0 | Low | Survival | 52.97 |
| GSM340696 | Male | 71 | T3N1M0 | High | Death | 11.5 |
| GSM340697 | Male | 60 | T3N0M0 | High | Death | 17.87 |
| GSM340698 | Male | 74 | T1N0M0 | High | Death | 28.53 |
| GSM340699 | Male | 80 | T1N0M0 | Low | Death | 17.63 |
| GSM340700 | Male | 85 | T2N0M0 | High | Death | 15.4 |
| GSM340701 | Female | 59 | T3bN0M0 | High | Survival | 48.43 |
| GSM340702 | Female | 65 | T4N1M1 | Low | Death | 4.5 |
| GSM340703 | Male | 57 | TaN0M0 | Low | Survival | 47.5 |
| GSM340704 | Male | 73 | T2N1M0 | High | Death | 5.23 |
| GSM340705 | Male | 66 | T3bN0M0 | High | Survival | 45.6 |
| GSM340706 | Male | 66 | T2N0M0 | High | Survival | 45.13 |
| GSM340707 | Male | 64 | TaN0M0 | Low | Survival | 43.5 |
| GSM340708 | Male | 68 | T1N0M0 | High | Survival | 44.1 |
| GSM340709 | Female | 59 | T2N0M0 | Low | Death | 5.93 |
| GSM340710 | Male | 66 | T1N0M0 | High | Death | 31.53 |
| GSM340711 | Female | 63 | T1N0M0 | Low | Survival | 41.8 |
| GSM340712 | Male | 79 | T2N2M0 | High | Death | 6.5 |
| GSM340713 | Male | 44 | T1N0M0 | Low | Survival | 41.07 |
| GSM340714 | Male | 66 | T2bN0M0 | High | Survival | 39.77 |
| GSM340715 | Male | 61 | T2N0M0 | Low | Survival | 38.97 |
| GSM340716 | Female | 80 | T1N0M0 | Low | Death | 31.97 |
| GSM340717 | Male | 57 | T1N0M0 | Low | Survival | 37.3 |
| GSM340718 | Male | 55 | T2aN0M0 | Low | Survival | 36.83 |
| GSM340719 | Male | 52 | T1N0M0 | Low | Survival | 36.57 |
| GSM340720 | Male | 50 | T2aN0M0 | High | Survival | 28.1 |
| GSM340721 | Male | 78 | T2N0M0 | High | Death | 3.93 |
| GSM340722 | Female | 71 | T1N0M0 | Low | Death | 23.53 |
| GSM340723 | Female | 81 | T3N0M0 | High | Death | 15.1 |
| GSM340724 | Male | 43 | TaN0M0 | Low | Survival | 34.87 |
| GSM340725 | Male | 80 | T3N1M0 | High | Survival | 34.23 |
| GSM340726 | Male | 47 | TaN0M0 | Low | Survival | 33.7 |
| GSM340727 | Male | 71 | T4aN0M0 | Low | Death | 11.07 |
| GSM340728 | Female | 73 | T3N0M0 | High | Death | 5.77 |
| GSM340729 | Male | 87 | T2N0M0 | Low | Survival | 33.07 |
| GSM340730 | Male | 67 | TaN0M0 | Low | Survival | 32.87 |
| GSM340731 | Male | 82 | T2N0M0 | High | Death | 9.23 |
| GSM340732 | Female | 78 | T3N1M0 | High | Death | 7.1 |
| GSM340733 | Male | 60 | T2N0M0 | Low | Death | 17.13 |
| GSM340734 | Male | 63 | TaN0M0 | Low | Death | 23.57 |
| GSM340735 | Male | 72 | T1N0M0 | Low | Survival | 30.7 |
| GSM340736 | Male | 68 | T1N0M0 | High | Survival | 30.1 |
| GSM340737 | Male | 64 | T1N0M0 | Low | Survival | 29.73 |
| GSM340738 | Male | 83 | T1N0M0 | Low | Death | 15.43 |
| GSM340739 | Male | 66 | T1N0M0 | Low | Survival | 29.37 |
| GSM340740 | Male | 68 | T1N0M0 | Low | Death | 17.67 |
| GSM340741 | Male | 63 | T1N0M0 | Low | Survival | 28.47 |
| GSM340742 | Female | 45 | T1N0M0 | Low | Survival | 23.9 |
| GSM340743 | Male | 84 | T1N0M0 | Low | Survival | 23.57 |
| GSM340744 | Male | 72 | T1N0M0 | High | Death | 13.93 |
| GSM340745 | Female | 72 | TaN3M1 | High | Death | 2.13 |
| GSM340746 | Male | 66 | T2N0M0 | High | Survival | 22.27 |
| GSM340747 | Male | 74 | T4N0M1 | Low | Death | 6.87 |
| GSM340748 | Male | 63 | T1N0M0 | Low | Survival | 20.83 |
| GSM340749 | Male | 58 | T1N0M0 | Low | Survival | 21.63 |
| GSM340750 | Male | 70 | TaN0M0 | Low | Survival | 21.4 |
| GSM340751 | Male | 40 | T1N0M0 | Low | Survival | 57.87 |
| GSM340752 | Female | 80 | T1N0M0 | High | Survival | 21.23 |
| GSM340753 | Female | 62 | TaN0M0 | Low | Survival | 21.23 |
| GSM340754 | Male | 24 | T1N0M0 | Low | Survival | 20.47 |
| GSM340755 | Male | 45 | T1N0M0 | High | Survival | 20.4 |
| GSM340756 | Female | 67 | TaN0M0 | Low | Survival | 19 |
| GSM340757 | Male | 65 | T2N0M0 | High | Survival | 79.3 |
| GSM340758 | Female | 59 | T4N1M0 | High | Death | 15.47 |
| GSM340759 | Male | 64 | T2N1M1 | High | Death | 26.43 |
| GSM340760 | Male | 70 | T4N0M0 | High | Death | 13.27 |
| GSM340761 | Male | 66 | T3NxM0 | High | Death | 6.4 |
| GSM340762 | Male | 77 | T4aN0M0 | High | Death | 10.67 |
| GSM340763 | Male | 67 | T2N2M0 | Low | Survival | 18.13 |
| GSM340764 | Male | 51 | T3aN0M0 | Low | Survival | 15.37 |
| GSM340765 | Male | 42 | T4N2M0 | High | Survival | 11.8 |
| GSM340766 | Male | 72 | T2N0M0 | High | Survival | 10.87 |
| GSM340767 | Female | 70 | T2N0M0 | Low | Survival | 10.03 |
| GSM340768 | Male | 54 | T1N0M0 | Low | Survival | 6.73 |
| GSM340769 | Male | 62 | T2aN0M0 | High | Survival | 5.3 |
|  |  |  |  |  |  |  |
