# Supplementary material for: Development and validation of a model based on immunogenic cell death related genes to predict the prognosis and immune response to bladder urothelial carcinoma
Source: Front Oncol. 2023 Nov 10;13:1291720. doi: 10.3389/fonc.2023.1291720 (PMC10676223; doi:10.3389/fonc.2023.1291720)
Supplement: Supplementary file 4 [file Table_4.docx]

**Supplementary Table 4 The list of ICD-related genes**

| ICD | -related Genes |
| --- | --- |
| *ATG5*  *BAX*  *CALR*  *CASP1*  *CASP8*  *CD4*  *CD8A*  *CD8B*  *CXCR3*  *EIF2AK3*  *ENTPD1*  *FOXP3*  *HMGB1*  *HSP90A1*  *IFNA1*  *IFNB1*  *IFNG*  *IFNGR1*  *IL10*  *IL17A*  *IL17RA*  *IL1B*  *IL1R1*  *IL6*  *LY96*  *MYD88*  *NLRP3*  *NT5E*  *P2RX7*  *PDIA3*  *PIK3CA*  *PRF1*  *TLR4*  *TNF* | |
