# Supplementary material for: Development and validation of a model based on immunogenic cell death related genes to predict the prognosis and immune response to bladder urothelial carcinoma
Source: Front Oncol. 2023 Nov 10;13:1291720. doi: 10.3389/fonc.2023.1291720 (PMC10676223; doi:10.3389/fonc.2023.1291720)
Supplement: Supplementary file 5 [file Table_5.docx]

**Supplementary Table 5 The list of the IRGs according the ICD -high and -low cohorts**

| ID | Cluster | Group |
| --- | --- | --- |
| TCGA-ZF-A9RN | C1 | ICD high |
| TCGA-FD-A6TK | C1 | ICD high |
| TCGA-BT-A20V | C1 | ICD high |
| TCGA-DK-A2I4 | C1 | ICD high |
| TCGA-2F-A9KO | C1 | ICD high |
| TCGA-UY-A9PH | C1 | ICD high |
| TCGA-DK-A3WW | C1 | ICD high |
| TCGA-K4-A5RH | C1 | ICD high |
| TCGA-UY-A8OB | C1 | ICD high |
| TCGA-K4-A5RJ | C1 | ICD high |
| TCGA-G2-A2ES | C1 | ICD high |
| TCGA-XF-A9SM | C1 | ICD high |
| TCGA-XF-AAMQ | C1 | ICD high |
| TCGA-XF-A9SX | C1 | ICD high |
| TCGA-FD-A3B8 | C1 | ICD high |
| TCGA-DK-AA74 | C1 | ICD high |
| TCGA-FD-A3B6 | C1 | ICD high |
| TCGA-4Z-AA87 | C1 | ICD high |
| TCGA-DK-A1A6 | C1 | ICD high |
| TCGA-FD-A5BS | C1 | ICD high |
| TCGA-FD-A3B3 | C1 | ICD high |
| TCGA-4Z-AA86 | C1 | ICD high |
| TCGA-ZF-A9RD | C1 | ICD high |
| TCGA-ZF-AA53 | C1 | ICD high |
| TCGA-ZF-A9R7 | C1 | ICD high |
| TCGA-FD-A62P | C1 | ICD high |
| TCGA-E7-A4IJ | C1 | ICD high |
| TCGA-BT-A42E | C1 | ICD high |
| TCGA-FD-A6TB | C1 | ICD high |
| TCGA-ZF-AA5H | C1 | ICD high |
| TCGA-XF-A8HD | C1 | ICD high |
| TCGA-DK-A6B6 | C1 | ICD high |
| TCGA-GU-A766 | C1 | ICD high |
| TCGA-DK-AA6S | C1 | ICD high |
| TCGA-4Z-AA7Q | C1 | ICD high |
| TCGA-E7-A7XN | C1 | ICD high |
| TCGA-FD-A6TD | C1 | ICD high |
| TCGA-BT-A20Q | C1 | ICD high |
| TCGA-GV-A40E | C1 | ICD high |
| TCGA-E7-A541 | C1 | ICD high |
| TCGA-XF-AAN2 | C1 | ICD high |
| TCGA-LC-A66R | C1 | ICD high |
| TCGA-ZF-A9R4 | C1 | ICD high |
| TCGA-E7-A97P | C1 | ICD high |
| TCGA-XF-A9T8 | C1 | ICD high |
| TCGA-XF-AAN5 | C1 | ICD high |
| TCGA-UY-A78P | C1 | ICD high |
| TCGA-K4-A54R | C1 | ICD high |
| TCGA-XF-A9T5 | C1 | ICD high |
| TCGA-BT-A20J | C1 | ICD high |
| TCGA-ZF-AA4V | C1 | ICD high |
| TCGA-DK-AA6Q | C1 | ICD high |
| TCGA-ZF-AA58 | C1 | ICD high |
| TCGA-GV-A3JX | C1 | ICD high |
| TCGA-G2-A2EO | C1 | ICD high |
| TCGA-DK-A3WX | C1 | ICD high |
| TCGA-FD-A62N | C1 | ICD high |
| TCGA-XF-A8HE | C1 | ICD high |
| TCGA-XF-A9SI | C1 | ICD high |
| TCGA-FD-A3B4 | C1 | ICD high |
| TCGA-FD-A43P | C1 | ICD high |
| TCGA-SY-A9G5 | C1 | ICD high |
| TCGA-GC-A6I1 | C1 | ICD high |
| TCGA-G2-A2EF | C1 | ICD high |
| TCGA-FT-A61P | C1 | ICD high |
| TCGA-GC-A3WC | C1 | ICD high |
| TCGA-FD-A3SP | C1 | ICD high |
| TCGA-FD-A3B7 | C1 | ICD high |
| TCGA-BT-A42F | C1 | ICD high |
| TCGA-ZF-A9RF | C1 | ICD high |
| TCGA-FD-A5BT | C1 | ICD high |
| TCGA-E7-A85H | C1 | ICD high |
| TCGA-GU-A762 | C1 | ICD high |
| TCGA-XF-A9SJ | C1 | ICD high |
| TCGA-FD-A5C1 | C1 | ICD high |
| TCGA-DK-A3IU | C1 | ICD high |
| TCGA-UY-A78K | C1 | ICD high |
| TCGA-BT-A20O | C1 | ICD high |
| TCGA-4Z-AA7W | C1 | ICD high |
| TCGA-E7-A3X6 | C1 | ICD high |
| TCGA-4Z-AA81 | C1 | ICD high |
| TCGA-GC-A3RC | C1 | ICD high |
| TCGA-UY-A9PB | C1 | ICD high |
| TCGA-XF-A9SY | C1 | ICD high |
| TCGA-CF-A1HR | C2 | ICD low |
| TCGA-XF-A8HH | C2 | ICD low |
| TCGA-ZF-AA4W | C2 | ICD low |
| TCGA-XF-A9SU | C2 | ICD low |
| TCGA-DK-A3IQ | C2 | ICD low |
| TCGA-DK-A1A7 | C2 | ICD low |
| TCGA-CU-A5W6 | C2 | ICD low |
| TCGA-DK-A3X1 | C2 | ICD low |
| TCGA-XF-A9ST | C2 | ICD low |
| TCGA-CF-A9FM | C2 | ICD low |
| TCGA-GV-A3QF | C2 | ICD low |
| TCGA-DK-A6B2 | C2 | ICD low |
| TCGA-DK-A1AG | C2 | ICD low |
| TCGA-XF-A9SZ | C2 | ICD low |
| TCGA-E7-A519 | C2 | ICD low |
| TCGA-ZF-A9R1 | C2 | ICD low |
| TCGA-4Z-AA7S | C2 | ICD low |
| TCGA-BL-A13J | C2 | ICD low |
| TCGA-DK-A3IK | C2 | ICD low |
| TCGA-C4-A0F1 | C2 | ICD low |
| TCGA-DK-A3IS | C2 | ICD low |
| TCGA-E5-A4TZ | C2 | ICD low |
| TCGA-ZF-AA56 | C2 | ICD low |
| TCGA-YC-A9TC | C2 | ICD low |
| TCGA-CF-A7I0 | C2 | ICD low |
| TCGA-YF-AA3L | C2 | ICD low |
| TCGA-CF-A3MI | C2 | ICD low |
| TCGA-DK-A6B0 | C2 | ICD low |
| TCGA-DK-A6B1 | C2 | ICD low |
| TCGA-G2-AA3C | C2 | ICD low |
| TCGA-XF-AAN3 | C2 | ICD low |
| TCGA-CU-A0YO | C2 | ICD low |
| TCGA-BT-A3PJ | C2 | ICD low |
| TCGA-CF-A47Y | C2 | ICD low |
| TCGA-FD-A62O | C2 | ICD low |
| TCGA-BL-A0C8 | C2 | ICD low |
| TCGA-E7-A7DV | C2 | ICD low |
| TCGA-DK-A3WY | C2 | ICD low |
| TCGA-BT-A20N | C2 | ICD low |
| TCGA-GC-A3YS | C2 | ICD low |
| TCGA-FD-A43X | C2 | ICD low |
| TCGA-DK-A6AV | C2 | ICD low |
| TCGA-GU-A42Q | C2 | ICD low |
| TCGA-C4-A0F7 | C2 | ICD low |
| TCGA-CF-A47W | C2 | ICD low |
| TCGA-K4-A3WS | C2 | ICD low |
| TCGA-C4-A0EZ | C2 | ICD low |
| TCGA-CF-A47S | C2 | ICD low |
| TCGA-ZF-AA5P | C2 | ICD low |
| TCGA-CF-A27C | C2 | ICD low |
| TCGA-XF-A9T4 | C2 | ICD low |
| TCGA-FD-A3SM | C2 | ICD low |
| TCGA-KQ-A41P | C2 | ICD low |
| TCGA-C4-A0F6 | C2 | ICD low |
| TCGA-FD-A3B5 | C2 | ICD low |
| TCGA-4Z-AA80 | C2 | ICD low |
| TCGA-DK-A1AD | C2 | ICD low |
| TCGA-2F-A9KW | C2 | ICD low |
| TCGA-DK-AA6T | C2 | ICD low |
| TCGA-4Z-AA84 | C2 | ICD low |
| TCGA-FJ-A3Z9 | C2 | ICD low |
| TCGA-CU-A0YR | C2 | ICD low |
| TCGA-FD-A43N | C2 | ICD low |
| TCGA-BL-A13I | C2 | ICD low |
| TCGA-KQ-A41S | C2 | ICD low |
| TCGA-E7-A5KF | C2 | ICD low |
| TCGA-XF-AAN7 | C2 | ICD low |
| TCGA-DK-AA6M | C2 | ICD low |
| TCGA-CU-A3YL | C2 | ICD low |
| TCGA-C4-A0F0 | C2 | ICD low |
| TCGA-FD-A3SR | C2 | ICD low |
| TCGA-DK-A6B5 | C2 | ICD low |
| TCGA-HQ-A5ND | C2 | ICD low |
| TCGA-DK-AA76 | C2 | ICD low |
| TCGA-CF-A47V | C2 | ICD low |
| TCGA-E7-A3Y1 | C2 | ICD low |
| TCGA-GV-A40G | C2 | ICD low |
| TCGA-4Z-AA7M | C2 | ICD low |
| TCGA-BT-A2LD | C2 | ICD low |
| TCGA-4Z-AA82 | C2 | ICD low |
| TCGA-DK-AA6U | C2 | ICD low |
| TCGA-FD-A3N6 | C2 | ICD low |
| TCGA-2F-A9KR | C2 | ICD low |
| TCGA-CF-A3MF | C2 | ICD low |
| TCGA-CU-A3KJ | C2 | ICD low |
| TCGA-FD-A6TC | C2 | ICD low |
| TCGA-XF-A8HB | C2 | ICD low |
| TCGA-XF-AAN1 | C2 | ICD low |
| TCGA-FJ-A871 | C2 | ICD low |
| TCGA-UY-A9PA | C2 | ICD low |
| TCGA-XF-AAMZ | C2 | ICD low |
| TCGA-XF-AAMR | C2 | ICD low |
| TCGA-K4-A6FZ | C2 | ICD low |
| TCGA-DK-A1AF | C2 | ICD low |
| TCGA-GU-AATO | C2 | ICD low |
| TCGA-BT-A20U | C2 | ICD low |
| TCGA-DK-A1AA | C2 | ICD low |
| TCGA-GV-A3JW | C2 | ICD low |
| TCGA-FD-A5BZ | C2 | ICD low |
| TCGA-ZF-A9R9 | C2 | ICD low |
| TCGA-G2-AA3F | C2 | ICD low |
| TCGA-FD-A6TH | C2 | ICD low |
| TCGA-4Z-AA7O | C2 | ICD low |
| TCGA-E7-A6MF | C2 | ICD low |
| TCGA-DK-AA6W | C2 | ICD low |
| TCGA-GC-A3BM | C2 | ICD low |
| TCGA-GU-A42P | C2 | ICD low |
| TCGA-GV-A3QI | C2 | ICD low |
| TCGA-XF-A9T3 | C2 | ICD low |
| TCGA-E7-A8O7 | C2 | ICD low |
| TCGA-DK-AA77 | C2 | ICD low |
| TCGA-XF-AAML | C2 | ICD low |
| TCGA-2F-A9KQ | C2 | ICD low |
| TCGA-CF-A3MH | C2 | ICD low |
| TCGA-XF-A8HC | C2 | ICD low |
| TCGA-ZF-AA4T | C2 | ICD low |
| TCGA-FD-A6TI | C2 | ICD low |
| TCGA-H4-A2HO | C2 | ICD low |
| TCGA-CF-A47X | C2 | ICD low |
| TCGA-XF-A9SP | C2 | ICD low |
| TCGA-K4-A4AC | C2 | ICD low |
| TCGA-BL-A5ZZ | C2 | ICD low |
| TCGA-S5-AA26 | C2 | ICD low |
| TCGA-E7-A677 | C2 | ICD low |
| TCGA-DK-A1AC | C2 | ICD low |
| TCGA-DK-A2HX | C2 | ICD low |
| TCGA-UY-A8OC | C2 | ICD low |
| TCGA-FD-A5BR | C2 | ICD low |
| TCGA-HQ-A2OE | C2 | ICD low |
| TCGA-XF-AAMX | C2 | ICD low |
| TCGA-LT-A5Z6 | C2 | ICD low |
| TCGA-YC-A89H | C2 | ICD low |
| TCGA-E7-A8O8 | C2 | ICD low |
| TCGA-GC-A3OO | C2 | ICD low |
| TCGA-DK-AA75 | C2 | ICD low |
| TCGA-BT-A2LB | C2 | ICD low |
| TCGA-ZF-AA54 | C2 | ICD low |
| TCGA-FT-A3EE | C2 | ICD low |
| TCGA-FJ-A3ZF | C2 | ICD low |
| TCGA-DK-A3X2 | C2 | ICD low |
| TCGA-XF-AAME | C2 | ICD low |
| TCGA-ZF-AA4R | C2 | ICD low |
| TCGA-GV-A3JZ | C2 | ICD low |
| TCGA-DK-A2I2 | C2 | ICD low |
| TCGA-K4-A83P | C2 | ICD low |
| TCGA-4Z-AA7Y | C2 | ICD low |
| TCGA-2F-A9KP | C2 | ICD low |
| TCGA-UY-A78M | C2 | ICD low |
| TCGA-ZF-AA51 | C2 | ICD low |
| TCGA-GU-A42R | C2 | ICD low |
| TCGA-PQ-A6FI | C2 | ICD low |
| TCGA-ZF-AA4X | C2 | ICD low |
| TCGA-FD-A5BU | C2 | ICD low |
| TCGA-CF-A8HX | C2 | ICD low |
| TCGA-XF-A9SK | C2 | ICD low |
| TCGA-GD-A3OS | C2 | ICD low |
| TCGA-FD-A6TG | C2 | ICD low |
| TCGA-4Z-AA7N | C2 | ICD low |
| TCGA-GV-A3QK | C2 | ICD low |
| TCGA-K4-A5RI | C2 | ICD low |
| TCGA-FD-A43Y | C2 | ICD low |
| TCGA-DK-A3IV | C2 | ICD low |
| TCGA-G2-A2EL | C2 | ICD low |
| TCGA-ZF-A9RE | C2 | ICD low |
| TCGA-UY-A9PE | C2 | ICD low |
| TCGA-XF-AAMG | C2 | ICD low |
| TCGA-GU-A763 | C2 | ICD low |
| TCGA-G2-AA3D | C2 | ICD low |
| TCGA-5N-A9KM | C2 | ICD low |
| TCGA-ZF-A9RC | C2 | ICD low |
| TCGA-MV-A51V | C2 | ICD low |
| TCGA-S5-A6DX | C2 | ICD low |
| TCGA-FJ-A3ZE | C2 | ICD low |
| TCGA-YC-A8S6 | C2 | ICD low |
| TCGA-UY-A9PF | C2 | ICD low |
| TCGA-GV-A3JV | C2 | ICD low |
| TCGA-ZF-AA5N | C2 | ICD low |
| TCGA-5N-A9KI | C2 | ICD low |
| TCGA-CF-A3MG | C2 | ICD low |
| TCGA-GC-A4ZW | C2 | ICD low |
| TCGA-E7-A4XJ | C2 | ICD low |
| TCGA-FD-A3NA | C2 | ICD low |
| TCGA-DK-A3IT | C2 | ICD low |
| TCGA-R3-A69X | C2 | ICD low |
| TCGA-4Z-AA83 | C2 | ICD low |
| TCGA-ZF-A9R0 | C2 | ICD low |
| TCGA-GC-A3I6 | C2 | ICD low |
| TCGA-XF-A9SH | C2 | ICD low |
| TCGA-UY-A78L | C2 | ICD low |
| TCGA-CF-A8HY | C2 | ICD low |
| TCGA-CF-A9FL | C2 | ICD low |
| TCGA-E7-A6MD | C2 | ICD low |
| TCGA-CF-A9FF | C2 | ICD low |
| TCGA-XF-AAMY | C2 | ICD low |
| TCGA-G2-A3IE | C2 | ICD low |
| TCGA-GU-A767 | C2 | ICD low |
| TCGA-G2-A3IB | C2 | ICD low |
| TCGA-DK-A3IN | C2 | ICD low |
| TCGA-2F-A9KT | C2 | ICD low |
| TCGA-UY-A78N | C2 | ICD low |
| TCGA-DK-A1A5 | C2 | ICD low |
| TCGA-E7-A5KE | C2 | ICD low |
| TCGA-FD-A62S | C2 | ICD low |
| TCGA-G2-AA3B | C2 | ICD low |
| TCGA-XF-AAMW | C2 | ICD low |
| TCGA-GU-AATP | C2 | ICD low |
| TCGA-CU-A0YN | C2 | ICD low |
| TCGA-XF-AAMH | C2 | ICD low |
| TCGA-DK-A2I6 | C2 | ICD low |
| TCGA-XF-A8HI | C2 | ICD low |
| TCGA-DK-A2I1 | C2 | ICD low |
| TCGA-E7-A7PW | C2 | ICD low |
| TCGA-XF-AAMT | C2 | ICD low |
| TCGA-BT-A20R | C2 | ICD low |
| TCGA-H4-A2HQ | C2 | ICD low |
| TCGA-FD-A43S | C2 | ICD low |
| TCGA-DK-AA6P | C2 | ICD low |
| TCGA-XF-AAN0 | C2 | ICD low |
| TCGA-BT-A0YX | C2 | ICD low |
| TCGA-ZF-A9R5 | C2 | ICD low |
| TCGA-ZF-A9R3 | C2 | ICD low |
| TCGA-CF-A5UA | C2 | ICD low |
| TCGA-K4-A3WU | C2 | ICD low |
| TCGA-DK-AA71 | C2 | ICD low |
| TCGA-XF-A9T2 | C2 | ICD low |
| TCGA-FD-A5BY | C2 | ICD low |
| TCGA-BT-A20T | C2 | ICD low |
| TCGA-UY-A78O | C2 | ICD low |
| TCGA-4Z-AA89 | C2 | ICD low |
| TCGA-XF-A9SW | C2 | ICD low |
| TCGA-BT-A20W | C2 | ICD low |
| TCGA-FD-A3N5 | C2 | ICD low |
| TCGA-HQ-A5NE | C2 | ICD low |
| TCGA-DK-AA6R | C2 | ICD low |
| TCGA-BT-A42C | C2 | ICD low |
| TCGA-KQ-A41O | C2 | ICD low |
| TCGA-YF-AA3M | C2 | ICD low |
| TCGA-ZF-AA4N | C2 | ICD low |
| TCGA-G2-A2EC | C2 | ICD low |
| TCGA-CF-A5U8 | C2 | ICD low |
| TCGA-UY-A8OD | C2 | ICD low |
| TCGA-4Z-AA7R | C2 | ICD low |
| TCGA-FJ-A3Z7 | C2 | ICD low |
| TCGA-G2-A2EJ | C2 | ICD low |
| TCGA-SY-A9G0 | C2 | ICD low |
| TCGA-G2-A2EK | C2 | ICD low |
| TCGA-FD-A5BX | C2 | ICD low |
| TCGA-XF-AAN4 | C2 | ICD low |
| TCGA-FD-A3SO | C2 | ICD low |
| TCGA-FD-A6TA | C2 | ICD low |
| TCGA-FD-A3SS | C2 | ICD low |
| TCGA-BT-A3PK | C2 | ICD low |
| TCGA-XF-A9T6 | C2 | ICD low |
| TCGA-FD-A5BV | C2 | ICD low |
| TCGA-ZF-AA4U | C2 | ICD low |
| TCGA-KQ-A41R | C2 | ICD low |
| TCGA-GU-A764 | C2 | ICD low |
| TCGA-K4-AAQO | C2 | ICD low |
| TCGA-DK-AA6L | C2 | ICD low |
| TCGA-K4-A6MB | C2 | ICD low |
| TCGA-BT-A20P | C2 | ICD low |
| TCGA-FD-A3SJ | C2 | ICD low |
| TCGA-G2-A3VY | C2 | ICD low |
| TCGA-CU-A3QU | C2 | ICD low |
| TCGA-GC-A3RB | C2 | ICD low |
| TCGA-GU-AATQ | C2 | ICD low |
| TCGA-GC-A6I3 | C2 | ICD low |
| TCGA-GC-A3RD | C2 | ICD low |
| TCGA-GD-A3OQ | C2 | ICD low |
| TCGA-CF-A47T | C2 | ICD low |
| TCGA-ZF-AA52 | C2 | ICD low |
| TCGA-XF-A8HG | C2 | ICD low |
| TCGA-DK-A3IL | C2 | ICD low |
| TCGA-E7-A6ME | C2 | ICD low |
| TCGA-GD-A2C5 | C2 | ICD low |
| TCGA-DK-AA6X | C2 | ICD low |
| TCGA-UY-A9PD | C2 | ICD low |
| TCGA-DK-A3IM | C2 | ICD low |
| TCGA-FD-A3SL | C2 | ICD low |
| TCGA-BT-A3PH | C2 | ICD low |
| TCGA-FD-A3SQ | C2 | ICD low |
| TCGA-DK-A1A3 | C2 | ICD low |
| TCGA-BT-A2LA | C2 | ICD low |
| TCGA-PQ-A6FN | C2 | ICD low |
| TCGA-GD-A3OP | C2 | ICD low |
| TCGA-BL-A3JM | C2 | ICD low |
| TCGA-CU-A72E | C2 | ICD low |
| TCGA-FD-A3SN | C2 | ICD low |
| TCGA-GV-A3QH | C2 | ICD low |
| TCGA-FD-A6TF | C2 | ICD low |
| TCGA-ZF-A9RL | C2 | ICD low |
| TCGA-E7-A678 | C2 | ICD low |
| TCGA-GD-A76B | C2 | ICD low |
| TCGA-GV-A6ZA | C2 | ICD low |
| TCGA-BT-A20X | C2 | ICD low |
| TCGA-K4-A3WV | C2 | ICD low |
| TCGA-FD-A5C0 | C2 | ICD low |
| TCGA-DK-A1AE | C2 | ICD low |
| TCGA-LT-A8JT | C2 | ICD low |
| TCGA-K4-A4AB | C2 | ICD low |
| TCGA-FD-A43U | C2 | ICD low |
| TCGA-FD-A6TE | C2 | ICD low |
| TCGA-XF-A9SV | C2 | ICD low |
| TCGA-GD-A6C6 | C2 | ICD low |
| TCGA-KQ-A41N | C2 | ICD low |
| TCGA-KQ-A41Q | C2 | ICD low |
| TCGA-XF-A9SL | C2 | ICD low |
| TCGA-DK-A1AB | C2 | ICD low |
| TCGA-XF-A9T0 | C2 | ICD low |
| TCGA-XF-AAN8 | C2 | ICD low |
| TCGA-XF-A8HF | C2 | ICD low |
| TCGA-E5-A2PC | C2 | ICD low |
| TCGA-ZF-A9R2 | C2 | ICD low |
| TCGA-E5-A4U1 | C2 | ICD low |
| TCGA-BT-A0S7 | C2 | ICD low |
| TCGA-ZF-A9RM | C2 | ICD low |
| TCGA-E7-A7DU | C2 | ICD low |
| TCGA-XF-AAMJ | C2 | ICD low |
|  |  |  |
