# Supplementary material for: Development and validation of a model based on immunogenic cell death related genes to predict the prognosis and immune response to bladder urothelial carcinoma
Source: Front Oncol. 2023 Nov 10;13:1291720. doi: 10.3389/fonc.2023.1291720 (PMC10676223; doi:10.3389/fonc.2023.1291720)
Supplement: Supplementary file 6 [file Table_6.docx]

**Supplementary Table 6 The list of differentially expressed genes (DEGs) in ICD -high and -low groups**

| Gene | LowMean | HighMean | LogFC | Pvalue | Fdr |
| --- | --- | --- | --- | --- | --- |
| *IFNG* | 0.409871698 | 7.806232143 | 4.251382087 | 2.91E-45 | 7.15E-41 |
| *FASLG* | 0.605001572 | 5.423960714 | 3.164335932 | 1.41E-41 | 1.73E-37 |
| *NKG7* | 12.03408836 | 114.5427607 | 3.25068752 | 4.37E-41 | 3.58E-37 |
| *GZMH* | 3.733333333 | 44.2944381 | 3.568589324 | 7.09E-41 | 4.36E-37 |
| *GZMB* | 18.62394308 | 85.18884286 | 2.19350594 | 1.81E-40 | 8.89E-37 |
| *LAG3* | 3.83380566 | 34.76748929 | 3.180889775 | 2.92E-40 | 1.20E-36 |
| *TBX21* | 0.580447799 | 4.129179762 | 2.830616994 | 1.66E-39 | 5.85E-36 |
| *LINC02446* | 0.641336164 | 12.00728452 | 4.226685349 | 1.99E-39 | 6.11E-36 |
| *CCL4* | 6.270995283 | 43.39896786 | 2.790894392 | 2.46E-39 | 6.72E-36 |
| *PRF1* | 8.35062044 | 48.16152976 | 2.52792592 | 3.95E-39 | 9.71E-36 |
| *CD8A* | 4.589598113 | 38.89693095 | 3.083216593 | 6.24E-39 | 1.40E-35 |
| *PDCD1* | 1.951063836 | 12.15584762 | 2.639317608 | 2.81E-38 | 5.76E-35 |
| *GBP5* | 4.326373899 | 57.48752143 | 3.732018572 | 5.81E-38 | 1.10E-34 |
| *SLA2* | 1.515792453 | 9.112360714 | 2.587752629 | 6.84E-38 | 1.20E-34 |
| *CXCR6* | 2.264489937 | 11.4611881 | 2.339498572 | 1.13E-37 | 1.84E-34 |
| *GZMA* | 27.66733522 | 94.38215476 | 1.770330411 | 1.20E-37 | 1.84E-34 |
| *LINC01871* | 4.641851887 | 31.7507381 | 2.774017735 | 1.56E-37 | 2.26E-34 |
| *AC022126.1* | 0.179781132 | 1.626683333 | 3.177619905 | 9.31E-37 | 1.27E-33 |
| *CCR5* | 2.62373805 | 15.17773929 | 2.532261322 | 1.24E-36 | 1.61E-33 |
| *CD2* | 10.70428333 | 58.33114286 | 2.446078132 | 1.35E-36 | 1.66E-33 |
| *LINC02195* | 1.708227358 | 14.15116071 | 3.050348481 | 1.71E-36 | 2.00E-33 |
| *ZNF683* | 1.135642453 | 14.7093131 | 3.695149285 | 2.70E-36 | 3.02E-33 |
| *TRGC2* | 1.11122327 | 8.081508333 | 2.862475866 | 3.01E-36 | 3.22E-33 |
| *CXCR3* | 2.028167296 | 12.67797738 | 2.644076035 | 6.52E-36 | 6.68E-33 |
| *CXCL9* | 24.65012453 | 277.15505 | 3.491026455 | 7.54E-36 | 7.42E-33 |
| *TIGIT* | 1.869307547 | 8.05085119 | 2.106637375 | 1.58E-35 | 1.50E-32 |
| *CD7* | 6.584187736 | 34.19516905 | 2.376715144 | 2.48E-35 | 2.26E-32 |
| *CD3E* | 9.113149371 | 44.2194631 | 2.278659889 | 2.94E-35 | 2.59E-32 |
| *CALHM6* | 1.008275157 | 11.49933929 | 3.511589663 | 3.06E-35 | 2.60E-32 |
| *AC243829.4* | 0.099390566 | 0.606011905 | 2.608165309 | 3.19E-35 | 2.61E-32 |
| *IRF1* | 19.43979057 | 65.95345238 | 1.762435504 | 5.61E-35 | 4.45E-32 |
| *CCL5* | 95.53524969 | 359.5874583 | 1.912237655 | 5.83E-35 | 4.48E-32 |
| *FTH1P22* | 0.482734591 | 3.512269048 | 2.863101248 | 1.02E-34 | 7.61E-32 |
| *CXCL10* | 59.39033868 | 642.0553452 | 3.434397498 | 1.17E-34 | 8.44E-32 |
| *IL12RB1* | 1.954029874 | 10.09401429 | 2.368975604 | 1.58E-34 | 1.11E-31 |
| *GNLY* | 4.958233019 | 47.98977857 | 3.274829177 | 1.66E-34 | 1.13E-31 |
| *WARS1* | 63.68210189 | 438.0401667 | 2.782103306 | 1.94E-34 | 1.25E-31 |
| *TRAC* | 35.37382893 | 159.949956 | 2.176866302 | 1.94E-34 | 1.25E-31 |
| *CXCR2P1* | 0.609195597 | 8.156655952 | 3.74300038 | 2.41E-34 | 1.52E-31 |
| *SH2D1A* | 1.117277358 | 5.558272619 | 2.314649225 | 2.61E-34 | 1.61E-31 |
| *PSMB9* | 41.35654308 | 196.4541786 | 2.248005351 | 4.28E-34 | 2.48E-31 |
| *AL031846.1* | 0.380091509 | 2.540121429 | 2.740478762 | 4.29E-34 | 2.48E-31 |

| *CD3G* | 1.298792767 | 6.370629762 | 2.29426474 | 4.33E-34 | 2.48E-31 |
| --- | --- | --- | --- | --- | --- |
| *KIR2DL4* | 0.567654403 | 4.448365476 | 2.970190559 | 5.90E-34 | 3.30E-31 |
| *SIRPG* | 2.505215409 | 10.25994286 | 2.034016133 | 7.17E-34 | 3.92E-31 |
| *IL2RB* | 6.407859434 | 24.38835833 | 1.928278243 | 7.55E-34 | 3.95E-31 |
| *GBP1* | 38.15193553 | 210.5267798 | 2.464175606 | 7.55E-34 | 3.95E-31 |
| *CD3D* | 15.15251541 | 69.84288095 | 2.20455576 | 8.81E-34 | 4.51E-31 |
| *CASP5* | 0.331630503 | 2.455520238 | 2.888380097 | 1.12E-33 | 5.63E-31 |
| *TAF5LP1* | 1.134250943 | 5.9858 | 2.399804216 | 1.36E-33 | 6.69E-31 |
| *KLRD1* | 0.33236195 | 1.714839286 | 2.367246243 | 1.60E-33 | 7.72E-31 |
| *UBASH3A* | 0.645221698 | 3.013645238 | 2.223642734 | 2.22E-33 | 1.05E-30 |
| *CXCL11* | 13.9070956 | 174.9854679 | 3.653342055 | 3.14E-33 | 1.45E-30 |
| *PYHIN1* | 0.817836478 | 3.728864286 | 2.188851972 | 3.65E-33 | 1.66E-30 |
| *TRG-AS1* | 0.27988522 | 1.24372381 | 2.151758936 | 4.68E-33 | 2.09E-30 |
| *IDO1* | 11.42169497 | 149.1367512 | 3.706787153 | 6.40E-33 | 2.81E-30 |
| *THEMIS* | 0.420437107 | 2.077992857 | 2.305228786 | 7.43E-33 | 3.21E-30 |
| *GPR174* | 0.588739623 | 2.541697619 | 2.110090775 | 1.40E-32 | 5.94E-30 |
| *IL18RAP* | 0.656966667 | 2.974363095 | 2.178688697 | 1.80E-32 | 7.48E-30 |
| *HAVCR2* | 7.22573805 | 29.46090714 | 2.027584994 | 3.75E-32 | 1.54E-29 |
| *TOMM20P2* | 0.359100314 | 2.175228571 | 2.598708185 | 4.75E-32 | 1.91E-29 |
| *TAP1* | 63.75787421 | 269.9901952 | 2.082231583 | 4.82E-32 | 1.91E-29 |
| *ITGAL* | 2.733704403 | 10.63403571 | 1.95976006 | 5.26E-32 | 2.05E-29 |
| *CCL3* | 1.29372673 | 6.378254762 | 2.30162881 | 6.34E-32 | 2.44E-29 |
| *CRTAM* | 0.693501572 | 3.355840476 | 2.274703079 | 6.92E-32 | 2.62E-29 |
| *PTPN7* | 3.526441824 | 11.24025357 | 1.672389437 | 7.05E-32 | 2.63E-29 |
| *UBE2L6* | 133.3575638 | 387.4069667 | 1.538550243 | 8.89E-32 | 3.26E-29 |
| *ZAP70* | 2.625998742 | 9.562582143 | 1.86453401 | 1.17E-31 | 4.23E-29 |
| *CTLA4* | 2.777270755 | 12.26628095 | 2.142958164 | 1.43E-31 | 5.08E-29 |
| *TMEM150B* | 1.497411635 | 6.927391667 | 2.209841375 | 1.57E-31 | 5.53E-29 |
| *SAMD3* | 0.147651572 | 0.665179762 | 2.171547556 | 1.61E-31 | 5.59E-29 |
| *LINC02528* | 0.133005975 | 1.502914286 | 3.498199771 | 2.17E-31 | 7.41E-29 |
| *TRBC1* | 22.33228931 | 90.83920833 | 2.024183982 | 2.49E-31 | 8.40E-29 |
| *CD247* | 3.109486792 | 10.44157619 | 1.747591113 | 3.44E-31 | 1.14E-28 |
| *GPR171* | 1.375058805 | 6.506138095 | 2.242308127 | 3.84E-31 | 1.26E-28 |
| *TBC1D10C* | 3.372781761 | 11.45927976 | 1.764505495 | 3.94E-31 | 1.26E-28 |
| *LCK* | 6.469248113 | 20.92609167 | 1.693632937 | 3.94E-31 | 1.26E-28 |
| *SIT1* | 4.300993711 | 12.3508631 | 1.521869936 | 4.35E-31 | 1.35E-28 |
| *LILRP2* | 0.096117296 | 0.584479762 | 2.604285109 | 4.38E-31 | 1.35E-28 |
| *GPR25* | 0.237790252 | 2.058986905 | 3.114173178 | 4.40E-31 | 1.35E-28 |
| *BATF2* | 7.295621384 | 42.8050369 | 2.552677804 | 5.30E-31 | 1.61E-28 |
| *GRAP2* | 0.637692138 | 2.418446429 | 1.92314858 | 6.69E-31 | 2.01E-28 |
| *IL21R* | 1.163239937 | 5.155369048 | 2.147927 | 7.75E-31 | 2.27E-28 |
| *GBP4* | 21.30151069 | 97.86515952 | 2.199839595 | 7.75E-31 | 2.27E-28 |
| *ICOS* | 1.344961635 | 5.741489286 | 2.093859986 | 8.04E-31 | 2.33E-28 |
| *ARHGAP9* | 3.572958491 | 11.85037738 | 1.729741945 | 8.35E-31 | 2.39E-28 |
| *KLRC1* | 0.884124528 | 2.148429762 | 1.280961121 | 9.75E-31 | 2.76E-28 |
| *SPN* | 1.758239937 | 6.468258333 | 1.879245339 | 1.08E-30 | 3.02E-28 |

| *TNFRSF9* | 0.949113208 | 3.993519048 | 2.073008512 | 1.11E-30 | 3.06E-28 |
| --- | --- | --- | --- | --- | --- |
| *PTPN22* | 1.456605031 | 4.955145238 | 1.766317611 | 1.64E-30 | 4.47E-28 |
| *LCP2* | 4.988577673 | 16.95780952 | 1.765249383 | 1.80E-30 | 4.88E-28 |
| *TRGV10* | 0.484873585 | 2.780664286 | 2.519749011 | 1.98E-30 | 5.28E-28 |
| *EOMES* | 0.547913208 | 3.184735714 | 2.53915437 | 2.04E-30 | 5.38E-28 |
| *GBP1P1* | 5.513450629 | 22.43382738 | 2.024648349 | 2.94E-30 | 7.68E-28 |
| *ADGRG5* | 0.897817296 | 3.085739286 | 1.78112238 | 3.63E-30 | 9.36E-28 |
| *CD6* | 2.713624843 | 9.853384524 | 1.860398076 | 3.66E-30 | 9.36E-28 |
| *AC015911.3* | 0.408015723 | 2.004328571 | 2.296422376 | 3.91E-30 | 9.92E-28 |
| *ACOD1* | 0.049453459 | 0.402280952 | 3.024060088 | 4.20E-30 | 1.05E-27 |
| *STAT1* | 71.43594371 | 242.491244 | 1.763210586 | 4.38E-30 | 1.09E-27 |
| *TAP2* | 17.23741635 | 53.43329167 | 1.632195344 | 4.95E-30 | 1.22E-27 |
| *ZBP1* | 1.383334277 | 8.280116667 | 2.581501276 | 5.07E-30 | 1.23E-27 |
| *HMGB3P32* | 0.102100314 | 0.566913095 | 2.473140285 | 5.09E-30 | 1.23E-27 |
| *CST7* | 13.62931352 | 51.2792369 | 1.911661894 | 6.54E-30 | 1.56E-27 |
| *SP140* | 1.772289308 | 6.556858333 | 1.887390596 | 7.38E-30 | 1.74E-27 |
| *IL2RG* | 7.246114465 | 31.4316381 | 2.116937961 | 7.74E-30 | 1.80E-27 |
| *FCER1G* | 62.2805827 | 220.7699702 | 1.825689598 | 7.74E-30 | 1.80E-27 |
| *TTC24* | 0.197415409 | 0.773539286 | 1.970239963 | 9.04E-30 | 2.08E-27 |
| *CCL4L2* | 4.529842453 | 25.015275 | 2.465276533 | 9.91E-30 | 2.26E-27 |
| *LAP3* | 62.96303491 | 183.6072167 | 1.544045777 | 1.05E-29 | 2.36E-27 |
| *C1QA* | 219.6995053 | 1003.350079 | 2.191221537 | 1.07E-29 | 2.40E-27 |
| *DOK2* | 8.592353774 | 31.60825 | 1.879175862 | 1.22E-29 | 2.71E-27 |
| *RASAL3* | 4.687546855 | 14.43102976 | 1.622269234 | 1.35E-29 | 2.96E-27 |
| *TNIP3* | 0.72155566 | 2.532666667 | 1.811474619 | 1.39E-29 | 3.03E-27 |
| *KIR3DL2* | 0.096878616 | 1.46145 | 3.915078401 | 1.45E-29 | 3.14E-27 |
| *TNFSF13B* | 4.423657233 | 19.64435238 | 2.150803099 | 1.65E-29 | 3.53E-27 |
| *CD8B* | 1.550625472 | 6.764409524 | 2.125113736 | 1.69E-29 | 3.56E-27 |
| *SLAMF6* | 2.363460692 | 9.572202381 | 2.017950029 | 1.69E-29 | 3.56E-27 |
| *LILRB4* | 3.739472327 | 21.52053571 | 2.524807379 | 1.71E-29 | 3.57E-27 |
| *FCGR3A* | 37.69481604 | 168.4224476 | 2.1596464 | 1.98E-29 | 4.09E-27 |
| *C1QB* | 181.5699047 | 839.0400929 | 2.208214655 | 2.10E-29 | 4.31E-27 |
| *TRAT1* | 0.546172956 | 2.599854762 | 2.251001246 | 2.19E-29 | 4.46E-27 |
| *GZMM* | 3.36244717 | 12.15930833 | 1.85447766 | 2.45E-29 | 4.95E-27 |
| *PSTPIP1* | 3.104527673 | 9.437438095 | 1.604021484 | 2.48E-29 | 4.97E-27 |
| *TRDC* | 1.860696541 | 12.11625238 | 2.703028843 | 2.51E-29 | 4.97E-27 |
| *HK3* | 3.786536164 | 15.68754286 | 2.050668788 | 2.64E-29 | 5.19E-27 |
| *ITK* | 0.891757233 | 3.467272619 | 1.959078358 | 2.72E-29 | 5.29E-27 |
| *PIK3AP1* | 4.753815094 | 15.07482143 | 1.664983218 | 2.73E-29 | 5.29E-27 |
| *MIR155HG* | 1.810775786 | 4.748194048 | 1.390770976 | 3.35E-29 | 6.43E-27 |
| *NLRC3* | 1.360625157 | 3.895270238 | 1.517453751 | 3.49E-29 | 6.66E-27 |
| *C1QC* | 175.4604381 | 731.7488119 | 2.060202723 | 3.55E-29 | 6.72E-27 |
| *IL15RA* | 11.95954937 | 41.07513333 | 1.780102228 | 4.00E-29 | 7.52E-27 |
| *ACAP1* | 4.028371384 | 10.61337857 | 1.397615385 | 4.41E-29 | 8.21E-27 |
| *CD74* | 734.6761044 | 2642.389424 | 1.846662844 | 4.51E-29 | 8.34E-27 |
| *BIN2* | 3.420583333 | 10.89910119 | 1.671894883 | 5.59E-29 | 1.03E-26 |

| *APOL3* | 13.18266855 | 43.2505631 | 1.714076473 | 5.93E-29 | 1.08E-26 |
| --- | --- | --- | --- | --- | --- |
| *KLRC2* | 0.408342767 | 1.400379762 | 1.777965538 | 6.19E-29 | 1.12E-26 |
| *HLA-DQA1* | 25.13754906 | 123.7310845 | 2.299292092 | 7.17E-29 | 1.29E-26 |
| *P2RY10* | 1.500549057 | 4.966170238 | 1.726643232 | 7.26E-29 | 1.29E-26 |
| *CD274* | 5.395174214 | 27.94301786 | 2.37274639 | 8.27E-29 | 1.45E-26 |
| *SASH3* | 8.086768239 | 24.22081548 | 1.582612269 | 8.27E-29 | 1.45E-26 |
| *SLAMF8* | 1.607867925 | 8.121402381 | 2.336579966 | 8.98E-29 | 1.57E-26 |
| *IL21* | 0.06333805 | 0.34895119 | 2.46188089 | 9.36E-29 | 1.62E-26 |
| *PDCD1LG2* | 3.231045283 | 17.37452857 | 2.426900958 | 1.12E-28 | 1.93E-26 |
| *MYO1G* | 2.709908176 | 8.409170238 | 1.633719484 | 1.18E-28 | 2.00E-26 |
| *SLAMF1* | 1.553727044 | 3.432869048 | 1.143681748 | 1.18E-28 | 2.00E-26 |
| *OR2I1P* | 4.28492044 | 35.70558929 | 3.058809606 | 1.33E-28 | 2.24E-26 |
| *LILRB2* | 4.271012893 | 17.49190714 | 2.034037437 | 1.36E-28 | 2.27E-26 |
| *FCRL6* | 0.429983648 | 1.944245238 | 2.176856505 | 1.45E-28 | 2.42E-26 |
| *FCGR1B* | 0.320727044 | 1.15184881 | 1.844533449 | 1.58E-28 | 2.62E-26 |
| *NLRC5* | 8.564080189 | 25.49375952 | 1.573773932 | 1.62E-28 | 2.66E-26 |
| *SNX20* | 2.024341195 | 6.609811905 | 1.707156746 | 1.98E-28 | 3.23E-26 |
| *CCL8* | 6.33331478 | 45.90720833 | 2.857688011 | 2.00E-28 | 3.24E-26 |
| *HAPLN3* | 14.11934308 | 55.488825 | 1.974524286 | 2.08E-28 | 3.34E-26 |
| *SCML4* | 0.218610377 | 0.942046429 | 2.107436278 | 2.18E-28 | 3.47E-26 |
| *LGALS17A* | 0.334183019 | 5.895302381 | 4.140855482 | 2.21E-28 | 3.50E-26 |
| *TRAV17* | 0.50481195 | 2.383214286 | 2.23909071 | 2.83E-28 | 4.46E-26 |
| *FCGR1A* | 2.225656604 | 8.935453571 | 2.005309947 | 3.10E-28 | 4.85E-26 |
| *LINC00426* | 0.385623585 | 1.314564286 | 1.7693195 | 3.15E-28 | 4.90E-26 |
| *MAP4K1* | 3.432962893 | 9.232059524 | 1.427198263 | 3.17E-28 | 4.90E-26 |
| *HLA-DRA* | 888.4126997 | 3257.856876 | 1.874621304 | 3.82E-28 | 5.88E-26 |
| *PSME2* | 126.4122701 | 271.0645012 | 1.100499685 | 3.91E-28 | 5.98E-26 |
| *CXCL13* | 29.39360692 | 193.0711417 | 2.715558231 | 4.01E-28 | 6.08E-26 |
| *ISG20* | 5.962751572 | 21.06677262 | 1.820919178 | 4.50E-28 | 6.80E-26 |
| *CD53* | 29.45173931 | 89.94462857 | 1.610684291 | 5.18E-28 | 7.77E-26 |
| *CD226* | 0.306234277 | 0.898825 | 1.55340448 | 5.21E-28 | 7.77E-26 |
| *TRGC1* | 0.258863522 | 1.227291667 | 2.245214561 | 5.38E-28 | 7.96E-26 |
| *WAS* | 10.81610189 | 28.10666071 | 1.377731414 | 6.70E-28 | 9.87E-26 |
| *AOAH* | 3.370699371 | 14.08635833 | 2.063178822 | 9.28E-28 | 1.36E-25 |
| *CYTIP* | 5.589265409 | 17.46054286 | 1.643367825 | 1.12E-27 | 1.63E-25 |
| *CD300LF* | 2.188944969 | 8.428902381 | 1.94510909 | 1.44E-27 | 2.09E-25 |
| *RHOH* | 1.70940283 | 4.893377381 | 1.517338133 | 1.49E-27 | 2.15E-25 |
| *SLAMF7* | 6.156034277 | 26.51582976 | 2.106780726 | 1.68E-27 | 2.40E-25 |
| *CD48* | 2.547651572 | 7.524819048 | 1.562488907 | 1.85E-27 | 2.63E-25 |
| *HLA-B* | 1458.107358 | 4694.937883 | 1.687009124 | 1.93E-27 | 2.72E-25 |
| *NCR1* | 0.262042453 | 0.971542857 | 1.89047708 | 1.97E-27 | 2.76E-25 |
| *CD52* | 32.01084811 | 79.84370833 | 1.318617827 | 1.99E-27 | 2.79E-25 |
| *NCF1* | 1.693783962 | 5.597763095 | 1.724600557 | 2.05E-27 | 2.85E-25 |
| *TLR8* | 0.88552956 | 4.148022619 | 2.22781139 | 2.34E-27 | 3.24E-25 |
| *CYTH4* | 4.926227987 | 14.6922369 | 1.576498761 | 2.43E-27 | 3.33E-25 |
| *SELPLG* | 13.54247767 | 43.4407631 | 1.681557734 | 2.51E-27 | 3.43E-25 |

| *AC004847.1* | 1.007744654 | 2.631853571 | 1.384949094 | 2.57E-27 | 3.47E-25 |
| --- | --- | --- | --- | --- | --- |
| *IL18BP* | 7.950437736 | 24.00580476 | 1.594277101 | 2.57E-27 | 3.47E-25 |
| *SAMHD1* | 20.11472013 | 51.93632262 | 1.368492202 | 2.72E-27 | 3.66E-25 |
| *CD5* | 3.336569182 | 11.42840952 | 1.776187317 | 3.24E-27 | 4.33E-25 |
| *IL10RA* | 4.776510692 | 15.08729881 | 1.659305534 | 3.76E-27 | 5.00E-25 |
| *CD86* | 6.454265723 | 22.34719524 | 1.791768892 | 3.85E-27 | 5.08E-25 |
| *CORO1A* | 26.71168994 | 62.98867024 | 1.237621102 | 3.89E-27 | 5.12E-25 |
| *LINC01358* | 0.126945597 | 0.422028571 | 1.73313031 | 4.14E-27 | 5.39E-25 |
| *SIGLEC1* | 4.319671698 | 14.90308095 | 1.786617039 | 4.14E-27 | 5.39E-25 |
| *PTPRC* | 6.896869497 | 21.82456905 | 1.661939593 | 4.26E-27 | 5.52E-25 |
| *HLA-DMB* | 11.98581069 | 38.85887381 | 1.696916597 | 4.31E-27 | 5.55E-25 |
| *HCST* | 3.027372013 | 10.67014048 | 1.817441296 | 4.86E-27 | 6.23E-25 |
| *FMNL1* | 7.642342767 | 20.68118452 | 1.436231948 | 4.89E-27 | 6.24E-25 |
| *MYO7A* | 2.519340881 | 5.819753571 | 1.207911726 | 5.09E-27 | 6.46E-25 |
| *LINC02539* | 0.081699057 | 0.443972619 | 2.44207938 | 6.37E-27 | 8.03E-25 |
| *SIGLEC10* | 2.822442767 | 14.64751786 | 2.375639977 | 6.51E-27 | 8.17E-25 |
| *NCF1C* | 2.789189308 | 8.571222619 | 1.619655152 | 7.22E-27 | 9.01E-25 |
| *FERMT3* | 17.26232893 | 46.53834881 | 1.430792907 | 1.01E-26 | 1.25E-24 |
| *TOGARAM2* | 0.194472642 | 0.55867381 | 1.522438978 | 1.02E-26 | 1.26E-24 |
| *TRBV9* | 0.834801572 | 4.815052381 | 2.52804627 | 1.07E-26 | 1.31E-24 |
| *SPI1* | 22.00636384 | 67.7929381 | 1.623214212 | 1.18E-26 | 1.44E-24 |
| *MYO1F* | 4.614110692 | 13.30711548 | 1.528073359 | 1.41E-26 | 1.72E-24 |
| *PLEK* | 11.29598742 | 39.60317381 | 1.809805666 | 1.43E-26 | 1.73E-24 |
| *GZMK* | 0.922243082 | 5.233117857 | 2.504451784 | 1.44E-26 | 1.73E-24 |
| *LTA* | 0.895401258 | 2.588083333 | 1.531277821 | 1.46E-26 | 1.75E-24 |
| *IGSF6* | 4.657217925 | 14.2937869 | 1.617847889 | 1.51E-26 | 1.81E-24 |
| *SLA* | 4.350373585 | 13.60349286 | 1.644765926 | 1.55E-26 | 1.84E-24 |
| *TYROBP* | 93.42273931 | 284.9731595 | 1.608980391 | 1.58E-26 | 1.87E-24 |
| *HLA-DRB1* | 602.6968513 | 2424.483686 | 2.008173113 | 1.62E-26 | 1.91E-24 |
| *TRBV2* | 0.563852201 | 3.056329762 | 2.438411258 | 1.64E-26 | 1.92E-24 |
| *GPR65* | 1.006665094 | 2.991538095 | 1.571303639 | 1.65E-26 | 1.92E-24 |
| *IL2RA* | 3.06692956 | 12.63027024 | 2.042018571 | 1.75E-26 | 2.03E-24 |
| *NFAM1* | 2.537173899 | 9.145357143 | 1.849817105 | 1.77E-26 | 2.04E-24 |
| *IL27* | 0.374966038 | 1.48874881 | 1.989268519 | 1.85E-26 | 2.12E-24 |
| *TRBV5-1* | 1.069735849 | 4.253229762 | 1.991304198 | 1.88E-26 | 2.14E-24 |
| *USP30-AS1* | 0.714101887 | 2.978977381 | 2.060615334 | 2.16E-26 | 2.45E-24 |
| *HLA-F* | 42.14054811 | 162.8514464 | 1.950275549 | 2.20E-26 | 2.48E-24 |
| *HLA-DPB1* | 61.87492327 | 210.3772821 | 1.765552186 | 2.25E-26 | 2.53E-24 |
| *IFI35* | 64.93846604 | 177.8304952 | 1.453357535 | 2.36E-26 | 2.63E-24 |
| *CYBB* | 17.49856792 | 59.79585833 | 1.772808705 | 2.70E-26 | 3.00E-24 |
| *BCL2A1* | 9.453750943 | 34.73441429 | 1.877407007 | 2.79E-26 | 3.09E-24 |
| *TRBV4-1* | 0.480127358 | 3.177558333 | 2.72642956 | 2.95E-26 | 3.25E-24 |
| *B2M* | 1154.707674 | 2881.903919 | 1.319494573 | 3.38E-26 | 3.71E-24 |
| *ITGB2* | 19.35686226 | 67.85998929 | 1.809716091 | 3.91E-26 | 4.28E-24 |
| *FUT7* | 0.69016761 | 2.444089286 | 1.824278316 | 3.95E-26 | 4.29E-24 |
| *PSMB8* | 81.18168553 | 199.2951143 | 1.295680144 | 4.05E-26 | 4.39E-24 |

| *AL021978.1* | 0.788804088 | 4.543196429 | 2.525968749 | 4.14E-26 | 4.46E-24 |
| --- | --- | --- | --- | --- | --- |
| *DBH-AS1* | 1.206863522 | 4.218480952 | 1.805461049 | 4.21E-26 | 4.52E-24 |
| *ETV7* | 16.51090692 | 50.27859643 | 1.606525009 | 5.49E-26 | 5.87E-24 |
| *AIF1* | 34.49774748 | 111.0098417 | 1.686113516 | 6.00E-26 | 6.39E-24 |
| *GNGT2* | 1.614861635 | 4.880742857 | 1.595690188 | 6.03E-26 | 6.40E-24 |
| *PILRA* | 5.392025472 | 18.24270238 | 1.758420242 | 6.14E-26 | 6.45E-24 |
| *PSME2P2* | 5.362360692 | 11.83503333 | 1.142123601 | 6.14E-26 | 6.45E-24 |
| *IL15* | 1.454950314 | 4.471825 | 1.619893844 | 6.17E-26 | 6.46E-24 |
| *MNDA* | 6.306299057 | 21.43597976 | 1.765168867 | 6.21E-26 | 6.47E-24 |
| *SOCS1* | 9.067067296 | 26.76186429 | 1.561470723 | 7.26E-26 | 7.54E-24 |
| *CD27* | 3.096157233 | 9.523984524 | 1.621086538 | 7.34E-26 | 7.59E-24 |
| *LINC01943* | 1.214384277 | 3.655141667 | 1.589702307 | 7.94E-26 | 8.17E-24 |
| *IKZF1* | 2.077528302 | 5.655108333 | 1.444686531 | 8.12E-26 | 8.29E-24 |
| *CIITA* | 3.046494025 | 9.38322381 | 1.622933766 | 8.12E-26 | 8.29E-24 |
| *CD80* | 0.635721069 | 2.491128571 | 1.970333675 | 8.58E-26 | 8.72E-24 |
| *TRAV4* | 0.743346541 | 3.514966667 | 2.241404165 | 9.33E-26 | 9.44E-24 |
| *TAGAP* | 2.319144654 | 6.59417381 | 1.507599105 | 9.71E-26 | 9.79E-24 |
| *LILRB3* | 2.994896541 | 9.731265476 | 1.700121263 | 1.02E-25 | 1.02E-23 |
| *LINC00996* | 0.485697484 | 1.62192381 | 1.739576131 | 1.15E-25 | 1.15E-23 |
| *HLA-E* | 693.7591626 | 1466.950398 | 1.080313264 | 1.17E-25 | 1.17E-23 |
| *TYMP* | 81.72539686 | 281.4314131 | 1.783926987 | 1.28E-25 | 1.27E-23 |
| *EVI2B* | 8.007558491 | 22.97237619 | 1.520465756 | 1.33E-25 | 1.31E-23 |
| *HLA-C* | 1418.307514 | 3461.983126 | 1.287428325 | 1.47E-25 | 1.44E-23 |
| *C11orf21* | 0.427064465 | 1.478161905 | 1.791278532 | 1.53E-25 | 1.50E-23 |
| *TRGV2* | 0.137531132 | 0.818630952 | 2.573454986 | 1.63E-25 | 1.59E-23 |
| *HLA-DQB1* | 35.32847893 | 154.8821048 | 2.132266923 | 1.64E-25 | 1.59E-23 |
| *IL32* | 38.58221101 | 147.1522976 | 1.931302342 | 1.85E-25 | 1.79E-23 |
| *PSMB8-AS1* | 5.571746226 | 13.76617738 | 1.30492655 | 2.07E-25 | 2.00E-23 |
| *TRBV20-1* | 2.532858176 | 9.208132143 | 1.86214224 | 2.53E-25 | 2.43E-23 |
| *TRAV16* | 0.417917296 | 1.702164286 | 2.026080915 | 2.80E-25 | 2.67E-23 |
| *AC006033.2* | 0.269093396 | 0.808955952 | 1.587954164 | 2.91E-25 | 2.76E-23 |
| *NCF1B* | 1.099038994 | 3.828510714 | 1.800540721 | 3.10E-25 | 2.94E-23 |
| *TRBV7-9* | 1.796018868 | 9.718208333 | 2.435887854 | 3.14E-25 | 2.96E-23 |
| *CLEC4E* | 1.437292453 | 6.484721429 | 2.173690957 | 3.19E-25 | 2.99E-23 |
| *SAMSN1* | 3.474343082 | 10.23745952 | 1.55904562 | 3.33E-25 | 3.12E-23 |
| *AC243960.1* | 1.121909748 | 2.822109524 | 1.330817355 | 3.37E-25 | 3.14E-23 |
| *LILRB1* | 2.506406604 | 8.12015 | 1.695885902 | 3.54E-25 | 3.29E-23 |
| *IL4I1* | 9.678221698 | 32.14847262 | 1.731936304 | 4.11E-25 | 3.79E-23 |
| *FYB1* | 5.988233962 | 22.58174881 | 1.914954724 | 4.30E-25 | 3.94E-23 |
| *ABI3* | 9.825262579 | 26.72674643 | 1.443716353 | 4.44E-25 | 4.06E-23 |
| *DOCK2* | 2.474818239 | 7.057438095 | 1.511821998 | 4.64E-25 | 4.23E-23 |
| *IFIH1* | 13.71925566 | 41.51082143 | 1.597285271 | 5.07E-25 | 4.60E-23 |
| *ZNF831* | 0.222969811 | 0.718767857 | 1.688677502 | 5.36E-25 | 4.84E-23 |
| *PCED1B-AS1* | 2.785365723 | 6.66347381 | 1.258407712 | 5.48E-25 | 4.93E-23 |
| *STAT4* | 1.160608176 | 3.0477 | 1.392839901 | 6.60E-25 | 5.93E-23 |
| *C2* | 7.330123899 | 25.23520595 | 1.783528372 | 7.45E-25 | 6.66E-23 |

| *SSTR3* | 0.087846226 | 0.358197619 | 2.027703528 | 7.51E-25 | 6.69E-23 |
| --- | --- | --- | --- | --- | --- |
| *LAT2* | 5.343805031 | 11.49984643 | 1.105675318 | 7.70E-25 | 6.83E-23 |
| *FPR3* | 12.83892233 | 39.9723869 | 1.638479614 | 8.22E-25 | 7.27E-23 |
| *IFI30* | 0.399098742 | 1.100684524 | 1.463583389 | 9.22E-25 | 8.13E-23 |
| *CMKLR1* | 4.416606289 | 13.87477857 | 1.651454611 | 9.38E-25 | 8.24E-23 |
| *HLA-DPA1* | 71.84668931 | 249.7147429 | 1.797287415 | 9.48E-25 | 8.30E-23 |
| *CD38* | 2.382961635 | 8.308939286 | 1.80190859 | 9.69E-25 | 8.43E-23 |
| *TRAV13-1* | 0.95285 | 3.801521429 | 1.996255899 | 9.70E-25 | 8.43E-23 |
| *LILRA6* | 1.553043082 | 5.865569048 | 1.917173227 | 1.13E-24 | 9.74E-23 |
| *BTN3A3* | 11.14333019 | 27.60688452 | 1.30884764 | 1.15E-24 | 9.92E-23 |
| *IFIT3* | 48.48894245 | 168.4211333 | 1.796345484 | 1.55E-24 | 1.33E-22 |
| *TRBV27* | 0.931763208 | 4.898838095 | 2.394404343 | 1.69E-24 | 1.44E-22 |
| *TRAV8-2* | 0.511649686 | 1.903438095 | 1.895379376 | 1.83E-24 | 1.56E-22 |
| *SEPTIN1* | 3.722897799 | 8.346290476 | 1.16470912 | 1.95E-24 | 1.65E-22 |
| *PARVG* | 2.470519497 | 6.399165476 | 1.373069332 | 1.96E-24 | 1.65E-22 |
| *EPSTI1* | 17.78349182 | 56.06863214 | 1.656655246 | 2.10E-24 | 1.77E-22 |
| *CD84* | 1.985213836 | 5.863008333 | 1.562346692 | 2.12E-24 | 1.78E-22 |
| *AC015911.7* | 0.60873522 | 2.257903571 | 1.891097129 | 2.23E-24 | 1.87E-22 |
| *GVINP1* | 0.724423585 | 2.036811905 | 1.491407335 | 2.29E-24 | 1.91E-22 |
| *MS4A6A* | 10.83123459 | 33.49571548 | 1.628778872 | 2.34E-24 | 1.95E-22 |
| *TRBV6-5* | 1.111786792 | 4.341486905 | 1.965309084 | 2.44E-24 | 2.02E-22 |
| *TNFAIP8L2* | 6.329857547 | 17.68862381 | 1.482576872 | 2.45E-24 | 2.02E-22 |
| *SLC15A3* | 10.62533113 | 34.06972619 | 1.68098255 | 2.47E-24 | 2.03E-22 |
| *FAM78A* | 3.219582704 | 7.417270238 | 1.204014622 | 2.70E-24 | 2.21E-22 |
| *LRRC25* | 4.78819434 | 14.56329405 | 1.6047831 | 2.82E-24 | 2.30E-22 |
| *SECTM1* | 25.96213428 | 88.6644369 | 1.771946571 | 2.97E-24 | 2.42E-22 |
| *SCIMP* | 1.114725472 | 3.498752381 | 1.650152108 | 3.14E-24 | 2.55E-22 |
| *TRGV9* | 0.080303145 | 0.40997381 | 2.352003359 | 3.15E-24 | 2.55E-22 |
| *PRKAR1B-AS1* | 2.73858522 | 7.831252381 | 1.515812268 | 3.46E-24 | 2.79E-22 |
| *TRAV21* | 0.811175472 | 3.811988095 | 2.23245768 | 3.61E-24 | 2.90E-22 |
| *HCK* | 11.08487013 | 36.73016071 | 1.728373341 | 3.77E-24 | 3.02E-22 |
| *PARP12* | 31.20363459 | 65.88157143 | 1.078160883 | 3.81E-24 | 3.04E-22 |
| *C3AR1* | 6.304075157 | 20.86529762 | 1.726748859 | 3.98E-24 | 3.16E-22 |
| *AL590764.1* | 0.841304717 | 2.11104881 | 1.327259597 | 4.05E-24 | 3.20E-22 |
| *AL162414.1* | 0.095060063 | 0.619222619 | 2.70354691 | 4.12E-24 | 3.25E-22 |
| *TRBV19* | 1.091419182 | 4.313758333 | 1.98274005 | 4.62E-24 | 3.63E-22 |
| *PATL2* | 0.721489623 | 1.801982143 | 1.320534165 | 4.86E-24 | 3.81E-22 |
| *CCL18* | 31.19109277 | 176.368525 | 2.499387115 | 5.16E-24 | 4.03E-22 |
| *TRAV12-3* | 0.650353145 | 2.846811905 | 2.130051948 | 5.29E-24 | 4.11E-22 |
| *DTHD1* | 0.172275157 | 0.492455952 | 1.515280015 | 5.71E-24 | 4.43E-22 |
| *GPR84* | 1.940063522 | 6.670236905 | 1.781634112 | 6.47E-24 | 5.00E-22 |
| *GFI1* | 1.970941195 | 4.746988095 | 1.268127699 | 6.86E-24 | 5.29E-22 |
| *RGL4* | 0.271509748 | 0.767684524 | 1.499509566 | 6.93E-24 | 5.33E-22 |
| *FOXP3* | 3.187278302 | 8.430830952 | 1.403349836 | 7.68E-24 | 5.88E-22 |
| *TRDV1* | 0.287046855 | 3.883441667 | 3.75797764 | 8.74E-24 | 6.67E-22 |
| *KLHDC7B-DT* | 1.914342453 | 7.938136905 | 2.05195151 | 9.83E-24 | 7.48E-22 |

| *TRBD1* | 7.745116667 | 32.59320595 | 2.07321239 | 9.85E-24 | 7.48E-22 |
| --- | --- | --- | --- | --- | --- |
| *TRAF3IP3* | 1.541624214 | 3.741592857 | 1.279201443 | 1.00E-23 | 7.60E-22 |
| *TRAV8-3* | 0.784895912 | 3.100909524 | 1.982118182 | 1.12E-23 | 8.40E-22 |
| *LAIR1* | 4.914587736 | 15.39634405 | 1.64744551 | 1.12E-23 | 8.40E-22 |
| *LST1* | 7.610433019 | 21.73585119 | 1.514026146 | 1.13E-23 | 8.47E-22 |
| *HLA-DRB5* | 187.7360865 | 720.0430012 | 1.939377077 | 1.14E-23 | 8.53E-22 |
| *UBD* | 0.041007547 | 0.37497619 | 3.192837635 | 1.18E-23 | 8.77E-22 |
| *TRAV12-1* | 0.599348742 | 2.819778571 | 2.234114265 | 1.26E-23 | 9.36E-22 |
| *HLA-A* | 1130.699865 | 3131.424008 | 1.469602841 | 1.33E-23 | 9.82E-22 |
| *GIMAP4* | 18.81976792 | 49.02677857 | 1.381321131 | 1.38E-23 | 1.02E-21 |
| *AC007991.2* | 1.563699057 | 14.80315476 | 3.242869878 | 1.39E-23 | 1.03E-21 |
| *TAPBP* | 189.8784893 | 379.9911917 | 1.0008895 | 1.43E-23 | 1.05E-21 |
| *AC004921.1* | 0.587298113 | 1.537653571 | 1.388565597 | 1.55E-23 | 1.13E-21 |
| *LINC02084* | 0.748649686 | 2.765233333 | 1.885038518 | 1.61E-23 | 1.18E-21 |
| *SAMD9L* | 10.38173239 | 33.06952262 | 1.671455014 | 1.62E-23 | 1.18E-21 |
| *SLC7A7* | 5.953338679 | 17.37432738 | 1.545186252 | 1.64E-23 | 1.19E-21 |
| *CXorf21* | 1.548792767 | 4.146864286 | 1.420876712 | 2.01E-23 | 1.45E-21 |
| *CD37* | 9.087377358 | 21.54191548 | 1.245210644 | 2.10E-23 | 1.51E-21 |
| *TRGV4* | 0.185487107 | 0.934172619 | 2.33237025 | 2.16E-23 | 1.55E-21 |
| *TRAV3* | 0.448381761 | 2.096602381 | 2.225253782 | 2.85E-23 | 2.04E-21 |
| *FCGR2A* | 12.73512579 | 40.95517857 | 1.685232675 | 2.94E-23 | 2.10E-21 |
| *SH2D2A* | 9.418065409 | 22.81853452 | 1.276703493 | 3.14E-23 | 2.24E-21 |
| *TRBV5-6* | 0.548952201 | 2.816090476 | 2.358941246 | 3.43E-23 | 2.44E-21 |
| *TRAV14DV4* | 0.38932327 | 1.928589286 | 2.308505454 | 3.61E-23 | 2.56E-21 |
| *NCKAP1L* | 4.880223899 | 12.8219369 | 1.393594971 | 3.64E-23 | 2.57E-21 |
| *CLNK* | 0.178353774 | 0.473257143 | 1.40788254 | 3.67E-23 | 2.59E-21 |
| *TRAV12-2* | 0.827324528 | 4.104629762 | 2.310726836 | 4.18E-23 | 2.93E-21 |
| *LYZ* | 72.46127956 | 401.2734464 | 2.469303504 | 5.04E-23 | 3.52E-21 |
| *IRF4* | 1.379021384 | 3.99387619 | 1.534144782 | 5.18E-23 | 3.61E-21 |
| *AL354833.2* | 0.134391509 | 0.654894048 | 2.284819524 | 5.21E-23 | 3.62E-21 |
| *FCGR1CP* | 0.719207862 | 2.189177381 | 1.605908159 | 5.50E-23 | 3.81E-21 |
| *ABCD2* | 0.224576415 | 0.741163095 | 1.722584622 | 6.62E-23 | 4.57E-21 |
| *C1orf162* | 8.334322013 | 20.98509405 | 1.332228181 | 7.14E-23 | 4.92E-21 |
| *AC007728.2* | 0.272871698 | 0.70575 | 1.370934456 | 7.24E-23 | 4.98E-21 |
| *ITGAX* | 5.667986164 | 13.98949643 | 1.30343589 | 7.52E-23 | 5.15E-21 |
| *PIK3R5* | 1.903576101 | 4.875592857 | 1.35686541 | 7.60E-23 | 5.19E-21 |
| *CD300C* | 2.111027358 | 6.688363095 | 1.663707894 | 7.84E-23 | 5.34E-21 |
| *IRF1-AS1* | 0.997730189 | 2.091859524 | 1.06806434 | 7.97E-23 | 5.41E-21 |
| *PSME2P1* | 3.136525472 | 6.748752381 | 1.105453542 | 8.80E-23 | 5.96E-21 |
| *XCL2* | 1.624043711 | 5.742294048 | 1.822036746 | 9.46E-23 | 6.39E-21 |
| *RASSF4* | 6.630125472 | 17.96426071 | 1.438021487 | 1.03E-22 | 6.94E-21 |
| *TRAV36DV7* | 0.306727987 | 1.307030952 | 2.09126159 | 1.03E-22 | 6.95E-21 |
| *TIFAB* | 0.143138365 | 0.570535714 | 1.994906795 | 1.05E-22 | 7.01E-21 |
| *CD300A* | 6.925544025 | 19.50713214 | 1.494002385 | 1.06E-22 | 7.10E-21 |
| *CLEC6A* | 0.070799057 | 0.388932143 | 2.457716428 | 1.09E-22 | 7.29E-21 |
| *CLEC4D* | 0.223930818 | 0.832160714 | 1.893809094 | 1.14E-22 | 7.58E-21 |

| *PLA2G7* | 9.344933019 | 26.84984167 | 1.522657352 | 1.31E-22 | 8.68E-21 |
| --- | --- | --- | --- | --- | --- |
| *TRAV19* | 0.948992138 | 3.84082381 | 2.016947744 | 1.38E-22 | 9.13E-21 |
| *AL096794.1* | 0.093759119 | 0.230008333 | 1.294655205 | 1.41E-22 | 9.29E-21 |
| *RUNX3* | 5.79535 | 16.87609048 | 1.542013029 | 1.45E-22 | 9.56E-21 |
| *ZC3H12D* | 0.697640881 | 1.574441667 | 1.174283819 | 1.56E-22 | 1.03E-20 |
| *RTP5* | 0.081075157 | 0.315477381 | 1.960204749 | 1.62E-22 | 1.06E-20 |
| *TRAV38-2DV8* | 0.361646855 | 1.95814881 | 2.436836895 | 1.72E-22 | 1.12E-20 |
| *ARHGAP25* | 4.642077358 | 9.920564286 | 1.095651619 | 1.97E-22 | 1.28E-20 |
| *AC096734.1* | 0.05192327 | 0.318503571 | 2.616856389 | 2.03E-22 | 1.31E-20 |
| *TRAV26-1* | 0.350766038 | 1.274595238 | 1.861458203 | 2.85E-22 | 1.84E-20 |
| *LINC01281* | 0.131661635 | 0.479816667 | 1.86564825 | 2.92E-22 | 1.88E-20 |
| *STX11* | 2.512491509 | 6.540960714 | 1.380383829 | 2.98E-22 | 1.92E-20 |
| *CD4* | 20.58693585 | 55.06627857 | 1.419439997 | 3.04E-22 | 1.95E-20 |
| *SIGLEC7* | 0.999883648 | 3.056272619 | 1.611941108 | 3.11E-22 | 1.99E-20 |
| *SIGLEC9* | 2.010782075 | 5.226117857 | 1.377982926 | 3.27E-22 | 2.09E-20 |
| *SRGN* | 148.1568072 | 430.5996833 | 1.539222344 | 3.55E-22 | 2.26E-20 |
| *TMC8* | 7.116906289 | 16.1027 | 1.177980466 | 3.74E-22 | 2.38E-20 |
| *MPEG1* | 10.6086783 | 28.24914286 | 1.412962166 | 3.82E-22 | 2.42E-20 |
| *TRAV8-6* | 0.618466981 | 2.882664286 | 2.220634351 | 4.24E-22 | 2.68E-20 |
| *TRAV2* | 0.672784277 | 2.227133333 | 1.726972037 | 4.44E-22 | 2.80E-20 |
| *TRIM22* | 21.8765827 | 62.006575 | 1.503033808 | 4.45E-22 | 2.80E-20 |
| *TSPAN32* | 0.505753774 | 1.41792381 | 1.487272929 | 4.57E-22 | 2.87E-20 |
| *SUSD3* | 7.896397484 | 17.73944405 | 1.167694278 | 4.99E-22 | 3.12E-20 |
| *LINC02345* | 0.393008491 | 1.512575 | 1.944374293 | 5.00E-22 | 3.12E-20 |
| *TRBV7-6* | 0.351136792 | 1.823534524 | 2.376632437 | 5.16E-22 | 3.22E-20 |
| *HLA-DMA* | 63.86405 | 158.2911131 | 1.30950431 | 5.36E-22 | 3.33E-20 |
| *IFITM1* | 131.2570535 | 438.5069202 | 1.740204658 | 5.70E-22 | 3.52E-20 |
| *CD14* | 101.4873415 | 292.392494 | 1.526606485 | 6.42E-22 | 3.96E-20 |
| *IRF8* | 6.158075786 | 13.65331905 | 1.148700178 | 6.58E-22 | 4.05E-20 |
| *ERFL* | 0.327821069 | 0.861995238 | 1.39477132 | 6.74E-22 | 4.13E-20 |
| *HNRNPA1P21* | 2.619725157 | 6.875841667 | 1.392120861 | 7.15E-22 | 4.37E-20 |
| *S1PR4* | 4.380076415 | 10.55113095 | 1.268369702 | 7.22E-22 | 4.41E-20 |
| *MS4A4A* | 8.342006604 | 24.37084524 | 1.546689929 | 7.92E-22 | 4.82E-20 |
| *TRBV15* | 0.238228931 | 1.38334881 | 2.537744444 | 8.12E-22 | 4.93E-20 |
| *TNFRSF1B* | 31.65874623 | 71.64959524 | 1.178354433 | 8.33E-22 | 5.05E-20 |
| *CCR1* | 8.10094717 | 21.54544881 | 1.411220647 | 8.77E-22 | 5.30E-20 |
| *NCF2* | 11.6274566 | 33.09733452 | 1.50917948 | 8.91E-22 | 5.37E-20 |
| *TRBV7-2* | 2.078489308 | 8.393771429 | 2.013783851 | 9.45E-22 | 5.68E-20 |
| *TRAV24* | 0.234813522 | 1.276920238 | 2.443081016 | 9.47E-22 | 5.68E-20 |
| *EML4-AS1* | 0.240577987 | 0.851997619 | 1.824344755 | 9.92E-22 | 5.93E-20 |
| *UNQ6494* | 0.070863208 | 0.293715476 | 2.051310611 | 1.29E-21 | 7.67E-20 |
| *ICAM3* | 0.751498428 | 1.883014286 | 1.325201954 | 1.35E-21 | 8.01E-20 |
| *AC006369.1* | 0.111097484 | 0.412758333 | 1.893471194 | 1.39E-21 | 8.26E-20 |
| *FGL2* | 7.375707547 | 19.96119048 | 1.436344407 | 1.40E-21 | 8.31E-20 |
| *LAX1* | 0.84298522 | 2.750734524 | 1.706237668 | 1.63E-21 | 9.64E-20 |
| *LILRB5* | 1.675033019 | 4.090808333 | 1.288196409 | 1.63E-21 | 9.64E-20 |

| *SMTNL1* | 0.966807862 | 4.008241667 | 2.051668385 | 1.74E-21 | 1.02E-19 |
| --- | --- | --- | --- | --- | --- |
| *RAC2* | 53.55005314 | 114.9415619 | 1.101940647 | 2.00E-21 | 1.18E-19 |
| *TRBV18* | 0.756834591 | 2.692564286 | 1.830930856 | 2.01E-21 | 1.18E-19 |
| *TRAV9-2* | 0.628445912 | 1.901183333 | 1.597037172 | 2.34E-21 | 1.37E-19 |
| *CD72* | 3.582090566 | 8.49114881 | 1.245157943 | 2.42E-21 | 1.41E-19 |
| *TRAV10* | 0.181670755 | 0.753859524 | 2.052969519 | 2.55E-21 | 1.48E-19 |
| *TRGV3* | 0.187122956 | 1.003635714 | 2.423177253 | 2.80E-21 | 1.62E-19 |
| *LY9* | 0.511630503 | 1.299472619 | 1.344752051 | 2.91E-21 | 1.68E-19 |
| *BTK* | 2.78222673 | 6.260583333 | 1.170057094 | 2.91E-21 | 1.68E-19 |
| *RNASE6* | 24.76400094 | 63.92744286 | 1.368190967 | 2.94E-21 | 1.69E-19 |
| *OSCAR* | 5.167164465 | 15.12585476 | 1.549571962 | 3.06E-21 | 1.76E-19 |
| *CD209* | 3.939135849 | 9.846583333 | 1.321744038 | 3.46E-21 | 1.98E-19 |
| *DDX58* | 17.7904478 | 44.6350619 | 1.327074602 | 3.56E-21 | 2.03E-19 |
| *LINC01094* | 0.874761321 | 2.471963095 | 1.49869587 | 3.64E-21 | 2.07E-19 |
| *CD163* | 15.7236761 | 51.64906905 | 1.715803795 | 3.86E-21 | 2.19E-19 |
| *TNFSF14* | 0.638973899 | 1.716964286 | 1.426031124 | 4.02E-21 | 2.27E-19 |
| *U62317.3* | 0.22822044 | 0.713461905 | 1.644408389 | 4.13E-21 | 2.33E-19 |
| *TLDC2* | 0.354727358 | 0.782444048 | 1.141276986 | 4.31E-21 | 2.42E-19 |
| *C19orf38* | 2.983569182 | 7.010925 | 1.232565571 | 4.31E-21 | 2.42E-19 |
| *AL353807.5* | 3.238780818 | 9.733086905 | 1.587446599 | 4.36E-21 | 2.44E-19 |
| *HCP5* | 10.40187673 | 29.39215714 | 1.498587399 | 4.45E-21 | 2.48E-19 |
| *AC007991.4* | 0.214934591 | 1.35710119 | 2.658558706 | 4.73E-21 | 2.63E-19 |
| *MCOLN2* | 1.927389623 | 4.769364286 | 1.307148738 | 5.44E-21 | 3.00E-19 |
| *TRAV1-2* | 0.394516981 | 1.95452381 | 2.308657855 | 5.81E-21 | 3.20E-19 |
| *TRBV11-2* | 0.477827673 | 2.094397619 | 2.131973049 | 6.41E-21 | 3.53E-19 |
| *EVI2A* | 2.737043711 | 6.158947619 | 1.170065384 | 6.58E-21 | 3.61E-19 |
| *KIR2DL3* | 0.047214151 | 0.710810714 | 3.912174196 | 6.97E-21 | 3.81E-19 |
| *TTC16* | 0.073140566 | 0.28575119 | 1.966015812 | 7.21E-21 | 3.93E-19 |
| *AL596214.1* | 0.214150943 | 0.562794048 | 1.393979037 | 7.49E-21 | 4.07E-19 |
| *KIR2DL1* | 0.035453459 | 0.545795238 | 3.944361507 | 7.50E-21 | 4.07E-19 |
| *AP003774.2* | 0.547493711 | 1.399384524 | 1.353878145 | 7.58E-21 | 4.11E-19 |
| *HLA-DQB1-AS1* | 1.856955975 | 5.989440476 | 1.689481623 | 7.98E-21 | 4.31E-19 |
| *TRBJ1-1* | 0.635164151 | 2.649353571 | 2.060438999 | 8.18E-21 | 4.41E-19 |
| *ODF3B* | 20.68701101 | 46.43192976 | 1.166392032 | 8.45E-21 | 4.54E-19 |
| *LAPTM5* | 61.83986352 | 150.567194 | 1.283798426 | 8.88E-21 | 4.77E-19 |
| *CCL7* | 0.982202516 | 7.91742381 | 3.010938656 | 8.93E-21 | 4.78E-19 |
| *TRIM21* | 33.85918333 | 69.396 | 1.035305331 | 9.42E-21 | 5.04E-19 |
| *BTLA* | 0.545116981 | 1.216560714 | 1.158170554 | 9.89E-21 | 5.27E-19 |
| *CARD16* | 9.565851572 | 21.91678452 | 1.196070841 | 9.91E-21 | 5.27E-19 |
| *JAML* | 2.677875157 | 6.284433333 | 1.230693959 | 1.08E-20 | 5.76E-19 |
| *BTN3A1* | 17.86515031 | 37.06600119 | 1.052948428 | 1.30E-20 | 6.87E-19 |
| *ALOX5AP* | 18.09378931 | 49.20960833 | 1.443445457 | 1.55E-20 | 8.20E-19 |
| *RSAD2* | 14.14191258 | 42.63655476 | 1.59211362 | 1.58E-20 | 8.34E-19 |
| *IL12RB2* | 2.182857862 | 8.093913095 | 1.890619168 | 1.62E-20 | 8.53E-19 |
| *TRAV6* | 0.215158491 | 1.071005952 | 2.31549482 | 1.63E-20 | 8.58E-19 |
| *KLRB1* | 2.157143711 | 5.449133333 | 1.336904499 | 1.65E-20 | 8.67E-19 |

| *GAB3* | 1.328131447 | 3.2221 | 1.278603331 | 1.68E-20 | 8.78E-19 |
| --- | --- | --- | --- | --- | --- |
| *ITGB2-AS1* | 1.690142453 | 3.867305952 | 1.194184056 | 1.71E-20 | 8.94E-19 |
| *CD33* | 0.900969811 | 2.05674881 | 1.190814937 | 1.73E-20 | 9.01E-19 |
| *TRBV12-4* | 0.534127987 | 1.932010714 | 1.854845709 | 1.80E-20 | 9.38E-19 |
| *ICAM1* | 34.57803616 | 106.2172262 | 1.61908992 | 1.95E-20 | 1.01E-18 |
| *P2RY13* | 1.587630189 | 5.689482143 | 1.841422444 | 2.13E-20 | 1.10E-18 |
| *PARP14* | 33.11626101 | 67.26040119 | 1.02221759 | 2.33E-20 | 1.20E-18 |
| *CEACAM4* | 0.936083019 | 3.373689286 | 1.849618719 | 2.37E-20 | 1.22E-18 |
| *HLA-DOA* | 17.31436447 | 49.45074405 | 1.514022795 | 2.49E-20 | 1.28E-18 |
| *PRAM1* | 1.337507233 | 3.426332143 | 1.357118318 | 2.54E-20 | 1.30E-18 |
| *TRBV29-1* | 1.340153145 | 5.367042857 | 2.001729536 | 2.59E-20 | 1.32E-18 |
| *TRAV8-4* | 0.551557862 | 2.009008333 | 1.864899402 | 2.90E-20 | 1.48E-18 |
| *LILRA5* | 3.061933019 | 10.43486905 | 1.768897867 | 3.59E-20 | 1.82E-18 |
| *APOL6* | 25.20157704 | 54.6737631 | 1.117334661 | 3.62E-20 | 1.84E-18 |
| *JAK3* | 7.452986164 | 16.9655881 | 1.186720954 | 3.66E-20 | 1.85E-18 |
| *TRBV10-3* | 0.70884434 | 2.210959524 | 1.641131859 | 3.74E-20 | 1.89E-18 |
| *TRBV4-2* | 0.769089308 | 3.467209524 | 2.17255198 | 3.75E-20 | 1.89E-18 |
| *PIK3CD* | 7.065598742 | 17.69973333 | 1.324843899 | 3.80E-20 | 1.91E-18 |
| *HLA-DRB6* | 40.91220597 | 142.3859679 | 1.799203742 | 4.04E-20 | 2.03E-18 |
| *TRBV10-2* | 0.140553774 | 1.293085714 | 3.201623817 | 4.06E-20 | 2.03E-18 |
| *ADAMDEC1* | 4.660700943 | 21.44046667 | 2.201717458 | 4.09E-20 | 2.05E-18 |
| *HLA-DOB* | 3.030570126 | 6.270144048 | 1.048909361 | 4.20E-20 | 2.09E-18 |
| *GIMAP7* | 13.66193868 | 32.53858333 | 1.251989218 | 4.24E-20 | 2.11E-18 |
| *VSIG4* | 17.86666321 | 56.9631119 | 1.672757742 | 4.72E-20 | 2.34E-18 |
| *NCR3* | 0.848103145 | 2.143139286 | 1.337413978 | 4.84E-20 | 2.39E-18 |
| *TRBV5-4* | 0.477240881 | 1.753641667 | 1.877564448 | 5.05E-20 | 2.50E-18 |
| *DOCK10* | 2.20647673 | 4.870082143 | 1.142201574 | 5.21E-20 | 2.57E-18 |
| *IFIT2* | 11.75486792 | 36.42047262 | 1.631491315 | 5.36E-20 | 2.64E-18 |
| *AC004687.1* | 2.421072327 | 5.481714286 | 1.178980956 | 5.63E-20 | 2.76E-18 |
| *PTAFR* | 12.36341635 | 31.78898333 | 1.362449423 | 7.33E-20 | 3.59E-18 |
| *SP110* | 9.09943239 | 19.17382738 | 1.075289889 | 8.08E-20 | 3.95E-18 |
| *MEFV* | 0.730861321 | 1.942065476 | 1.409922252 | 8.52E-20 | 4.15E-18 |
| *SPOCK2* | 11.75511164 | 37.60247143 | 1.677539246 | 9.08E-20 | 4.41E-18 |
| *AC093063.1* | 0.078608805 | 1.127263095 | 3.841989541 | 9.20E-20 | 4.46E-18 |
| *MATK* | 2.132814465 | 4.351692857 | 1.028818264 | 9.34E-20 | 4.52E-18 |
| *TRGV8* | 0.155954717 | 0.683794048 | 2.132434675 | 9.96E-20 | 4.81E-18 |
| *TRAV39* | 0.349751572 | 1.450742857 | 2.052389377 | 1.00E-19 | 4.83E-18 |
| *AC004585.1* | 1.463896541 | 3.937916667 | 1.427618985 | 1.11E-19 | 5.36E-18 |
| *SYTL3* | 4.669572642 | 12.10597262 | 1.374356567 | 1.23E-19 | 5.92E-18 |
| *BFSP2* | 0.178660692 | 0.408088095 | 1.191658371 | 1.26E-19 | 6.02E-18 |
| *TRBC2* | 89.75126478 | 208.8549119 | 1.218496899 | 1.29E-19 | 6.13E-18 |
| *CCR2* | 1.246003459 | 3.849732143 | 1.627449996 | 1.32E-19 | 6.29E-18 |
| *TRAV22* | 0.256147484 | 1.129966667 | 2.141233587 | 1.45E-19 | 6.89E-18 |
| *CTSS* | 72.33714025 | 157.8830821 | 1.126048119 | 1.56E-19 | 7.37E-18 |
| *PSMB10* | 41.43620503 | 82.90624524 | 1.000588904 | 1.56E-19 | 7.37E-18 |
| *PIK3R6* | 1.237881761 | 2.770521429 | 1.162284007 | 1.59E-19 | 7.50E-18 |

| *KIR3DL1* | 0.047672327 | 0.64025 | 3.747411393 | 1.68E-19 | 7.90E-18 |
| --- | --- | --- | --- | --- | --- |
| *CSF1R* | 19.9733544 | 50.7663869 | 1.345796941 | 1.72E-19 | 8.07E-18 |
| *TRAV5* | 0.354173585 | 1.546420238 | 2.126403902 | 1.79E-19 | 8.35E-18 |
| *AC008105.3* | 1.683890252 | 4.562269048 | 1.437953415 | 1.92E-19 | 8.97E-18 |
| *SERPING1* | 78.43888396 | 219.1020762 | 1.481962241 | 1.97E-19 | 9.17E-18 |
| *CMPK2* | 11.53731824 | 28.32425119 | 1.295729896 | 2.15E-19 | 9.98E-18 |
| *LAMP3* | 15.08577107 | 40.59078214 | 1.427963702 | 2.50E-19 | 1.16E-17 |
| *CD70* | 3.123092453 | 11.7344369 | 1.90970143 | 2.55E-19 | 1.18E-17 |
| *ZBED2* | 7.355366981 | 21.93932976 | 1.576650224 | 2.61E-19 | 1.21E-17 |
| *TRBJ1-6* | 2.152359119 | 6.759502381 | 1.650998232 | 2.62E-19 | 1.21E-17 |
| *SERPINA1* | 38.05871289 | 117.5528929 | 1.627011366 | 2.84E-19 | 1.31E-17 |
| *PTGDR* | 0.271855975 | 0.714763095 | 1.394622611 | 2.99E-19 | 1.37E-17 |
| *AL096816.1* | 0.105230189 | 0.426630952 | 2.01943999 | 3.01E-19 | 1.38E-17 |
| *MPP1* | 6.911673585 | 14.8903119 | 1.107266984 | 3.27E-19 | 1.50E-17 |
| *PIM2* | 24.45592987 | 52.23232262 | 1.094758537 | 3.40E-19 | 1.56E-17 |
| *ITGAM* | 4.248912893 | 15.42427738 | 1.860037229 | 3.47E-19 | 1.58E-17 |
| *TRAV25* | 0.238759434 | 1.012092857 | 2.083712016 | 3.48E-19 | 1.58E-17 |
| *CCL13* | 11.52675629 | 56.69719524 | 2.298290783 | 3.60E-19 | 1.64E-17 |
| *KIR2DS4* | 0.0778 | 2.442759524 | 4.97259788 | 3.80E-19 | 1.73E-17 |
| *PELATON* | 1.133103774 | 2.906541667 | 1.359023596 | 4.08E-19 | 1.85E-17 |
| *HLA-H* | 91.85146226 | 234.4962179 | 1.35219006 | 4.28E-19 | 1.93E-17 |
| *MSR1* | 5.145450943 | 13.59479643 | 1.401685124 | 4.45E-19 | 2.01E-17 |
| *RGS18* | 0.848612264 | 2.062082143 | 1.28092437 | 4.80E-19 | 2.16E-17 |
| *AL139125.1* | 0.359903145 | 0.87394881 | 1.27994007 | 4.90E-19 | 2.20E-17 |
| *HEATR9* | 0.123687421 | 0.281302381 | 1.185422973 | 5.42E-19 | 2.43E-17 |
| *IFI44L* | 12.02838836 | 29.33162976 | 1.28601388 | 5.64E-19 | 2.53E-17 |
| *GPRIN3* | 1.377407862 | 2.807897619 | 1.027534517 | 5.86E-19 | 2.62E-17 |
| *MMP25* | 1.943139937 | 4.17754881 | 1.104266883 | 5.97E-19 | 2.67E-17 |
| *TRBJ1-5* | 1.807381132 | 5.957971429 | 1.720920438 | 6.22E-19 | 2.77E-17 |
| *TRAV20* | 0.252506289 | 1.150272619 | 2.187584599 | 6.51E-19 | 2.89E-17 |
| *SNX10* | 10.1737456 | 22.99986786 | 1.176774648 | 7.02E-19 | 3.10E-17 |
| *TESPA1* | 1.057665094 | 2.168809524 | 1.036020478 | 7.09E-19 | 3.13E-17 |
| *SH2D1B* | 0.315359119 | 0.862516667 | 1.451556681 | 7.35E-19 | 3.24E-17 |
| *CLEC7A* | 7.42871761 | 19.18096071 | 1.368489892 | 7.43E-19 | 3.26E-17 |
| *CCL3L3* | 2.064747799 | 7.408566667 | 1.843228879 | 8.09E-19 | 3.54E-17 |
| *CASP4LP* | 0.803646855 | 1.815563095 | 1.175783483 | 8.40E-19 | 3.68E-17 |
| *AC023825.2* | 0.315122956 | 0.693086905 | 1.137121405 | 8.78E-19 | 3.84E-17 |
| *GPR141* | 0.331492453 | 0.915857143 | 1.466146557 | 9.11E-19 | 3.97E-17 |
| *GLIPR2* | 16.89831698 | 40.88584048 | 1.274721732 | 9.15E-19 | 3.98E-17 |
| *ADAP2* | 6.393964465 | 12.92953929 | 1.015888238 | 9.41E-19 | 4.09E-17 |
| *TRBV3-1* | 0.688447799 | 2.41050119 | 1.80791397 | 1.10E-18 | 4.77E-17 |
| *WIPF1* | 10.91470786 | 22.71729048 | 1.057517257 | 1.13E-18 | 4.88E-17 |
| *NLRP3* | 1.422112579 | 4.076580952 | 1.519323987 | 1.15E-18 | 4.96E-17 |
| *GRAMD1B* | 0.682565723 | 2.059353571 | 1.593151675 | 1.21E-18 | 5.22E-17 |
| *VAV1* | 5.823169182 | 12.5388869 | 1.106532844 | 1.23E-18 | 5.28E-17 |
| *CSF1* | 22.25709937 | 50.74430595 | 1.188980358 | 1.31E-18 | 5.63E-17 |

| *ITGB7* | 1.663383648 | 3.854739286 | 1.212512333 | 1.36E-18 | 5.84E-17 |
| --- | --- | --- | --- | --- | --- |
| *E2F3P1* | 0.086209434 | 0.302615476 | 1.811568113 | 1.41E-18 | 6.06E-17 |
| *SLCO2B1* | 6.54920566 | 17.46964643 | 1.415458569 | 1.44E-18 | 6.15E-17 |
| *THEMIS2* | 12.15381761 | 28.09766786 | 1.209040843 | 1.82E-18 | 7.75E-17 |
| *TRBV6-2* | 0.670112579 | 2.597096429 | 1.954424188 | 1.94E-18 | 8.24E-17 |
| *RASGRP1* | 1.834741195 | 4.334441667 | 1.240269594 | 1.95E-18 | 8.29E-17 |
| *PPP1R16B* | 2.808788365 | 5.797403571 | 1.045458994 | 2.34E-18 | 9.88E-17 |
| *ACY3* | 2.108786478 | 5.314270238 | 1.333458568 | 2.35E-18 | 9.91E-17 |
| *TRBV24-1* | 0.337863208 | 1.702409524 | 2.333066969 | 2.63E-18 | 1.11E-16 |
| *TRBV6-6* | 0.544917925 | 2.518570238 | 2.208494113 | 2.71E-18 | 1.14E-16 |
| *ZMYND15* | 2.338732075 | 4.771444048 | 1.028699359 | 2.88E-18 | 1.21E-16 |
| *RUNX3-AS1* | 0.241451887 | 0.547671429 | 1.181574881 | 2.99E-18 | 1.25E-16 |
| *FCGR2C* | 1.358489308 | 3.242546429 | 1.255124021 | 3.25E-18 | 1.36E-16 |
| *AC093583.1* | 0.07645566 | 0.195188095 | 1.352169844 | 3.30E-18 | 1.37E-16 |
| *SCO2* | 0.893741195 | 2.857311905 | 1.676729501 | 3.37E-18 | 1.40E-16 |
| *PLAT4* | 107.7991497 | 365.6661345 | 1.762181222 | 3.53E-18 | 1.47E-16 |
| *TRAV26-2* | 0.295552516 | 1.19524881 | 2.015824563 | 4.01E-18 | 1.66E-16 |
| *TREM2* | 19.32981541 | 45.93899167 | 1.24889133 | 4.06E-18 | 1.68E-16 |
| *TRBV21-1* | 0.145710377 | 0.747130952 | 2.358257503 | 4.41E-18 | 1.82E-16 |
| *FPR2* | 0.726598428 | 2.620490476 | 1.850606717 | 4.77E-18 | 1.97E-16 |
| *IGHG1* | 1413.466631 | 5505.0988 | 1.961530632 | 5.16E-18 | 2.12E-16 |
| *KCNA3* | 0.382043711 | 1.04032619 | 1.445226335 | 5.17E-18 | 2.12E-16 |
| *AC096667.1* | 0.091462893 | 0.228047619 | 1.318076649 | 5.88E-18 | 2.40E-16 |
| *IGFLR1* | 1.10263805 | 2.291741667 | 1.055485135 | 6.27E-18 | 2.54E-16 |
| *PGBD4P1* | 0.108977673 | 0.503241667 | 2.207218787 | 6.39E-18 | 2.59E-16 |
| *TRBJ1-4* | 1.400304088 | 4.305702381 | 1.620508446 | 7.51E-18 | 3.03E-16 |
| *TRBV28* | 1.462334277 | 7.240809524 | 2.307877864 | 7.97E-18 | 3.21E-16 |
| *OASL* | 19.05891509 | 44.24811786 | 1.215150092 | 8.26E-18 | 3.32E-16 |
| *C9orf139* | 0.306412264 | 0.70915 | 1.210616778 | 8.38E-18 | 3.36E-16 |
| *TRAV29DV5* | 0.714071384 | 2.318835714 | 1.699260402 | 1.00E-17 | 4.01E-16 |
| *AC021188.1* | 0.215949686 | 0.541365476 | 1.325907672 | 1.02E-17 | 4.09E-16 |
| *AL157871.2* | 0.719855031 | 2.82742619 | 1.973711061 | 1.07E-17 | 4.25E-16 |
| *TFAP2E-AS1* | 0.631309748 | 1.376832143 | 1.124932749 | 1.10E-17 | 4.37E-16 |
| *CCL1* | 0.106060063 | 0.513278571 | 2.274860522 | 1.26E-17 | 4.99E-16 |
| *AC090559.1* | 1.432813836 | 3.347530952 | 1.224246221 | 1.36E-17 | 5.37E-16 |
| *AC092580.4* | 0.138369497 | 0.277466667 | 1.003788524 | 1.41E-17 | 5.57E-16 |
| *FCRL3* | 0.612487421 | 1.448717857 | 1.242024531 | 1.50E-17 | 5.91E-16 |
| *BATF3* | 2.06215283 | 4.704407143 | 1.189861666 | 1.57E-17 | 6.18E-16 |
| *IL7R* | 7.837594025 | 25.815675 | 1.71976457 | 1.61E-17 | 6.34E-16 |
| *LY86* | 8.733928931 | 17.75085833 | 1.02318609 | 1.88E-17 | 7.38E-16 |
| *TRBV7-3* | 0.598474843 | 1.888215476 | 1.6576609 | 1.91E-17 | 7.47E-16 |
| *C5AR1* | 10.95685912 | 28.95162024 | 1.401809793 | 2.22E-17 | 8.67E-16 |
| *CARD17* | 0.80572327 | 2.424079762 | 1.589080842 | 2.28E-17 | 8.90E-16 |
| *HSPA7* | 7.38447044 | 20.10692143 | 1.445125837 | 2.32E-17 | 9.03E-16 |
| *CCL25* | 0.13778522 | 0.806554762 | 2.549351348 | 2.33E-17 | 9.05E-16 |
| *GIMAP1* | 1.966972327 | 4.108140476 | 1.062508854 | 2.39E-17 | 9.29E-16 |

| *OAS2* | 49.55538239 | 106.6186202 | 1.10534575 | 2.43E-17 | 9.39E-16 |
| --- | --- | --- | --- | --- | --- |
| *PLA2G2D* | 3.000606604 | 10.69322262 | 1.833370613 | 2.70E-17 | 1.04E-15 |
| *TRAF1* | 8.349094969 | 17.54859048 | 1.071663431 | 2.71E-17 | 1.04E-15 |
| *LINC00861* | 0.284380818 | 0.707529762 | 1.314966682 | 2.84E-17 | 1.09E-15 |
| *TLR1* | 2.307246541 | 5.480921429 | 1.248246282 | 2.84E-17 | 1.09E-15 |
| *IGHG3* | 204.9840918 | 659.3993024 | 1.68564041 | 2.91E-17 | 1.11E-15 |
| *PIK3CD-AS1* | 0.094287736 | 0.202775 | 1.10473776 | 2.93E-17 | 1.12E-15 |
| *AL365361.1* | 0.690950629 | 1.798617857 | 1.380234164 | 3.02E-17 | 1.15E-15 |
| *RGS1* | 28.51301447 | 77.43945119 | 1.441448156 | 3.10E-17 | 1.18E-15 |
| *IGKV3-11* | 105.9781755 | 548.147325 | 2.370796502 | 3.13E-17 | 1.19E-15 |
| *TMEM176B* | 66.13463491 | 170.0133512 | 1.362170128 | 3.13E-17 | 1.19E-15 |
| *LTB* | 17.43906447 | 36.21909286 | 1.054427766 | 3.33E-17 | 1.26E-15 |
| *TBXAS1* | 4.389127987 | 9.545925 | 1.120950662 | 3.45E-17 | 1.31E-15 |
| *AC245884.12* | 1.723169182 | 11.48241786 | 2.736290204 | 4.03E-17 | 1.53E-15 |
| *TRGV5* | 0.134345283 | 0.736784524 | 2.45529709 | 4.27E-17 | 1.61E-15 |
| *LINC02384* | 0.235256289 | 0.763588095 | 1.698561318 | 4.34E-17 | 1.64E-15 |
| *LINC02100* | 1.211172956 | 3.442085714 | 1.506878126 | 4.45E-17 | 1.68E-15 |
| *IGHM* | 210.2754739 | 704.2543131 | 1.743815907 | 4.53E-17 | 1.70E-15 |
| *TRAV23DV6* | 0.327090881 | 1.001941667 | 1.615035075 | 4.57E-17 | 1.72E-15 |
| *PEAK3* | 0.427773899 | 0.937042857 | 1.131266574 | 4.67E-17 | 1.75E-15 |
| *SOCAR* | 0.317250314 | 0.805652381 | 1.344535892 | 4.84E-17 | 1.81E-15 |
| *WAKMAR2* | 0.806344654 | 1.802408333 | 1.160457365 | 5.20E-17 | 1.94E-15 |
| *TMEM106A* | 2.549592138 | 5.803511905 | 1.186659713 | 5.92E-17 | 2.20E-15 |
| *TRAV41* | 0.449830818 | 1.458315476 | 1.696848443 | 6.27E-17 | 2.32E-15 |
| *CASP1* | 18.29599497 | 46.60495714 | 1.348955541 | 7.41E-17 | 2.74E-15 |
| *IFI44* | 65.55587107 | 150.4131869 | 1.198134159 | 7.47E-17 | 2.76E-15 |
| *TRBV5-5* | 0.225610063 | 0.907617857 | 2.008253576 | 7.72E-17 | 2.85E-15 |
| *SERPINB9* | 17.57417956 | 37.31042262 | 1.086121362 | 8.54E-17 | 3.15E-15 |
| *TRAV38-1* | 0.158333019 | 0.869158333 | 2.456656867 | 8.96E-17 | 3.29E-15 |
| *IFITM3* | 904.7640336 | 2303.024015 | 1.34791597 | 9.17E-17 | 3.37E-15 |
| *AIM2* | 27.18532736 | 91.36844405 | 1.748867786 | 9.51E-17 | 3.48E-15 |
| *JAK2* | 6.000848113 | 12.40529881 | 1.047718167 | 9.51E-17 | 3.48E-15 |
| *Z95114.3* | 0.379061635 | 1.005357143 | 1.407203741 | 9.57E-17 | 3.50E-15 |
| *AC012236.1* | 1.184273585 | 3.43484881 | 1.536244194 | 9.78E-17 | 3.56E-15 |
| *DDX60* | 17.21367201 | 38.13504048 | 1.147562346 | 1.02E-16 | 3.71E-15 |
| *XIRP1* | 0.420007862 | 2.884008333 | 2.779587096 | 1.16E-16 | 4.22E-15 |
| *HMGB1P17* | 0.071146855 | 0.282138095 | 1.98752958 | 1.16E-16 | 4.22E-15 |
| *VIM-AS1* | 1.357177044 | 2.996267857 | 1.142557669 | 1.24E-16 | 4.47E-15 |
| *LILRA1* | 0.354289937 | 0.940419048 | 1.408373271 | 1.32E-16 | 4.77E-15 |
| *IFNL1* | 0.240135535 | 0.935433333 | 1.961785933 | 1.44E-16 | 5.20E-15 |
| *TRGV7* | 0.421376415 | 1.377097619 | 1.70844936 | 1.57E-16 | 5.67E-15 |
| *ARRDC5* | 0.313946855 | 0.666539286 | 1.086169548 | 1.59E-16 | 5.71E-15 |
| *TNFRSF8* | 1.007060692 | 2.017966667 | 1.002751712 | 1.60E-16 | 5.77E-15 |
| *LINC01127* | 0.29946195 | 3.323070238 | 3.472072179 | 1.62E-16 | 5.80E-15 |
| *MRC1* | 10.0958022 | 24.55485357 | 1.282252669 | 1.78E-16 | 6.39E-15 |
| *CACNA1D* | 1.815963836 | 0.491413095 | -1.885727264 | 2.07E-16 | 7.39E-15 |

| *ART3* | 0.312825786 | 1.025170238 | 1.712432157 | 2.07E-16 | 7.39E-15 |
| --- | --- | --- | --- | --- | --- |
| *MZB1* | 14.06290535 | 44.25220952 | 1.65385481 | 2.09E-16 | 7.43E-15 |
| *IL12B* | 0.114355031 | 0.367652381 | 1.684822484 | 2.34E-16 | 8.31E-15 |
| *LAIR2* | 1.065291824 | 3.246588095 | 1.607675663 | 2.55E-16 | 9.02E-15 |
| *MX1* | 44.54750943 | 95.70403929 | 1.103235041 | 2.72E-16 | 9.60E-15 |
| *LINC00528* | 0.513261321 | 1.08747619 | 1.083218366 | 3.11E-16 | 1.10E-14 |
| *LINC00944* | 0.10131195 | 0.319665476 | 1.657758593 | 3.46E-16 | 1.21E-14 |
| *VNN2* | 1.757768553 | 4.724407143 | 1.426388177 | 3.60E-16 | 1.26E-14 |
| *AL357054.4* | 0.716942453 | 1.685353571 | 1.23312206 | 3.63E-16 | 1.27E-14 |
| *UPK1A* | 393.9701623 | 125.5960452 | -1.649295332 | 3.93E-16 | 1.37E-14 |
| *C20orf141* | 0.116213836 | 1.055122619 | 3.182556917 | 4.13E-16 | 1.44E-14 |
| *VSIG2* | 300.2827723 | 70.1753131 | -2.097286207 | 4.14E-16 | 1.44E-14 |
| *RUFY4* | 0.735569497 | 1.604197619 | 1.124918317 | 4.24E-16 | 1.48E-14 |
| *FAS* | 6.469166038 | 14.88405595 | 1.202116072 | 4.47E-16 | 1.55E-14 |
| *ZNF80* | 0.068927987 | 0.271002381 | 1.97514373 | 4.83E-16 | 1.67E-14 |
| *BTN3A2* | 18.5253827 | 38.37422857 | 1.050634402 | 5.10E-16 | 1.76E-14 |
| *LINC01857* | 1.622875472 | 3.399395238 | 1.066725808 | 5.51E-16 | 1.89E-14 |
| *TNFSF8* | 1.08587044 | 2.270554762 | 1.064192853 | 5.58E-16 | 1.92E-14 |
| *APOE* | 209.1306255 | 695.9585679 | 1.734597073 | 5.96E-16 | 2.04E-14 |
| *TRAV13-2* | 0.721485849 | 2.027089286 | 1.490366632 | 6.02E-16 | 2.06E-14 |
| *TRBV12-3* | 0.257268239 | 1.177442857 | 2.19431178 | 6.23E-16 | 2.13E-14 |
| *C1R* | 121.129428 | 279.702719 | 1.207344873 | 6.44E-16 | 2.20E-14 |
| *FPR1* | 6.346980189 | 17.46443929 | 1.46027808 | 6.50E-16 | 2.22E-14 |
| *FAM20A* | 4.901101258 | 11.97919048 | 1.289352559 | 6.56E-16 | 2.23E-14 |
| *NRROS* | 3.56642956 | 7.431525 | 1.059177813 | 6.79E-16 | 2.31E-14 |
| *IGHV3-21* | 77.59848616 | 303.3084417 | 1.966685239 | 6.89E-16 | 2.34E-14 |
| *PDE4B* | 2.882453459 | 6.47279881 | 1.167092348 | 7.03E-16 | 2.38E-14 |
| *ADGRE1* | 1.539029245 | 4.357134524 | 1.50135901 | 7.18E-16 | 2.43E-14 |
| *IGLV5-45* | 8.780798428 | 94.70946548 | 3.431084586 | 7.55E-16 | 2.55E-14 |
| *TRGV5P* | 0.389834591 | 1.541033333 | 1.982964052 | 7.91E-16 | 2.67E-14 |
| *AC243962.1* | 0.147836478 | 0.553483333 | 1.904537583 | 7.95E-16 | 2.68E-14 |
| *IGLC3* | 304.7053308 | 1166.556785 | 1.93676989 | 8.21E-16 | 2.76E-14 |
| *LGALS2* | 3.815048113 | 12.3152869 | 1.690677081 | 8.35E-16 | 2.80E-14 |
| *FGD2* | 2.134669182 | 4.592036905 | 1.105121729 | 8.42E-16 | 2.82E-14 |
| *LINC00460* | 0.55398239 | 2.372552381 | 2.098527918 | 9.34E-16 | 3.12E-14 |
| *CD40LG* | 0.667386478 | 1.398996429 | 1.067797918 | 1.00E-15 | 3.34E-14 |
| *AC092580.2* | 0.077907862 | 0.251969048 | 1.693405699 | 1.03E-15 | 3.44E-14 |
| *LINC00892* | 0.297134591 | 0.617413095 | 1.055119515 | 1.04E-15 | 3.47E-14 |
| *IGHV3-23* | 158.3483767 | 469.579281 | 1.568266676 | 1.08E-15 | 3.58E-14 |
| *SIRPB1* | 0.60775 | 1.562659524 | 1.362453581 | 1.11E-15 | 3.69E-14 |
| *MAPK10* | 2.26223805 | 0.933517857 | -1.277001224 | 1.18E-15 | 3.89E-14 |
| *LY96* | 19.73531038 | 42.29015476 | 1.099542631 | 1.25E-15 | 4.13E-14 |
| *TRAV8-1* | 0.310693711 | 1.152360714 | 1.89102744 | 1.27E-15 | 4.19E-14 |
| *CLEC10A* | 3.364587421 | 9.338759524 | 1.472801321 | 1.36E-15 | 4.49E-14 |
| *KSR2* | 1.046010692 | 0.233890476 | -2.160992575 | 1.38E-15 | 4.54E-14 |
| *NAIPP1* | 0.205611635 | 0.592702381 | 1.527385946 | 1.44E-15 | 4.72E-14 |

| *ANTXRLP1* | 0.082510692 | 0.177092857 | 1.101853041 | 1.48E-15 | 4.84E-14 |
| --- | --- | --- | --- | --- | --- |
| *EBI3* | 5.028500943 | 10.41524643 | 1.05049669 | 1.51E-15 | 4.93E-14 |
| *CGAS* | 9.635806918 | 20.03432262 | 1.055996341 | 1.55E-15 | 5.05E-14 |
| *GIMAP5* | 0.192123585 | 0.440582143 | 1.197376389 | 1.62E-15 | 5.27E-14 |
| *MAML3* | 9.254208805 | 4.393521429 | -1.074731921 | 1.63E-15 | 5.30E-14 |
| *MILR1* | 5.148018239 | 12.45416905 | 1.274539697 | 1.78E-15 | 5.76E-14 |
| *HMGCS2* | 338.6205289 | 50.54622976 | -2.743994049 | 1.89E-15 | 6.13E-14 |
| *PLAUR* | 33.2531 | 72.07600119 | 1.116030131 | 2.00E-15 | 6.48E-14 |
| *P2RX7* | 0.630245283 | 1.462821429 | 1.214768345 | 2.09E-15 | 6.75E-14 |
| *LILRA2* | 0.419901258 | 1.034378571 | 1.300642278 | 2.11E-15 | 6.80E-14 |
| *MOB3B* | 5.46825566 | 14.3926881 | 1.396183466 | 2.12E-15 | 6.85E-14 |
| *PTCRA* | 0.266631132 | 0.775722619 | 1.540695629 | 2.15E-15 | 6.93E-14 |
| *MARCO* | 11.82405409 | 28.95873929 | 1.292274022 | 2.16E-15 | 6.93E-14 |
| *AC007569.1* | 0.207484277 | 0.462566667 | 1.156659294 | 2.18E-15 | 6.99E-14 |
| *IFT140* | 15.56823365 | 7.418935714 | -1.069321123 | 2.21E-15 | 7.08E-14 |
| *PTPRN2-AS1* | 0.264427673 | 0.661046429 | 1.321878437 | 2.25E-15 | 7.20E-14 |
| *CASS4* | 0.706066352 | 1.477861905 | 1.065635795 | 2.31E-15 | 7.39E-14 |
| *LINC00930* | 6.137996226 | 1.000907143 | -2.616459622 | 2.34E-15 | 7.46E-14 |
| *AL161669.1* | 0.189151258 | 1.035429762 | 2.452617417 | 2.37E-15 | 7.56E-14 |
| *AC124014.1* | 0.254096226 | 0.535182143 | 1.074655029 | 2.58E-15 | 8.19E-14 |
| *BIRC3* | 11.61888113 | 37.35971786 | 1.685012412 | 2.81E-15 | 8.91E-14 |
| *SOD2* | 48.05771698 | 105.1782024 | 1.129995726 | 2.93E-15 | 9.28E-14 |
| *HRH2* | 2.529318553 | 5.485125 | 1.116775751 | 3.09E-15 | 9.74E-14 |
| *IGKV4-1* | 227.9450792 | 588.9085869 | 1.369357444 | 3.11E-15 | 9.81E-14 |
| *GPR132* | 3.448839308 | 7.301841667 | 1.082149475 | 3.25E-15 | 1.02E-13 |
| *KLRK1* | 0.270042767 | 0.614577381 | 1.186406762 | 3.25E-15 | 1.02E-13 |
| *IGHV3-33* | 25.49223208 | 155.835131 | 2.611890901 | 3.30E-15 | 1.04E-13 |
| *HS3ST3B1* | 1.869908491 | 3.880911905 | 1.053428016 | 3.36E-15 | 1.05E-13 |
| *KLRC4* | 0.062545283 | 0.249002381 | 1.993186548 | 3.53E-15 | 1.10E-13 |
| *IGKV3-20* | 260.6941113 | 1413.892433 | 2.439242466 | 3.55E-15 | 1.11E-13 |
| *IGLV3-21* | 143.3731865 | 630.7865774 | 2.137376723 | 3.97E-15 | 1.24E-13 |
| *IGKC* | 5878.350681 | 20997.07631 | 1.836705125 | 3.97E-15 | 1.24E-13 |
| *L3MBTL4-AS1* | 0.196083648 | 0.428583333 | 1.128106519 | 4.19E-15 | 1.30E-13 |
| *ARNTL2* | 17.9896195 | 39.52222738 | 1.135499584 | 4.22E-15 | 1.31E-13 |
| *IGLC2* | 458.868644 | 1792.734961 | 1.966009084 | 4.25E-15 | 1.32E-13 |
| *KIF28P* | 1.048263836 | 0.344511905 | -1.605376131 | 4.62E-15 | 1.43E-13 |
| *CCR8* | 0.735937736 | 1.690355952 | 1.199671462 | 4.68E-15 | 1.44E-13 |
| *CR1L* | 0.128373585 | 0.419940476 | 1.709836476 | 5.13E-15 | 1.58E-13 |
| *RPL4P1* | 0.138737736 | 0.414788095 | 1.580014243 | 5.23E-15 | 1.60E-13 |
| *LINC02577* | 1.512679874 | 3.252430952 | 1.104411725 | 5.32E-15 | 1.63E-13 |
| *CD300E* | 1.784619811 | 6.981459524 | 1.967911913 | 5.61E-15 | 1.71E-13 |
| *IGLV1-40* | 123.8839695 | 532.3323202 | 2.103337646 | 6.46E-15 | 1.96E-13 |
| *TRAV35* | 0.2007 | 0.895430952 | 2.157541572 | 6.48E-15 | 1.97E-13 |
| *CSF2RB* | 7.879919497 | 17.85397381 | 1.179992419 | 7.64E-15 | 2.31E-13 |
| *RNASE2* | 2.937287421 | 7.30537619 | 1.314474122 | 7.82E-15 | 2.37E-13 |
| *HLA-DPA3* | 0.662499371 | 2.410109524 | 1.863107719 | 7.86E-15 | 2.37E-13 |

| *VMO1* | 10.72343994 | 23.10228452 | 1.107267746 | 7.90E-15 | 2.38E-13 |
| --- | --- | --- | --- | --- | --- |
| *GGTA1* | 2.177600943 | 4.873269048 | 1.162150277 | 8.10E-15 | 2.44E-13 |
| *AC084871.3* | 0.183025157 | 0.733414286 | 2.002586404 | 8.10E-15 | 2.44E-13 |
| *BST2* | 504.7891613 | 1070.543013 | 1.084589924 | 8.38E-15 | 2.51E-13 |
| *CCSER1* | 2.903655975 | 0.834922619 | -1.798156134 | 8.59E-15 | 2.58E-13 |
| *AC008964.1* | 0.090463522 | 0.228339286 | 1.335771026 | 8.96E-15 | 2.68E-13 |
| *IGLV3-1* | 153.0136588 | 691.1249071 | 2.175286032 | 9.41E-15 | 2.81E-13 |
| *LINC02362* | 0.311628931 | 0.918921429 | 1.560112335 | 9.53E-15 | 2.85E-13 |
| *IGHV3-69-1* | 2.350480503 | 10.46302976 | 2.154273052 | 9.57E-15 | 2.85E-13 |
| *TOX3* | 15.43414717 | 3.915232143 | -1.978956012 | 1.07E-14 | 3.17E-13 |
| *ISG15* | 565.8825654 | 1281.262426 | 1.178991402 | 1.10E-14 | 3.26E-13 |
| *TRBV25-1* | 0.174098742 | 0.789930952 | 2.181820774 | 1.12E-14 | 3.32E-13 |
| *CCL23* | 1.638268553 | 3.502284524 | 1.096124422 | 1.32E-14 | 3.88E-13 |
| *IGKV1-5* | 166.389145 | 563.8628357 | 1.760782941 | 1.35E-14 | 3.99E-13 |
| *KCNJ10* | 0.377300629 | 1.447055952 | 1.939334297 | 1.46E-14 | 4.29E-13 |
| *IGLV3-19* | 105.7684623 | 628.2754917 | 2.57048779 | 1.66E-14 | 4.88E-13 |
| *CD101* | 1.382179245 | 2.871389286 | 1.054804215 | 1.68E-14 | 4.92E-13 |
| *IGLV1-47* | 88.93174277 | 212.1366333 | 1.254223415 | 1.73E-14 | 5.06E-13 |
| *AC099063.4* | 0.238427987 | 0.518707143 | 1.121366643 | 1.89E-14 | 5.52E-13 |
| *KLHDC7B* | 55.76246541 | 237.7089643 | 2.091830057 | 1.90E-14 | 5.53E-13 |
| *IFI6* | 970.0866283 | 2022.353726 | 1.059849868 | 2.03E-14 | 5.89E-13 |
| *SPATC1* | 0.257250943 | 0.586809524 | 1.189715918 | 2.06E-14 | 5.96E-13 |
| *IGLV5-48* | 0.625526101 | 1.666713095 | 1.413863794 | 2.27E-14 | 6.57E-13 |
| *IGLV2-23* | 91.86656887 | 348.4338012 | 1.923272734 | 2.31E-14 | 6.68E-13 |
| *BCAS1* | 36.97695472 | 9.854310714 | -1.907799548 | 2.35E-14 | 6.79E-13 |
| *IGHV3-30* | 61.87966918 | 159.2884524 | 1.364104293 | 2.38E-14 | 6.86E-13 |
| *AC083837.1* | 1.812962579 | 4.037182143 | 1.154999531 | 2.47E-14 | 7.11E-13 |
| *FMO9P* | 39.7169066 | 19.2958131 | -1.041465423 | 2.68E-14 | 7.69E-13 |
| *PTBP1P* | 0.114034277 | 0.249655952 | 1.130473766 | 2.94E-14 | 8.41E-13 |
| *GPR183* | 13.54141604 | 30.31264048 | 1.162540917 | 3.03E-14 | 8.65E-13 |
| *SERPINB1* | 110.3742494 | 230.3681429 | 1.061537597 | 3.10E-14 | 8.86E-13 |
| *HLA-DQA2* | 31.39557642 | 103.9225976 | 1.726876192 | 3.13E-14 | 8.92E-13 |
| *HERC6* | 13.86347421 | 28.63617619 | 1.046550016 | 3.18E-14 | 9.05E-13 |
| *IL1R2* | 4.581923585 | 17.33195119 | 1.919408775 | 3.26E-14 | 9.27E-13 |
| *SRCIN1* | 6.658199057 | 2.251890476 | -1.563995341 | 3.45E-14 | 9.80E-13 |
| *LINC02672* | 29.78282264 | 5.092995238 | -2.547894217 | 3.55E-14 | 1.01E-12 |
| *SLC11A1* | 3.592451572 | 7.960477381 | 1.147886241 | 3.57E-14 | 1.01E-12 |
| *CASP1P2* | 0.852834906 | 3.783617857 | 2.149427992 | 3.60E-14 | 1.02E-12 |
| *CLEC9A* | 0.196601258 | 0.490571429 | 1.31919066 | 3.85E-14 | 1.09E-12 |
| *IGHV4-34* | 44.30367264 | 139.4021167 | 1.653754263 | 4.11E-14 | 1.16E-12 |
| *IGHV6-1* | 32.06593491 | 88.08649405 | 1.457879363 | 4.21E-14 | 1.18E-12 |
| *CAB39L* | 37.01775912 | 16.481275 | -1.167389709 | 4.40E-14 | 1.24E-12 |
| *SLC2A5* | 2.542354717 | 5.345040476 | 1.072035539 | 4.47E-14 | 1.25E-12 |
| *SPINK1* | 1280.606937 | 161.6840262 | -2.985578672 | 4.53E-14 | 1.27E-12 |
| *MAFB* | 29.78537075 | 69.4227 | 1.220803559 | 5.39E-14 | 1.50E-12 |
| *SUGCT-AS1* | 0.247439308 | 0.591140476 | 1.256426302 | 5.69E-14 | 1.58E-12 |

| *PPP1R2P1* | 0.10284717 | 0.381217857 | 1.890113606 | 6.33E-14 | 1.75E-12 |
| --- | --- | --- | --- | --- | --- |
| *XXYLT1-AS2* | 0.420388994 | 0.998184524 | 1.247581638 | 6.38E-14 | 1.76E-12 |
| *GPR82* | 0.566941509 | 1.36632381 | 1.269027626 | 6.54E-14 | 1.80E-12 |
| *HERC5* | 8.289447799 | 16.74190714 | 1.014115976 | 7.32E-14 | 2.01E-12 |
| *IFIT1* | 20.21531069 | 45.48582857 | 1.169968757 | 7.62E-14 | 2.09E-12 |
| *SMCO2* | 1.004628931 | 2.24349881 | 1.159087692 | 7.68E-14 | 2.10E-12 |
| *ETV7-AS1* | 0.713546855 | 1.773329762 | 1.313380766 | 7.89E-14 | 2.15E-12 |
| *S* *A1* | 79.08928176 | 418.790706 | 2.404675328 | 8.00E-14 | 2.18E-12 |
| *IGLV1-44* | 89.25817736 | 303.5504929 | 1.765880263 | 8.44E-14 | 2.29E-12 |
| *MMP2-AS1* | 0.434148428 | 1.434803571 | 1.724592977 | 8.46E-14 | 2.30E-12 |
| *HLA-DRB9* | 0.820447484 | 2.3592 | 1.523814831 | 9.18E-14 | 2.49E-12 |
| *CLEC4A* | 0.129152201 | 0.304658333 | 1.23811997 | 9.64E-14 | 2.60E-12 |
| *TMEM176A* | 23.49198616 | 49.38582262 | 1.071928248 | 9.85E-14 | 2.66E-12 |
| *ERVE-1* | 8.017837421 | 2.966452381 | -1.43447454 | 1.00E-13 | 2.70E-12 |
| *AC091544.4* | 1.388317296 | 0.291082143 | -2.253839087 | 1.00E-13 | 2.70E-12 |
| *UPK3A* | 252.6659164 | 35.0270881 | -2.850688101 | 1.04E-13 | 2.80E-12 |
| *TRDJ1* | 0.483418868 | 1.90739881 | 1.980260834 | 1.07E-13 | 2.87E-12 |
| *C5AR2* | 0.828627987 | 3.032622619 | 1.871769525 | 1.09E-13 | 2.92E-12 |
| *CLEC5A* | 1.733522642 | 4.060091667 | 1.227805621 | 1.11E-13 | 2.97E-12 |
| *AC007278.1* | 0.085820755 | 0.26822619 | 1.64405162 | 1.11E-13 | 2.98E-12 |
| *HS6ST3* | 3.445786478 | 0.890364286 | -1.952365677 | 1.14E-13 | 3.03E-12 |
| *NRIR* | 0.510771069 | 1.140525 | 1.158949354 | 1.21E-13 | 3.21E-12 |
| *MT2A* | 667.2624664 | 1968.949717 | 1.561100009 | 1.23E-13 | 3.27E-12 |
| *AC127070.1* | 2.161629874 | 0.970103571 | -1.155908831 | 1.24E-13 | 3.29E-12 |
| *GCNT1* | 3.04909717 | 6.415205952 | 1.073113455 | 1.29E-13 | 3.41E-12 |
| *OVGP1* | 8.675325786 | 2.48867381 | -1.801540788 | 1.31E-13 | 3.47E-12 |
| *TRBV7-4* | 0.233010377 | 0.621379762 | 1.415081046 | 1.32E-13 | 3.48E-12 |
| *TNFRSF17* | 2.158809434 | 5.209155952 | 1.270813732 | 1.33E-13 | 3.51E-12 |
| *IGHV3-11* | 42.11805 | 155.319619 | 1.882729525 | 1.37E-13 | 3.62E-12 |
| *IGKV3-15* | 86.98631855 | 331.3512738 | 1.929501053 | 1.40E-13 | 3.68E-12 |
| *SLC14A1* | 52.10783082 | 12.49701071 | -2.059917155 | 1.48E-13 | 3.89E-12 |
| *AL390755.2* | 0.12521195 | 3.288580952 | 4.715021026 | 1.49E-13 | 3.92E-12 |
| *AC015819.1* | 0.87917044 | 2.017571429 | 1.198404965 | 1.50E-13 | 3.93E-12 |
| *TNFSF10* | 134.3029626 | 284.8605405 | 1.084764661 | 1.51E-13 | 3.96E-12 |
| *ALDH1L1-AS2* | 2.054366667 | 0.54457381 | -1.915494194 | 1.59E-13 | 4.16E-12 |
| *NUAK2* | 9.097042767 | 20.46140476 | 1.169435655 | 1.60E-13 | 4.17E-12 |
| *HIVEP3* | 2.25093522 | 4.825646429 | 1.100197676 | 1.71E-13 | 4.43E-12 |
| *AC011899.2* | 0.251618868 | 0.596080952 | 1.244268165 | 1.72E-13 | 4.46E-12 |
| *HLA-DQB2* | 15.26025535 | 37.71576905 | 1.305388741 | 1.72E-13 | 4.46E-12 |
| *LINC01914* | 0.413924843 | 2.844261905 | 2.780613574 | 1.76E-13 | 4.55E-12 |
| *DERL3* | 1.814301887 | 4.156046429 | 1.195797241 | 1.77E-13 | 4.59E-12 |
| *IGKV1-17* | 37.0387978 | 134.729356 | 1.862955054 | 1.89E-13 | 4.88E-12 |
| *AC008750.1* | 0.120480818 | 0.36414881 | 1.595724663 | 2.03E-13 | 5.20E-12 |
| *AL023653.1* | 0.605205975 | 1.247819048 | 1.043910601 | 2.20E-13 | 5.63E-12 |
| *IGHV1-69D* | 32.00818113 | 187.7873369 | 2.552587178 | 2.22E-13 | 5.66E-12 |
| *IGHV4-59* | 46.27962736 | 204.559281 | 2.144069841 | 2.46E-13 | 6.26E-12 |

| *TMEM244* | 0.082882704 | 0.253741667 | 1.614217459 | 2.51E-13 | 6.36E-12 |
| --- | --- | --- | --- | --- | --- |
| *LRFN2* | 1.870856918 | 0.42335119 | -2.143772375 | 2.65E-13 | 6.72E-12 |
| *RHOF* | 0.378519182 | 0.912435714 | 1.269356503 | 2.77E-13 | 7.02E-12 |
| *IGHV3-48* | 49.14756132 | 191.1138071 | 1.959240273 | 2.91E-13 | 7.36E-12 |
| *IGHGP* | 246.6007258 | 1066.41561 | 2.112520852 | 2.94E-13 | 7.41E-12 |
| *GDF7* | 2.845794025 | 0.916778571 | -1.634186017 | 2.95E-13 | 7.43E-12 |
| *CD300H* | 0.420986164 | 1.067688095 | 1.34264553 | 3.03E-13 | 7.63E-12 |
| *TSHR* | 0.076924843 | 0.472267857 | 2.618083853 | 3.11E-13 | 7.82E-12 |
| *ZBTB7C* | 22.35261415 | 8.866271429 | -1.334044131 | 3.18E-13 | 7.97E-12 |
| *VIPR1-AS1* | 0.568750629 | 0.174366667 | -1.705671966 | 3.40E-13 | 8.51E-12 |
| *TRBV14* | 0.342271384 | 1.147733333 | 1.745574899 | 3.41E-13 | 8.51E-12 |
| *IGHV3-53* | 7.672533019 | 29.84285714 | 1.959610811 | 3.45E-13 | 8.59E-12 |
| *FAM3D* | 17.67826164 | 3.001786905 | -2.558082947 | 3.52E-13 | 8.75E-12 |
| *KRT81* | 20.41291918 | 261.1105119 | 3.677106122 | 3.83E-13 | 9.49E-12 |
| *AC007036.1* | 0.226203459 | 0.4789 | 1.082103444 | 3.91E-13 | 9.66E-12 |
| *C1S* | 111.6458403 | 242.2702976 | 1.117688041 | 3.95E-13 | 9.78E-12 |
| *CYP4Z2P* | 3.798142138 | 1.079058333 | -1.815521038 | 4.12E-13 | 1.02E-11 |
| *HLA-S* | 1.21721761 | 3.453847619 | 1.504617322 | 4.16E-13 | 1.03E-11 |
| *IGKV1-39* | 4.116262893 | 26.78848333 | 2.702205873 | 4.39E-13 | 1.08E-11 |
| *AC092329.4* | 2.539939623 | 0.897869048 | -1.500217252 | 4.39E-13 | 1.08E-11 |
| *AC034199.1* | 0.078740252 | 0.229853571 | 1.545541856 | 4.47E-13 | 1.10E-11 |
| *SPHK1* | 22.20328239 | 50.48824286 | 1.185174497 | 4.48E-13 | 1.10E-11 |
| *CFB* | 0.947231761 | 2.135021429 | 1.172461189 | 4.66E-13 | 1.14E-11 |
| *OSM* | 4.286397484 | 10.93942024 | 1.351698736 | 4.82E-13 | 1.18E-11 |
| *TMPRSS2* | 53.29112893 | 20.14567857 | -1.403424995 | 5.11E-13 | 1.24E-11 |
| *ADAT* | 12.41278428 | 6.138930952 | -1.01576741 | 5.23E-13 | 1.27E-11 |
| *CAPN5* | 45.75425566 | 21.26911071 | -1.105146223 | 5.23E-13 | 1.27E-11 |
| *REEP6* | 88.09816384 | 41.1622119 | -1.097791441 | 5.40E-13 | 1.31E-11 |
| *IGHV3-66* | 5.350488365 | 23.33924643 | 2.125015496 | 5.46E-13 | 1.32E-11 |
| *IGLV6-57* | 80.58452296 | 335.2897107 | 2.056833524 | 5.68E-13 | 1.37E-11 |
| *IGHV4-4* | 26.1237217 | 175.0055274 | 2.74396814 | 5.79E-13 | 1.40E-11 |
| *SLC23A3* | 0.950157862 | 0.425916667 | -1.157596041 | 6.06E-13 | 1.46E-11 |
| *FOXH1* | 1.634840566 | 0.334457143 | -2.289256685 | 6.56E-13 | 1.58E-11 |
| *ELF5* | 11.6445066 | 4.766341667 | -1.288695234 | 6.65E-13 | 1.60E-11 |
| *MFAP3L* | 10.22485409 | 4.552464286 | -1.167360651 | 6.71E-13 | 1.61E-11 |
| *IGKV5-2* | 2.462109748 | 30.31742143 | 3.622180078 | 7.06E-13 | 1.68E-11 |
| *EVX1* | 6.922364151 | 1.981947619 | -1.804346002 | 7.18E-13 | 1.71E-11 |
| *AC004080.1* | 11.09681541 | 1.352492857 | -3.036452828 | 7.27E-13 | 1.73E-11 |
| *IGLV2-14* | 147.1888459 | 461.829 | 1.649690421 | 7.47E-13 | 1.78E-11 |
| *DSE* | 4.517467925 | 9.750347619 | 1.109939297 | 7.83E-13 | 1.86E-11 |
| *CNGA1* | 15.87627107 | 4.707929762 | -1.753707398 | 7.95E-13 | 1.88E-11 |
| *AL671883.2* | 0.273186792 | 0.602638095 | 1.141404138 | 8.00E-13 | 1.89E-11 |
| *CAPN9* | 4.597391195 | 0.671139286 | -2.776131317 | 8.13E-13 | 1.92E-11 |
| *TRAV1-1* | 0.177317296 | 0.762583333 | 2.104561736 | 8.27E-13 | 1.95E-11 |
| *CEBPE* | 0.257590881 | 0.521022619 | 1.016264487 | 8.28E-13 | 1.95E-11 |
| *IGHV1-2* | 65.85781258 | 216.9041071 | 1.719630871 | 8.94E-13 | 2.10E-11 |

| *IGHV1-18* | 108.8216336 | 425.0700202 | 1.965735119 | 8.95E-13 | 2.10E-11 |
| --- | --- | --- | --- | --- | --- |
| *GATA3-AS1* | 17.37678333 | 5.505680952 | -1.65816813 | 9.15E-13 | 2.15E-11 |
| *AC019117.2* | 3.822236478 | 1.303313095 | -1.552233335 | 9.28E-13 | 2.17E-11 |
| *AL158206.1* | 204.4802314 | 70.00462262 | -1.546439278 | 9.56E-13 | 2.24E-11 |
| *TRBV6-1* | 0.707358805 | 2.270032143 | 1.682198618 | 1.11E-12 | 2.60E-11 |
| *IGLL5* | 1.544800629 | 4.073230952 | 1.398752961 | 1.13E-12 | 2.64E-11 |
| *MICB* | 9.442248742 | 19.27027857 | 1.029175033 | 1.14E-12 | 2.66E-11 |
| *LINC01341* | 2.510858805 | 0.964964286 | -1.37963345 | 1.15E-12 | 2.67E-11 |
| *TRAV34* | 0.102446226 | 0.501440476 | 2.291211611 | 1.20E-12 | 2.79E-11 |
| *AL591848.3* | 1.97613805 | 0.742269048 | -1.41266962 | 1.21E-12 | 2.80E-11 |
| *HSD17B2* | 15.39995975 | 2.927883333 | -2.394996607 | 1.24E-12 | 2.87E-11 |
| *ZNF440* | 22.2365934 | 11.07441905 | -1.005704768 | 1.33E-12 | 3.07E-11 |
| *MT-ND5* | 5203.43395 | 2488.580698 | -1.064140858 | 1.40E-12 | 3.24E-11 |
| *TEX45* | 2.280781761 | 0.803667857 | -1.50485712 | 1.43E-12 | 3.29E-11 |
| *IGLV4-69* | 36.90897013 | 181.8214476 | 2.300479002 | 1.45E-12 | 3.33E-11 |
| *HLA-L* | 3.230409434 | 7.788136905 | 1.269561216 | 1.55E-12 | 3.56E-11 |
| *AC010487.1* | 7.853297484 | 2.407092857 | -1.706006751 | 1.55E-12 | 3.57E-11 |
| *IGLV3-10* | 42.7648173 | 168.8780702 | 1.981485717 | 1.61E-12 | 3.70E-11 |
| *AL645939.5* | 0.300091824 | 1.004082143 | 1.742401382 | 1.62E-12 | 3.70E-11 |
| *FKBP5* | 14.21461069 | 29.39413333 | 1.048153654 | 1.66E-12 | 3.79E-11 |
| *AL157871.3* | 0.620971384 | 1.554159524 | 1.323535903 | 1.68E-12 | 3.83E-11 |
| *GPR68* | 11.56749811 | 27.44010238 | 1.246209001 | 1.69E-12 | 3.87E-11 |
| *TSPAN12* | 25.72720597 | 9.93557381 | -1.372619591 | 1.71E-12 | 3.89E-11 |
| *BEND5* | 9.351619811 | 3.951292857 | -1.2428915 | 1.75E-12 | 3.98E-11 |
| *IGHV3-49* | 38.04754654 | 82.66867143 | 1.119537275 | 1.75E-12 | 3.98E-11 |
| *BTBD16* | 64.96721478 | 9.646941667 | -2.751568309 | 1.79E-12 | 4.08E-11 |
| *ZDHHC20P1* | 0.449551887 | 1.90697381 | 2.084725485 | 1.83E-12 | 4.15E-11 |
| *IGHV1OR15-2* | 1.814116981 | 6.161910714 | 1.764110289 | 1.96E-12 | 4.44E-11 |
| *SORL1* | 25.52698648 | 9.438030952 | -1.435465426 | 2.05E-12 | 4.63E-11 |
| *IGHV4-39* | 87.2329739 | 353.6512071 | 2.019381707 | 2.08E-12 | 4.69E-11 |
| *PLCE1* | 7.763801887 | 3.423294048 | -1.181378085 | 2.11E-12 | 4.76E-11 |
| *TRBJ1-2* | 0.764887736 | 2.235621429 | 1.547355987 | 2.12E-12 | 4.79E-11 |
| *IGHV2-5* | 61.59025723 | 159.3438607 | 1.371369377 | 2.14E-12 | 4.82E-11 |
| *IGKJ1* | 27.77966164 | 79.58784286 | 1.518519047 | 2.16E-12 | 4.87E-11 |
| *ACOXL-AS1* | 1.167273585 | 0.374428571 | -1.640380307 | 2.26E-12 | 5.08E-11 |
| *AC090398.2* | 1.292908176 | 0.601260714 | -1.104557214 | 2.28E-12 | 5.12E-11 |
| *IGKV1-6* | 25.46405346 | 100.0747167 | 1.974543535 | 2.50E-12 | 5.57E-11 |
| *AC008759.1* | 1.114358491 | 0.322135714 | -1.790472902 | 2.57E-12 | 5.73E-11 |
| *MMEL1* | 2.092160377 | 0.710825 | -1.55742712 | 2.62E-12 | 5.82E-11 |
| *AC026765.3* | 1.002505346 | 0.336327381 | -1.575671789 | 2.76E-12 | 6.12E-11 |
| *BHMT* | 46.7299566 | 17.0403369 | -1.455393838 | 2.77E-12 | 6.15E-11 |
| *ZNF321P* | 6.501019497 | 2.788107143 | -1.221379978 | 2.88E-12 | 6.37E-11 |
| *IGLV3-25* | 116.5961038 | 360.8367893 | 1.629826857 | 3.02E-12 | 6.68E-11 |
| *AL135999.3* | 11.44983774 | 4.879121429 | -1.230633859 | 3.09E-12 | 6.82E-11 |
| *EVX1-AS* | 3.32673522 | 0.631105952 | -2.398152909 | 3.18E-12 | 7.00E-11 |
| *MIR3945HG* | 0.124353774 | 0.413336905 | 1.732867893 | 3.22E-12 | 7.08E-11 |

| *TRAJ31* | 0.522048742 | 1.358717857 | 1.379989488 | 3.54E-12 | 7.74E-11 |
| --- | --- | --- | --- | --- | --- |
| *FAM174B* | 67.37992579 | 27.253675 | -1.305868057 | 3.74E-12 | 8.17E-11 |
| *AC139720.1* | 0.255766352 | 0.643997619 | 1.332228874 | 3.75E-12 | 8.18E-11 |
| *GORAB-AS1* | 0.221463522 | 0.490345238 | 1.14672878 | 3.81E-12 | 8.31E-11 |
| *SEMA6A* | 14.73840157 | 5.154352381 | -1.515716991 | 3.94E-12 | 8.58E-11 |
| *IGLV2-5* | 2.397325786 | 8.091778571 | 1.755030863 | 3.95E-12 | 8.60E-11 |
| *IGHJ4* | 13.28983145 | 35.96605595 | 1.436313155 | 4.08E-12 | 8.85E-11 |
| *STK32A* | 3.473960063 | 1.21497619 | -1.515653127 | 4.11E-12 | 8.90E-11 |
| *ACSM6* | 4.280079245 | 1.036458333 | -2.045975388 | 4.15E-12 | 8.98E-11 |
| *TDH* | 2.319768239 | 0.770165476 | -1.590740319 | 4.35E-12 | 9.40E-11 |
| *GPA33* | 0.353235535 | 1.000345238 | 1.501795601 | 4.74E-12 | 1.02E-10 |
| *IGHV3-71* | 1.171984906 | 2.717785714 | 1.213477721 | 4.77E-12 | 1.03E-10 |
| *APOBEC3H* | 3.600198428 | 7.497182143 | 1.058272029 | 4.80E-12 | 1.03E-10 |
| *IGLV9-49* | 8.228199057 | 39.75250714 | 2.272397251 | 4.94E-12 | 1.06E-10 |
| *IGHV4-31* | 23.92705849 | 97.94157024 | 2.033278278 | 4.97E-12 | 1.07E-10 |
| *HID1* | 52.08502862 | 22.08834167 | -1.237583633 | 5.12E-12 | 1.09E-10 |
| *TRBV13* | 0.462033333 | 1.372580952 | 1.570822396 | 5.20E-12 | 1.11E-10 |
| *AC135068.3* | 0.516747799 | 3.789214286 | 2.874366486 | 5.23E-12 | 1.12E-10 |
| *GPLD1* | 2.734592138 | 0.844416667 | -1.695298713 | 5.35E-12 | 1.14E-10 |
| *TRBV11-3* | 0.145580818 | 0.635725 | 2.126582552 | 5.68E-12 | 1.21E-10 |
| *IGLV2-11* | 63.15942358 | 213.8692381 | 1.759659077 | 5.79E-12 | 1.23E-10 |
| *GRHL3* | 100.1293868 | 45.79640119 | -1.128559313 | 5.93E-12 | 1.26E-10 |
| *IGHV3-73* | 17.27401226 | 69.12681429 | 2.000642221 | 6.24E-12 | 1.32E-10 |
| *TTC23L* | 1.180165094 | 0.34855 | -1.759551161 | 6.27E-12 | 1.32E-10 |
| *AP000866.5* | 2.108174528 | 0.676075 | -1.640739102 | 6.27E-12 | 1.32E-10 |
| *DPYD* | 8.620468239 | 18.23919881 | 1.081204218 | 6.39E-12 | 1.34E-10 |
| *SAMD9* | 20.31019686 | 41.89817381 | 1.04468314 | 6.39E-12 | 1.34E-10 |
| *IFI27* | 228.3250119 | 466.1395976 | 1.029673163 | 6.44E-12 | 1.35E-10 |
| *IGKV2D-40* | 8.871336164 | 42.77403095 | 2.269511852 | 6.52E-12 | 1.37E-10 |
| *PACRG* | 2.602681132 | 0.601560714 | -2.113216312 | 6.60E-12 | 1.39E-10 |
| *IGHV3-7* | 80.28008931 | 193.0246274 | 1.265670801 | 6.62E-12 | 1.39E-10 |
| *SYNGR1* | 15.32116069 | 7.133575 | -1.102828425 | 6.63E-12 | 1.39E-10 |
| *SNTG1* | 0.936299371 | 0.179282143 | -2.384738091 | 7.44E-12 | 1.55E-10 |
| *MYCL* | 94.36217799 | 36.22801667 | -1.381102892 | 7.52E-12 | 1.57E-10 |
| *RNF128* | 99.37244937 | 37.3998119 | -1.409814911 | 7.89E-12 | 1.64E-10 |
| *ADGRG3* | 1.10498522 | 2.223094048 | 1.00854191 | 8.03E-12 | 1.66E-10 |
| *VNN1* | 1.054745283 | 7.687354762 | 2.865592613 | 8.24E-12 | 1.70E-10 |
| *SIGLEC12* | 1.356179874 | 4.400605952 | 1.698153653 | 8.38E-12 | 1.73E-10 |
| *AC008760.2* | 1.372862264 | 4.348310714 | 1.663268143 | 8.48E-12 | 1.75E-10 |
| *IGHJ3* | 19.91361635 | 54.55079881 | 1.453845082 | 8.93E-12 | 1.84E-10 |
| *EXTL3-AS1* | 0.529146855 | 0.230538095 | -1.198663004 | 8.97E-12 | 1.84E-10 |
| *THRB* | 10.49764497 | 5.04652381 | -1.056703846 | 8.97E-12 | 1.84E-10 |
| *TRBV12-5* | 0.099783648 | 0.39330119 | 1.978759237 | 9.19E-12 | 1.89E-10 |
| *MPPED2* | 2.759452516 | 1.14879881 | -1.264255901 | 9.44E-12 | 1.93E-10 |
| *TLR10* | 0.83417673 | 2.009085714 | 1.268114144 | 9.55E-12 | 1.95E-10 |
| *FAM163B* | 0.253371069 | 0.591694048 | 1.22359958 | 9.92E-12 | 2.02E-10 |

| *UPK2* | 1117.270543 | 282.3065417 | -1.984644107 | 9.94E-12 | 2.03E-10 |
| --- | --- | --- | --- | --- | --- |
| *ANKRD36BP2* | 0.313310377 | 0.787971429 | 1.330550764 | 1.01E-11 | 2.06E-10 |
| *AC007998.4* | 5.988609434 | 2.086527381 | -1.521117193 | 1.02E-11 | 2.08E-10 |
| *LINC01612* | 3.194846226 | 0.765885714 | -2.060545459 | 1.03E-11 | 2.08E-10 |
| *IGKJ5* | 5.202857547 | 13.53886667 | 1.379730864 | 1.03E-11 | 2.10E-10 |
| *CASKIN1* | 0.654545912 | 0.158909524 | -2.0422888 | 1.05E-11 | 2.14E-10 |
| *RBBP8NL* | 20.91872484 | 10.34317976 | -1.016115135 | 1.08E-11 | 2.18E-10 |
| *FOLR2* | 24.03846226 | 50.01543929 | 1.0570289 | 1.09E-11 | 2.20E-10 |
| *LINC01825* | 0.061075786 | 0.25264881 | 2.048460948 | 1.11E-11 | 2.24E-10 |
| *TTC6* | 1.344991509 | 0.525186905 | -1.356694216 | 1.11E-11 | 2.25E-10 |
| *IFNB1* | 0.076815094 | 0.364179762 | 2.245189015 | 1.18E-11 | 2.36E-10 |
| *SHH* | 17.98716981 | 2.049433333 | -3.133671238 | 1.18E-11 | 2.37E-10 |
| *SLC9A4* | 8.00525 | 1.753136905 | -2.191007796 | 1.20E-11 | 2.41E-10 |
| *TBX2-AS1* | 31.67981667 | 8.193472619 | -1.951017046 | 1.21E-11 | 2.42E-10 |
| *IGHV3-15* | 57.39115535 | 143.3275845 | 1.320415972 | 1.23E-11 | 2.47E-10 |
| *HLA-G* | 8.184839308 | 17.59631429 | 1.104247277 | 1.25E-11 | 2.49E-10 |
| *MYLPF* | 4.303701887 | 1.030488095 | -2.062250309 | 1.26E-11 | 2.51E-10 |
| *IGHG2* | 276.9079846 | 809.3100167 | 1.547285797 | 1.27E-11 | 2.53E-10 |
| *AC092112.1* | 0.184784591 | 0.852480952 | 2.205823141 | 1.27E-11 | 2.53E-10 |
| *AL451123.1* | 0.286201572 | 0.830495238 | 1.536940298 | 1.28E-11 | 2.54E-10 |
| *IGHV4-28* | 7.189054403 | 24.95149286 | 1.795252209 | 1.29E-11 | 2.57E-10 |
| *IGKV1-12* | 31.32870063 | 75.68222857 | 1.272469637 | 1.32E-11 | 2.62E-10 |
| *LINC01010* | 0.160712893 | 0.398697619 | 1.310809317 | 1.32E-11 | 2.63E-10 |
| *FREM2* | 3.471645283 | 0.847088095 | -2.035035628 | 1.33E-11 | 2.63E-10 |
| *IGF2BP2* | 15.01115629 | 35.84579643 | 1.255768837 | 1.33E-11 | 2.64E-10 |
| *AC023421.2* | 4.045292767 | 0.734419048 | -2.461568736 | 1.46E-11 | 2.89E-10 |
| *RAP1GAP* | 21.33285252 | 8.88205119 | -1.264112097 | 1.48E-11 | 2.91E-10 |
| *HS3ST5* | 1.553649686 | 0.364140476 | -2.093094226 | 1.48E-11 | 2.91E-10 |
| *IGKV3OR2-268* | 2.329724528 | 4.959030952 | 1.089898853 | 1.49E-11 | 2.93E-10 |
| *VIPR1* | 10.97694748 | 4.565464286 | -1.265643431 | 1.51E-11 | 2.96E-10 |
| *IGHV1-24* | 49.06756069 | 121.6375024 | 1.309746641 | 1.51E-11 | 2.96E-10 |
| *ABAT* | 10.82909497 | 3.950420238 | -1.454834638 | 1.59E-11 | 3.10E-10 |
| *AC026765.4* | 0.313574528 | 0.080616667 | -1.959658337 | 1.65E-11 | 3.22E-10 |
| *TGM2* | 107.0877456 | 293.7595869 | 1.455842538 | 1.66E-11 | 3.23E-10 |
| *IGKV1-27* | 24.71418711 | 88.43060119 | 1.839206243 | 1.69E-11 | 3.30E-10 |
| *AC091544.5* | 1.035579245 | 0.324017857 | -1.676292727 | 1.73E-11 | 3.37E-10 |
| *IGLV3-16* | 1.231579245 | 4.319675 | 1.810413312 | 1.80E-11 | 3.50E-10 |
| *AL137186.3* | 0.254203459 | 0.769335714 | 1.597629621 | 1.80E-11 | 3.50E-10 |
| *TEKT5* | 3.626935535 | 1.193675 | -1.603341013 | 1.83E-11 | 3.55E-10 |
| *SIRPA* | 23.72834057 | 48.79648095 | 1.040165899 | 1.90E-11 | 3.69E-10 |
| *FADS6* | 3.390663522 | 0.610707143 | -2.473014999 | 2.07E-11 | 4.01E-10 |
| *ACER2* | 57.93513585 | 18.85236786 | -1.619692826 | 2.07E-11 | 4.01E-10 |
| *PPM1L* | 6.896855346 | 3.300472619 | -1.063266079 | 2.09E-11 | 4.04E-10 |
| *TRBJ2-7* | 3.545446226 | 7.306032143 | 1.043120886 | 2.10E-11 | 4.06E-10 |
| *SIGLEC8* | 0.607396226 | 1.678861905 | 1.466773715 | 2.14E-11 | 4.13E-10 |
| *HS3ST6* | 15.32566478 | 5.204728571 | -1.558054821 | 2.17E-11 | 4.17E-10 |

| *SLC9A2* | 10.59677673 | 4.2362 | -1.322782892 | 2.20E-11 | 4.22E-10 |
| --- | --- | --- | --- | --- | --- |
| *FRY* | 7.308133962 | 3.529919048 | -1.049867981 | 2.41E-11 | 4.63E-10 |
| *IGKJ3* | 4.343478302 | 11.45310952 | 1.398816608 | 2.47E-11 | 4.73E-10 |
| *S* *A2* | 3.117550314 | 12.78342024 | 2.035789136 | 2.48E-11 | 4.74E-10 |
| *IGHV5-51* | 99.51107358 | 401.5645024 | 2.01270276 | 2.57E-11 | 4.92E-10 |
| *SLC5A7* | 1.583262579 | 0.426333333 | -1.892846778 | 2.68E-11 | 5.12E-10 |
| *AQP9* | 3.64939717 | 8.081417857 | 1.146950261 | 2.71E-11 | 5.16E-10 |
| *SSH3* | 174.5803393 | 86.63364643 | -1.010891749 | 2.77E-11 | 5.26E-10 |
| *LINC01303* | 0.213910377 | 0.477272619 | 1.157807099 | 2.81E-11 | 5.33E-10 |
| *SCNN1G* | 47.73173239 | 17.70246667 | -1.430998299 | 3.05E-11 | 5.78E-10 |
| *AC004923.1* | 1.550722642 | 0.505110714 | -1.618269123 | 3.11E-11 | 5.89E-10 |
| *TMEM51-AS1* | 2.618242453 | 0.906258333 | -1.530604438 | 3.15E-11 | 5.96E-10 |
| *TRBJ1-3* | 1.116502516 | 3.004625 | 1.428198441 | 3.34E-11 | 6.29E-10 |
| *FUT9* | 2.08956761 | 0.537420238 | -1.959081883 | 3.38E-11 | 6.36E-10 |
| *AC091544.2* | 0.693686478 | 0.13947381 | -2.314289525 | 3.40E-11 | 6.40E-10 |
| *LINC02593* | 2.545541195 | 0.763959524 | -1.736404305 | 3.44E-11 | 6.47E-10 |
| *TTLL11-IT1* | 0.266696226 | 1.072117857 | 2.007194192 | 3.56E-11 | 6.67E-10 |
| *IGKV1D-39* | 5.014656604 | 17.23922143 | 1.781471804 | 3.59E-11 | 6.72E-10 |
| *IGHV4-61* | 4.882339623 | 17.42221667 | 1.835283633 | 3.59E-11 | 6.72E-10 |
| *MUC16* | 1.71141761 | 4.797964286 | 1.487230578 | 3.63E-11 | 6.80E-10 |
| *IGKV3D-11* | 3.968866667 | 12.41608571 | 1.645411421 | 3.74E-11 | 6.98E-10 |
| *FMO5* | 3.969595597 | 1.688013095 | -1.233665944 | 3.78E-11 | 7.05E-10 |
| *IGHV3-43* | 11.08223774 | 38.948075 | 1.813302804 | 3.81E-11 | 7.11E-10 |
| *AC107884.2* | 0.521669182 | 0.119861905 | -2.121762003 | 3.83E-11 | 7.13E-10 |
| *ADAD2* | 0.745078302 | 0.321527381 | -1.212450448 | 4.00E-11 | 7.43E-10 |
| *WASIR2* | 2.619568239 | 1.074596429 | -1.285534096 | 4.18E-11 | 7.76E-10 |
| *LINC00582* | 0.372394654 | 0.917428571 | 1.300763475 | 4.35E-11 | 8.05E-10 |
| *IGHV3-62* | 1.258039623 | 3.176239286 | 1.336142242 | 4.40E-11 | 8.14E-10 |
| *PRR36* | 10.88112987 | 4.612217857 | -1.238295806 | 4.45E-11 | 8.22E-10 |
| *TGFBR3* | 17.22738019 | 8.162869048 | -1.077555106 | 4.47E-11 | 8.25E-10 |
| *THRB-AS1* | 0.216117925 | 0.084471429 | -1.355283377 | 4.49E-11 | 8.29E-10 |
| *KRTAP5-9* | 7.045584277 | 1.83549881 | -1.940547173 | 4.52E-11 | 8.34E-10 |
| *AC100860.1* | 2.624660063 | 0.620333333 | -2.081015027 | 4.54E-11 | 8.37E-10 |
| *MT2P1* | 6.657131761 | 20.98421071 | 1.656331572 | 4.72E-11 | 8.67E-10 |
| *AL022326.2* | 1.44169434 | 0.52729881 | -1.45107268 | 4.80E-11 | 8.81E-10 |
| *POF1B* | 36.01518019 | 14.70319881 | -1.292475062 | 4.83E-11 | 8.86E-10 |
| *GRM3-AS1* | 0.220994969 | 0.066511905 | -1.732329031 | 4.95E-11 | 9.07E-10 |
| *AC010329.1* | 5.424027358 | 1.76934881 | -1.616145966 | 5.01E-11 | 9.17E-10 |
| *AC040174.1* | 0.454880818 | 0.114227381 | -1.993580083 | 5.09E-11 | 9.31E-10 |
| *PSCA* | 2080.432449 | 575.9793536 | -1.852794442 | 5.15E-11 | 9.41E-10 |
| *KCNE1* | 0.116640881 | 0.277295238 | 1.249349326 | 5.18E-11 | 9.47E-10 |
| *IGHV4-55* | 5.018418868 | 16.01448095 | 1.674072242 | 5.59E-11 | 1.02E-09 |
| *MSX2* | 55.35076321 | 27.48575952 | -1.009918863 | 5.72E-11 | 1.04E-09 |
| *HMGB2P1* | 0.266303774 | 1.081170238 | 2.021448927 | 5.75E-11 | 1.04E-09 |
| *AP002026.1* | 3.545733019 | 1.404715476 | -1.335805967 | 5.83E-11 | 1.06E-09 |
| *VSTM1* | 0.170828616 | 0.739494048 | 2.113988867 | 5.90E-11 | 1.07E-09 |

| *GPRC5C* | 43.50189245 | 19.21589762 | -1.178777794 | 6.10E-11 | 1.10E-09 |
| --- | --- | --- | --- | --- | --- |
| *IGHV3-20* | 8.397860692 | 44.23408095 | 2.397064587 | 6.11E-11 | 1.10E-09 |
| *MICB-DT* | 1.110605031 | 2.27132381 | 1.03218756 | 6.14E-11 | 1.11E-09 |
| *UBE2FP3* | 3.823661006 | 1.828164286 | -1.064558902 | 6.19E-11 | 1.12E-09 |
| *AL135818.2* | 1.476516038 | 3.068615476 | 1.055390848 | 6.20E-11 | 1.12E-09 |
| *IGKJ4* | 7.332662264 | 18.61704405 | 1.344215028 | 6.28E-11 | 1.13E-09 |
| *RGS20* | 2.971612579 | 6.710613095 | 1.175198542 | 6.32E-11 | 1.14E-09 |
| *RHBG* | 14.0180717 | 3.584625 | -1.967393802 | 6.39E-11 | 1.15E-09 |
| *TBX2* | 73.82900849 | 24.53313929 | -1.589455929 | 6.48E-11 | 1.16E-09 |
| *MELTF* | 9.393653145 | 32.66072262 | 1.797798482 | 6.55E-11 | 1.17E-09 |
| *TTYH1* | 1.299267296 | 0.472384524 | -1.459664659 | 6.57E-11 | 1.18E-09 |
| *SLITRK6* | 64.05748522 | 30.14954643 | -1.087230863 | 6.73E-11 | 1.20E-09 |
| *IGHG4* | 153.9099066 | 616.2726512 | 2.001482673 | 6.73E-11 | 1.20E-09 |
| *EXOC3L4* | 2.04152956 | 6.984157143 | 1.774435563 | 6.78E-11 | 1.21E-09 |
| *FMO1* | 1.696152201 | 3.659935714 | 1.109552675 | 7.02E-11 | 1.25E-09 |
| *RN7SKP23* | 0.317353774 | 0.117703571 | -1.430933901 | 7.14E-11 | 1.27E-09 |
| *OR13A1* | 2.485473585 | 0.97460119 | -1.350636881 | 7.30E-11 | 1.29E-09 |
| *AP001205.1* | 1.134004717 | 0.283838095 | -1.998286503 | 7.34E-11 | 1.30E-09 |
| *AC008738.2* | 1.058967925 | 0.507580952 | -1.060949055 | 7.36E-11 | 1.30E-09 |
| *GRIP2* | 0.28196478 | 0.599482143 | 1.088201813 | 7.51E-11 | 1.33E-09 |
| *ZNF888* | 32.43909308 | 14.86124048 | -1.126178942 | 7.75E-11 | 1.37E-09 |
| *SLC30A2* | 19.33199182 | 4.868358333 | -1.989483024 | 7.88E-11 | 1.39E-09 |
| *AC005009.1* | 1.720183962 | 0.352883333 | -2.285299661 | 8.19E-11 | 1.45E-09 |
| *AC008752.3* | 5.652985535 | 2.645038095 | -1.095724504 | 8.23E-11 | 1.45E-09 |
| *IGKV2-28* | 2.104057862 | 10.16861548 | 2.272876976 | 8.49E-11 | 1.49E-09 |
| *SCNN1B* | 89.67089465 | 31.36759405 | -1.515364916 | 8.58E-11 | 1.51E-09 |
| *AL450384.2* | 5.840754717 | 2.113739286 | -1.466357358 | 8.61E-11 | 1.51E-09 |
| *PPFIBP2* | 28.51397138 | 14.10210595 | -1.015758365 | 8.92E-11 | 1.56E-09 |
| *AC008679.1* | 0.505066981 | 0.168371429 | -1.584827384 | 8.96E-11 | 1.57E-09 |
| *ENPP7P7* | 0.249272956 | 0.048227381 | -2.369802001 | 9.01E-11 | 1.58E-09 |
| *KRTAP5-8* | 1.786082704 | 0.318820238 | -2.485983769 | 9.32E-11 | 1.63E-09 |
| *AL161804.1* | 0.345830189 | 0.078332143 | -2.142387482 | 9.34E-11 | 1.63E-09 |
| *THRB-IT1* | 0.785678616 | 0.147245238 | -2.415718317 | 9.79E-11 | 1.71E-09 |
| *APOC1* | 38.0641783 | 82.61062738 | 1.117893453 | 9.84E-11 | 1.71E-09 |
| *GRM3* | 1.307936164 | 0.085142857 | -3.941262815 | 1.03E-10 | 1.80E-09 |
| *TLE2* | 49.04183742 | 22.11045238 | -1.14928449 | 1.04E-10 | 1.80E-09 |
| *IGHV1-58* | 7.692207862 | 18.1051619 | 1.234931425 | 1.05E-10 | 1.81E-09 |
| *IGKV1OR2-108* | 4.708106289 | 10.67824048 | 1.181455148 | 1.09E-10 | 1.89E-09 |
| *PLA2G4C* | 1.568079245 | 3.30612381 | 1.076142282 | 1.11E-10 | 1.92E-09 |
| *CYP4B1* | 193.6012349 | 88.21519167 | -1.133989124 | 1.12E-10 | 1.94E-09 |
| *AL035661.1* | 60.58270818 | 29.44847262 | -1.040713262 | 1.13E-10 | 1.95E-09 |
| *FCAR* | 0.219487736 | 0.628833333 | 1.518537365 | 1.14E-10 | 1.97E-09 |
| *NDRG2* | 63.36030818 | 28.10407381 | -1.172800082 | 1.16E-10 | 2.00E-09 |
| *PNCK* | 20.63912327 | 3.620065476 | -2.511293991 | 1.19E-10 | 2.04E-09 |
| *AC138305.1* | 0.45707327 | 0.133619048 | -1.774299772 | 1.21E-10 | 2.07E-09 |
| *KLHDC7A* | 24.30388931 | 11.4019631 | -1.091904967 | 1.25E-10 | 2.14E-09 |

| *TRBV11-1* | 0.114315723 | 0.498716667 | 2.125196568 | 1.26E-10 | 2.15E-09 |
| --- | --- | --- | --- | --- | --- |
| *PTCHD1* | 2.491625786 | 1.083479762 | -1.201415202 | 1.29E-10 | 2.20E-09 |
| *SIGLEC15* | 10.02751226 | 2.669167857 | -1.90950179 | 1.32E-10 | 2.24E-09 |
| *AP000942.5* | 0.13202956 | 0.580166667 | 2.135606441 | 1.33E-10 | 2.27E-09 |
| *AC110774.1* | 1.006891824 | 0.27922619 | -1.850402522 | 1.36E-10 | 2.31E-09 |
| *AC108112.1* | 2.613941824 | 0.914665476 | -1.51491093 | 1.39E-10 | 2.36E-09 |
| *CRTAC1* | 52.46444843 | 2.811070238 | -4.222148731 | 1.41E-10 | 2.39E-09 |
| *SAMD12-AS1* | 0.709127673 | 0.274382143 | -1.369858805 | 1.43E-10 | 2.42E-09 |
| *IGKV1-9* | 52.0957544 | 239.4662571 | 2.200584674 | 1.46E-10 | 2.47E-09 |
| *GPD1L* | 52.31480597 | 23.48225 | -1.155648661 | 1.48E-10 | 2.50E-09 |
| *Z97192.1* | 0.43232673 | 0.076678571 | -2.495226673 | 1.50E-10 | 2.54E-09 |
| *TM7SF2* | 47.58679277 | 22.86342738 | -1.057519534 | 1.51E-10 | 2.55E-09 |
| *CYP4F22* | 50.94315755 | 11.17031429 | -2.189218605 | 1.56E-10 | 2.62E-09 |
| *GPX2* | 656.6050189 | 287.1322131 | -1.193310583 | 1.56E-10 | 2.62E-09 |
| *CYP2J2* | 41.86770094 | 14.52640833 | -1.527159659 | 1.57E-10 | 2.63E-09 |
| *IFNG-AS1* | 0.087656289 | 0.179379762 | 1.033087618 | 1.58E-10 | 2.66E-09 |
| *AC116914.1* | 0.273260377 | 0.614564286 | 1.169287645 | 1.61E-10 | 2.71E-09 |
| *WFDC13* | 1.168104717 | 0.242582143 | -2.267624354 | 1.64E-10 | 2.75E-09 |
| *DHRS2* | 472.5463733 | 185.8382988 | -1.346408062 | 1.73E-10 | 2.89E-09 |
| *MYZAP* | 8.843079874 | 4.068479762 | -1.120059106 | 1.74E-10 | 2.91E-09 |
| *CYP4F23P* | 21.40235849 | 7.612508333 | -1.49132598 | 1.75E-10 | 2.93E-09 |
| *AC010735.2* | 4.649432075 | 1.409021429 | -1.72236095 | 1.79E-10 | 2.99E-09 |
| *AP002954.1* | 0.407695597 | 0.954770238 | 1.227661218 | 1.80E-10 | 3.01E-09 |
| *IGKV3D-15* | 5.502455031 | 22.65064881 | 2.041405021 | 1.83E-10 | 3.04E-09 |
| *IGKV2-24* | 22.13917767 | 66.51605238 | 1.587100913 | 1.83E-10 | 3.04E-09 |
| *CIBAR2* | 0.335861006 | 0.708336905 | 1.076571402 | 1.87E-10 | 3.11E-09 |
| *Z73965.1* | 0.542142453 | 0.240115476 | -1.17494359 | 1.88E-10 | 3.12E-09 |
| *IGHV1-46* | 29.81847767 | 91.24973452 | 1.613613756 | 1.93E-10 | 3.19E-09 |
| *CDH26* | 11.38830692 | 4.741067857 | -1.264269333 | 1.99E-10 | 3.30E-09 |
| *AL139351.3* | 0.607192138 | 1.571263095 | 1.371699752 | 2.03E-10 | 3.36E-09 |
| *TMEM97* | 194.7390994 | 90.19778929 | -1.110378596 | 2.08E-10 | 3.44E-09 |
| *IGLV7-43* | 12.7763544 | 98.34165476 | 2.944326394 | 2.11E-10 | 3.48E-09 |
| *PSMC1P7* | 0.432439308 | 0.100990476 | -2.098278421 | 2.11E-10 | 3.49E-09 |
| *TNFAIP6* | 9.346969497 | 20.39004167 | 1.125294133 | 2.14E-10 | 3.52E-09 |
| *DACH2* | 0.772560692 | 0.169515476 | -2.188231282 | 2.27E-10 | 3.74E-09 |
| *TPPP2* | 0.293004088 | 0.088678571 | -1.724263359 | 2.28E-10 | 3.74E-09 |
| *ATBC* | 19.58885881 | 6.070045238 | -1.690254179 | 2.70E-10 | 4.40E-09 |
| *FAM3B* | 51.87313648 | 19.50159405 | -1.41139555 | 2.79E-10 | 4.54E-09 |
| *CRISP3* | 19.97207704 | 1.966969048 | -3.343938217 | 2.80E-10 | 4.55E-09 |
| *BMP5* | 2.300350943 | 0.44357619 | -2.374600143 | 2.86E-10 | 4.65E-09 |
| *LINC02574* | 0.37588522 | 0.812930952 | 1.11284063 | 2.90E-10 | 4.71E-09 |
| *LINC00173* | 0.375561006 | 0.819232143 | 1.125225042 | 2.91E-10 | 4.71E-09 |
| *IGHV2-26* | 19.05038994 | 83.75644643 | 2.136379706 | 3.00E-10 | 4.86E-09 |
| *SCUBE2* | 31.4368978 | 14.81412619 | -1.085485327 | 3.01E-10 | 4.87E-09 |
| *IGHV3OR16-9* | 1.042259748 | 2.867625 | 1.460141509 | 3.02E-10 | 4.88E-09 |
| *AL359715.1* | 1.380119811 | 0.491434524 | -1.489722399 | 3.09E-10 | 4.99E-09 |

| *AC108134.1* | 3.674037107 | 1.644885714 | -1.159378848 | 3.12E-10 | 5.03E-09 |
| --- | --- | --- | --- | --- | --- |
| *AL138720.1* | 0.24135283 | 0.548620238 | 1.184664098 | 3.13E-10 | 5.03E-09 |
| *ADAMTS19* | 0.660720126 | 0.205659524 | -1.683781408 | 3.13E-10 | 5.03E-09 |
| *AC142381.1* | 1.33288805 | 2.91212619 | 1.12751726 | 3.15E-10 | 5.06E-09 |
| *SPTSSB* | 82.23150786 | 36.35063571 | -1.177710689 | 3.15E-10 | 5.07E-09 |
| *EHD4-AS1* | 1.353168868 | 0.575155952 | -1.234316793 | 3.21E-10 | 5.16E-09 |
| *IGHV3OR16-8* | 0.746448113 | 2.012475 | 1.430856977 | 3.33E-10 | 5.33E-09 |
| *IGLV1-36* | 15.29208082 | 50.85078452 | 1.733485302 | 3.35E-10 | 5.36E-09 |
| *MCCC1-AS1* | 2.383489623 | 1.127525 | -1.079915922 | 3.40E-10 | 5.43E-09 |
| *RHOU* | 70.6310478 | 33.53418095 | -1.074670133 | 3.42E-10 | 5.46E-09 |
| *AC079015.1* | 0.221651887 | 0.666008333 | 1.587244586 | 3.44E-10 | 5.48E-09 |
| *IGKV3-7* | 2.863943711 | 13.20779048 | 2.205314097 | 3.45E-10 | 5.50E-09 |
| *SLC35F4* | 0.154322013 | 0.033942857 | -2.184763949 | 3.46E-10 | 5.51E-09 |
| *Z99572.1* | 1.595307233 | 0.750741667 | -1.087445832 | 3.52E-10 | 5.59E-09 |
| *IGSF5* | 0.660127987 | 0.156254762 | -2.078845608 | 3.81E-10 | 6.04E-09 |
| *AC034105.1* | 0.503969497 | 1.518280952 | 1.59103046 | 3.86E-10 | 6.12E-09 |
| *GATA3* | 281.1534283 | 131.8485714 | -1.092475699 | 3.92E-10 | 6.20E-09 |
| *GPR55* | 0.231045912 | 0.470416667 | 1.025759612 | 3.93E-10 | 6.22E-09 |
| *IL22RA2* | 0.419751572 | 1.288092857 | 1.617628965 | 3.95E-10 | 6.24E-09 |
| *MIR4538* | 0.687925157 | 1.496722619 | 1.121483357 | 3.99E-10 | 6.30E-09 |
| *AC010300.1* | 1.22757673 | 0.337267857 | -1.863846467 | 4.01E-10 | 6.33E-09 |
| *UGT1A1* | 5.668198742 | 1.5783 | -1.844518888 | 4.10E-10 | 6.47E-09 |
| *IGHV3-60* | 0.478783648 | 1.24735 | 1.38142055 | 4.13E-10 | 6.51E-09 |
| *BAIAP3* | 8.434167296 | 3.511557143 | -1.264134726 | 4.16E-10 | 6.54E-09 |
| *IGHV3OR16-13* | 1.022719811 | 2.726097619 | 1.414426272 | 4.26E-10 | 6.69E-09 |
| *TMEM178A* | 3.430945283 | 0.933554762 | -1.87779956 | 4.35E-10 | 6.83E-09 |
| *OR7E91P* | 12.46356478 | 5.703552381 | -1.127784094 | 4.38E-10 | 6.86E-09 |
| *BAMBI* | 122.3897022 | 54.48633452 | -1.167515831 | 4.49E-10 | 7.03E-09 |
| *AC011487.2* | 49.11689025 | 7.466478571 | -2.717719334 | 4.53E-10 | 7.09E-09 |
| *SLC6A4* | 0.876711635 | 0.368214286 | -1.251556794 | 4.67E-10 | 7.29E-09 |
| *TNF* | 2.812698428 | 7.946485714 | 1.4983621 | 4.77E-10 | 7.43E-09 |
| *AC068700.1* | 0.993346855 | 0.297560714 | -1.739113498 | 4.85E-10 | 7.55E-09 |
| *CDH23* | 4.159055031 | 1.349821429 | -1.623487212 | 4.95E-10 | 7.70E-09 |
| *IGKV1OR2-3* | 0.230508491 | 0.759180952 | 1.719623904 | 4.97E-10 | 7.73E-09 |
| *TREML1* | 0.621403774 | 1.929261905 | 1.6344461 | 5.01E-10 | 7.79E-09 |
| *AC011416.4* | 0.130905975 | 0.062677381 | -1.062514145 | 5.21E-10 | 8.08E-09 |
| *IGKV1OR9-2* | 0.129916981 | 0.476634524 | 1.875293439 | 5.25E-10 | 8.13E-09 |
| *STK32A-AS1* | 3.479868239 | 0.437717857 | -2.990959535 | 5.26E-10 | 8.14E-09 |
| *MIR429* | 25.04163994 | 10.26162976 | -1.287069166 | 5.34E-10 | 8.26E-09 |
| *C9orf152* | 4.024231132 | 1.670696429 | -1.268263554 | 5.67E-10 | 8.74E-09 |
| *UGT2B28* | 1.203318868 | 0.296896429 | -2.018987349 | 5.70E-10 | 8.78E-09 |
| *IGLV3-27* | 23.10143994 | 51.56429643 | 1.158389697 | 5.70E-10 | 8.78E-09 |
| *PPP1R3C* | 39.78834025 | 17.85307976 | -1.15617275 | 5.71E-10 | 8.78E-09 |
| *ANKRD29* | 1.118053459 | 3.043052381 | 1.444529996 | 5.73E-10 | 8.81E-09 |
| *CYP3A5* | 11.77660692 | 3.702634524 | -1.669299872 | 5.83E-10 | 8.95E-09 |
| *RIMKLA* | 1.634733019 | 0.606166667 | -1.431268612 | 5.89E-10 | 9.04E-09 |

| *LINC01926* | 0.100014465 | 0.32334881 | 1.692882622 | 5.90E-10 | 9.05E-09 |
| --- | --- | --- | --- | --- | --- |
| *TNNC1* | 29.59450031 | 10.3111869 | -1.521118689 | 5.91E-10 | 9.05E-09 |
| *ADH6* | 3.064708491 | 0.57050119 | -2.425448052 | 5.93E-10 | 9.07E-09 |
| *AC073046.1* | 7.673631132 | 3.466446429 | -1.146451952 | 5.99E-10 | 9.15E-09 |
| *TRHDE-AS1* | 3.269720126 | 0.549789286 | -2.572216455 | 6.05E-10 | 9.24E-09 |
| *SLC23A2* | 29.33730314 | 14.50611667 | -1.016074899 | 6.32E-10 | 9.62E-09 |
| *PLIN5* | 7.832617296 | 2.902580952 | -1.432158165 | 6.36E-10 | 9.67E-09 |
| *IFNL2* | 0.10177327 | 0.374210714 | 1.878492162 | 6.62E-10 | 1.00E-08 |
| *SNX19P4* | 0.936571069 | 0.30684881 | -1.609860487 | 6.63E-10 | 1.00E-08 |
| *AC134879.2* | 0.715928302 | 1.71892381 | 1.263618581 | 6.73E-10 | 1.02E-08 |
| *PWWP3B* | 3.675451572 | 1.028679762 | -1.837127586 | 6.88E-10 | 1.04E-08 |
| *MMP12* | 56.61867484 | 153.235131 | 1.436397201 | 6.89E-10 | 1.04E-08 |
| *LINC00092* | 0.187515409 | 0.39264881 | 1.066230371 | 6.91E-10 | 1.04E-08 |
| *IGHV1-69* | 14.06463553 | 66.39855476 | 2.239079672 | 6.93E-10 | 1.05E-08 |
| *GGT6* | 78.18643836 | 35.03564881 | -1.158094777 | 6.96E-10 | 1.05E-08 |
| *OGFRL1* | 5.248291195 | 12.1732381 | 1.213793304 | 6.98E-10 | 1.05E-08 |
| *AL137186.1* | 0.206704717 | 0.587842857 | 1.507859232 | 7.05E-10 | 1.06E-08 |
| *KRTAP5-10* | 3.38796195 | 0.629788095 | -2.42747928 | 7.10E-10 | 1.07E-08 |
| *MROH2A* | 8.126742138 | 0.499410714 | -4.02437844 | 7.13E-10 | 1.07E-08 |
| *TRIM15* | 0.313538994 | 1.599685714 | 2.351071709 | 7.19E-10 | 1.08E-08 |
| *COL9A1* | 1.147821698 | 0.152940476 | -2.907856376 | 7.23E-10 | 1.09E-08 |
| *AL356234.2* | 0.121324843 | 0.506565476 | 2.061873766 | 7.46E-10 | 1.12E-08 |
| *CPAMD8* | 5.636098742 | 2.085338095 | -1.434415581 | 7.49E-10 | 1.12E-08 |
| *VCAM1* | 11.29394717 | 28.86965 | 1.354003829 | 7.72E-10 | 1.15E-08 |
| *AC012511.2* | 0.886138365 | 0.118409524 | -2.903746861 | 7.84E-10 | 1.17E-08 |
| *AC079848.2* | 1.356800943 | 0.45792619 | -1.567022093 | 8.11E-10 | 1.20E-08 |
| *CABP1* | 0.867005346 | 0.274915476 | -1.657052764 | 8.11E-10 | 1.20E-08 |
| *AL031705.1* | 1.122012893 | 0.511225 | -1.13405896 | 8.42E-10 | 1.25E-08 |
| *TRBV10-1* | 0.06112327 | 0.339832143 | 2.475028673 | 8.44E-10 | 1.25E-08 |
| *KRT33A* | 10.31667736 | 0.621107143 | -4.053992434 | 8.49E-10 | 1.26E-08 |
| *ID4* | 36.13957296 | 10.2716631 | -1.81490967 | 8.53E-10 | 1.26E-08 |
| *PPARG* | 102.2682321 | 49.80674881 | -1.03794492 | 8.53E-10 | 1.26E-08 |
| *IGHV1-3* | 53.18475189 | 114.1709321 | 1.1021108 | 8.60E-10 | 1.27E-08 |
| *LINC00967* | 11.79128711 | 0.745154762 | -3.984037306 | 8.82E-10 | 1.30E-08 |
| *AF131216.3* | 2.016175472 | 0.703771429 | -1.518442354 | 8.88E-10 | 1.31E-08 |
| *AC136428.1* | 0.703997484 | 2.185655952 | 1.634424143 | 8.90E-10 | 1.31E-08 |
| *IFNL3* | 0.156253774 | 0.460469048 | 1.559213151 | 9.07E-10 | 1.33E-08 |
| *LINC02273* | 0.272752201 | 0.562964286 | 1.045452561 | 9.08E-10 | 1.34E-08 |
| *IGHV3-74* | 25.9375434 | 114.3350702 | 2.140154242 | 9.30E-10 | 1.36E-08 |
| *AC027348.1* | 2.396366038 | 0.819078571 | -1.548774536 | 9.72E-10 | 1.42E-08 |
| *FRY-AS1* | 0.391322642 | 0.155332143 | -1.333002188 | 9.92E-10 | 1.45E-08 |
| *RSPO3* | 1.159263836 | 3.023438095 | 1.382981094 | 1.02E-09 | 1.48E-08 |
| *ZNF536* | 0.336310377 | 0.120564286 | -1.479990689 | 1.04E-09 | 1.51E-08 |
| *IGLV2-18* | 7.191508491 | 46.99202619 | 2.708049648 | 1.04E-09 | 1.51E-08 |
| *ACOXL* | 6.430702201 | 2.879209524 | -1.159303502 | 1.05E-09 | 1.52E-08 |
| *TBX3* | 105.3473553 | 45.40266429 | -1.214305233 | 1.05E-09 | 1.52E-08 |

| *IGKV2D-29* | 14.96355252 | 50.75417381 | 1.76207374 | 1.06E-09 | 1.53E-08 |
| --- | --- | --- | --- | --- | --- |
| *ATP6V0CP2* | 3.855983648 | 1.833720238 | -1.072325383 | 1.10E-09 | 1.59E-08 |
| *IGKV1-13* | 0.759739623 | 2.064557143 | 1.442255381 | 1.16E-09 | 1.67E-08 |
| *KLF3-AS1* | 1.326925786 | 0.613733333 | -1.112403837 | 1.17E-09 | 1.68E-08 |
| *AL390719.2* | 27.78287013 | 13.10681905 | -1.083878052 | 1.17E-09 | 1.69E-08 |
| *C10orf99* | 251.7170664 | 74.84432143 | -1.749838268 | 1.17E-09 | 1.69E-08 |
| *AP001094.2* | 1.143639937 | 0.316225 | -1.854609573 | 1.20E-09 | 1.72E-08 |
| *AL645608.2* | 0.729922642 | 0.255155952 | -1.516364275 | 1.21E-09 | 1.74E-08 |
| *FXYD4* | 68.00904874 | 16.92353452 | -2.006695802 | 1.21E-09 | 1.74E-08 |
| *NRXN3* | 2.156144969 | 0.775502381 | -1.475251065 | 1.27E-09 | 1.81E-08 |
| *SEMA5A* | 30.20130472 | 11.72337857 | -1.365222475 | 1.28E-09 | 1.84E-08 |
| *SAMD13* | 3.108772013 | 1.355603571 | -1.197409475 | 1.29E-09 | 1.84E-08 |
| *B3GALT5* | 3.21799434 | 0.975508333 | -1.721935686 | 1.34E-09 | 1.91E-08 |
| *LINC02562* | 25.57116698 | 11.63576667 | -1.13595173 | 1.37E-09 | 1.94E-08 |
| *BMP3* | 25.46529748 | 10.77143214 | -1.241322491 | 1.39E-09 | 1.97E-08 |
| *G0S2* | 54.35905566 | 189.8361 | 1.804162069 | 1.42E-09 | 2.01E-08 |
| *APOBEC3A* | 6.599409748 | 22.670575 | 1.780412082 | 1.43E-09 | 2.02E-08 |
| *IGHV2-70* | 14.17785849 | 80.14354762 | 2.498946738 | 1.45E-09 | 2.05E-08 |
| *MTUS2* | 3.000286792 | 1.066214286 | -1.492602995 | 1.48E-09 | 2.09E-08 |
| *SGK2* | 7.237872956 | 2.076220238 | -1.801606297 | 1.48E-09 | 2.09E-08 |
| *AC006042.1* | 9.376087736 | 4.334796429 | -1.113021827 | 1.58E-09 | 2.22E-08 |
| *DEGS2* | 19.79683585 | 7.545465476 | -1.391588053 | 1.61E-09 | 2.25E-08 |
| *AC092279.2* | 1.880346541 | 0.700994048 | -1.423524471 | 1.61E-09 | 2.26E-08 |
| *SMAD6* | 6.54201478 | 2.453520238 | -1.414881847 | 1.62E-09 | 2.26E-08 |
| *PLA2G4F* | 12.36169497 | 5.710902381 | -1.114085942 | 1.62E-09 | 2.27E-08 |
| *VWA3B* | 0.091925786 | 0.213125 | 1.213158319 | 1.64E-09 | 2.29E-08 |
| *AL022322.1* | 4.625189308 | 2.031339286 | -1.187081189 | 1.69E-09 | 2.35E-08 |
| *PRRX1* | 9.586087107 | 22.36691548 | 1.22235236 | 1.69E-09 | 2.36E-08 |
| *IGLV7-46* | 16.65773805 | 43.52722619 | 1.385725575 | 1.74E-09 | 2.42E-08 |
| *AKR1C2* | 130.4989182 | 64.66497262 | -1.01298149 | 1.79E-09 | 2.50E-08 |
| *AC008759.2* | 2.722520755 | 0.993290476 | -1.454655463 | 1.82E-09 | 2.53E-08 |
| *AL358075.2* | 3.475331761 | 1.575266667 | -1.141554633 | 1.82E-09 | 2.53E-08 |
| *HDHD5-AS1* | 1.026251572 | 0.439620238 | -1.223054724 | 1.85E-09 | 2.56E-08 |
| *PLAC8* | 6.13205 | 17.54656786 | 1.516747498 | 1.87E-09 | 2.58E-08 |
| *ST3GAL4* | 58.23464497 | 24.85305595 | -1.228454439 | 1.94E-09 | 2.68E-08 |
| *SRRM3* | 8.584679245 | 3.849914286 | -1.156937905 | 1.96E-09 | 2.70E-08 |
| *FSTL4* | 8.084501572 | 3.95397619 | -1.031854647 | 2.02E-09 | 2.77E-08 |
| *RPL23AP85* | 0.18242956 | 0.042159524 | -2.113409037 | 2.04E-09 | 2.80E-08 |
| *CYP4F2* | 2.739358491 | 0.268716667 | -3.349680369 | 2.07E-09 | 2.84E-08 |
| *AL645608.6* | 2.373801572 | 0.65255119 | -1.863036358 | 2.11E-09 | 2.88E-08 |
| *FER1L4* | 86.28163994 | 29.33428333 | -1.556465852 | 2.12E-09 | 2.91E-08 |
| *IGLV8-61* | 17.31090189 | 60.91300952 | 1.815069495 | 2.13E-09 | 2.92E-08 |
| *MIR29B2CHG* | 2.092891509 | 0.469695238 | -2.155700655 | 2.15E-09 | 2.94E-08 |
| *COL23A1* | 1.505368553 | 3.059971429 | 1.023401443 | 2.20E-09 | 3.00E-08 |
| *B3GALT5-AS1* | 1.703306289 | 0.465925 | -1.870168237 | 2.20E-09 | 3.00E-08 |
| *AL121772.3* | 6.462419182 | 3.226007143 | -1.002324701 | 2.22E-09 | 3.02E-08 |

| *TMC7* | 9.700262264 | 4.463513095 | -1.119844095 | 2.32E-09 | 3.15E-08 |
| --- | --- | --- | --- | --- | --- |
| *SNHG18* | 67.31948176 | 30.49665357 | -1.142373128 | 2.36E-09 | 3.19E-08 |
| *IGKV1D-8* | 3.36469434 | 11.4056131 | 1.761196643 | 2.37E-09 | 3.21E-08 |
| *CLEC2B* | 24.22835094 | 50.89124524 | 1.070721279 | 2.37E-09 | 3.21E-08 |
| *AC010329.2* | 2.127378931 | 0.759967857 | -1.485066724 | 2.38E-09 | 3.22E-08 |
| *IGHJ5* | 4.616759748 | 10.62453333 | 1.202446911 | 2.39E-09 | 3.24E-08 |
| *RAPGEFL1* | 81.59198491 | 39.98321429 | -1.029032981 | 2.40E-09 | 3.25E-08 |
| *IGHJ2* | 9.356010692 | 29.83533929 | 1.673056768 | 2.44E-09 | 3.29E-08 |
| *DLEC1* | 0.634933333 | 0.30812381 | -1.043094952 | 2.47E-09 | 3.32E-08 |
| *SPIB* | 2.390792453 | 27.54015238 | 3.525975743 | 2.48E-09 | 3.34E-08 |
| *ANXA3* | 18.21139969 | 37.61206071 | 1.046353543 | 2.49E-09 | 3.35E-08 |
| *CACNA2D2* | 1.617205346 | 0.621211905 | -1.380345495 | 2.50E-09 | 3.36E-08 |
| *TMEM45B* | 52.73387453 | 24.89420952 | -1.082919794 | 2.51E-09 | 3.38E-08 |
| *CRH* | 157.3586736 | 9.412820238 | -4.063285848 | 2.53E-09 | 3.39E-08 |
| *LINC00973* | 0.812186478 | 5.69127619 | 2.80886928 | 2.56E-09 | 3.44E-08 |
| *AL359715.2* | 1.451860377 | 0.531959524 | -1.448514337 | 2.63E-09 | 3.52E-08 |
| *ZNF737* | 15.18296792 | 6.174754762 | -1.298000088 | 2.66E-09 | 3.56E-08 |
| *IGLV2-34* | 0.741619497 | 2.994575 | 2.013600188 | 2.69E-09 | 3.60E-08 |
| *AC010329.5* | 2.056705975 | 0.55097619 | -1.90027368 | 2.79E-09 | 3.71E-08 |
| *CEACAM3* | 0.234713836 | 0.67697381 | 1.528197129 | 2.80E-09 | 3.72E-08 |
| *MIR4537* | 0.750135535 | 1.611270238 | 1.102975289 | 2.87E-09 | 3.81E-08 |
| *G6PC* | 0.375117296 | 0.123171429 | -1.606674143 | 2.92E-09 | 3.87E-08 |
| *EMX2OS* | 2.822504088 | 1.102503571 | -1.356192341 | 3.21E-09 | 4.25E-08 |
| *AC073534.2* | 2.350739623 | 0.851120238 | -1.465679888 | 3.29E-09 | 4.34E-08 |
| *PDZD3* | 1.643781447 | 0.448230952 | -1.874704312 | 3.29E-09 | 4.34E-08 |
| *PDZK1IP1* | 72.2209217 | 167.2811679 | 1.211786302 | 3.32E-09 | 4.37E-08 |
| *SNORD69* | 8.943943082 | 3.957436905 | -1.176344658 | 3.35E-09 | 4.42E-08 |
| *AL391427.1* | 5.260727673 | 2.397577381 | -1.133684991 | 3.43E-09 | 4.51E-08 |
| *ZNF711* | 7.082922956 | 3.405102381 | -1.056646673 | 3.47E-09 | 4.56E-08 |
| *AL162274.1* | 0.447807233 | 0.167408333 | -1.419506485 | 3.50E-09 | 4.60E-08 |
| *GSTM2* | 11.22783239 | 3.557804762 | -1.658020184 | 3.51E-09 | 4.61E-08 |
| *AC026167.1* | 2.137763522 | 0.437297619 | -2.289414873 | 3.63E-09 | 4.75E-08 |
| *B3GAT1-DT* | 2.451925472 | 0.624614286 | -1.972877657 | 3.69E-09 | 4.83E-08 |
| *IGLC6* | 1.597272642 | 6.03010119 | 1.916571622 | 3.76E-09 | 4.91E-08 |
| *AC019117.1* | 11.00953742 | 3.019294048 | -1.866470681 | 3.82E-09 | 4.98E-08 |
| *IPO5P1* | 14.23501069 | 6.939404762 | -1.036559753 | 3.89E-09 | 5.07E-08 |
| *SCAT1* | 0.759302201 | 1.671334524 | 1.138254427 | 3.91E-09 | 5.09E-08 |
| *AC120498.4* | 17.1131066 | 5.396245238 | -1.665073862 | 4.01E-09 | 5.21E-08 |
| *AC084759.3* | 1.746659434 | 0.232864286 | -2.907037041 | 4.01E-09 | 5.21E-08 |
| *EPS8L3* | 6.079170126 | 2.969672619 | -1.033570498 | 4.17E-09 | 5.41E-08 |
| *AMPD1* | 0.096258176 | 0.226359524 | 1.233635015 | 4.22E-09 | 5.46E-08 |
| *ROBO2* | 0.776806918 | 0.249394048 | -1.639129017 | 4.24E-09 | 5.48E-08 |
| *IGHV1OR15-9* | 1.330962893 | 3.149211905 | 1.242520487 | 4.26E-09 | 5.50E-08 |
| *AC005336.3* | 0.629927987 | 0.094622619 | -2.734929912 | 4.38E-09 | 5.64E-08 |
| *AL121601.1* | 2.771733962 | 1.373433333 | -1.013001907 | 4.58E-09 | 5.88E-08 |
| *AC109479.1* | 0.276060692 | 0.667057143 | 1.272824876 | 4.64E-09 | 5.94E-08 |

| *PSME2P3* | 0.529144654 | 1.139017857 | 1.106056289 | 4.74E-09 | 6.06E-08 |
| --- | --- | --- | --- | --- | --- |
| *IL12A* | 0.707360692 | 1.4245 | 1.009937666 | 4.74E-09 | 6.06E-08 |
| *AC010260.1* | 0.768394025 | 0.282804762 | -1.442039888 | 4.80E-09 | 6.13E-08 |
| *ITGAD* | 0.251328302 | 0.631111905 | 1.328322696 | 5.06E-09 | 6.45E-08 |
| *SV2C* | 0.300197799 | 0.139182143 | -1.108939274 | 5.09E-09 | 6.47E-08 |
| *OR5M11* | 0.448263836 | 0.12894881 | -1.797549661 | 5.12E-09 | 6.50E-08 |
| *PM20D1* | 96.45872516 | 33.2527 | -1.536440256 | 5.33E-09 | 6.75E-08 |
| *AC015563.1* | 0.275692767 | 0.073559524 | -1.906077374 | 5.59E-09 | 7.07E-08 |
| *AL023882.1* | 0.696671698 | 0.302853571 | -1.201858531 | 5.66E-09 | 7.15E-08 |
| *F3* | 63.72168899 | 214.8867226 | 1.753719932 | 5.73E-09 | 7.24E-08 |
| *ANKRD33B* | 1.197971384 | 3.262967857 | 1.445591329 | 5.84E-09 | 7.37E-08 |
| *HLA-W* | 0.777457233 | 1.65295119 | 1.088208903 | 6.05E-09 | 7.60E-08 |
| *IGKV2D-28* | 2.079851258 | 7.091438095 | 1.769597869 | 6.05E-09 | 7.60E-08 |
| *IFITM3P2* | 3.495469811 | 9.729559524 | 1.476888118 | 6.07E-09 | 7.62E-08 |
| *P2RY12* | 0.361441509 | 0.926930952 | 1.358699675 | 6.22E-09 | 7.80E-08 |
| *SLC45A3* | 17.22043491 | 8.528292857 | -1.013792693 | 6.22E-09 | 7.80E-08 |
| *IYD* | 0.688708805 | 0.246811905 | -1.480482138 | 6.32E-09 | 7.89E-08 |
| *CDH3* | 83.4631934 | 190.6067155 | 1.191386923 | 6.34E-09 | 7.92E-08 |
| *SLC38A11* | 1.099925157 | 0.323741667 | -1.764490399 | 6.40E-09 | 7.98E-08 |
| *AC133644.1* | 1.927223585 | 4.024959524 | 1.062450321 | 6.44E-09 | 8.03E-08 |
| *AC103718.1* | 0.793494654 | 2.259544048 | 1.509739273 | 6.54E-09 | 8.15E-08 |
| *AL391650.2* | 7.650703774 | 1.69060119 | -2.178056094 | 6.57E-09 | 8.18E-08 |
| *PKIA* | 30.67529686 | 12.24884524 | -1.32443156 | 6.59E-09 | 8.19E-08 |
| *IGHJ3P* | 6.19501478 | 13.15260476 | 1.086168912 | 6.70E-09 | 8.32E-08 |
| *TMEM171* | 4.566838365 | 10.04411071 | 1.137082202 | 6.80E-09 | 8.43E-08 |
| *ANAT* | 0.337064151 | 0.67964881 | 1.011766269 | 7.05E-09 | 8.73E-08 |
| *PHGR1* | 14.57469654 | 3.023540476 | -2.269155048 | 7.16E-09 | 8.85E-08 |
| *SNORD123* | 14.85215472 | 5.972465476 | -1.314273735 | 7.18E-09 | 8.87E-08 |
| *DPP4* | 1.736116667 | 3.638869048 | 1.067626234 | 7.26E-09 | 8.96E-08 |
| *PADI2* | 3.725000314 | 10.03726071 | 1.430053142 | 7.33E-09 | 9.03E-08 |
| *KCNC3* | 5.477979245 | 2.709553571 | -1.015588629 | 7.33E-09 | 9.03E-08 |
| *LINC02814* | 1.584849686 | 0.458305952 | -1.789963087 | 7.35E-09 | 9.04E-08 |
| *CYP4F12* | 25.81984465 | 10.932825 | -1.239814085 | 7.38E-09 | 9.07E-08 |
| *LINC02038* | 2.1296 | 0.703691667 | -1.597567143 | 7.51E-09 | 9.22E-08 |
| *L1CAM* | 5.700141824 | 26.42158333 | 2.212647204 | 7.59E-09 | 9.31E-08 |
| *ZBTB8B* | 0.740139308 | 0.298136905 | -1.311821869 | 7.61E-09 | 9.33E-08 |
| *AC009123.1* | 0.322013836 | 0.126883333 | -1.343620103 | 7.65E-09 | 9.37E-08 |
| *HLA-DPB2* | 1.841114151 | 4.365155952 | 1.245454119 | 7.66E-09 | 9.38E-08 |
| *C1QTNF7* | 1.286434277 | 0.441055952 | -1.54434416 | 7.68E-09 | 9.41E-08 |
| *KCNQ1* | 19.51967358 | 8.459278571 | -1.206322391 | 7.80E-09 | 9.54E-08 |
| *DGKK* | 0.366928931 | 0.036859524 | -3.315391322 | 7.81E-09 | 9.54E-08 |
| *SGSM1* | 1.895419497 | 0.614919048 | -1.624048782 | 7.85E-09 | 9.59E-08 |
| *LINC01357* | 0.187507862 | 0.446885714 | 1.252954842 | 7.96E-09 | 9.71E-08 |
| *AL356740.1* | 2.131083648 | 0.967617857 | -1.139077923 | 8.11E-09 | 9.88E-08 |
| *UGT1A6* | 3.705911006 | 1.258120238 | -1.55855843 | 8.14E-09 | 9.91E-08 |
| *IGHA1* | 861.6285585 | 1898.392357 | 1.139640224 | 8.15E-09 | 9.91E-08 |

| *PKD1L1* | 1.747323899 | 0.513654762 | -1.766276139 | 8.18E-09 | 9.94E-08 |
| --- | --- | --- | --- | --- | --- |
| *S* *A4* | 0.124082075 | 0.506055952 | 2.028002183 | 8.34E-09 | 1.01E-07 |
| *CNTN3* | 3.083617925 | 1.08662619 | -1.504768294 | 8.49E-09 | 1.03E-07 |
| *LRATD1* | 53.50010094 | 21.95975952 | -1.284679358 | 8.60E-09 | 1.04E-07 |
| *MIR3972* | 12.13443774 | 2.668703571 | -2.18489629 | 8.66E-09 | 1.05E-07 |
| *AC068707.1* | 0.45947327 | 0.170217857 | -1.432598542 | 8.75E-09 | 1.06E-07 |
| *AC092168.2* | 1.141868553 | 0.27327619 | -2.06296491 | 8.77E-09 | 1.06E-07 |
| *AL645939.4* | 0.574925472 | 1.743519048 | 1.60055527 | 8.84E-09 | 1.07E-07 |
| *RPS6KA6* | 3.33992327 | 1.547021429 | -1.110321779 | 8.84E-09 | 1.07E-07 |
| *IGLV1-50* | 0.843522013 | 2.623354762 | 1.636915297 | 9.00E-09 | 1.08E-07 |
| *AC095057.3* | 3.039057862 | 1.45955119 | -1.058099332 | 9.07E-09 | 1.09E-07 |
| *IGLV10-54* | 4.446966981 | 16.49475119 | 1.891113418 | 9.26E-09 | 1.11E-07 |
| *IGHV3OR16-11* | 0.198620126 | 0.675854762 | 1.766701436 | 9.32E-09 | 1.12E-07 |
| *C1DP5* | 0.281946855 | 0.589515476 | 1.064106436 | 9.42E-09 | 1.13E-07 |
| *AC129507.3* | 0.745027673 | 0.246783333 | -1.594049049 | 9.57E-09 | 1.15E-07 |
| *ADAMTS2* | 14.2284544 | 28.49324881 | 1.001841173 | 9.65E-09 | 1.16E-07 |
| *MAP1LC3BP1* | 0.395775786 | 0.076059524 | -2.379482538 | 9.67E-09 | 1.16E-07 |
| *MCF2L-AS1* | 10.73373553 | 5.040115476 | -1.090623554 | 9.73E-09 | 1.16E-07 |
| *AC040174.2* | 1.612627673 | 0.360464286 | -2.161485153 | 9.73E-09 | 1.16E-07 |
| *IGHV1-14* | 0.439696541 | 1.317866667 | 1.583624327 | 9.83E-09 | 1.17E-07 |
| *TRBV6-4* | 0.077220755 | 0.23885 | 1.629044317 | 9.88E-09 | 1.18E-07 |
| *AL391834.3* | 0.571858805 | 0.16480119 | -1.794932318 | 9.99E-09 | 1.19E-07 |
| *AL136296.1* | 1.654598428 | 0.588703571 | -1.490867831 | 1.03E-08 | 1.23E-07 |
| *HPGD* | 184.3956827 | 69.13205952 | -1.415378067 | 1.04E-08 | 1.23E-07 |
| *IGLV1-41* | 2.971494969 | 10.55925476 | 1.829247174 | 1.05E-08 | 1.24E-07 |
| *AL158175.1* | 4.11058239 | 1.518296429 | -1.436889324 | 1.09E-08 | 1.28E-07 |
| *NNMT* | 98.08241415 | 222.1726655 | 1.179614934 | 1.10E-08 | 1.30E-07 |
| *AL133467.1* | 0.413643711 | 0.842272619 | 1.02589862 | 1.13E-08 | 1.33E-07 |
| *AL161668.4* | 1.535309119 | 0.468916667 | -1.711125695 | 1.13E-08 | 1.33E-07 |
| *AC011008.2* | 0.682930818 | 0.16309881 | -2.065993186 | 1.14E-08 | 1.34E-07 |
| *RNF186* | 2.132073585 | 0.456922619 | -2.222235464 | 1.16E-08 | 1.36E-07 |
| *ZPLD2P* | 0.750925786 | 0.312361905 | -1.265451818 | 1.17E-08 | 1.37E-07 |
| *AC115282.2* | 0.264275786 | 0.111008333 | -1.251376264 | 1.18E-08 | 1.38E-07 |
| *WSCD2* | 4.978675157 | 1.97315 | -1.335261252 | 1.20E-08 | 1.40E-07 |
| *LINC02875* | 4.74622044 | 2.302885714 | -1.043336293 | 1.20E-08 | 1.40E-07 |
| *LINC01889* | 1.664342453 | 0.437092857 | -1.928940604 | 1.22E-08 | 1.43E-07 |
| *LINC01933* | 0.277182075 | 0.065396429 | -2.083550211 | 1.23E-08 | 1.43E-07 |
| *AC121493.1* | 0.902754717 | 0.419327381 | -1.106257016 | 1.24E-08 | 1.45E-07 |
| *IGKV1OR2-6* | 1.459370755 | 4.865382143 | 1.737206675 | 1.25E-08 | 1.45E-07 |
| *IGKV6-21* | 5.801664465 | 31.12705476 | 2.42363031 | 1.25E-08 | 1.45E-07 |
| *IFITM4P* | 0.598906604 | 2.087967857 | 1.801696558 | 1.28E-08 | 1.49E-07 |
| *AC090236.2* | 0.48038522 | 0.154529762 | -1.636307042 | 1.29E-08 | 1.50E-07 |
| *EIF5AP3* | 0.751258176 | 0.36929881 | -1.024520175 | 1.29E-08 | 1.50E-07 |
| *CHP2* | 23.30592516 | 5.996520238 | -1.958499326 | 1.30E-08 | 1.51E-07 |
| *TPSP2* | 14.2158695 | 3.471846429 | -2.033727303 | 1.31E-08 | 1.51E-07 |
| *ZNF66* | 3.89087956 | 1.761021429 | -1.143683859 | 1.35E-08 | 1.56E-07 |

| *FMO8P* | 3.106048113 | 0.256991667 | -3.595286694 | 1.36E-08 | 1.57E-07 |
| --- | --- | --- | --- | --- | --- |
| *SPAG17* | 2.305319811 | 0.887845238 | -1.376586781 | 1.37E-08 | 1.59E-07 |
| *AL390294.1* | 1.278750314 | 0.429814286 | -1.572949255 | 1.41E-08 | 1.62E-07 |
| *ENPP7P2* | 0.387725786 | 0.079259524 | -2.290380482 | 1.42E-08 | 1.63E-07 |
| *LAMA3* | 22.23683899 | 71.25574048 | 1.680054524 | 1.43E-08 | 1.65E-07 |
| *AC120036.4* | 0.78227044 | 0.384566667 | -1.024433732 | 1.46E-08 | 1.68E-07 |
| *AC114812.4* | 0.650722642 | 0.092610714 | -2.812791736 | 1.48E-08 | 1.70E-07 |
| *IGKV1-33* | 1.871293082 | 6.285414286 | 1.747972308 | 1.50E-08 | 1.72E-07 |
| *HRC* | 1.739347799 | 0.587121429 | -1.566815624 | 1.53E-08 | 1.75E-07 |
| *AC012307.1* | 6.838507547 | 0.609882143 | -3.487079122 | 1.54E-08 | 1.76E-07 |
| *AL008718.2* | 0.525220126 | 0.222914286 | -1.236433123 | 1.58E-08 | 1.80E-07 |
| *AL022316.1* | 0.68095566 | 1.91177619 | 1.489280871 | 1.59E-08 | 1.82E-07 |
| *AXL* | 25.37875126 | 60.85313333 | 1.261710465 | 1.62E-08 | 1.85E-07 |
| *LINC02633* | 1.119945283 | 0.424627381 | -1.399158941 | 1.63E-08 | 1.85E-07 |
| *AL138721.1* | 1.443463836 | 0.388336905 | -1.894154241 | 1.64E-08 | 1.86E-07 |
| *HOXB3* | 12.57519088 | 5.370225 | -1.227525859 | 1.64E-08 | 1.86E-07 |
| *AC004817.3* | 0.278310063 | 0.864440476 | 1.635073553 | 1.64E-08 | 1.87E-07 |
| *CSF2* | 3.279858176 | 11.5710369 | 1.818812815 | 1.65E-08 | 1.87E-07 |
| *FOLR3* | 0.394028616 | 2.228039286 | 2.499402357 | 1.66E-08 | 1.88E-07 |
| *CAPN13* | 7.437312893 | 3.705113095 | -1.005263888 | 1.70E-08 | 1.92E-07 |
| *Z82188.2* | 0.107484906 | 0.239117857 | 1.1535878 | 1.71E-08 | 1.93E-07 |
| *AC018755.3* | 1.578142453 | 0.754254762 | -1.065103633 | 1.75E-08 | 1.97E-07 |
| *AC010636.1* | 2.734367925 | 0.555658333 | -2.298937411 | 1.75E-08 | 1.98E-07 |
| *NKPD1* | 1.048830189 | 0.509421429 | -1.041849564 | 1.77E-08 | 1.99E-07 |
| *AC073534.1* | 1.732454088 | 0.741833333 | -1.22365012 | 1.78E-08 | 2.00E-07 |
| *AC005746.2* | 2.032293396 | 0.959361905 | -1.082961637 | 1.79E-08 | 2.02E-07 |
| *CAPS* | 151.8664239 | 70.15025833 | -1.114282618 | 1.80E-08 | 2.03E-07 |
| *IGLV5-37* | 3.399553774 | 16.68824524 | 2.295414969 | 1.81E-08 | 2.03E-07 |
| *NELL1* | 1.629206604 | 0.077961905 | -4.385256418 | 1.84E-08 | 2.06E-07 |
| *AC087857.1* | 7.147058491 | 2.673388095 | -1.418680307 | 1.84E-08 | 2.07E-07 |
| *CASC22* | 2.538841824 | 0.094115476 | -4.753594727 | 1.85E-08 | 2.07E-07 |
| *AC011365.1* | 0.175801572 | 0.033179762 | -2.405572535 | 1.85E-08 | 2.08E-07 |
| *CA4* | 6.087976101 | 1.900513095 | -1.679573729 | 1.89E-08 | 2.12E-07 |
| *MTND5P11* | 0.85859717 | 0.418885714 | -1.035424734 | 1.89E-08 | 2.12E-07 |
| *NFE2* | 1.515800629 | 3.136860714 | 1.04924146 | 1.95E-08 | 2.18E-07 |
| *SULT2A1* | 17.09842547 | 4.018869048 | -2.089002005 | 1.97E-08 | 2.20E-07 |
| *IGKV1D-12* | 1.605046541 | 3.608269048 | 1.168691785 | 2.00E-08 | 2.23E-07 |
| *IGHV7-4-1* | 11.42096887 | 85.66525714 | 2.907025173 | 2.02E-08 | 2.25E-07 |
| *GAPDHP14* | 0.274331761 | 0.579166667 | 1.078056911 | 2.05E-08 | 2.29E-07 |
| *IGHV1OR21-1* | 0.490309748 | 2.566179762 | 2.387856884 | 2.06E-08 | 2.29E-07 |
| *AL035425.3* | 2.136331132 | 0.089514286 | -4.57687353 | 2.09E-08 | 2.32E-07 |
| *FSIP2* | 0.779306289 | 0.25685119 | -1.601257699 | 2.11E-08 | 2.34E-07 |
| *AC079296.1* | 0.186527358 | 0.054259524 | -1.781438959 | 2.11E-08 | 2.34E-07 |
| *CACNB2* | 1.034148113 | 0.448516667 | -1.205209326 | 2.12E-08 | 2.35E-07 |
| *WFDC1* | 4.927264151 | 2.271865476 | -1.116909406 | 2.12E-08 | 2.35E-07 |
| *TRBV23-1* | 0.23616195 | 1.246159524 | 2.399640321 | 2.13E-08 | 2.35E-07 |

| *GSDMC* | 11.36503459 | 28.75225357 | 1.339072962 | 2.14E-08 | 2.36E-07 |
| --- | --- | --- | --- | --- | --- |
| *PROSER2* | 15.9916195 | 38.38160952 | 1.263099162 | 2.18E-08 | 2.40E-07 |
| *ESRRG* | 0.847941824 | 0.224283333 | -1.91864287 | 2.21E-08 | 2.44E-07 |
| *ADAM11* | 1.809272327 | 0.904488095 | -1.000236156 | 2.22E-08 | 2.45E-07 |
| *MFSD6L* | 4.065076415 | 1.522107143 | -1.417212557 | 2.23E-08 | 2.46E-07 |
| *AC016738.1* | 0.726980503 | 0.3154 | -1.204734013 | 2.27E-08 | 2.50E-07 |
| *ACTBL2* | 0.259876415 | 0.880370238 | 1.760284665 | 2.28E-08 | 2.50E-07 |
| *RNF157-AS1* | 1.070399057 | 0.46199881 | -1.21218771 | 2.32E-08 | 2.55E-07 |
| *AC110619.1* | 10.10342893 | 3.44422381 | -1.552594203 | 2.33E-08 | 2.55E-07 |
| *SLC7A4* | 10.83447925 | 3.882240476 | -1.480668422 | 2.36E-08 | 2.59E-07 |
| *ZSWIM5* | 2.481682704 | 1.076388095 | -1.205120331 | 2.40E-08 | 2.62E-07 |
| *AC133041.1* | 7.76437044 | 3.28004881 | -1.243151668 | 2.40E-08 | 2.62E-07 |
| *CHIT1* | 1.800854717 | 6.075509524 | 1.754323608 | 2.46E-08 | 2.69E-07 |
| *AC116317.1* | 0.426015409 | 0.186358333 | -1.19282628 | 2.50E-08 | 2.72E-07 |
| *IGLV3-12* | 1.055749057 | 3.8041 | 1.849288213 | 2.50E-08 | 2.72E-07 |
| *AL355499.1* | 0.163624843 | 0.04140119 | -1.982647648 | 2.52E-08 | 2.74E-07 |
| *LINC01615* | 3.310346855 | 8.362585714 | 1.336966704 | 2.60E-08 | 2.82E-07 |
| *MAGIX* | 8.107757233 | 3.792669048 | -1.096089405 | 2.61E-08 | 2.82E-07 |
| *AC139769.2* | 0.503943396 | 0.100380952 | -2.327776158 | 2.62E-08 | 2.83E-07 |
| *ALDH1L1* | 12.46542075 | 4.644196429 | -1.424430682 | 2.67E-08 | 2.88E-07 |
| *TRBV7-7* | 0.195401887 | 0.531496429 | 1.4436156 | 2.69E-08 | 2.90E-07 |
| *EEF1GP4* | 0.276801572 | 0.102289286 | -1.4361971 | 2.72E-08 | 2.93E-07 |
| *RGS6* | 0.993166667 | 0.38154881 | -1.380168216 | 2.74E-08 | 2.95E-07 |
| *AL032819.1* | 0.899273585 | 0.438825 | -1.035114373 | 2.74E-08 | 2.95E-07 |
| *IGLVI-70* | 0.613061635 | 4.169184524 | 2.765661196 | 2.75E-08 | 2.96E-07 |
| *TBC1D30* | 1.513016667 | 0.75352619 | -1.005698318 | 2.76E-08 | 2.96E-07 |
| *VEGFC* | 15.75476415 | 43.75771905 | 1.47374938 | 2.77E-08 | 2.98E-07 |
| *GTSF1L* | 0.115063836 | 0.279659524 | 1.281236983 | 2.82E-08 | 3.02E-07 |
| *KRTAP5-7* | 0.727505346 | 0.21002619 | -1.792388605 | 3.01E-08 | 3.21E-07 |
| *ASTN2* | 3.773114151 | 1.569457143 | -1.265490114 | 3.01E-08 | 3.21E-07 |
| *AC011498.4* | 0.812528616 | 0.304978571 | -1.413710745 | 3.07E-08 | 3.28E-07 |
| *KIF5C* | 1.64293522 | 0.736994048 | -1.156550724 | 3.08E-08 | 3.28E-07 |
| *AP000553.2* | 2.358468239 | 0.983716667 | -1.261535422 | 3.10E-08 | 3.30E-07 |
| *AL022724.2* | 0.117016667 | 0.35922619 | 1.618178511 | 3.11E-08 | 3.31E-07 |
| *ASIC5* | 1.031762264 | 0.039079762 | -4.722545099 | 3.11E-08 | 3.31E-07 |
| *CPLX2* | 2.288116038 | 0.064478571 | -5.149196627 | 3.12E-08 | 3.32E-07 |
| *SULT1A2* | 1.000013836 | 0.209219048 | -2.256933854 | 3.24E-08 | 3.44E-07 |
| *RADX* | 15.90955535 | 7.49762381 | -1.085388169 | 3.25E-08 | 3.45E-07 |
| *AL109659.2* | 0.336931761 | 0.138596429 | -1.281566349 | 3.29E-08 | 3.49E-07 |
| *DNAJB13* | 1.103562579 | 0.290035714 | -1.927865975 | 3.40E-08 | 3.59E-07 |
| *LINC01873* | 0.358546226 | 0.143238095 | -1.323743895 | 3.42E-08 | 3.61E-07 |
| *BIRC7* | 0.872456918 | 2.985652381 | 1.774890404 | 3.49E-08 | 3.68E-07 |
| *SMAD9* | 4.489477673 | 1.60912619 | -1.480270136 | 3.65E-08 | 3.83E-07 |
| *LINC01767* | 0.820953145 | 0.259271429 | -1.662836652 | 3.67E-08 | 3.86E-07 |
| *AL121829.2* | 1.623129245 | 0.698713095 | -1.216005798 | 3.71E-08 | 3.89E-07 |
| *IGHD3-3* | 1.447872013 | 4.853109524 | 1.744975341 | 3.73E-08 | 3.90E-07 |

| *POU2AF1* | 4.515022642 | 9.079470238 | 1.0078749 | 3.84E-08 | 4.02E-07 |
| --- | --- | --- | --- | --- | --- |
| *AC005746.3* | 3.041338679 | 1.470171429 | -1.048722092 | 3.88E-08 | 4.05E-07 |
| *IGKV1OR22-5* | 0.281758176 | 1.089154762 | 1.950679586 | 3.93E-08 | 4.10E-07 |
| *RMST* | 0.169338679 | 0.030209524 | -2.486836194 | 3.94E-08 | 4.11E-07 |
| *CECR2* | 2.795512893 | 1.033391667 | -1.435725844 | 3.97E-08 | 4.13E-07 |
| *WNT10B* | 1.01012956 | 2.227155952 | 1.140662238 | 3.97E-08 | 4.13E-07 |
| *IGLJ2* | 0.922539937 | 3.380639286 | 1.873612816 | 3.97E-08 | 4.13E-07 |
| *CXCL5* | 8.550870126 | 33.1091369 | 1.953086263 | 4.05E-08 | 4.21E-07 |
| *AP000867.5* | 2.008496226 | 0.500419048 | -2.004907141 | 4.06E-08 | 4.22E-07 |
| *AL035425.1* | 0.637618553 | 0.129507143 | -2.299661939 | 4.07E-08 | 4.22E-07 |
| *OR7E15P* | 0.223334277 | 0.091846429 | -1.28190916 | 4.21E-08 | 4.36E-07 |
| *MS4A8* | 5.794205346 | 0.608009524 | -3.252444987 | 4.21E-08 | 4.36E-07 |
| *RN7SL268P* | 1.792958805 | 0.775929762 | -1.208344372 | 4.21E-08 | 4.36E-07 |
| *DPT* | 5.480013836 | 12.7548869 | 1.218798666 | 4.28E-08 | 4.42E-07 |
| *LINC01213* | 5.328965094 | 2.465803571 | -1.111797506 | 4.36E-08 | 4.50E-07 |
| *AC003681.1* | 0.282921384 | 0.11894881 | -1.250060389 | 4.36E-08 | 4.50E-07 |
| *IGHV3-64D* | 18.45250943 | 63.58925119 | 1.784965892 | 4.38E-08 | 4.51E-07 |
| *SSTR2* | 0.832675472 | 2.435689286 | 1.548503872 | 4.40E-08 | 4.53E-07 |
| *LGALS9C* | 0.223241195 | 0.513469048 | 1.201674038 | 4.44E-08 | 4.57E-07 |
| *AC011503.1* | 3.083593082 | 0.455420238 | -2.759342087 | 4.46E-08 | 4.58E-07 |
| *TRHDE* | 1.773705975 | 0.666716667 | -1.411621178 | 4.47E-08 | 4.60E-07 |
| *OR7E121P* | 0.916406289 | 0.311142857 | -1.558410233 | 4.50E-08 | 4.62E-07 |
| *AC026803.3* | 2.135601572 | 0.812903571 | -1.393486385 | 4.51E-08 | 4.63E-07 |
| *AP005432.2* | 54.38024623 | 11.7334881 | -2.212450727 | 4.59E-08 | 4.71E-07 |
| *IGHV1-67* | 1.162260692 | 2.849402381 | 1.29372567 | 4.61E-08 | 4.73E-07 |
| *AL359183.1* | 2.15238522 | 1.048766667 | -1.037242568 | 4.63E-08 | 4.74E-07 |
| *AC137770.1* | 0.288806918 | 0.091353571 | -1.660572265 | 4.67E-08 | 4.78E-07 |
| *AC002480.1* | 0.314081447 | 0.901730952 | 1.521558321 | 4.93E-08 | 5.03E-07 |
| *AC096751.1* | 0.803084906 | 0.261638095 | -1.617979907 | 4.96E-08 | 5.05E-07 |
| *IGHV3-52* | 0.38502044 | 1.344170238 | 1.803708922 | 4.98E-08 | 5.07E-07 |
| *ATRNL1* | 0.421060692 | 0.080091667 | -2.394304152 | 4.99E-08 | 5.07E-07 |
| *LINC02018* | 0.461582075 | 0.207386905 | -1.154262402 | 5.00E-08 | 5.08E-07 |
| *GJC3* | 0.776183019 | 0.304261905 | -1.351083159 | 5.09E-08 | 5.15E-07 |
| *AL033381.1* | 0.26553239 | 0.103630952 | -1.357432883 | 5.14E-08 | 5.20E-07 |
| *IGHJ1* | 3.717981132 | 8.326625 | 1.163212403 | 5.19E-08 | 5.25E-07 |
| *LINC01882* | 0.970556604 | 3.284209524 | 1.75866191 | 5.23E-08 | 5.28E-07 |
| *KRT20* | 327.4522808 | 137.724894 | -1.249495327 | 5.30E-08 | 5.35E-07 |
| *UBE2L4* | 0.886513836 | 0.417509524 | -1.086334041 | 5.42E-08 | 5.47E-07 |
| *IGLV2-28* | 7.387483648 | 19.87897857 | 1.428088693 | 5.46E-08 | 5.50E-07 |
| *AL133370.1* | 10.14446541 | 3.473030952 | -1.546425667 | 5.54E-08 | 5.58E-07 |
| *KRT86* | 4.410567925 | 23.25237857 | 2.398341962 | 5.58E-08 | 5.62E-07 |
| *MYCN* | 12.38057264 | 5.4483 | -1.184199996 | 5.63E-08 | 5.67E-07 |
| *MIR3189* | 31.72233082 | 9.896013095 | -1.680579463 | 6.00E-08 | 6.01E-07 |
| *IGKV1D-16* | 3.204513208 | 10.784175 | 1.750738692 | 6.23E-08 | 6.22E-07 |
| *AC010754.1* | 0.170296226 | 0.406361905 | 1.254718693 | 6.25E-08 | 6.23E-07 |
| *AGAP11* | 0.514739623 | 0.151369048 | -1.765772611 | 6.36E-08 | 6.34E-07 |

| *ERAP2* | 20.52164874 | 43.57374524 | 1.086312477 | 6.47E-08 | 6.44E-07 |
| --- | --- | --- | --- | --- | --- |
| *LONRF2* | 1.00762044 | 0.283917857 | -1.827406798 | 6.66E-08 | 6.61E-07 |
| *SGO1-AS1* | 0.148423585 | 0.069567857 | -1.09322757 | 6.68E-08 | 6.62E-07 |
| *LINC01506* | 0.293150314 | 0.666119048 | 1.184139432 | 6.77E-08 | 6.70E-07 |
| *AC110285.2* | 7.491394969 | 2.768192857 | -1.43628993 | 6.91E-08 | 6.82E-07 |
| *ADAMTS19-AS1* | 0.120293082 | 0.042178571 | -1.511971536 | 6.95E-08 | 6.86E-07 |
| *GCNT4* | 11.20410629 | 4.925428571 | -1.185706408 | 7.07E-08 | 6.96E-07 |
| *RPRM* | 5.741868239 | 2.635041667 | -1.12369445 | 7.07E-08 | 6.96E-07 |
| *LINC01668* | 7.611791824 | 2.556709524 | -1.573947845 | 7.13E-08 | 7.01E-07 |
| *HEPHL1* | 3.302897484 | 8.548059524 | 1.371864762 | 7.13E-08 | 7.01E-07 |
| *FLJ31356* | 2.497162579 | 1.198310714 | -1.059287713 | 7.17E-08 | 7.05E-07 |
| *MCEMP1* | 0.707473899 | 1.58675119 | 1.165327096 | 7.42E-08 | 7.28E-07 |
| *VWA5B1* | 1.897356604 | 0.202534524 | -3.227751101 | 7.44E-08 | 7.29E-07 |
| *LEAP2* | 13.61985189 | 4.608372619 | -1.563381736 | 7.68E-08 | 7.50E-07 |
| *AP000553.7* | 6.910491509 | 2.808865476 | -1.298800794 | 7.74E-08 | 7.56E-07 |
| *PTGDR2* | 1.414018868 | 0.186740476 | -2.920694799 | 7.77E-08 | 7.58E-07 |
| *PLA2G2F* | 58.26532358 | 18.912525 | -1.623295534 | 7.86E-08 | 7.66E-07 |
| *AC005753.3* | 1.290922642 | 0.550796429 | -1.22881144 | 7.86E-08 | 7.66E-07 |
| *AC092802.1* | 0.229521069 | 0.108159524 | -1.085465889 | 8.04E-08 | 7.83E-07 |
| *LINC00456* | 0.894162893 | 0.336959524 | -1.407962374 | 8.05E-08 | 7.83E-07 |
| *MYCL-AS1* | 1.975557862 | 0.630167857 | -1.648452028 | 8.15E-08 | 7.92E-07 |
| *MTND2P28* | 734.2327289 | 341.5506238 | -1.104137998 | 8.23E-08 | 7.99E-07 |
| *AC103409.1* | 0.622172642 | 0.100185714 | -2.634638151 | 8.41E-08 | 8.15E-07 |
| *AL451069.2* | 3.089358805 | 1.443317857 | -1.097918384 | 8.41E-08 | 8.15E-07 |
| *FCRLB* | 28.47929717 | 8.841104762 | -1.687614982 | 8.83E-08 | 8.54E-07 |
| *PLEKHH1* | 7.456772642 | 3.271344048 | -1.188667858 | 8.88E-08 | 8.59E-07 |
| *ZDHHC20-IT1* | 1.188756289 | 0.492883333 | -1.270134871 | 8.98E-08 | 8.66E-07 |
| *IGLJ3* | 0.186616981 | 0.550894048 | 1.561694605 | 9.02E-08 | 8.69E-07 |
| *BNIPL* | 17.86112862 | 8.094794048 | -1.141756965 | 9.15E-08 | 8.80E-07 |
| *RNF223* | 18.92885189 | 9.133890476 | -1.051285513 | 9.28E-08 | 8.92E-07 |
| *ATOH8* | 5.664023585 | 1.592921429 | -1.830152166 | 9.31E-08 | 8.94E-07 |
| *AC005622.1* | 0.082736478 | 0.174996429 | 1.080730029 | 9.32E-08 | 8.94E-07 |
| *MEGF11* | 0.194747484 | 0.068109524 | -1.515676241 | 9.33E-08 | 8.96E-07 |
| *RGL3* | 22.15868396 | 9.135967857 | -1.27824272 | 9.36E-08 | 8.98E-07 |
| *LPA* | 0.32835283 | 0.137070238 | -1.260331536 | 9.49E-08 | 9.09E-07 |
| *FOXJ1* | 28.03811541 | 7.40944881 | -1.919951252 | 9.52E-08 | 9.11E-07 |
| *GUCA2A* | 27.01434182 | 1.051844048 | -4.682732809 | 9.62E-08 | 9.19E-07 |
| *DAB1* | 3.469740881 | 1.573422619 | -1.140921698 | 9.70E-08 | 9.25E-07 |
| *LINC02568* | 0.619557862 | 0.199345238 | -1.635969882 | 9.86E-08 | 9.40E-07 |
| *AC020951.1* | 0.605767296 | 0.166505952 | -1.863189939 | 9.93E-08 | 9.46E-07 |
| *AC008780.1* | 0.116755975 | 0.280160714 | 1.262758287 | 1.02E-07 | 9.65E-07 |
| *AC091544.3* | 0.395456289 | 0.158746429 | -1.316794104 | 1.02E-07 | 9.70E-07 |
| *SOST* | 5.851786478 | 14.76564762 | 1.3352956 | 1.03E-07 | 9.74E-07 |
| *LINC00518* | 0.165349057 | 0.783041667 | 2.243574263 | 1.03E-07 | 9.74E-07 |
| *PROK1* | 0.448893396 | 0.0474 | -3.243413908 | 1.03E-07 | 9.78E-07 |
| *AC108066.2* | 0.728986792 | 0.225428571 | -1.693222298 | 1.04E-07 | 9.79E-07 |

| *IGLV4-60* | 9.802693711 | 47.47452143 | 2.275903306 | 1.05E-07 | 9.92E-07 |
| --- | --- | --- | --- | --- | --- |
| *IGKV1OR2-11* | 0.309057862 | 1.518552381 | 2.296747805 | 1.06E-07 | 9.97E-07 |
| *TAC3* | 146.9990915 | 1.824591667 | -6.332089796 | 1.06E-07 | 1.00E-06 |
| *ERBB4* | 0.470594969 | 0.149205952 | -1.657180805 | 1.06E-07 | 1.00E-06 |
| *AC021491.4* | 1.579483648 | 0.455590476 | -1.793643507 | 1.09E-07 | 1.02E-06 |
| *EML6* | 1.571403145 | 0.53122381 | -1.564661638 | 1.10E-07 | 1.03E-06 |
| *AC011442.1* | 1.688791195 | 0.622589286 | -1.439638307 | 1.10E-07 | 1.04E-06 |
| *TMEM238L* | 0.71860566 | 0.270647619 | -1.408784603 | 1.13E-07 | 1.06E-06 |
| *AL022345.4* | 2.135795283 | 1.037416667 | -1.041777918 | 1.15E-07 | 1.08E-06 |
| *SNHG14* | 0.936991824 | 0.371420238 | -1.334984032 | 1.16E-07 | 1.09E-06 |
| *ADH1A* | 0.297723585 | 0.067875 | -2.133021315 | 1.18E-07 | 1.11E-06 |
| *RNU4-78P* | 3.123996541 | 0.861513095 | -1.858448225 | 1.19E-07 | 1.11E-06 |
| *MIR6730* | 3.463644969 | 1.346419048 | -1.363163567 | 1.19E-07 | 1.11E-06 |
| *ALKAL1* | 4.872680503 | 2.29810119 | -1.084273305 | 1.19E-07 | 1.11E-06 |
| *SIGLEC22P* | 0.146402516 | 0.30910119 | 1.078138866 | 1.20E-07 | 1.12E-06 |
| *ABCA10* | 2.829938679 | 1.35995 | -1.057217182 | 1.23E-07 | 1.14E-06 |
| *AC129507.2* | 1.465148428 | 0.566419048 | -1.371105139 | 1.24E-07 | 1.16E-06 |
| *SEMA6A-AS2* | 0.201423899 | 0.084579762 | -1.251850468 | 1.32E-07 | 1.22E-06 |
| *SNURF* | 0.721877987 | 0.295721429 | -1.287516224 | 1.32E-07 | 1.22E-06 |
| *AC097499.2* | 0.731092138 | 0.28687381 | -1.349636977 | 1.34E-07 | 1.24E-06 |
| *FUNDC2P4* | 0.090293082 | 0.230192857 | 1.350155709 | 1.36E-07 | 1.25E-06 |
| *PTPRQ* | 1.457171698 | 0.502119048 | -1.537069521 | 1.36E-07 | 1.26E-06 |
| *MIR548AN* | 4.167157233 | 1.159839286 | -1.845138625 | 1.38E-07 | 1.27E-06 |
| *NPTXR* | 22.71276195 | 11.02395238 | -1.042861592 | 1.46E-07 | 1.34E-06 |
| *PKIA-AS1* | 0.184716667 | 0.082694048 | -1.159458652 | 1.49E-07 | 1.36E-06 |
| *AP000553.6* | 7.757909119 | 3.46320119 | -1.163561674 | 1.52E-07 | 1.39E-06 |
| *TBX1* | 28.99582956 | 12.13437857 | -1.256745187 | 1.53E-07 | 1.40E-06 |
| *AC015908.2* | 0.374974843 | 0.113821429 | -1.720021616 | 1.57E-07 | 1.43E-06 |
| *AOC2* | 5.847454717 | 2.878114286 | -1.022684904 | 1.59E-07 | 1.45E-06 |
| *HOXB-AS3* | 3.920903459 | 1.391134524 | -1.494924184 | 1.64E-07 | 1.49E-06 |
| *IGHV3-35* | 0.80345 | 2.097708333 | 1.384533948 | 1.65E-07 | 1.49E-06 |
| *MIR7848* | 0.354624843 | 0.737732143 | 1.056803489 | 1.65E-07 | 1.49E-06 |
| *UPK3B* | 280.7981349 | 110.9443655 | -1.339696954 | 1.66E-07 | 1.50E-06 |
| *SLC16A5* | 43.85577736 | 20.03300714 | -1.13038791 | 1.66E-07 | 1.50E-06 |
| *SRMS* | 11.1661934 | 5.516860714 | -1.017217987 | 1.67E-07 | 1.51E-06 |
| *AGR2* | 253.1493638 | 124.4117131 | -1.02486654 | 1.67E-07 | 1.51E-06 |
| *FAM155B* | 2.893472013 | 1.105316667 | -1.388341937 | 1.71E-07 | 1.54E-06 |
| *C4B* | 2.662453459 | 5.447417857 | 1.03281623 | 1.75E-07 | 1.58E-06 |
| *LUZP2* | 2.309477673 | 0.762342857 | -1.59905471 | 1.77E-07 | 1.60E-06 |
| *IGHJ6* | 13.38617138 | 62.85442381 | 2.231270897 | 1.80E-07 | 1.62E-06 |
| *HMSD* | 0.466406918 | 1.304369048 | 1.483691018 | 1.81E-07 | 1.63E-06 |
| *SPATA17* | 1.428500314 | 0.698113095 | -1.032968675 | 1.82E-07 | 1.63E-06 |
| *IGKV1D-33* | 1.58780566 | 3.625161905 | 1.191011085 | 1.83E-07 | 1.64E-06 |
| *SNORD19C* | 18.94047799 | 8.993813095 | -1.074467931 | 1.84E-07 | 1.65E-06 |
| *HDAC1P1* | 0.149731132 | 0.048559524 | -1.624548038 | 1.87E-07 | 1.68E-06 |
| *TRABD2A* | 1.019877987 | 3.489532143 | 1.774637054 | 1.92E-07 | 1.72E-06 |

| *RPS3AP53* 0.404975472 | 0.191191667 | -1.082814887 | 1.94E-07 | 1.73E-06 |
| --- | --- | --- | --- | --- |
| *PPP1R9A* 3.677457862 | 1.666407143 | -1.141967882 | 1.94E-07 | 1.73E-06 |
| *AL591848.2* 0.393810377 | 0.190602381 | -1.046934987 | 1.96E-07 | 1.75E-06 |
| *AL136982.6* 0.380178931 | 0.122861905 | -1.629640926 | 2.05E-07 | 1.82E-06 |
| *GOLT1A* 51.17765409 | 25.01465714 | -1.032740339 | 2.06E-07 | 1.83E-06 |
| *AC021594.1* 0.133834277 | 0.331315476 | 1.307757939 | 2.07E-07 | 1.84E-06 |
| *AP005432.1* 0.664499686 | 0.104421429 | -2.669850716 | 2.08E-07 | 1.84E-06 |
| *AC073174.1* 1.050855346 | 0.038320238 | -4.777313755 | 2.10E-07 | 1.86E-06 |
| *LINC02862* 1.716634906 | 0.54504881 | -1.655125904 | 2.11E-07 | 1.86E-06 |
| *CLMP* 11.89476792 | 24.58379762 | 1.047380671 | 2.11E-07 | 1.87E-06 |
| *SYT2* 0.391423585 | 0.174647619 | -1.164283712 | 2.12E-07 | 1.87E-06 |
| *AL139351.1* 0.44256478 | 1.034814286 | 1.225411327 | 2.12E-07 | 1.88E-06 |
| *AL512652.2* 0.203786164 | 0.08365 | -1.284618655 | 2.17E-07 | 1.91E-06 |
| *BX324167.2* 0.27356761 | 0.10175 | -1.426868633 | 2.17E-07 | 1.91E-06 |
| *AC004597.1* 1.248355031 | 0.268257143 | -2.218339803 | 2.21E-07 | 1.94E-06 |
| *AP002754.1* 0.281471069 | 0.102869048 | -1.45217769 | 2.25E-07 | 1.98E-06 |
| *RRS1-AS1* 0.171464151 | 0.056267857 | -1.607524048 | 2.42E-07 | 2.12E-06 |
| *AC021491.2* 0.377967296 | 0.161233333 | -1.229111371 | 2.44E-07 | 2.13E-06 |
| *AC105235.1* 0.448367925 | 0.187896429 | -1.254745429 | 2.48E-07 | 2.16E-06 |
| *AC104461.1* 0.064928302 | 0.240819048 | 1.891030124 | 2.50E-07 | 2.18E-06 |
| *AC004706.1* 1.834158805 | 0.85902381 | -1.094348532 | 2.56E-07 | 2.22E-06 |
| *AC083801.2* 1.013039937 | 0.427246429 | -1.245550713 | 2.56E-07 | 2.22E-06 |
| *FGFR3* 203.9258648 | 82.27907738 | -1.309447248 | 2.57E-07 | 2.22E-06 |
| *CTXND1* 2.405260063 | 1.073316667 | -1.164117105 | 2.57E-07 | 2.23E-06 |
| *USP3-AS1* 0.325128302 | 0.136814286 | -1.248790266 | 2.60E-07 | 2.25E-06 |
| *AC009242.1* 0.283858491 | 0.128432143 | -1.144165583 | 2.61E-07 | 2.25E-06 |
| *SMIM32* 0.25838805 | 0.060128571 | -2.103416764 | 2.61E-07 | 2.26E-06 |
| *AC113346.1* 1.930412579 | 4.648638095 | 1.267898893 | 2.62E-07 | 2.26E-06 |
| *AC025268.1* 0.501107862 | 0.160453571 | -1.64296527 | 2.64E-07 | 2.28E-06 |
| *AUXG01000058.*0.702616981 | 0.263733333 | -1.413658519 | 2.68E-07 | 2.31E-06 |
| *U73166.1* 1.184242138 | 0.560019048 | -1.080416292 | 2.74E-07 | 2.35E-06 |
| *HMGB3P10* 0.912974214 | 0.31422381 | -1.538781612 | 2.81E-07 | 2.41E-06 |
| *ERBB2* 211.216606 | 104.7800595 | -1.011359078 | 2.84E-07 | 2.43E-06 |
| *FABP6-AS1* 0.30004434 | 0.09704881 | -1.628393293 | 2.87E-07 | 2.46E-06 |
| *IFNK* 0.180022013 | 7.798578571 | 5.43696596 | 2.87E-07 | 2.46E-06 |
| *IGHV3OR16-6* 0.336240881 | 0.81792619 | 1.282475521 | 2.88E-07 | 2.46E-06 |
| *RAD1P2* 0.091874214 | 0.560772619 | 2.609684003 | 2.88E-07 | 2.47E-06 |
| *NKILA* 2.56373239 | 5.679955952 | 1.147634065 | 2.94E-07 | 2.51E-06 |
| *EPHA7* 2.360787736 | 0.91630119 | -1.365374532 | 2.95E-07 | 2.52E-06 |
| *MIR7152* 4.709712264 | 1.059641667 | -2.152062443 | 2.96E-07 | 2.52E-06 |
| *BNC1* 6.893513836 | 17.75870595 | 1.365214997 | 3.02E-07 | 2.57E-06 |
| *OVOL1-AS1* 2.802792138 | 1.088308333 | -1.364777404 | 3.11E-07 | 2.64E-06 |
| *ADH1C* 26.44881824 | 6.935310714 | -1.931170838 | 3.14E-07 | 2.66E-06 |
| *AF064860.2* 0.348554717 | 0.092795238 | -1.909262473 | 3.17E-07 | 2.68E-06 |
| *IGHV3-64* 3.631574528 | 10.89565952 | 1.585086434 | 3.23E-07 | 2.73E-06 |
| *NDNF* 1.692991195 | 0.55029881 | -1.621287357 | 3.26E-07 | 2.75E-06 |

| *ERN2* | 13.81706604 | 3.975640476 | -1.797192099 | 3.30E-07 | 2.78E-06 |
| --- | --- | --- | --- | --- | --- |
| *AC021146.12* | 3.505822642 | 0.986783333 | -1.828947758 | 3.35E-07 | 2.82E-06 |
| *AC099518.2* | 3.004875786 | 1.151790476 | -1.383427057 | 3.45E-07 | 2.89E-06 |
| *AC007336.2* | 0.127912264 | 0.349375 | 1.449621782 | 3.46E-07 | 2.90E-06 |
| *NEURL3* | 2.404938679 | 5.938791667 | 1.304169315 | 3.53E-07 | 2.95E-06 |
| *AC009228.1* | 0.138859119 | 0.069105952 | -1.006740042 | 3.55E-07 | 2.96E-06 |
| *SEMA6A-AS1* | 0.792948742 | 0.333539286 | -1.249370912 | 3.63E-07 | 3.02E-06 |
| *BTNL9* | 4.102371698 | 1.736677381 | -1.240128442 | 3.66E-07 | 3.04E-06 |
| *SLC15A5* | 0.191425786 | 0.019657143 | -3.283659634 | 3.68E-07 | 3.05E-06 |
| *MT1L* | 18.14974119 | 50.45313333 | 1.474994893 | 3.69E-07 | 3.06E-06 |
| *RPL35AP6* | 0.465888365 | 0.16465 | -1.500581789 | 3.71E-07 | 3.07E-06 |
| *GJB1* | 1.779110692 | 0.355038095 | -2.325110536 | 3.73E-07 | 3.09E-06 |
| *AC104819.3* | 0.957355346 | 0.4678 | -1.033162654 | 3.76E-07 | 3.11E-06 |
| *SLAMF9* | 1.431110063 | 3.089919048 | 1.110434411 | 3.79E-07 | 3.13E-06 |
| *AC026368.1* | 1.37692044 | 0.575541667 | -1.25845292 | 3.87E-07 | 3.20E-06 |
| *AC093690.1* | 0.39177327 | 0.159669048 | -1.294934301 | 3.88E-07 | 3.20E-06 |
| *LINC00840* | 0.840515094 | 0.218614286 | -1.942886049 | 3.90E-07 | 3.21E-06 |
| *AP005900.1* | 0.399353145 | 0.063009524 | -2.664023262 | 3.93E-07 | 3.23E-06 |
| *NT5E* | 20.55668616 | 57.41009286 | 1.481696675 | 3.93E-07 | 3.24E-06 |
| *IGHV3-41* | 0.726983333 | 1.957813095 | 1.429248848 | 3.94E-07 | 3.24E-06 |
| *SLC31A1P1* | 0.177734591 | 0.378879762 | 1.092015589 | 3.95E-07 | 3.24E-06 |
| *AP000897.2* | 0.243815723 | 0.027754762 | -3.134983944 | 4.05E-07 | 3.32E-06 |
| *AC099518.4* | 0.226791195 | 0.093822619 | -1.27335695 | 4.09E-07 | 3.35E-06 |
| *C17orf50* | 0.816248428 | 0.388635714 | -1.070589822 | 4.16E-07 | 3.40E-06 |
| *IGFBP3* | 914.1764997 | 437.9337679 | -1.061760036 | 4.34E-07 | 3.53E-06 |
| *KREMEN2* | 3.48952327 | 7.836727381 | 1.167221358 | 4.43E-07 | 3.60E-06 |
| *MAP1LC3C* | 0.333906289 | 1.055114286 | 1.659884102 | 4.46E-07 | 3.62E-06 |
| *NSG1* | 19.28632327 | 6.594520238 | -1.548238525 | 4.54E-07 | 3.68E-06 |
| *AJ239328.1* | 0.29894717 | 0.101953571 | -1.551978239 | 4.71E-07 | 3.80E-06 |
| *AC073316.2* | 1.24639434 | 0.381584524 | -1.707686021 | 4.77E-07 | 3.84E-06 |
| *AC102945.2* | 2.935072956 | 1.272890476 | -1.205288074 | 4.77E-07 | 3.84E-06 |
| *LINC01977* | 1.182191824 | 0.561980952 | -1.07287101 | 4.81E-07 | 3.87E-06 |
| *AC009522.1* | 0.352710377 | 0.15245119 | -1.210136607 | 4.83E-07 | 3.88E-06 |
| *SCARNA7* | 7.466318239 | 0.8778 | -3.088432827 | 4.89E-07 | 3.92E-06 |
| *LINC00862* | 0.185118553 | 0.533084524 | 1.525914803 | 4.96E-07 | 3.97E-06 |
| *LINC00390* | 0.128423585 | 0.051578571 | -1.316066456 | 4.97E-07 | 3.98E-06 |
| *SLC26A5* | 0.311004088 | 0.097221429 | -1.677587306 | 5.02E-07 | 4.02E-06 |
| *ODF4* | 0.525713836 | 0.256684524 | -1.034281392 | 5.04E-07 | 4.03E-06 |
| *AL731567.1* | 10.11453522 | 4.403197619 | -1.199806527 | 5.06E-07 | 4.04E-06 |
| *AL035587.3* | 0.788110377 | 0.234833333 | -1.746760492 | 5.07E-07 | 4.05E-06 |
| *RFLNA* | 3.056574214 | 7.888202381 | 1.36778097 | 5.08E-07 | 4.06E-06 |
| *IGHV7-56* | 0.283777673 | 1.635669048 | 2.52704788 | 5.27E-07 | 4.20E-06 |
| *AL445309.1* | 0.549636164 | 0.202355952 | -1.441581643 | 5.37E-07 | 4.27E-06 |
| *CHAD* | 2.164803459 | 0.756020238 | -1.51773929 | 5.46E-07 | 4.33E-06 |
| *AC002044.1* | 2.766959119 | 1.11387381 | -1.31271553 | 5.49E-07 | 4.35E-06 |
| *PCP2* | 4.25689717 | 1.708552381 | -1.317027762 | 5.61E-07 | 4.44E-06 |

| *FAM242C* | 0.223589308 | 0.102809524 | -1.120877286 | 5.63E-07 | 4.45E-06 |
| --- | --- | --- | --- | --- | --- |
| *HRH3* | 1.626648742 | 0.54390119 | -1.580486261 | 5.63E-07 | 4.45E-06 |
| *COLEC10* | 0.585696226 | 0.214186905 | -1.451282322 | 5.66E-07 | 4.47E-06 |
| *MYT1* | 3.346846855 | 0.890102381 | -1.910759346 | 5.67E-07 | 4.48E-06 |
| *MST1L* | 0.607383333 | 0.281160714 | -1.111212296 | 5.69E-07 | 4.49E-06 |
| *AC004593.1* | 0.479341509 | 0.218891667 | -1.130836847 | 5.70E-07 | 4.49E-06 |
| *RHOT1P1* | 1.538139937 | 0.432052381 | -1.831908626 | 5.72E-07 | 4.51E-06 |
| *AC092569.1* | 1.950036478 | 0.93507381 | -1.060348959 | 5.75E-07 | 4.52E-06 |
| *FBLL1* | 3.700012893 | 1.50982381 | -1.293150095 | 5.82E-07 | 4.58E-06 |
| *AC025043.1* | 0.174294969 | 0.045196429 | -1.947250243 | 5.91E-07 | 4.64E-06 |
| *HLA-F-AS1* | 0.572639308 | 2.125288095 | 1.891959809 | 5.95E-07 | 4.66E-06 |
| *CELA2B* | 0.395384591 | 0.193921429 | -1.027784416 | 5.96E-07 | 4.67E-06 |
| *SPDYE3* | 0.118113836 | 0.048465476 | -1.285148648 | 5.97E-07 | 4.68E-06 |
| *AL049836.1* | 0.360175786 | 0.72307381 | 1.005441726 | 5.98E-07 | 4.69E-06 |
| *NMUR2* | 0.249446855 | 0.046222619 | -2.432061569 | 6.01E-07 | 4.70E-06 |
| *LGALS9B* | 0.318907547 | 0.756690476 | 1.246565047 | 6.06E-07 | 4.74E-06 |
| *AC027644.1* | 3.892211321 | 1.884407143 | -1.046479335 | 6.11E-07 | 4.77E-06 |
| *AC003956.1* | 1.232595912 | 0.581758333 | -1.083208035 | 6.12E-07 | 4.78E-06 |
| *FGF5* | 0.296202201 | 1.702757143 | 2.523218417 | 6.17E-07 | 4.81E-06 |
| *ANKRD20A11P* | 0.812637736 | 0.289152381 | -1.490782378 | 6.20E-07 | 4.83E-06 |
| *C4A* | 2.269406289 | 5.027217857 | 1.147445294 | 6.23E-07 | 4.85E-06 |
| *CHI3L2* | 6.29166478 | 16.299725 | 1.373333913 | 6.30E-07 | 4.90E-06 |
| *MIR200B* | 4.965827673 | 2.297932143 | -1.111698001 | 6.37E-07 | 4.94E-06 |
| *AXDND1* | 0.841887107 | 0.298779762 | -1.494544358 | 6.40E-07 | 4.96E-06 |
| *AP000553.5* | 1.40035283 | 0.667459524 | -1.069038115 | 6.43E-07 | 4.99E-06 |
| *AC100791.2* | 0.598541824 | 0.261232143 | -1.196119639 | 6.45E-07 | 5.00E-06 |
| *PADI3* | 173.4926069 | 66.24247738 | -1.389045652 | 6.51E-07 | 5.04E-06 |
| *AC006026.3* | 6.570430503 | 2.943794048 | -1.158311159 | 6.60E-07 | 5.11E-06 |
| *AC010735.1* | 0.869379874 | 0.233644048 | -1.895674415 | 6.71E-07 | 5.18E-06 |
| *IHH* | 4.741720755 | 1.116472619 | -2.086462833 | 6.83E-07 | 5.27E-06 |
| *CASQ1* | 15.1425327 | 7.014886905 | -1.110114778 | 7.02E-07 | 5.40E-06 |
| *MFSD4A* | 3.025241509 | 1.403572619 | -1.10794661 | 7.04E-07 | 5.41E-06 |
| *BRWD1P3* | 0.601121069 | 0.124971429 | -2.266057289 | 7.14E-07 | 5.48E-06 |
| *LINC01087* | 0.28591195 | 0.057609524 | -2.311191681 | 7.17E-07 | 5.50E-06 |
| *UNC13C* | 0.407808805 | 0.022854762 | -4.157326231 | 7.32E-07 | 5.60E-06 |
| *CLCA4* | 53.05616258 | 23.80892857 | -1.156017634 | 7.34E-07 | 5.61E-06 |
| *OR7E108P* | 0.424298428 | 0.168115476 | -1.335626792 | 7.41E-07 | 5.67E-06 |
| *KCNG2* | 0.231127673 | 0.463445238 | 1.003708872 | 7.43E-07 | 5.68E-06 |
| *LNC-LBCS* | 0.457667296 | 0.111507143 | -2.037163074 | 7.48E-07 | 5.71E-06 |
| *HLA-P* | 0.074709748 | 0.205971429 | 1.463075818 | 7.52E-07 | 5.73E-06 |
| *AC127024.2* | 0.469094025 | 0.211730952 | -1.147644937 | 7.54E-07 | 5.74E-06 |
| *KRT12* | 0.866912264 | 0.365483333 | -1.246080375 | 7.65E-07 | 5.81E-06 |
| *AC091544.7* | 0.440705031 | 0.146796429 | -1.585996499 | 7.65E-07 | 5.81E-06 |
| *KCNF1* | 4.942237736 | 1.998746429 | -1.306068954 | 7.68E-07 | 5.83E-06 |
| *LINC02067* | 0.206998113 | 0.066479762 | -1.638630497 | 7.70E-07 | 5.85E-06 |
| *RIPPLY2* | 0.300948428 | 0.110407143 | -1.446682768 | 7.86E-07 | 5.96E-06 |

| *TFF2* | 26.92274371 | 5.759735714 | -2.224750923 | 8.00E-07 | 6.04E-06 |
| --- | --- | --- | --- | --- | --- |
| *AC093520.2* | 0.187826415 | 0.390841667 | 1.057184306 | 8.08E-07 | 6.10E-06 |
| *UGT2B15* | 10.27176635 | 3.696046429 | -1.474629507 | 8.32E-07 | 6.27E-06 |
| *BRWD1P2* | 0.668377673 | 0.288902381 | -1.210081447 | 8.47E-07 | 6.35E-06 |
| *IGHV4OR15-8* | 0.166674843 | 0.502845238 | 1.59307808 | 8.82E-07 | 6.61E-06 |
| *AC021683.2* | 0.16307673 | 0.040236905 | -2.018959695 | 8.85E-07 | 6.63E-06 |
| *AC008870.5* | 1.414113208 | 0.360409524 | -1.972188581 | 9.20E-07 | 6.86E-06 |
| *MCOLN3* | 1.462106604 | 3.520607143 | 1.267775745 | 9.25E-07 | 6.90E-06 |
| *BMP7* | 19.99270881 | 8.459115476 | -1.240895234 | 9.40E-07 | 7.00E-06 |
| *TNFSF9* | 5.595359434 | 11.20127262 | 1.001359937 | 9.51E-07 | 7.07E-06 |
| *IGHV2-70D* | 5.089364151 | 31.64827024 | 2.636569323 | 9.68E-07 | 7.18E-06 |
| *HOXB-AS2* | 1.559212579 | 0.483371429 | -1.689613529 | 9.73E-07 | 7.21E-06 |
| *ST3GAL5-AS1* | 1.389624843 | 0.396871429 | -1.807951842 | 9.74E-07 | 7.22E-06 |
| *IGHV3-6* | 0.89016195 | 1.87197619 | 1.072422347 | 9.91E-07 | 7.35E-06 |
| *AC103563.2* | 0.922080189 | 0.114388095 | -3.010955306 | 9.94E-07 | 7.36E-06 |
| *KCTD14* | 0.930160063 | 2.645675 | 1.508084946 | 1.01E-06 | 7.46E-06 |
| *UGT1A9* | 0.367650943 | 0.13959881 | -1.397050048 | 1.01E-06 | 7.48E-06 |
| *HOXB8* | 17.26531761 | 5.833891667 | -1.565346373 | 1.04E-06 | 7.65E-06 |
| *AC019197.1* | 0.523211635 | 0.166892857 | -1.648472414 | 1.04E-06 | 7.70E-06 |
| *ATP1A4* | 6.063361006 | 2.358380952 | -1.362320944 | 1.09E-06 | 7.97E-06 |
| *AC022167.3* | 0.793816981 | 0.365744048 | -1.117972039 | 1.09E-06 | 7.98E-06 |
| *AC096751.2* | 1.225044969 | 0.441347619 | -1.472847388 | 1.09E-06 | 8.02E-06 |
| *AC093838.1* | 0.268443711 | 0.039679762 | -2.758144331 | 1.10E-06 | 8.04E-06 |
| *AC103563.1* | 0.824148428 | 2.855103571 | 1.792566989 | 1.12E-06 | 8.20E-06 |
| *CD1B* | 0.422560377 | 0.902992857 | 1.095557081 | 1.13E-06 | 8.24E-06 |
| *AC104964.3* | 0.797533962 | 0.328461905 | -1.279819901 | 1.13E-06 | 8.30E-06 |
| *TMEM255A* | 0.741353459 | 2.023952381 | 1.448941894 | 1.14E-06 | 8.32E-06 |
| *AL691432.4* | 2.583485535 | 1.232577381 | -1.067640584 | 1.14E-06 | 8.32E-06 |
| *DEPDC7* | 3.42474717 | 7.289460714 | 1.0898146 | 1.15E-06 | 8.39E-06 |
| *AC005920.1* | 0.311093711 | 0.130729762 | -1.250761606 | 1.16E-06 | 8.45E-06 |
| *HNF1B* | 10.17617013 | 4.332054762 | -1.23207131 | 1.16E-06 | 8.45E-06 |
| *SMIM6* | 4.904742138 | 2.092146429 | -1.229193461 | 1.16E-06 | 8.46E-06 |
| *MT-TL1* | 16.7296239 | 6.02279881 | -1.47389904 | 1.17E-06 | 8.55E-06 |
| *AC134407.3* | 0.288193711 | 0.119108333 | -1.274764498 | 1.18E-06 | 8.60E-06 |
| *AC099778.2* | 3.06152327 | 0.614321429 | -2.317184036 | 1.19E-06 | 8.67E-06 |
| *RDM1P3* | 0.401327987 | 0.159715476 | -1.329277654 | 1.20E-06 | 8.70E-06 |
| *AC012467.1* | 1.159825157 | 0.307730952 | -1.91416587 | 1.23E-06 | 8.93E-06 |
| *THBS4-AS1* | 0.372657233 | 0.151461905 | -1.298894283 | 1.27E-06 | 9.13E-06 |
| *LINC01517* | 0.174775786 | 0.019132143 | -3.19143495 | 1.28E-06 | 9.20E-06 |
| *CXXC4* | 1.645393396 | 0.654965476 | -1.32894179 | 1.33E-06 | 9.59E-06 |
| *KRT40* | 2.068573899 | 0.162114286 | -3.673553365 | 1.35E-06 | 9.67E-06 |
| *FMO2* | 0.753461006 | 3.853329762 | 2.354500899 | 1.35E-06 | 9.70E-06 |
| *AC068580.2* | 3.728251887 | 1.75270119 | -1.088919275 | 1.36E-06 | 9.74E-06 |
| *AL109659.3* | 0.173841509 | 0.072267857 | -1.266346585 | 1.37E-06 | 9.80E-06 |
| *AL137802.2* | 0.250974528 | 0.109717857 | -1.1937426 | 1.38E-06 | 9.90E-06 |
| *AC012317.2* | 0.509263836 | 0.128371429 | -1.988089133 | 1.41E-06 | 1.01E-05 |

| *CCL15* | 1.863206604 | 0.259911905 | -2.841693038 | 1.44E-06 | 1.03E-05 |
| --- | --- | --- | --- | --- | --- |
| *AL357873.1* | 0.134525157 | 0.060295238 | -1.15776002 | 1.45E-06 | 1.04E-05 |
| *AC243829.1* | 0.326868868 | 0.835760714 | 1.354377968 | 1.47E-06 | 1.05E-05 |
| *AC008543.3* | 0.799317296 | 0.273180952 | -1.548911412 | 1.50E-06 | 1.07E-05 |
| *AC084740.1* | 0.219748113 | 0.075614286 | -1.539120046 | 1.52E-06 | 1.08E-05 |
| *AC027104.1* | 0.798974843 | 0.369084524 | -1.114198833 | 1.52E-06 | 1.08E-05 |
| *TTC34* | 0.286949057 | 0.12897619 | -1.153689868 | 1.54E-06 | 1.09E-05 |
| *ITGA8* | 3.866517925 | 1.847240476 | -1.065663211 | 1.54E-06 | 1.09E-05 |
| *TRAV8-5* | 0.178941509 | 0.381777381 | 1.093243539 | 1.54E-06 | 1.09E-05 |
| *CFAP77* | 0.687103774 | 0.252878571 | -1.442083216 | 1.56E-06 | 1.11E-05 |
| *AC093323.2* | 0.372779245 | 0.141238095 | -1.400192269 | 1.58E-06 | 1.12E-05 |
| *MROH3P* | 2.161878302 | 0.744370238 | -1.538193033 | 1.60E-06 | 1.13E-05 |
| *Z73429.1* | 0.293651887 | 0.078285714 | -1.907285936 | 1.60E-06 | 1.13E-05 |
| *MSMB* | 38.20950472 | 4.972111905 | -2.942000885 | 1.63E-06 | 1.15E-05 |
| *LINC00671* | 0.263439308 | 0.082519048 | -1.674671553 | 1.66E-06 | 1.17E-05 |
| *SSTR5* | 0.909924214 | 0.314803571 | -1.531294482 | 1.66E-06 | 1.17E-05 |
| *RAPSN* | 2.145593396 | 0.81215 | -1.401558586 | 1.67E-06 | 1.17E-05 |
| *SLC44A4* | 75.74782075 | 36.54514286 | -1.05152471 | 1.67E-06 | 1.17E-05 |
| *AL022318.1* | 0.634119497 | 0.226279762 | -1.486647177 | 1.70E-06 | 1.19E-05 |
| *AC108860.1* | 1.220940881 | 0.437633333 | -1.480198811 | 1.70E-06 | 1.19E-05 |
| *MAGI1-IT1* | 0.401347799 | 0.180575 | -1.152254815 | 1.70E-06 | 1.19E-05 |
| *AP003108.5* | 0.761037421 | 0.26879881 | -1.501440646 | 1.70E-06 | 1.19E-05 |
| *AC067852.4* | 0.70370566 | 0.222744048 | -1.659585238 | 1.70E-06 | 1.19E-05 |
| *AC103563.3* | 1.254510063 | 2.75192619 | 1.133317733 | 1.71E-06 | 1.20E-05 |
| *AF131215.7* | 1.679682704 | 0.750195238 | -1.16285072 | 1.75E-06 | 1.22E-05 |
| *AC116552.1* | 0.639342138 | 0.299183333 | -1.095558375 | 1.76E-06 | 1.23E-05 |
| *AL008718.1* | 0.412593082 | 0.085064286 | -2.278094183 | 1.78E-06 | 1.24E-05 |
| *CCL22* | 7.20827956 | 20.64519643 | 1.518079275 | 1.78E-06 | 1.24E-05 |
| *AC010201.1* | 1.285266352 | 0.634620238 | -1.018101933 | 1.78E-06 | 1.24E-05 |
| *AC084398.2* | 0.157449371 | 0.320370238 | 1.024852135 | 1.78E-06 | 1.24E-05 |
| *AL355076.2* | 0.080356918 | 0.189994048 | 1.241460079 | 1.79E-06 | 1.25E-05 |
| *AL022345.1* | 1.30144434 | 0.554105952 | -1.231879842 | 1.79E-06 | 1.25E-05 |
| *ZC3H12B* | 0.76596195 | 0.381359524 | -1.006120997 | 1.82E-06 | 1.26E-05 |
| *HOXB5* | 14.48761164 | 5.88595119 | -1.299472294 | 1.83E-06 | 1.27E-05 |
| *RPL12P30* | 0.311178931 | 0.088047619 | -1.821388485 | 1.83E-06 | 1.27E-05 |
| *LINC02800* | 0.304654717 | 0.137077381 | -1.152184545 | 1.83E-06 | 1.27E-05 |
| *STEAP2-AS1* | 0.337575472 | 0.130703571 | -1.368911519 | 1.84E-06 | 1.28E-05 |
| *TMEM220-AS1* | 0.333512264 | 0.119845238 | -1.476567228 | 1.84E-06 | 1.28E-05 |
| *IGKV1D-13* | 4.922486478 | 17.38807976 | 1.82063947 | 1.85E-06 | 1.28E-05 |
| *ZNF334* | 3.586246226 | 0.986942857 | -1.861436083 | 1.85E-06 | 1.28E-05 |
| *KRTAP1-1* | 1.185967296 | 0.192830952 | -2.620655676 | 1.86E-06 | 1.29E-05 |
| *AL592430.1* | 0.25204434 | 0.072267857 | -1.802251533 | 1.86E-06 | 1.29E-05 |
| *AL121895.1* | 0.459772642 | 0.150375 | -1.612355885 | 1.87E-06 | 1.29E-05 |
| *AC012358.1* | 0.495131447 | 0.247419048 | -1.000855009 | 1.93E-06 | 1.33E-05 |
| *MIR200A* | 8.829996541 | 4.184986905 | -1.077189764 | 1.97E-06 | 1.36E-05 |
| *HPCA* | 1.297678931 | 0.463528571 | -1.485203306 | 2.02E-06 | 1.39E-05 |

| *AC119744.1* | 0.196118868 | 0.060165476 | -1.704720548 | 2.03E-06 | 1.39E-05 |
| --- | --- | --- | --- | --- | --- |
| *BNIP3P40* | 0.578082075 | 0.111844048 | -2.369785862 | 2.09E-06 | 1.43E-05 |
| *MYRIP* | 0.551942453 | 0.227434524 | -1.279066588 | 2.11E-06 | 1.45E-05 |
| *NT5DC4* | 0.131643396 | 0.282963095 | 1.103978754 | 2.13E-06 | 1.45E-05 |
| *AC009803.1* | 0.331306604 | 0.152839286 | -1.11615154 | 2.13E-06 | 1.46E-05 |
| *GDF15* | 348.3315082 | 168.1272512 | -1.050907392 | 2.18E-06 | 1.49E-05 |
| *SYN2* | 2.522235849 | 0.900659524 | -1.485649453 | 2.27E-06 | 1.54E-05 |
| *SSTR5-AS1* | 1.153951887 | 0.500615476 | -1.204808276 | 2.33E-06 | 1.58E-05 |
| *PDE7B* | 3.374522956 | 1.565904762 | -1.107687097 | 2.33E-06 | 1.58E-05 |
| *KRT18P59* | 0.300686792 | 0.111780952 | -1.427587127 | 2.34E-06 | 1.58E-05 |
| *ADRA2B* | 1.401594969 | 0.520314286 | -1.429614278 | 2.35E-06 | 1.58E-05 |
| *AL122058.1* | 0.600404403 | 0.233332143 | -1.363549495 | 2.35E-06 | 1.59E-05 |
| *AC005041.1* | 0.26248805 | 0.116516667 | -1.171715412 | 2.41E-06 | 1.62E-05 |
| *SNAP91* | 1.459708176 | 0.445066667 | -1.713586616 | 2.41E-06 | 1.62E-05 |
| *AL136115.1* | 0.733580503 | 0.34280119 | -1.097583176 | 2.42E-06 | 1.63E-05 |
| *MFSD13B* | 0.64021195 | 0.31132619 | -1.040122656 | 2.45E-06 | 1.64E-05 |
| *AC072022.1* | 0.325895912 | 0.129284524 | -1.333861669 | 2.53E-06 | 1.69E-05 |
| *AC099482.1* | 0.603542453 | 0.114195238 | -2.401952761 | 2.54E-06 | 1.70E-05 |
| *AL645940.1* | 1.836880503 | 0.893085714 | -1.040387226 | 2.54E-06 | 1.70E-05 |
| *DLX6-AS1* | 1.083846226 | 0.373920238 | -1.535357622 | 2.58E-06 | 1.72E-05 |
| *AC002480.2* | 0.136272013 | 0.399732143 | 1.552544293 | 2.58E-06 | 1.72E-05 |
| *EDA* | 0.751431132 | 0.330314286 | -1.185801523 | 2.61E-06 | 1.74E-05 |
| *CHST9* | 0.694219811 | 0.299561905 | -1.212538369 | 2.66E-06 | 1.77E-05 |
| *IGHV3OR16-15* | 0.18467327 | 0.460884524 | 1.319430258 | 2.66E-06 | 1.77E-05 |
| *AL135818.3* | 0.552468553 | 1.136507143 | 1.040642498 | 2.67E-06 | 1.78E-05 |
| *AL031280.1* | 1.23467327 | 0.355904762 | -1.794566173 | 2.68E-06 | 1.78E-05 |
| *AL390755.3* | 0.184713208 | 0.484870238 | 1.392311675 | 2.83E-06 | 1.87E-05 |
| *GAPDHP62* | 0.262146855 | 0.119528571 | -1.133019727 | 2.93E-06 | 1.93E-05 |
| *FGFBP2* | 0.968712893 | 7.075872619 | 2.868767028 | 2.96E-06 | 1.95E-05 |
| *AC026348.1* | 0.3304 | 0.118183333 | -1.483187091 | 3.00E-06 | 1.97E-05 |
| *VSTM2A* | 0.151727358 | 0.017622619 | -3.10598099 | 3.01E-06 | 1.97E-05 |
| *MYO18B* | 0.552016038 | 0.117705952 | -2.229522903 | 3.04E-06 | 2.00E-05 |
| *AL031686.1* | 0.12641478 | 0.035477381 | -1.833193733 | 3.05E-06 | 2.00E-05 |
| *KRT14* | 1253.598149 | 2611.839802 | 1.058991457 | 3.05E-06 | 2.00E-05 |
| *AC117395.1* | 0.612394025 | 0.271279762 | -1.174678784 | 3.08E-06 | 2.02E-05 |
| *LINC01708* | 0.16791478 | 0.031202381 | -2.428001196 | 3.11E-06 | 2.04E-05 |
| *IGHD* | 19.00371164 | 39.13452738 | 1.042160801 | 3.12E-06 | 2.04E-05 |
| *AC124067.4* | 1.06998522 | 0.334364286 | -1.678098206 | 3.12E-06 | 2.04E-05 |
| *CRYM* | 4.259886792 | 1.103121429 | -1.949223483 | 3.17E-06 | 2.07E-05 |
| *UPK1A-AS1* | 0.524670126 | 0.211136905 | -1.313231876 | 3.22E-06 | 2.10E-05 |
| *SBK1* | 18.56064151 | 7.485190476 | -1.310135641 | 3.25E-06 | 2.12E-05 |
| *AC025539.1* | 0.433403459 | 0.183061905 | -1.243379071 | 3.28E-06 | 2.13E-05 |
| *CPA4* | 7.065179874 | 24.02890357 | 1.765972624 | 3.31E-06 | 2.15E-05 |
| *AVPR1B* | 0.155010063 | 0.043089286 | -1.846960787 | 3.37E-06 | 2.19E-05 |
| *PLPPR1* | 1.401694969 | 0.374427381 | -1.904414585 | 3.38E-06 | 2.20E-05 |
| *CDA* | 31.55946132 | 87.90281905 | 1.477836853 | 3.41E-06 | 2.21E-05 |

| *BX470102.1* | 24.60492044 | 12.19013452 | -1.013232805 | 3.44E-06 | 2.23E-05 |
| --- | --- | --- | --- | --- | --- |
| *CLDN14* | 0.546468553 | 1.452478571 | 1.410306496 | 3.49E-06 | 2.26E-05 |
| *AC068594.1* | 1.156044969 | 0.498617857 | -1.213191061 | 3.51E-06 | 2.27E-05 |
| *ERP27* | 69.21134434 | 31.60938214 | -1.130655692 | 3.54E-06 | 2.29E-05 |
| *CDKN2A* | 37.96651069 | 82.75842381 | 1.124178754 | 3.58E-06 | 2.31E-05 |
| *ALOX15* | 5.326525157 | 2.511545238 | -1.084619414 | 3.65E-06 | 2.35E-05 |
| *CACNA1I* | 1.270593082 | 0.355844048 | -1.83618506 | 3.67E-06 | 2.36E-05 |
| *TNC* | 47.93887264 | 97.9459 | 1.030789121 | 3.67E-06 | 2.36E-05 |
| *AL049869.2* | 0.591994025 | 0.250044048 | -1.243400354 | 3.74E-06 | 2.40E-05 |
| *AL138885.3* | 0.217953145 | 0.485491667 | 1.155428513 | 3.74E-06 | 2.40E-05 |
| *AC007849.1* | 1.526898113 | 0.435482143 | -1.80991833 | 3.77E-06 | 2.42E-05 |
| *AC136475.9* | 0.501459119 | 3.150884524 | 2.651552884 | 3.77E-06 | 2.42E-05 |
| *ZNF486* | 37.1740456 | 14.79066905 | -1.32960839 | 3.80E-06 | 2.44E-05 |
| *CTAGE3P* | 0.160781761 | 0.03317381 | -2.276987158 | 3.92E-06 | 2.50E-05 |
| *FAM189A1* | 3.097557547 | 1.544372619 | -1.004110204 | 4.00E-06 | 2.55E-05 |
| *AL356390.1* | 0.288892453 | 0.113988095 | -1.341649356 | 4.00E-06 | 2.55E-05 |
| *AP005435.1* | 0.956812893 | 0.282103571 | -1.762011899 | 4.03E-06 | 2.56E-05 |
| *AL049780.2* | 0.527934906 | 0.251146429 | -1.071831297 | 4.03E-06 | 2.57E-05 |
| *AC018665.1* | 6.719823899 | 3.307292857 | -1.022772626 | 4.07E-06 | 2.59E-05 |
| *AC093865.1* | 1.269765094 | 0.396520238 | -1.679095217 | 4.08E-06 | 2.59E-05 |
| *AP005212.4* | 0.260981132 | 0.081692857 | -1.675663663 | 4.14E-06 | 2.63E-05 |
| *FREM1* | 1.553963208 | 0.728634524 | -1.092685087 | 4.15E-06 | 2.63E-05 |
| *AC114812.1* | 0.171414465 | 0.060036905 | -1.513567356 | 4.20E-06 | 2.66E-05 |
| *ANGPT4* | 1.629794025 | 0.168354762 | -3.275113213 | 4.24E-06 | 2.69E-05 |
| *GGT2* | 0.886826415 | 0.223916667 | -1.985689828 | 4.31E-06 | 2.73E-05 |
| *FBLN1* | 390.9939157 | 185.1177762 | -1.078702719 | 4.43E-06 | 2.79E-05 |
| *FABP6* | 42.46423113 | 16.63373929 | -1.352135604 | 4.62E-06 | 2.90E-05 |
| *LINC02666* | 0.379389937 | 0.121830952 | -1.638800702 | 4.65E-06 | 2.91E-05 |
| *AF131215.6* | 4.100477044 | 1.86055119 | -1.140061676 | 4.66E-06 | 2.92E-05 |
| *DIRC3-AS1* | 0.403715409 | 0.025790476 | -3.968428336 | 4.70E-06 | 2.94E-05 |
| *AC073210.3* | 20.08803428 | 7.814619048 | -1.362088945 | 4.72E-06 | 2.95E-05 |
| *NR1H4* | 2.751178302 | 0.842269048 | -1.707696588 | 4.76E-06 | 2.97E-05 |
| *IGKV1D-27* | 0.911876101 | 2.032361905 | 1.156247608 | 4.78E-06 | 2.98E-05 |
| *BRINP2* | 0.627610692 | 0.060907143 | -3.365186595 | 4.78E-06 | 2.98E-05 |
| *TNNT2* | 0.427776415 | 0.163389286 | -1.388543562 | 4.80E-06 | 2.99E-05 |
| *SLC16A9* | 4.380884906 | 2.14634881 | -1.029337761 | 4.80E-06 | 2.99E-05 |
| *HOXB6* | 8.12082044 | 3.292171429 | -1.302586028 | 4.90E-06 | 3.05E-05 |
| *AP000553.4* | 4.104374214 | 1.721425 | -1.253558948 | 4.96E-06 | 3.08E-05 |
| *AL034550.1* | 1.00986195 | 0.380971429 | -1.406403377 | 5.08E-06 | 3.15E-05 |
| *MUC2* | 7.267963522 | 2.435957143 | -1.577062427 | 5.10E-06 | 3.16E-05 |
| *AL161668.3* | 0.707211321 | 0.275030952 | -1.362547378 | 5.18E-06 | 3.20E-05 |
| *PALM3* | 36.36906635 | 17.31142262 | -1.070987603 | 5.23E-06 | 3.23E-05 |
| *AMOT* | 8.310155346 | 3.305258333 | -1.330112412 | 5.28E-06 | 3.26E-05 |
| *TH* | 14.38317704 | 4.026639286 | -1.836734239 | 5.29E-06 | 3.27E-05 |
| *PIANP* | 1.809172327 | 0.559504762 | -1.693107519 | 5.36E-06 | 3.31E-05 |
| *AC021683.1* | 0.418338679 | 0.116975 | -1.838471166 | 5.38E-06 | 3.32E-05 |

| *PDPN* | 34.87949308 | 72.88802381 | 1.063302713 | 5.42E-06 | 3.34E-05 |
| --- | --- | --- | --- | --- | --- |
| *ATP2C2* | 8.076543711 | 3.924732143 | -1.04114384 | 5.49E-06 | 3.38E-05 |
| *HAS1* | 2.482615094 | 8.036863095 | 1.694771904 | 5.50E-06 | 3.38E-05 |
| *EGFEM1P* | 0.432807547 | 0.186046429 | -1.218062961 | 5.56E-06 | 3.42E-05 |
| *LINC01844* | 0.166337107 | 0.051321429 | -1.69647681 | 5.63E-06 | 3.46E-05 |
| *AL359715.4* | 1.651192453 | 0.644430952 | -1.357410587 | 5.67E-06 | 3.48E-05 |
| *MPV17L* | 2.537844969 | 1.232082143 | -1.042505497 | 5.70E-06 | 3.49E-05 |
| *AC087392.2* | 0.501236478 | 0.181105952 | -1.468657448 | 5.82E-06 | 3.56E-05 |
| *PIFO* | 5.853414151 | 2.435771429 | -1.2648996 | 5.91E-06 | 3.61E-05 |
| *MAN1C1* | 27.64786981 | 6.57269881 | -2.072610548 | 5.97E-06 | 3.65E-05 |
| *AC073592.8* | 0.089137736 | 0.393754762 | 2.14318915 | 5.98E-06 | 3.65E-05 |
| *C10orf82* | 1.503910377 | 0.467815476 | -1.6847071 | 6.03E-06 | 3.68E-05 |
| *AL157834.2* | 0.922313208 | 0.23860119 | -1.950655516 | 6.13E-06 | 3.73E-05 |
| *PROM1* | 4.653145597 | 1.685719048 | -1.464842222 | 6.19E-06 | 3.76E-05 |
| *AC000093.1* | 0.296140881 | 0.097240476 | -1.606654796 | 6.20E-06 | 3.77E-05 |
| *AL139039.3* | 0.082505346 | 0.1715 | 1.05564907 | 6.28E-06 | 3.82E-05 |
| *AL450263.1* | 1.26571195 | 0.581789286 | -1.121380481 | 6.36E-06 | 3.86E-05 |
| *LINC01342* | 0.383754717 | 0.179807143 | -1.093734151 | 6.41E-06 | 3.89E-05 |
| *IGLL1* | 0.128550943 | 0.416461905 | 1.695844336 | 6.42E-06 | 3.89E-05 |
| *AL033397.2* | 1.670573585 | 0.781604762 | -1.095832369 | 6.44E-06 | 3.90E-05 |
| *AL356433.1* | 6.158844654 | 2.90302619 | -1.085102152 | 6.47E-06 | 3.92E-05 |
| *AL353997.2* | 0.129966352 | 0.049953571 | -1.379478431 | 6.51E-06 | 3.94E-05 |
| *ITPRIP-AS1* | 0.708667925 | 0.236108333 | -1.58566079 | 6.56E-06 | 3.97E-05 |
| *LINC02721* | 0.090927044 | 0.187382143 | 1.043202116 | 6.56E-06 | 3.97E-05 |
| *MYLK3* | 0.161597484 | 0.07249881 | -1.156375528 | 6.58E-06 | 3.98E-05 |
| *AC090844.2* | 0.908197484 | 0.289083333 | -1.651520606 | 6.72E-06 | 4.05E-05 |
| *SPRR1A* | 532.5742503 | 207.7523393 | -1.358117955 | 6.85E-06 | 4.12E-05 |
| *GDPD2* | 11.28845377 | 4.639710714 | -1.282741126 | 6.94E-06 | 4.17E-05 |
| *AC073878.1* | 0.159299057 | 0.063516667 | -1.326530616 | 6.98E-06 | 4.19E-05 |
| *SLIT3* | 6.692883962 | 3.095366667 | -1.112517685 | 6.99E-06 | 4.19E-05 |
| *SMARCE1P6* | 0.448181761 | 0.219546429 | -1.029557873 | 7.01E-06 | 4.21E-05 |
| *AF064860.1* | 0.330566352 | 0.092992857 | -1.829748072 | 7.09E-06 | 4.25E-05 |
| *DISC1FP1* | 0.140830818 | 0.040295238 | -1.805281806 | 7.11E-06 | 4.26E-05 |
| *AC068587.4* | 0.335235849 | 0.157830952 | -1.086796273 | 7.15E-06 | 4.28E-05 |
| *AL772337.1* | 2.874519811 | 0.292708333 | -3.295785249 | 7.33E-06 | 4.38E-05 |
| *CPNE4* | 1.163257547 | 0.546380952 | -1.090191452 | 7.36E-06 | 4.39E-05 |
| *ATP7B* | 1.893060377 | 0.876861905 | -1.110298867 | 7.38E-06 | 4.40E-05 |
| *EVPLL* | 0.385787421 | 0.17027619 | -1.179929386 | 7.44E-06 | 4.44E-05 |
| *LINC01068* | 0.726872642 | 0.318921429 | -1.188501569 | 7.47E-06 | 4.45E-05 |
| *TMEM74B* | 3.284694025 | 1.393169048 | -1.237388662 | 7.49E-06 | 4.46E-05 |
| *RRAD* | 32.01777956 | 142.1350714 | 2.150317414 | 7.49E-06 | 4.46E-05 |
| *AP000553.9* | 0.29877327 | 0.084183333 | -1.827444543 | 7.55E-06 | 4.49E-05 |
| *FAHD2P1* | 5.711666667 | 1.697103571 | -1.750837173 | 7.56E-06 | 4.49E-05 |
| *IL24* | 3.137208805 | 6.881204762 | 1.13317962 | 7.60E-06 | 4.51E-05 |
| *AC008763.1* | 2.294280503 | 1.104394048 | -1.054786771 | 7.71E-06 | 4.57E-05 |
| *AC011379.1* | 0.149049057 | 0.049102381 | -1.601922357 | 7.79E-06 | 4.61E-05 |

| *AC129926.1* | 6.954009434 | 2.761917857 | -1.332174612 | 7.88E-06 | 4.66E-05 |
| --- | --- | --- | --- | --- | --- |
| *ZNF423* | 2.596692767 | 1.283266667 | -1.016854331 | 7.93E-06 | 4.68E-05 |
| *LINC01136* | 0.583303145 | 0.271853571 | -1.10141607 | 7.94E-06 | 4.69E-05 |
| *ZNF33BP1* | 0.191784277 | 0.058610714 | -1.710248122 | 7.96E-06 | 4.70E-05 |
| *SEMA5A-AS1* | 0.658554403 | 0.07677381 | -3.100616483 | 8.09E-06 | 4.77E-05 |
| *DAZL* | 0.114926415 | 0.257357143 | 1.163061394 | 8.19E-06 | 4.82E-05 |
| *CDKN2A-DT* | 0.513863208 | 1.04284881 | 1.021073749 | 8.23E-06 | 4.84E-05 |
| *EFEMP1* | 41.42819182 | 97.606275 | 1.236361046 | 8.27E-06 | 4.86E-05 |
| *AC015883.1* | 0.431648428 | 0.130471429 | -1.726122822 | 8.28E-06 | 4.87E-05 |
| *SCGB2A1* | 3.186490881 | 0.275270238 | -3.533047991 | 8.28E-06 | 4.87E-05 |
| *AC008739.2* | 3.080381447 | 0.912036905 | -1.755944904 | 8.30E-06 | 4.87E-05 |
| *CFL1P6* | 0.355937736 | 0.160003571 | -1.153520785 | 8.31E-06 | 4.88E-05 |
| *SEPTIN5* | 0.189699057 | 0.068492857 | -1.469687056 | 8.32E-06 | 4.88E-05 |
| *AC007834.2* | 1.022759748 | 0.459991667 | -1.152787658 | 8.48E-06 | 4.96E-05 |
| *AC022400.2* | 0.303980189 | 0.059494048 | -2.353160063 | 8.56E-06 | 5.00E-05 |
| *AC078880.3* | 3.376298742 | 0.464205952 | -2.862605635 | 8.89E-06 | 5.18E-05 |
| *PTPRR* | 13.69734434 | 3.939741667 | -1.79772327 | 9.01E-06 | 5.24E-05 |
| *AC122129.1* | 0.964289937 | 0.474694048 | -1.022469034 | 9.10E-06 | 5.28E-05 |
| *AL161431.1* | 8.155555346 | 38.23072024 | 2.228877355 | 9.15E-06 | 5.31E-05 |
| *TTR* | 21.14167956 | 0.838680952 | -4.655824092 | 9.17E-06 | 5.32E-05 |
| *HOXD4* | 7.467066038 | 3.261780952 | -1.19488159 | 9.19E-06 | 5.33E-05 |
| *AP003717.1* | 0.536972956 | 0.088802381 | -2.596179167 | 9.20E-06 | 5.33E-05 |
| *AC011944.1* | 0.21592044 | 0.4634 | 1.101758221 | 9.25E-06 | 5.35E-05 |
| *LINC02253* | 2.11397327 | 0.749486905 | -1.495981959 | 9.37E-06 | 5.41E-05 |
| *RNU6ATAC18P* | 5.155172642 | 2.354578571 | -1.130551877 | 9.38E-06 | 5.42E-05 |
| *AC131649.2* | 1.422117296 | 0.644316667 | -1.142198644 | 9.42E-06 | 5.44E-05 |
| *ZBTB2P1* | 0.67816478 | 0.293169048 | -1.209903067 | 9.44E-06 | 5.45E-05 |
| *AC094019.2* | 0.741744969 | 0.251684524 | -1.559306728 | 9.48E-06 | 5.47E-05 |
| *AL355482.2* | 0.811748742 | 0.372736905 | -1.122875576 | 9.63E-06 | 5.55E-05 |
| *RNY3P8* | 6.769994969 | 2.54177619 | -1.413317758 | 9.66E-06 | 5.56E-05 |
| *AC010969.3* | 0.205887421 | 0.081307143 | -1.340401688 | 9.72E-06 | 5.59E-05 |
| *FAM66A* | 0.198178302 | 0.088225 | -1.167539583 | 9.77E-06 | 5.62E-05 |
| *HMGA2* | 3.659226415 | 8.483369048 | 1.213098639 | 9.90E-06 | 5.69E-05 |
| *AC009336.1* | 0.332831761 | 0.160245238 | -1.054511626 | 1.00E-05 | 5.75E-05 |
| *AC099050.1* | 0.214124528 | 0.104385714 | -1.036525783 | 1.01E-05 | 5.80E-05 |
| *AC103702.1* | 0.186181132 | 0.070009524 | -1.411083776 | 1.03E-05 | 5.86E-05 |
| *ALDH1L1-AS1* | 0.239110063 | 0.069165476 | -1.789550843 | 1.03E-05 | 5.87E-05 |
| *AC003682.1* | 0.438781447 | 0.21492619 | -1.029661227 | 1.03E-05 | 5.88E-05 |
| *AL035416.1* | 0.199650314 | 0.082592857 | -1.27338642 | 1.03E-05 | 5.91E-05 |
| *IGKV7-3* | 0.319340252 | 0.851375 | 1.414700317 | 1.04E-05 | 5.91E-05 |
| *IGDCC3* | 2.063153459 | 0.810127381 | -1.34863046 | 1.04E-05 | 5.95E-05 |
| *IVL* | 168.4358009 | 82.78610357 | -1.024738292 | 1.05E-05 | 5.96E-05 |
| *TMEM163* | 6.878828302 | 2.810441667 | -1.291365975 | 1.06E-05 | 6.02E-05 |
| *HSD17B13* | 1.004758176 | 0.364828571 | -1.461557694 | 1.07E-05 | 6.09E-05 |
| *AP003068.4* | 2.393151887 | 1.076064286 | -1.153147694 | 1.08E-05 | 6.11E-05 |
| *AC021066.2* | 0.412933962 | 0.177853571 | -1.215221135 | 1.08E-05 | 6.12E-05 |

| *AP001994.3* | 2.122309119 | 0.460041667 | -2.205798364 | 1.08E-05 | 6.15E-05 |
| --- | --- | --- | --- | --- | --- |
| *H2BP9* | 0.383072013 | 0.183139286 | -1.064674325 | 1.09E-05 | 6.19E-05 |
| *OSR1* | 16.7957283 | 7.649310714 | -1.134692699 | 1.09E-05 | 6.19E-05 |
| *AC068587.2* | 2.01232327 | 0.690816667 | -1.542487291 | 1.11E-05 | 6.27E-05 |
| *IGKV3D-7* | 0.215955031 | 0.562853571 | 1.382028719 | 1.11E-05 | 6.27E-05 |
| *AP000424.1* | 1.912998428 | 0.478775 | -1.998415961 | 1.11E-05 | 6.29E-05 |
| *AC002306.1* | 0.261622013 | 0.107553571 | -1.282428501 | 1.12E-05 | 6.30E-05 |
| *AC004470.2* | 0.218897799 | 0.062694048 | -1.803857066 | 1.12E-05 | 6.31E-05 |
| *DACH1* | 1.801339937 | 0.711080952 | -1.340984746 | 1.12E-05 | 6.31E-05 |
| *AC021146.3* | 0.903842767 | 0.271696429 | -1.734076221 | 1.12E-05 | 6.32E-05 |
| *C1orf195* | 0.759011321 | 0.244557143 | -1.633949801 | 1.12E-05 | 6.33E-05 |
| *RPL21P54* | 0.307893396 | 0.06932619 | -2.150958534 | 1.12E-05 | 6.34E-05 |
| *SMYD3-AS1* | 0.506489937 | 0.028770238 | -4.13788454 | 1.13E-05 | 6.34E-05 |
| *PRKCG* | 0.161448428 | 0.549758333 | 1.767724177 | 1.13E-05 | 6.35E-05 |
| *BNIP3P16* | 1.507271069 | 0.487538095 | -1.628352038 | 1.14E-05 | 6.40E-05 |
| *AP006621.1* | 0.288534277 | 0.128259524 | -1.16967676 | 1.15E-05 | 6.47E-05 |
| *MIR6867* | 1.92199434 | 0.784758333 | -1.292283738 | 1.17E-05 | 6.54E-05 |
| *AC108215.1* | 0.250385849 | 0.100167857 | -1.321733392 | 1.18E-05 | 6.58E-05 |
| *AP000851.1* | 0.198526101 | 1.27327619 | 2.681144794 | 1.24E-05 | 6.89E-05 |
| *GAS6* | 38.34549528 | 79.30603333 | 1.048373521 | 1.25E-05 | 6.96E-05 |
| *AC006942.1* | 0.258321069 | 0.124692857 | -1.050786493 | 1.26E-05 | 7.02E-05 |
| *AC097637.2* | 0.707881447 | 0.324244048 | -1.126427673 | 1.27E-05 | 7.04E-05 |
| *AC116337.3* | 0.860607862 | 0.405583333 | -1.085357651 | 1.29E-05 | 7.15E-05 |
| *AC120036.3* | 1.364627673 | 0.544515476 | -1.325462419 | 1.30E-05 | 7.22E-05 |
| *ATP4A* | 0.327296855 | 0.088691667 | -1.883729279 | 1.31E-05 | 7.26E-05 |
| *LHX1-DT* | 0.552753774 | 1.24420119 | 1.170510917 | 1.32E-05 | 7.31E-05 |
| *AC004009.2* | 0.552027987 | 0.236433333 | -1.223307966 | 1.35E-05 | 7.43E-05 |
| *AL354953.1* | 2.375002201 | 5.284729762 | 1.15390085 | 1.35E-05 | 7.46E-05 |
| *S100A8* | 1582.850148 | 3836.072098 | 1.277105156 | 1.37E-05 | 7.52E-05 |
| *LINC02082* | 0.248370755 | 0.083225 | -1.577406438 | 1.37E-05 | 7.55E-05 |
| *MIR6784* | 5.017710063 | 1.782122619 | -1.493432505 | 1.38E-05 | 7.58E-05 |
| *FAM167A-AS1* | 0.137106918 | 0.050255952 | -1.447934983 | 1.38E-05 | 7.58E-05 |
| *LINC01983* | 2.920233648 | 1.02774881 | -1.506596103 | 1.43E-05 | 7.86E-05 |
| *AC025917.1* | 1.226063836 | 0.449785714 | -1.446724351 | 1.45E-05 | 7.94E-05 |
| *AC022613.1* | 0.60181478 | 1.563425 | 1.377318569 | 1.46E-05 | 7.99E-05 |
| *SULT4A1* | 2.247934277 | 0.624471429 | -1.847892385 | 1.46E-05 | 7.99E-05 |
| *AC005041.2* | 0.221859748 | 0.082019048 | -1.435617049 | 1.46E-05 | 8.00E-05 |
| *KRT6C* | 41.30488711 | 97.34243095 | 1.236756315 | 1.49E-05 | 8.11E-05 |
| *C1orf68* | 0.24323522 | 0.92317381 | 1.924250151 | 1.49E-05 | 8.12E-05 |
| *MFFP1* | 0.375515409 | 0.111191667 | -1.75582344 | 1.50E-05 | 8.16E-05 |
| *PCNPP3* | 0.392833333 | 1.419871429 | 1.85377104 | 1.52E-05 | 8.26E-05 |
| *AC013451.2* | 0.188266981 | 0.687960714 | 1.869546185 | 1.53E-05 | 8.33E-05 |
| *Z82217.1* | 0.256522327 | 0.059260714 | -2.113936479 | 1.53E-05 | 8.33E-05 |
| *AC018659.8* | 0.314963208 | 0.115355952 | -1.449090861 | 1.54E-05 | 8.37E-05 |
| *CYP2C8* | 1.231153145 | 0.45862381 | -1.424627072 | 1.55E-05 | 8.40E-05 |
| *CCIN* | 0.272681132 | 0.607857143 | 1.156517427 | 1.59E-05 | 8.59E-05 |

| *PART1* | 0.532985535 | 0.2519 | -1.081245256 | 1.59E-05 | 8.61E-05 |
| --- | --- | --- | --- | --- | --- |
| *TDGF1P5* | 0.473895597 | 0.164995238 | -1.522144871 | 1.59E-05 | 8.61E-05 |
| *RPL17P11* | 0.715688994 | 0.18792619 | -1.92916665 | 1.61E-05 | 8.73E-05 |
| *KPRP* | 1.433557862 | 0.334061905 | -2.101412759 | 1.62E-05 | 8.77E-05 |
| *LINC01698* | 0.114945597 | 0.269739286 | 1.230614445 | 1.65E-05 | 8.90E-05 |
| *AC011447.2* | 0.628052201 | 0.280982143 | -1.160406029 | 1.67E-05 | 9.00E-05 |
| *CSF3* | 8.614592453 | 18.76350833 | 1.123075152 | 1.70E-05 | 9.11E-05 |
| *AC114812.3* | 0.53405283 | 0.115285714 | -2.211768713 | 1.70E-05 | 9.12E-05 |
| *BPIFA2* | 0.685536164 | 0.079215476 | -3.113378554 | 1.72E-05 | 9.24E-05 |
| *MASP1* | 0.528368239 | 0.194608333 | -1.440970257 | 1.73E-05 | 9.27E-05 |
| *LINC01361* | 0.163475786 | 0.079817857 | -1.034293509 | 1.74E-05 | 9.32E-05 |
| *AC023090.1* | 2.343282075 | 0.840161905 | -1.479791354 | 1.76E-05 | 9.42E-05 |
| *OR10H1* | 0.966087421 | 0.397191667 | -1.28231839 | 1.76E-05 | 9.42E-05 |
| *SCGB1B2P* | 5.979253145 | 2.276632143 | -1.393064091 | 1.78E-05 | 9.48E-05 |
| *KCTD16* | 0.236043082 | 0.079182143 | -1.575803184 | 1.78E-05 | 9.52E-05 |
| *MICE* | 0.976661006 | 2.086095238 | 1.094875221 | 1.81E-05 | 9.63E-05 |
| *Z97653.2* | 0.303054403 | 0.124442857 | -1.284093377 | 1.83E-05 | 9.73E-05 |
| *LRTM1* | 1.969151887 | 0.12167381 | -4.016483831 | 1.90E-05 | 0.000100783 |
| *LINC02885* | 0.133288994 | 0.031386905 | -2.086322987 | 1.92E-05 | 0.000101623 |
| *AC020663.2* | 0.149345597 | 0.067019048 | -1.156011619 | 1.97E-05 | 0.000103856 |
| *KIF1A* | 1.920580818 | 0.926757143 | -1.051279439 | 1.99E-05 | 0.000104944 |
| *PPM1N* | 10.79809591 | 3.717645238 | -1.538315926 | 2.01E-05 | 0.000105579 |
| *AC009230.1* | 0.347309434 | 0.042678571 | -3.024637808 | 2.01E-05 | 0.000105584 |
| *AL133255.1* | 0.510453459 | 0.247889286 | -1.042083512 | 2.02E-05 | 0.000105872 |
| *AL157786.1* | 1.219003145 | 0.451367857 | -1.433326258 | 2.02E-05 | 0.000105876 |
| *GPC3* | 32.08480314 | 11.67180952 | -1.458861887 | 2.02E-05 | 0.000105876 |
| *AP001628.2* | 2.599678931 | 0.781220238 | -1.734532228 | 2.02E-05 | 0.000105894 |
| *AC108727.1* | 1.006172642 | 0.48069881 | -1.065672732 | 2.04E-05 | 0.000106891 |
| *AC005803.1* | 0.175852201 | 0.055522619 | -1.663215866 | 2.09E-05 | 0.000108937 |
| *UGT1A13P* | 0.530677673 | 0.123585714 | -2.102323865 | 2.10E-05 | 0.00010951 |
| *AL031663.3* | 0.398681132 | 0.097032143 | -2.038700692 | 2.10E-05 | 0.000109676 |
| *IGF2* | 25.29550566 | 11.10413929 | -1.187783509 | 2.13E-05 | 0.000110777 |
| *TFF1* | 126.6450623 | 32.3397619 | -1.969409867 | 2.14E-05 | 0.000111144 |
| *AP000344.2* | 0.343842453 | 0.12954881 | -1.408251921 | 2.15E-05 | 0.000111482 |
| *VEZTP1* | 0.174256604 | 0.073727381 | -1.240940917 | 2.16E-05 | 0.000111951 |
| *AC005790.1* | 1.252427358 | 0.486784524 | -1.363371722 | 2.16E-05 | 0.000112142 |
| *AC211433.1* | 0.479899686 | 0.154982143 | -1.630630871 | 2.18E-05 | 0.000112734 |
| *SLC8A2* | 1.130779874 | 0.480957143 | -1.233337862 | 2.18E-05 | 0.000113021 |
| *AC079416.2* | 0.191059748 | 0.06332381 | -1.593203915 | 2.19E-05 | 0.000113411 |
| *UGT1A4* | 0.127436478 | 0.029305952 | -2.120512672 | 2.19E-05 | 0.000113593 |
| *AC005336.2* | 0.382893396 | 0.075844048 | -2.335834914 | 2.22E-05 | 0.000114754 |
| *PICSAR* | 9.648836164 | 30.81290595 | 1.675107908 | 2.23E-05 | 0.000115035 |
| *AF131215.4* | 0.128728616 | 0.042339286 | -1.604263964 | 2.27E-05 | 0.000116751 |
| *IGKV2-29* | 5.324139937 | 11.15505357 | 1.067077046 | 2.28E-05 | 0.000117203 |
| *MYO16-AS1* | 0.670760377 | 1.809945238 | 1.432076673 | 2.29E-05 | 0.000117825 |
| *ASCL5* | 1.312030503 | 0.254941667 | -2.363562175 | 2.30E-05 | 0.000118364 |

| *AL390728.2* | 0.693480818 | 0.335429762 | -1.047845275 | 2.33E-05 | 0.000119587 |
| --- | --- | --- | --- | --- | --- |
| *CDK6-AS1* | 0.31750283 | 0.93012619 | 1.550657008 | 2.35E-05 | 0.000120363 |
| *AL450338.2* | 0.333615409 | 0.159014286 | -1.069029543 | 2.35E-05 | 0.000120363 |
| *SFTPA2* | 4.811851572 | 0.938071429 | -2.358822457 | 2.38E-05 | 0.00012221 |
| *CCDC198* | 3.219094025 | 0.483071429 | -2.736346285 | 2.45E-05 | 0.000125142 |
| *MT-TT* | 17.78277484 | 7.824861905 | -1.184343265 | 2.47E-05 | 0.000126106 |
| *AL031008.1* | 0.262397484 | 0.104528571 | -1.327856551 | 2.48E-05 | 0.000126347 |
| *AC011487.1* | 0.328877987 | 0.135086905 | -1.283664621 | 2.49E-05 | 0.000127141 |
| *AL136980.1* | 0.868125472 | 0.432622619 | -1.004794476 | 2.52E-05 | 0.000128367 |
| *MED28P7* | 0.25695 | 0.10235119 | -1.327959769 | 2.55E-05 | 0.00012935 |
| *WNT8B* | 1.102922013 | 0.296280952 | -1.896292997 | 2.56E-05 | 0.000130195 |
| *ZP1* | 1.220245912 | 0.459575 | -1.408799693 | 2.58E-05 | 0.000130801 |
| *RPSAP52* | 0.913238365 | 2.059371429 | 1.173140685 | 2.58E-05 | 0.000131025 |
| *LRRC4B* | 2.388724528 | 1.163916667 | -1.037252721 | 2.59E-05 | 0.000131278 |
| *AF131215.5* | 2.965578616 | 1.349332143 | -1.136068101 | 2.61E-05 | 0.000132034 |
| *TSPEAR-AS2* | 2.509829245 | 0.570077381 | -2.138359549 | 2.63E-05 | 0.000133197 |
| *IGKV2OR22-3* | 0.430935535 | 1.17319881 | 1.44490354 | 2.65E-05 | 0.000133719 |
| *PIWIL3* | 0.567660377 | 0.103705952 | -2.452529343 | 2.65E-05 | 0.000133849 |
| *AC090044.1* | 0.609316352 | 0.133595238 | -2.189322874 | 2.65E-05 | 0.000133877 |
| *AC092669.1* | 0.229156918 | 0.052 | -2.139752313 | 2.66E-05 | 0.000134059 |
| *AC023232.1* | 0.92715283 | 2.122265476 | 1.194726061 | 2.66E-05 | 0.000134073 |
| *AC019181.1* | 1.874480503 | 0.932144048 | -1.007865998 | 2.67E-05 | 0.000134597 |
| *AC008752.1* | 0.425072956 | 0.17385119 | -1.289857528 | 2.67E-05 | 0.000134706 |
| *AL591002.1* | 0.705714465 | 0.212922619 | -1.728755365 | 2.68E-05 | 0.000134934 |
| *AL355987.4* | 1.990482075 | 0.516741667 | -1.945602757 | 2.69E-05 | 0.000135294 |
| *FENDRR* | 4.598352516 | 1.804763095 | -1.349307597 | 2.69E-05 | 0.000135294 |
| *AC125616.1* | 0.325018868 | 0.058840476 | -2.465642644 | 2.70E-05 | 0.000135898 |
| *AL162457.2* | 0.10284717 | 0.234408333 | 1.188521765 | 2.71E-05 | 0.000136407 |
| *ZPLD1* | 0.141506604 | 0.510230952 | 1.850281039 | 2.74E-05 | 0.000137586 |
| *LINC02668* | 0.902650314 | 0.279711905 | -1.69022554 | 2.74E-05 | 0.000137632 |
| *AC027290.2* | 0.911423585 | 0.455409524 | -1.000957241 | 2.75E-05 | 0.000138058 |
| *AC004221.1* | 0.346751887 | 0.158110714 | -1.132968598 | 2.76E-05 | 0.000138618 |
| *LINC02865* | 0.07318805 | 0.328804762 | 2.167551174 | 2.80E-05 | 0.00014015 |
| *VEGFD* | 1.683363208 | 0.819269048 | -1.038937275 | 2.82E-05 | 0.000141201 |
| *SLC35D3* | 1.057051572 | 0.212417857 | -2.315068808 | 2.83E-05 | 0.000141482 |
| *SCN11A* | 0.538407547 | 0.083513095 | -2.688624292 | 2.86E-05 | 0.000143038 |
| *GDA* | 3.05594434 | 1.402638095 | -1.12347545 | 2.87E-05 | 0.000143538 |
| *PPIAP78* | 0.194276101 | 0.441355952 | 1.18383422 | 2.94E-05 | 0.00014647 |
| *SNX32* | 0.629227358 | 0.289669048 | -1.119175865 | 2.97E-05 | 0.000147914 |
| *MALAT1* | 117.092428 | 49.54291786 | -1.24089704 | 2.98E-05 | 0.000148138 |
| *AIFM3* | 7.020036478 | 2.603844048 | -1.430835484 | 2.98E-05 | 0.000148452 |
| *FBXO39* | 0.384642453 | 0.800471429 | 1.057331908 | 3.01E-05 | 0.000149422 |
| *BPIFB1* | 28.53652642 | 6.947833333 | -2.038174683 | 3.02E-05 | 0.000149808 |
| *AC010328.1* | 0.510584277 | 0.154677381 | -1.72288687 | 3.02E-05 | 0.000149949 |
| *C2orf72* | 2.983743082 | 1.162659524 | -1.35969464 | 3.07E-05 | 0.000152104 |
| *ALDH1A2* | 9.649685849 | 2.758784524 | -1.806449197 | 3.12E-05 | 0.000154289 |

| *AL161669.3* | 10.43927484 | 4.557385714 | -1.195743116 | 3.13E-05 | 0.000154815 |
| --- | --- | --- | --- | --- | --- |
| *TRIM9* | 1.611287107 | 0.723436905 | -1.155274482 | 3.13E-05 | 0.00015488 |
| *AC008752.2* | 0.343004717 | 0.13724881 | -1.321434781 | 3.19E-05 | 0.000157425 |
| *AC092338.1* | 0.68466761 | 0.266615476 | -1.360643237 | 3.19E-05 | 0.000157592 |
| *AC005856.1* | 0.449650314 | 0.11872619 | -1.921165255 | 3.21E-05 | 0.000158326 |
| *AC011322.1* | 1.79245 | 0.876939286 | -1.031384009 | 3.21E-05 | 0.000158326 |
| *MTCYBP18* | 3.954337107 | 0.99387381 | -1.992301274 | 3.28E-05 | 0.000161088 |
| *TRPM2-AS* | 7.165667296 | 3.219908333 | -1.154081443 | 3.32E-05 | 0.000162907 |
| *RPS3AP34* | 0.291472642 | 0.132869048 | -1.133355412 | 3.32E-05 | 0.000163042 |
| *AC002044.2* | 1.009761321 | 0.42864881 | -1.236146279 | 3.40E-05 | 0.000166533 |
| *AC097059.1* | 0.250011321 | 0.746195238 | 1.577559731 | 3.46E-05 | 0.000169105 |
| *MAPT* | 0.990461006 | 0.459878571 | -1.106847203 | 3.48E-05 | 0.000169965 |
| *GRM6* | 0.343868868 | 0.11562619 | -1.572390288 | 3.56E-05 | 0.000173412 |
| *ALG13-AS1* | 1.935998742 | 0.809584524 | -1.257824399 | 3.59E-05 | 0.000174954 |
| *AC008554.1* | 0.240904088 | 0.055796429 | -2.11021419 | 3.64E-05 | 0.000177018 |
| *CACNA1S* | 0.14159717 | 0.021630952 | -2.710623338 | 3.65E-05 | 0.000177365 |
| *SH2D5* | 1.300983333 | 3.535675 | 1.442383188 | 3.66E-05 | 0.000177488 |
| *AL137800.1* | 1.214538679 | 2.551266667 | 1.070805266 | 3.68E-05 | 0.000178425 |
| *AC009387.1* | 0.289316352 | 0.14407619 | -1.005815926 | 3.76E-05 | 0.000181797 |
| *MEIS1-AS3* | 0.30547044 | 0.133479762 | -1.194411762 | 3.76E-05 | 0.000181967 |
| *IGHV5-10-1* | 33.68646509 | 154.5708476 | 2.198027298 | 3.76E-05 | 0.000181967 |
| *TNRC18P1* | 0.277313836 | 0.136205952 | -1.025729849 | 3.79E-05 | 0.000182993 |
| *HOXD1* | 0.946442138 | 0.350785714 | -1.431924313 | 3.85E-05 | 0.000185605 |
| *LINC01399* | 0.932921069 | 0.397129762 | -1.232144541 | 3.87E-05 | 0.000186383 |
| *AC091906.1* | 0.440427987 | 0.085069048 | -2.372199941 | 3.88E-05 | 0.000186922 |
| *LAMC2* | 135.9179355 | 319.7636655 | 1.234270171 | 3.91E-05 | 0.000188014 |
| *AC100801.1* | 0.391960692 | 1.460565476 | 1.89774615 | 4.05E-05 | 0.000194188 |
| *ANKRD20A5P* | 0.75222327 | 0.27702381 | -1.44115096 | 4.10E-05 | 0.000196099 |
| *RPL23AP66* | 0.361298742 | 0.173961905 | -1.054420822 | 4.10E-05 | 0.000196323 |
| *MYH15* | 0.237601572 | 0.701563095 | 1.562028476 | 4.15E-05 | 0.000198575 |
| *AC002064.2* | 1.19609717 | 0.251289286 | -2.250913532 | 4.21E-05 | 0.000200936 |
| *TBL1Y* | 0.847625472 | 0.235163095 | -1.84976527 | 4.25E-05 | 0.000202426 |
| *UFL1-AS1* | 0.157348428 | 0.064970238 | -1.276111865 | 4.28E-05 | 0.000204107 |
| *MT-TM* | 6.691346541 | 2.012288095 | -1.733459695 | 4.42E-05 | 0.000209668 |
| *AC090061.1* | 0.798551887 | 0.318739286 | -1.325009304 | 4.59E-05 | 0.000216692 |
| *LGALS12* | 0.521612264 | 2.901772619 | 2.475884779 | 4.64E-05 | 0.000218788 |
| *FAM66D* | 0.258928616 | 0.127590476 | -1.021033773 | 4.66E-05 | 0.000219188 |
| *AC093535.1* | 0.530902516 | 0.231577381 | -1.196952631 | 4.68E-05 | 0.000220007 |
| *CHRNA9* | 0.277203459 | 2.227559524 | 3.006446818 | 4.73E-05 | 0.000222197 |
| *CYP1A1* | 180.8276119 | 67.83920714 | -1.414423777 | 4.74E-05 | 0.000222371 |
| *AL512328.1* | 0.290047799 | 0.077145238 | -1.910641658 | 4.76E-05 | 0.000223499 |
| *AC105101.2* | 0.121585849 | 0.049122619 | -1.307515941 | 4.81E-05 | 0.000225192 |
| *GGT3P* | 0.294575472 | 0.059591667 | -2.305454804 | 4.87E-05 | 0.000227967 |
| *OR1Q1* | 0.212741195 | 0.096675 | -1.137884658 | 4.93E-05 | 0.000230315 |
| *AC105219.1* | 4.205943082 | 2.089085714 | -1.00955764 | 4.96E-05 | 0.000231641 |
| *PROSER2-AS1* | 0.472638994 | 0.956975 | 1.017742577 | 4.99E-05 | 0.000233122 |

| *AC011451.3* | 0.7591 | 0.35907381 | -1.080009523 | 5.00E-05 | 0.000233195 |
| --- | --- | --- | --- | --- | --- |
| *AC005746.1* | 0.399721384 | 0.144982143 | -1.463119536 | 5.02E-05 | 0.000234003 |
| *TFPI2* | 30.72865314 | 74.52320476 | 1.278105182 | 5.02E-05 | 0.000234003 |
| *ADH4* | 1.210713208 | 0.328819048 | -1.880491383 | 5.10E-05 | 0.000237442 |
| *AC060834.1* | 0.170808491 | 0.052160714 | -1.711344159 | 5.15E-05 | 0.000239174 |
| *CADM3* | 1.098261006 | 5.697264286 | 2.375048374 | 5.18E-05 | 0.000240645 |
| *AC034102.8* | 0.515133019 | 0.235959524 | -1.126405614 | 5.22E-05 | 0.000242322 |
| *FABP4* | 237.8011277 | 78.77703095 | -1.593908607 | 5.22E-05 | 0.000242322 |
| *SH2D7* | 0.29007673 | 0.134121429 | -1.11289481 | 5.23E-05 | 0.000242554 |
| *AC010307.4* | 0.785895597 | 0.31815 | -1.304630548 | 5.26E-05 | 0.000243889 |
| *RPL6P4* | 1.742212893 | 0.178230952 | -3.289101119 | 5.27E-05 | 0.000244 |
| *AC022929.2* | 0.138040566 | 0.04125119 | -1.742584635 | 5.38E-05 | 0.000248398 |
| *KBTBD12* | 1.317722642 | 0.395366667 | -1.736783591 | 5.44E-05 | 0.000251169 |
| *AC005592.1* | 0.131578302 | 0.037022619 | -1.829442736 | 5.46E-05 | 0.000252027 |
| *ZNF662* | 5.958008176 | 2.297460714 | -1.374789911 | 5.50E-05 | 0.000253664 |
| *AC092969.1* | 0.406099686 | 0.18155 | -1.16146698 | 5.53E-05 | 0.000254523 |
| *MTND6P3* | 0.318488679 | 0.148603571 | -1.099773303 | 5.59E-05 | 0.000257011 |
| *SMIM1* | 4.452984277 | 2.090794048 | -1.090721561 | 5.59E-05 | 0.000257011 |
| *AL136531.2* | 0.602755975 | 0.236942857 | -1.347034876 | 5.60E-05 | 0.00025736 |
| *TAC1* | 2.406170755 | 0.161396429 | -3.898058468 | 5.65E-05 | 0.000259572 |
| *SLITRK2* | 0.085549686 | 1.048479762 | 3.615392652 | 5.70E-05 | 0.000261378 |
| *LINC01300* | 1.426527358 | 0.449958333 | -1.664644098 | 5.75E-05 | 0.000263344 |
| *IGHV1OR16-1* | 0.193489308 | 0.636557143 | 1.71803618 | 5.79E-05 | 0.000265023 |
| *AC018695.6* | 9.125926101 | 4.396108333 | -1.053744032 | 5.79E-05 | 0.000265302 |
| *REN* | 9.085044969 | 1.365479762 | -2.734085724 | 5.83E-05 | 0.000266804 |
| *DNAJB6P8* | 0.184715409 | 0.034789286 | -2.408589256 | 5.91E-05 | 0.000269568 |
| *DPP4-DT* | 0.180113836 | 0.37305119 | 1.050464598 | 5.92E-05 | 0.000269919 |
| *DPYS* | 0.08688239 | 0.234735714 | 1.433901666 | 5.96E-05 | 0.000271661 |
| *UGT1A2P* | 0.377692767 | 0.103313095 | -1.870190025 | 6.02E-05 | 0.000273883 |
| *TMEM191C* | 0.812773585 | 0.366527381 | -1.148932538 | 6.02E-05 | 0.000273922 |
| *AC110048.2* | 0.129904717 | 0.038952381 | -1.737670397 | 6.05E-05 | 0.000274845 |
| *AC124944.3* | 1.25106478 | 0.514691667 | -1.281376165 | 6.06E-05 | 0.000275308 |
| *FGF12-AS2* | 0.371666667 | 0.12282381 | -1.597419049 | 6.06E-05 | 0.000275405 |
| *LINC02443* | 0.189853459 | 0.055867857 | -1.764795894 | 6.06E-05 | 0.000275405 |
| *DCDC2C* | 0.138403774 | 0.051464286 | -1.42723977 | 6.13E-05 | 0.000277947 |
| *TACR3* | 0.18196478 | 0.045852381 | -1.988590682 | 6.17E-05 | 0.000279789 |
| *AP003327.2* | 0.459609119 | 0.185686905 | -1.307535349 | 6.37E-05 | 0.000287469 |
| *AL356867.1* | 3.280050314 | 1.012278571 | -1.696111582 | 6.42E-05 | 0.000289409 |
| *AC009879.4* | 0.220539937 | 0.049983333 | -2.141520913 | 6.42E-05 | 0.000289409 |
| *SKINT1L* | 0.206174214 | 0.093938095 | -1.134081661 | 6.43E-05 | 0.000289752 |
| *AC018647.1* | 0.112524843 | 0.055252381 | -1.026135009 | 6.48E-05 | 0.000291627 |
| *POU3F1* | 9.768149371 | 3.01027619 | -1.698189402 | 6.54E-05 | 0.00029396 |
| *COLEC11* | 3.159001887 | 1.417195238 | -1.156430276 | 6.57E-05 | 0.000295118 |
| *AP001931.1* | 0.312983648 | 0.14509881 | -1.109051601 | 6.63E-05 | 0.000297533 |
| *MOGAT2* | 4.445243396 | 1.496911905 | -1.570273096 | 6.65E-05 | 0.000298414 |
| *RCOR2* | 11.88196289 | 4.699497619 | -1.338194744 | 6.65E-05 | 0.00029854 |

| *LIPH* | 31.13597296 | 15.06059405 | -1.047803686 | 6.68E-05 | 0.000299762 |
| --- | --- | --- | --- | --- | --- |
| *SMIM23* | 0.215182704 | 0.056353571 | -1.932983174 | 6.70E-05 | 0.000300507 |
| *AC114341.2* | 0.119419182 | 0.493041667 | 2.045674977 | 6.72E-05 | 0.000301144 |
| *ERI3-IT1* | 0.352200943 | 0.104733333 | -1.749678094 | 6.73E-05 | 0.000301581 |
| *HOGA1* | 0.824658176 | 0.404988095 | -1.025916741 | 6.85E-05 | 0.000306479 |
| *PDE9A-AS1* | 0.114157547 | 0.053738095 | -1.087009151 | 6.88E-05 | 0.000307669 |
| *SPINK6* | 3.090533333 | 28.93065714 | 3.226671363 | 6.89E-05 | 0.000307974 |
| *AL109924.1* | 0.112414465 | 0.265230952 | 1.238421455 | 6.94E-05 | 0.00031 |
| *ADHFE1* | 1.358642138 | 0.611720238 | -1.151221593 | 6.94E-05 | 0.00031 |
| *LINC01082* | 0.219725157 | 0.106616667 | -1.043267077 | 7.10E-05 | 0.000316581 |
| *MEIOB* | 0.464577044 | 0.135172619 | -1.781114924 | 7.13E-05 | 0.000317659 |
| *AC091181.1* | 0.309706289 | 0.091977381 | -1.751549658 | 7.15E-05 | 0.000318216 |
| *AC005753.2* | 0.352546541 | 0.124116667 | -1.506116868 | 7.19E-05 | 0.000320258 |
| *LINC01833* | 4.518319182 | 2.109379762 | -1.098967335 | 7.20E-05 | 0.000320435 |
| *ELOCP21* | 0.366046855 | 0.135758333 | -1.430987572 | 7.23E-05 | 0.000321748 |
| *FADS3* | 11.93939811 | 28.06055119 | 1.232813238 | 7.24E-05 | 0.000321879 |
| *OR9K1P* | 0.562881761 | 0.251647619 | -1.161426954 | 7.31E-05 | 0.000324833 |
| *AC005696.4* | 1.110999686 | 0.513979762 | -1.112074949 | 7.32E-05 | 0.000325194 |
| *NPIPB1P* | 0.274524528 | 0.126225 | -1.12093738 | 7.34E-05 | 0.000325887 |
| *TLE6* | 6.643228302 | 3.214075 | -1.047480901 | 7.35E-05 | 0.000326257 |
| *AC061975.7* | 0.390039308 | 0.193665476 | -1.010052732 | 7.45E-05 | 0.000330139 |
| *AC012486.1* | 0.723584277 | 0.320616667 | -1.174311636 | 7.47E-05 | 0.00033088 |
| *ST3GAL5* | 10.2345739 | 4.316138095 | -1.245638109 | 7.50E-05 | 0.000331858 |
| *RPL3P1* | 0.2756 | 0.117254762 | -1.232929374 | 7.51E-05 | 0.000332294 |
| *C2orf66* | 2.117177358 | 0.915646429 | -1.209279608 | 7.52E-05 | 0.000332294 |
| *UOX* | 0.150822642 | 0.052863095 | -1.512520217 | 7.56E-05 | 0.000334083 |
| *ASTL* | 0.989411006 | 0.320458333 | -1.626433161 | 7.62E-05 | 0.00033613 |
| *AC022872.1* | 0.508848113 | 0.145492857 | -1.806286762 | 7.69E-05 | 0.000338601 |
| *LINC01414* | 0.201401258 | 0.069696429 | -1.530916058 | 7.76E-05 | 0.000341829 |
| *AC005740.3* | 0.49013239 | 0.179702381 | -1.447561965 | 7.81E-05 | 0.00034365 |
| *SLC26A9* | 0.602107862 | 2.577777381 | 2.098033817 | 7.92E-05 | 0.000348223 |
| *ERVW-1* | 0.500799686 | 0.036136905 | -3.792688811 | 7.95E-05 | 0.000349209 |
| *ORAOV1P1* | 0.917733333 | 0.308594048 | -1.572364775 | 7.97E-05 | 0.000349737 |
| *AC027811.1* | 0.147357233 | 0.06255 | -1.236236084 | 8.04E-05 | 0.000352444 |
| *AC010655.2* | 0.138144969 | 0.052005952 | -1.409434355 | 8.12E-05 | 0.000355448 |
| *AC073648.6* | 7.731839623 | 3.228989286 | -1.259729059 | 8.32E-05 | 0.000363262 |
| *NHS-AS1* | 0.52307327 | 0.200688095 | -1.38205801 | 8.43E-05 | 0.000367102 |
| *AC073349.2* | 1.133439623 | 0.481085714 | -1.236341677 | 8.43E-05 | 0.000367102 |
| *BNIP3P15* | 1.42666478 | 0.609780952 | -1.226283397 | 8.43E-05 | 0.000367102 |
| *AC087783.1* | 2.610594654 | 0.888675 | -1.55465066 | 8.45E-05 | 0.000368094 |
| *AC127164.1* | 0.161239623 | 0.079039286 | -1.028564498 | 8.46E-05 | 0.000368298 |
| *RN7SL441P* | 0.698488365 | 0.288386905 | -1.276230428 | 8.50E-05 | 0.000369491 |
| *CYP3A7* | 0.461034277 | 0.171054762 | -1.430415748 | 8.54E-05 | 0.000371104 |
| *AC009549.1* | 1.377401887 | 2.929157143 | 1.088536034 | 8.56E-05 | 0.000371657 |
| *DLX5* | 19.76513962 | 9.701540476 | -1.026672395 | 8.56E-05 | 0.000371657 |
| *LINC01549* | 0.31965283 | 0.104619048 | -1.611360326 | 8.68E-05 | 0.000375908 |

| *AC012618.2* | 0.70800566 | 0.33362381 | -1.085538642 | 8.69E-05 | 0.000376441 |
| --- | --- | --- | --- | --- | --- |
| *CPNE6* | 0.148466667 | 0.065913095 | -1.171502032 | 8.86E-05 | 0.000382494 |
| *BEAN1-AS1* | 0.277198113 | 0.138384524 | -1.002234829 | 8.89E-05 | 0.000383349 |
| *AC005821.2* | 0.179637736 | 0.053909524 | -1.736478372 | 8.99E-05 | 0.000387416 |
| *LINC00565* | 0.20595283 | 0.523108333 | 1.344795801 | 9.08E-05 | 0.000390969 |
| *SYT12* | 2.517265723 | 5.525202381 | 1.134169794 | 9.18E-05 | 0.000395015 |
| *KRT33B* | 3.24145 | 1.465594048 | -1.14515377 | 9.43E-05 | 0.000404134 |
| *AC007114.2* | 0.117402201 | 0.05250119 | -1.161037417 | 9.50E-05 | 0.000406999 |
| *RN7SL192P* | 1.437649371 | 0.58377619 | -1.300224583 | 9.52E-05 | 0.000407837 |
| *RNA5SP111* | 1.662054403 | 0.789885714 | -1.07325177 | 9.52E-05 | 0.000407837 |
| *SLC45A2* | 0.412723899 | 0.166733333 | -1.307634425 | 9.54E-05 | 0.000408498 |
| *LINC00189* | 0.202660692 | 0.457344048 | 1.174213584 | 9.69E-05 | 0.000414302 |
| *FRRS1L* | 0.236222642 | 0.04697381 | -2.330218747 | 9.69E-05 | 0.000414429 |
| *TFAP2B* | 0.972472956 | 0.205702381 | -2.241099637 | 9.85E-05 | 0.000419997 |
| *MUC13* | 1.04365566 | 4.62702381 | 2.148438729 | 9.91E-05 | 0.000422462 |
| *LINC02718* | 0.273616981 | 0.135413095 | -1.014790506 | 9.97E-05 | 0.000424346 |
| *NPAS2-AS1* | 0.129724214 | 0.049570238 | -1.387901699 | 9.97E-05 | 0.000424456 |
| *ANO2* | 0.493203145 | 0.239091667 | -1.044618151 | 9.98E-05 | 0.000424472 |
| *ITIH1* | 0.157312893 | 0.452514286 | 1.524326422 | 9.98E-05 | 0.000424541 |
| *AC012354.4* | 1.829311321 | 0.783257143 | -1.223742694 | 0.000100114 | 0.000425766 |
| *FBP2* | 1.06117044 | 0.371042857 | -1.515998654 | 0.00010057 | 0.000427189 |
| *MAB21L3* | 3.131970755 | 1.296969048 | -1.271926691 | 0.000100983 | 0.000428594 |
| *AC015922.1* | 0.700625157 | 0.282994048 | -1.307871083 | 0.000100988 | 0.000428594 |
| *MIR4728* | 6.446941824 | 2.341195238 | -1.461369716 | 0.000101128 | 0.000428965 |
| *DEFB126* | 4.15532044 | 1.899416667 | -1.129403318 | 0.000103314 | 0.000436806 |
| *AC022613.3* | 0.263583962 | 0.119254762 | -1.144215717 | 0.00010444 | 0.000440736 |
| *ZNF114* | 3.536383962 | 8.1451 | 1.203657488 | 0.000104642 | 0.000441287 |
| *RNF138P1* | 0.904327358 | 0.193109524 | -2.227425793 | 0.000105743 | 0.000445622 |
| *AL513548.4* | 0.190154403 | 0.087025 | -1.127669527 | 0.000108196 | 0.000454402 |
| *AC124319.2* | 0.258384591 | 0.092930952 | -1.475288939 | 0.000108503 | 0.00045538 |
| *EFNB3* | 6.65806195 | 2.898807143 | -1.19964294 | 0.000109043 | 0.000457566 |
| *CCL14* | 0.253063208 | 0.084790476 | -1.577523639 | 0.000110021 | 0.000461121 |
| *ALOXE3P1* | 0.55426478 | 0.155769048 | -1.831166748 | 0.000110415 | 0.000462537 |
| *DRD1* | 2.857662579 | 1.088389286 | -1.392640919 | 0.00011113 | 0.000465137 |
| *H3-4* | 0.240140881 | 0.10099881 | -1.249542734 | 0.00011192 | 0.000467647 |
| *PAGE5* | 5.909361006 | 0.56232619 | -3.393522989 | 0.000113242 | 0.000472365 |
| *IGKV1OR22-1* | 0.592681132 | 1.535395238 | 1.373282043 | 0.00011584 | 0.000481652 |
| *SLCO1B3* | 1.095947484 | 3.167397619 | 1.531119319 | 0.000116053 | 0.00048239 |
| *MYO1A* | 0.16202044 | 0.328832143 | 1.021175494 | 0.000116568 | 0.000484188 |
| *TAC4* | 1.034884906 | 0.441208333 | -1.229938383 | 0.000118147 | 0.000490168 |
| *ZNF432* | 16.48302956 | 7.802595238 | -1.078955466 | 0.000118626 | 0.000491989 |
| *PRR26* | 0.364504717 | 0.108414286 | -1.749382612 | 0.000119135 | 0.000493781 |
| *RPL21P13* | 5.407106918 | 1.467105952 | -1.88188382 | 0.000119604 | 0.000495412 |
| *FDCSP* | 34.05514214 | 639.4451595 | 4.230876076 | 0.000120696 | 0.000499228 |
| *AC090796.1* | 0.644083333 | 0.110407143 | -2.544413849 | 0.000120975 | 0.000500212 |
| *AC083967.1* | 0.499089308 | 9.035053571 | 4.178163252 | 0.000121466 | 0.000501751 |

| *AC005828.6* | 1.311487421 | 0.438322619 | -1.581138936 | 0.000121613 | 0.00050226 |
| --- | --- | --- | --- | --- | --- |
| *DPP10-AS1* | 0.205830818 | 0.024985714 | -3.042283636 | 0.000121745 | 0.000502696 |
| *AC022215.2* | 0.174299371 | 0.060035714 | -1.537674466 | 0.000121917 | 0.000503262 |
| *SCARNA21* | 12.56538333 | 2.839919048 | -2.145532973 | 0.000122424 | 0.000504928 |
| *AC010327.7* | 0.234455346 | 0.092296429 | -1.344966446 | 0.000123484 | 0.000508873 |
| *GRIN1* | 0.369587421 | 0.165302381 | -1.160808152 | 0.00012491 | 0.000514149 |
| *AL157813.2* | 0.286400943 | 0.077036905 | -1.894414601 | 0.000125314 | 0.000515552 |
| *AC004947.3* | 1.380896226 | 0.603472619 | -1.194244687 | 0.000125496 | 0.00051613 |
| *RABGAP1L-IT1* | 0.252696541 | 0.083803571 | -1.592322282 | 0.000125587 | 0.000516417 |
| *CYP2W1* | 6.34487044 | 2.823710714 | -1.167998411 | 0.000125646 | 0.000516573 |
| *AC023794.4* | 0.356622013 | 0.118792857 | -1.585947665 | 0.000125844 | 0.000517302 |
| *MYH7B* | 5.251665409 | 1.943629762 | -1.434021574 | 0.000126533 | 0.000519352 |
| *SAMD11* | 3.412704717 | 1.274885714 | -1.420547667 | 0.000129276 | 0.000529292 |
| *AL135787.1* | 0.943634277 | 0.332755952 | -1.503763351 | 0.000129278 | 0.000529292 |
| *LINC02418* | 0.229248742 | 0.065166667 | -1.814707711 | 0.000130502 | 0.000533415 |
| *VN1R83P* | 2.617645912 | 1.21640119 | -1.105650824 | 0.000131 | 0.000534919 |
| *AC048382.2* | 0.418587421 | 0.206928571 | -1.016396102 | 0.000131637 | 0.000537073 |
| *TIMM9P2* | 0.868785849 | 0.295932143 | -1.5537342 | 0.000131663 | 0.000537092 |
| *ZBTB40-IT1* | 0.979872013 | 0.473529762 | -1.049138218 | 0.000131764 | 0.000537414 |
| *VWA5B2* | 0.720648113 | 0.167041667 | -2.109086963 | 0.000132076 | 0.000538506 |
| *LINC01124* | 0.619402516 | 1.648461905 | 1.4121714 | 0.000132373 | 0.000539539 |
| *AC026369.1* | 5.022740881 | 0.60547619 | -3.052332715 | 0.000133681 | 0.000544061 |
| *AC011352.3* | 0.576373585 | 1.235197619 | 1.099665753 | 0.000133963 | 0.000545116 |
| *ADH7* | 19.8486522 | 9.190538095 | -1.110819809 | 0.000134045 | 0.000545358 |
| *LCN15* | 1.790147484 | 0.032971429 | -5.762718244 | 0.000134077 | 0.000545401 |
| *AC092042.4* | 0.13597956 | 0.05545119 | -1.294099465 | 0.000134545 | 0.000546853 |
| *AC015849.5* | 1.127818239 | 0.363770238 | -1.632435161 | 0.000135215 | 0.000549428 |
| *AP001630.1* | 0.74467327 | 0.216588095 | -1.781653626 | 0.000136027 | 0.000552328 |
| *ZNF660* | 1.067771698 | 0.531408333 | -1.006710458 | 0.000136385 | 0.000553502 |
| *AL359834.1* | 0.106102201 | 0.256541667 | 1.273738575 | 0.000136406 | 0.000553502 |
| *PEG3* | 0.502640566 | 0.070458333 | -2.834684856 | 0.000137475 | 0.000557288 |
| *AC110995.1* | 0.646157233 | 1.38194881 | 1.096747005 | 0.000137719 | 0.000558092 |
| *MMP11* | 144.8777352 | 293.3108262 | 1.017594424 | 0.00013844 | 0.000560553 |
| *ZNF114-AS1* | 0.206424528 | 0.496990476 | 1.267603797 | 0.000138981 | 0.000562558 |
| *SNORD19* | 8.111724843 | 4.050403571 | -1.001943054 | 0.00013968 | 0.00056492 |
| *CLEC4F* | 0.505887107 | 0.239864286 | -1.076597106 | 0.000140226 | 0.000566849 |
| *LINC02575* | 7.391666038 | 1.835897619 | -2.009413968 | 0.000141947 | 0.000572678 |
| *IGF2-AS* | 1.752312579 | 0.56709881 | -1.627588114 | 0.00014225 | 0.000573526 |
| *MSTN* | 0.220414151 | 0.088857143 | -1.310657192 | 0.000142416 | 0.000574007 |
| *RPL7P16* | 0.857478616 | 0.395902381 | -1.11495595 | 0.000142979 | 0.000576084 |
| *AC006960.3* | 0.238177673 | 0.101236905 | -1.234302876 | 0.000143673 | 0.000578787 |
| *MTCO3P12* | 106.4568623 | 23.48386786 | -2.180527001 | 0.00014386 | 0.000579255 |
| *SMAD9-IT1* | 0.250732704 | 0.077444048 | -1.694923925 | 0.000145754 | 0.000585825 |
| *C1GALT1P1* | 1.041444654 | 0.510258333 | -1.029286428 | 0.000146398 | 0.00058784 |
| *AL450326.2* | 0.170732704 | 0.063963095 | -1.416427779 | 0.000148332 | 0.000595023 |
| *AC005281.1* | 0.542675472 | 0.15809881 | -1.779263198 | 0.000148686 | 0.000596149 |

| *TSPEAR-AS1* | 1.838345283 | 0.402822619 | -2.190191163 | 0.000149946 | 0.000600222 |
| --- | --- | --- | --- | --- | --- |
| *MYO15A* | 0.591320755 | 0.133997619 | -2.141733549 | 0.000150113 | 0.000600511 |
| *SNRPGP4* | 1.326149057 | 0.367857143 | -1.85002543 | 0.000153591 | 0.000613417 |
| *AC104076.1* | 0.786507862 | 0.336641667 | -1.224247434 | 0.000153852 | 0.00061426 |
| *CYP2C9* | 2.132685849 | 0.202657143 | -3.395558537 | 0.000154197 | 0.000615136 |
| *ENPP7P4* | 0.414674843 | 0.140244048 | -1.564040986 | 0.000154419 | 0.000615924 |
| *AC012511.1* | 2.820890881 | 0.918308333 | -1.619100317 | 0.000155842 | 0.000620877 |
| *MTND4P35* | 0.740959434 | 0.328304762 | -1.174358884 | 0.000156537 | 0.00062296 |
| *AC093416.2* | 1.253027358 | 0.393083333 | -1.672510815 | 0.000156538 | 0.00062296 |
| *CYP4Z1* | 1.146759119 | 0.52987619 | -1.113835173 | 0.000156798 | 0.000623793 |
| *ANKK1* | 2.386568239 | 1.178603571 | -1.017859045 | 0.00015729 | 0.000625353 |
| *CD207* | 5.346580503 | 2.31047381 | -1.21042775 | 0.000159301 | 0.000632523 |
| *AC012501.2* | 0.242862579 | 0.09932381 | -1.289928708 | 0.000160344 | 0.00063615 |
| *LINC02598* | 2.186437107 | 1.065730952 | -1.036738579 | 0.000165203 | 0.000653322 |
| *AL078621.2* | 0.309575157 | 0.138233333 | -1.163184156 | 0.000166044 | 0.000656012 |
| *VCX3A* | 3.279120126 | 0.274183333 | -3.580095971 | 0.000166463 | 0.000657564 |
| *AC121764.1* | 6.964197799 | 2.069740476 | -1.750507299 | 0.000167406 | 0.000660971 |
| *PCDH15* | 0.124465094 | 0.025682143 | -2.276903714 | 0.000167445 | 0.000661016 |
| *CLDN24* | 0.126561635 | 0.348084524 | 1.459597527 | 0.000168779 | 0.000665325 |
| *IGKV1OR10-1* | 0.197289937 | 0.508070238 | 1.364710584 | 0.000170387 | 0.000670372 |
| *AC243829.2* | 0.117218868 | 0.324755952 | 1.470151159 | 0.000176233 | 0.000690281 |
| *Z69666.1* | 0.326270755 | 0.14450119 | -1.174988299 | 0.000176655 | 0.000691712 |
| *AL645924.2* | 0.490010063 | 0.17659881 | -1.472335759 | 0.000177381 | 0.000694334 |
| *OCLNP1* | 0.758997799 | 0.270482143 | -1.488562351 | 0.000179389 | 0.000700967 |
| *AC090912.2* | 0.265986164 | 0.728902381 | 1.454374414 | 0.000184181 | 0.0007173 |
| *AC005064.1* | 0.263234591 | 0.094397619 | -1.479526707 | 0.000184489 | 0.000718384 |
| *HSPA8P15* | 0.588875786 | 0.292458333 | -1.009732255 | 0.000185214 | 0.000720637 |
| *AC117498.2* | 0.437079245 | 0.214502381 | -1.026901212 | 0.000186063 | 0.000723371 |
| *SBK3* | 0.20995283 | 1.43900119 | 2.776930644 | 0.000187318 | 0.00072779 |
| *LINC01843* | 0.567445912 | 1.405505952 | 1.308534775 | 0.000190083 | 0.000736787 |
| *AC090164.2* | 1.678132704 | 0.702728571 | -1.255817345 | 0.000192121 | 0.000743629 |
| *SCIN* | 6.302561321 | 2.435042857 | -1.371991086 | 0.000192154 | 0.000743642 |
| *LHFPL1* | 0.812668239 | 0.314688095 | -1.368743909 | 0.000193226 | 0.00074732 |
| *AC090844.3* | 0.088979245 | 0.189261905 | 1.088843283 | 0.000193445 | 0.00074793 |
| *SPINK4* | 19.27262987 | 5.271517857 | -1.87026312 | 0.000195667 | 0.000755809 |
| *ZNF727* | 1.24721195 | 0.591421429 | -1.076448234 | 0.000199906 | 0.000770248 |
| *Z97653.1* | 0.891856918 | 0.433852381 | -1.039608029 | 0.000201145 | 0.000774295 |
| *ARL14EPL* | 0.203493711 | 0.932788095 | 2.19656517 | 0.000201232 | 0.000774508 |
| *HDAC1P2* | 0.121078616 | 0.054766667 | -1.144574114 | 0.000201379 | 0.00077483 |
| *TFF3* | 50.46576352 | 12.14451667 | -2.054999907 | 0.000204116 | 0.00078433 |
| *LINC02712* | 0.160772642 | 0.061010714 | -1.397887399 | 0.000205214 | 0.000787798 |
| *AP000697.1* | 0.990137107 | 0.482920238 | -1.035843388 | 0.00020523 | 0.000787798 |
| *NSA2P5* | 0.318588994 | 0.134692857 | -1.242023081 | 0.000211699 | 0.000810101 |
| *PPM1AP1* | 0.161425786 | 0.059378571 | -1.442856764 | 0.000212009 | 0.000810908 |
| *LINC00520* | 0.738521069 | 1.944560714 | 1.396733295 | 0.000212813 | 0.000813607 |
| *NTF3* | 1.018632075 | 0.426891667 | -1.254691147 | 0.000213714 | 0.000816665 |

| *RDH10-AS1* | 1.117432704 | 0.23115 | -2.273286682 | 0.000216425 | 0.000825491 |
| --- | --- | --- | --- | --- | --- |
| *AC083906.3* | 0.394184591 | 0.132091667 | -1.577331929 | 0.000219549 | 0.000835463 |
| *RNF183* | 1.587398428 | 0.72592619 | -1.128769509 | 0.000220288 | 0.000837884 |
| *FAM13C* | 0.76443239 | 0.328977381 | -1.216400514 | 0.000220975 | 0.000840113 |
| *AL031283.2* | 0.652874214 | 0.259857143 | -1.329086346 | 0.000221142 | 0.000840612 |
| *AC016717.2* | 0.826501887 | 0.174269048 | -2.245701762 | 0.000224696 | 0.000852278 |
| *AC136475.7* | 0.130409748 | 0.264225 | 1.018715257 | 0.000225034 | 0.000853428 |
| *ADAC* | 26.38908459 | 5.377266667 | -2.294996382 | 0.000226967 | 0.00085983 |
| *AC091151.1* | 0.190366038 | 0.050705952 | -1.908549097 | 0.000227715 | 0.000862135 |
| *CEACAM7* | 30.99627201 | 6.307161905 | -2.297031837 | 0.000229779 | 0.00086888 |
| *NXPH1* | 0.223217296 | 0.055530952 | -2.007084772 | 0.000230615 | 0.000871504 |
| *AC012354.2* | 13.29152516 | 6.576435714 | -1.015128867 | 0.000232171 | 0.00087671 |
| *SMCR5* | 0.186430503 | 0.0226 | -3.044243251 | 0.000242573 | 0.000912348 |
| *GREM2* | 1.446134591 | 0.491752381 | -1.556197887 | 0.000242916 | 0.000913496 |
| *AC092042.3* | 0.270882704 | 0.128267857 | -1.078508593 | 0.000243065 | 0.0009135 |
| *TPRG1-AS1* | 0.37841195 | 1.399040476 | 1.88640815 | 0.000244309 | 0.000917193 |
| *ASTN1* | 0.286071384 | 0.111669048 | -1.357145833 | 0.00024683 | 0.00092567 |
| *RHOT1P2* | 0.830368868 | 0.348641667 | -1.252007357 | 0.000247665 | 0.000928092 |
| *AC092902.4* | 0.144861635 | 0.061752381 | -1.230108898 | 0.000251751 | 0.000942254 |
| *AC128687.3* | 0.496308805 | 0.208569048 | -1.250712977 | 0.00025305 | 0.000946542 |
| *SLC4A4* | 2.648209434 | 1.152645238 | -1.200068675 | 0.000253297 | 0.000946918 |
| *AC112512.1* | 0.197160377 | 0.057353571 | -1.781414416 | 0.000253305 | 0.000946918 |
| *FOXN4* | 1.460102201 | 0.170522619 | -3.098034331 | 0.000254047 | 0.000949548 |
| *LINC02009* | 1.05500566 | 3.172509524 | 1.588373756 | 0.000258864 | 0.000964787 |
| *AC007431.1* | 0.128387107 | 0.030488095 | -2.074182404 | 0.000258869 | 0.000964787 |
| *RNY1P16* | 1.424673899 | 0.521477381 | -1.449955149 | 0.000260283 | 0.000969174 |
| *AC090826.3* | 0.320814151 | 0.107235714 | -1.580952312 | 0.000261679 | 0.000973194 |
| *MIR186* | 7.115028302 | 3.34582619 | -1.088506993 | 0.000262876 | 0.000977498 |
| *MKRN7P* | 0.20111195 | 0.087267857 | -1.204476529 | 0.000263966 | 0.000980959 |
| *AL139280.1* | 1.735268239 | 0.201791667 | -3.104220191 | 0.000266072 | 0.000988188 |
| *HSP90AB4P* | 0.703268553 | 0.301371429 | -1.22253306 | 0.000268842 | 0.000997571 |
| *SARNP* | 0.177043396 | 0.084315476 | -1.070233663 | 0.000271574 | 0.001006398 |
| *RPL11P3* | 0.964537107 | 0.413069048 | -1.223453782 | 0.000274966 | 0.001017227 |
| *KIT* | 10.9687544 | 3.347740476 | -1.712140108 | 0.000277254 | 0.001024612 |
| *AC018755.1* | 0.365576415 | 0.057235714 | -2.675185444 | 0.000277705 | 0.001026028 |
| *MRGPRX2* | 0.343590881 | 0.106610714 | -1.68833931 | 0.000279781 | 0.001032707 |
| *AC023510.2* | 2.092163836 | 0.965132143 | -1.116197443 | 0.000279849 | 0.001032805 |
| *VSNL1* | 7.498079874 | 15.16917738 | 1.016549752 | 0.000283565 | 0.001045265 |
| *RNF225* | 1.015165409 | 0.428453571 | -1.244504033 | 0.00028463 | 0.001048091 |
| *MTUS2-AS1* | 0.127953145 | 0.03835 | -1.738317122 | 0.000286555 | 0.001054232 |
| *AP000763.2* | 5.079283962 | 1.993308333 | -1.349460242 | 0.000287713 | 0.001057699 |
| *MYH16* | 0.413906604 | 1.673090476 | 2.015138293 | 0.000290009 | 0.001064408 |
| *PSD2* | 0.958079874 | 0.414157143 | -1.209967666 | 0.000291725 | 0.001070368 |
| *FGD5P1* | 0.159883333 | 0.075375 | -1.084861554 | 0.000293124 | 0.001075022 |
| *AC114812.2* | 0.939774214 | 0.452965476 | -1.052913087 | 0.000293399 | 0.001075711 |
| *AL162171.2* | 0.192983648 | 0.070596429 | -1.450811502 | 0.000298191 | 0.001091163 |

| *AC073349.1* | 0.207668553 | 0.072041667 | -1.527379307 | 0.000299833 | 0.001095704 |
| --- | --- | --- | --- | --- | --- |
| *DPP10* | 0.353541509 | 0.022430952 | -3.978316831 | 0.000302408 | 0.001103476 |
| *IGHV3OR16-10* | 0.280812893 | 0.584396429 | 1.057338185 | 0.000303372 | 0.001106499 |
| *LRRC3-DT* | 0.38228239 | 0.161032143 | -1.247290057 | 0.000309715 | 0.001126461 |
| *AC006059.1* | 1.024986792 | 0.42702619 | -1.263208858 | 0.000312084 | 0.00113391 |
| *MIR199A1* | 1.569996226 | 0.62152381 | -1.336879527 | 0.000313312 | 0.001137688 |
| *AC109992.2* | 0.189072013 | 0.086707143 | -1.124713072 | 0.000313616 | 0.001138461 |
| *ZDHHC8P1* | 1.710950314 | 0.785194048 | -1.123676724 | 0.000314272 | 0.001140232 |
| *ZNF454* | 0.599401572 | 0.245613095 | -1.287135386 | 0.000314274 | 0.001140232 |
| *AL096855.1* | 2.076717925 | 0.841629762 | -1.303047644 | 0.000315639 | 0.001144179 |
| *AC245884.9* | 0.427783019 | 0.185561905 | -1.204978654 | 0.000315656 | 0.001144179 |
| *ANGPTL3* | 0.298922642 | 0.085739286 | -1.801743873 | 0.000316248 | 0.001145817 |
| *AL008723.2* | 0.761650629 | 0.199341667 | -1.933886086 | 0.000316783 | 0.001147248 |
| *AC002401.4* | 7.764308176 | 18.25308929 | 1.23321137 | 0.000318774 | 0.001153608 |
| *AC083906.5* | 0.966301258 | 0.414529762 | -1.220997353 | 0.000319609 | 0.001156121 |
| *LINC01764* | 0.375449686 | 0.113770238 | -1.72249638 | 0.000322702 | 0.001166279 |
| *SALL1* | 0.869540252 | 0.334261905 | -1.379273869 | 0.00032434 | 0.001171752 |
| *NRG2* | 1.268818868 | 0.585578571 | -1.115551464 | 0.000324357 | 0.001171752 |
| *BRS3* | 0.247581761 | 0.022567857 | -3.455563693 | 0.000325562 | 0.001175581 |
| *AC002451.1* | 0.136683333 | 0.060314286 | -1.18026568 | 0.000325858 | 0.001176475 |
| *RPSAP1* | 0.312050943 | 0.141919048 | -1.13671334 | 0.000326247 | 0.001177706 |
| *RNASEH2B-AS1* | 0.241006289 | 0.11302619 | -1.092413682 | 0.000326982 | 0.001180012 |
| *RPSAP72* | 0.412592138 | 0.156988095 | -1.394061172 | 0.000328429 | 0.001184365 |
| *NUDT19P3* | 0.169838679 | 0.06520119 | -1.381194846 | 0.000330374 | 0.001190334 |
| *SULT1E1* | 8.829516038 | 3.198945238 | -1.464738068 | 0.000331529 | 0.00119378 |
| *SLC39A5* | 0.86613239 | 0.330829762 | -1.388498533 | 0.000332999 | 0.001197397 |
| *CADM2* | 0.313766038 | 0.107732143 | -1.542240447 | 0.000333862 | 0.001200265 |
| *AC007786.2* | 0.187753774 | 0.087538095 | -1.100859007 | 0.000333969 | 0.001200472 |
| *RPL35P6* | 0.307939937 | 0.128280952 | -1.263342015 | 0.000335933 | 0.001206649 |
| *GBX2* | 0.181795912 | 0.416996429 | 1.19771527 | 0.000337169 | 0.001209678 |
| *HYDIN2* | 0.129761635 | 0.059494048 | -1.125046666 | 0.000337738 | 0.001211436 |
| *AL139423.2* | 0.222788365 | 0.087186905 | -1.353490522 | 0.000337942 | 0.00121192 |
| *AC138409.1* | 0.502517925 | 0.070542857 | -2.832603143 | 0.000340026 | 0.001218328 |
| *ROS1* | 0.732507862 | 3.460464286 | 2.240049467 | 0.00034064 | 0.001219992 |
| *KCNK15-AS1* | 0.606102516 | 0.262164286 | -1.209090668 | 0.000342668 | 0.001226366 |
| *AL137785.1* | 2.111027044 | 0.960388095 | -1.136255635 | 0.000346977 | 0.00123872 |
| *AC124067.2* | 5.120481132 | 2.420915476 | -1.080726666 | 0.000348815 | 0.001244559 |
| *DCBLD2* | 13.56139906 | 35.15360476 | 1.374166614 | 0.0003524 | 0.001256078 |
| *AC011939.1* | 0.274257233 | 0.106989286 | -1.358063341 | 0.000355464 | 0.001265578 |
| *AC127024.3* | 0.407056918 | 0.151670238 | -1.424292522 | 0.000356709 | 0.001269412 |
| *AL117339.4* | 0.261195283 | 0.125419048 | -1.058372374 | 0.000358304 | 0.00127398 |
| *CORO6* | 3.718456918 | 8.060128571 | 1.116098795 | 0.000361754 | 0.00128532 |
| *AC093844.1* | 0.137727673 | 0.524894048 | 1.930207775 | 0.0003619 | 0.00128565 |
| *C10orf67* | 0.29918239 | 0.60219881 | 1.009214596 | 0.000363284 | 0.001289824 |
| *AC103563.7* | 2.274436478 | 0.592488095 | -1.940651071 | 0.00036587 | 0.001297318 |
| *AL136984.1* | 0.212962264 | 0.037708333 | -2.497642523 | 0.000365946 | 0.001297403 |

| *AC008180.3* | 0.131557547 | 0.033039286 | -1.993439613 | 0.00036856 | 0.001305353 |
| --- | --- | --- | --- | --- | --- |
| *AL513217.1* | 0.541885535 | 0.265902381 | -1.02709144 | 0.000368755 | 0.001305854 |
| *GBX1* | 0.771683333 | 0.342019048 | -1.173932274 | 0.000370336 | 0.001311265 |
| *AL022069.2* | 0.198066352 | 0.09862381 | -1.005975928 | 0.000371619 | 0.00131486 |
| *CTSL3P* | 0.710881447 | 0.303294048 | -1.228891798 | 0.000373697 | 0.001322022 |
| *BBIP1P1* | 0.85736195 | 0.305829762 | -1.487175581 | 0.000379012 | 0.001337396 |
| *NADK2-AS1* | 1.193941195 | 0.465009524 | -1.360399612 | 0.000379813 | 0.001339996 |
| *FAM3D-AS1* | 0.124755975 | 0.034958333 | -1.835400601 | 0.000383935 | 0.001352795 |
| *AC090515.5* | 0.282373899 | 0.111396429 | -1.341903762 | 0.000386934 | 0.001362188 |
| *AC004223.2* | 0.632265094 | 0.30220119 | -1.065020231 | 0.000388037 | 0.001365142 |
| *TRIM63* | 1.306860692 | 0.584834524 | -1.160004978 | 0.000390554 | 0.00137238 |
| *LINC00165* | 1.533375157 | 3.809838095 | 1.313018978 | 0.000391175 | 0.001373966 |
| *MAL2-AS1* | 0.697063522 | 0.228411905 | -1.609652287 | 0.000391988 | 0.001376435 |
| *ORM2* | 0.895614151 | 5.199897619 | 2.53753399 | 0.000393033 | 0.001379516 |
| *TCF15* | 4.117778302 | 1.653144048 | -1.316653718 | 0.000393572 | 0.001381013 |
| *C9orf24* | 1.409121069 | 0.691332143 | -1.027344662 | 0.00039675 | 0.001390972 |
| *AL031666.3* | 0.409607233 | 0.07610119 | -2.428250263 | 0.000400896 | 0.001404106 |
| *CENPUP2* | 0.26355566 | 0.073958333 | -1.833323058 | 0.000402631 | 0.00140938 |
| *AC103739.2* | 0.266832704 | 0.121221429 | -1.138290752 | 0.000405012 | 0.001417312 |
| *AL161669.2* | 4.441861321 | 1.887355952 | -1.234797813 | 0.000405437 | 0.001418598 |
| *NLGN4X* | 3.230346855 | 1.168490476 | -1.467043106 | 0.000406384 | 0.001421111 |
| *AC091806.1* | 0.188025157 | 0.069404762 | -1.437819148 | 0.000406728 | 0.001421902 |
| *AC022893.3* | 0.332467925 | 0.09617619 | -1.789463473 | 0.00040936 | 0.001429475 |
| *LINC02600* | 0.983060063 | 0.156432143 | -2.651742584 | 0.000410733 | 0.001433254 |
| *BRSK2* | 1.255531132 | 0.407089286 | -1.624880646 | 0.000418736 | 0.001456443 |
| *SYT10* | 0.374361321 | 0.125654762 | -1.574966037 | 0.000419284 | 0.001458128 |
| *FAM240C* | 0.661562893 | 0.311761905 | -1.085433665 | 0.000419399 | 0.001458321 |
| *ZNF730* | 0.911713522 | 0.441795238 | -1.045202704 | 0.000422244 | 0.001465934 |
| *PKDCC* | 6.753164465 | 13.87446786 | 1.038796841 | 0.000434027 | 0.001503869 |
| *RPL37P12* | 1.418923899 | 0.621361905 | -1.191291517 | 0.000434397 | 0.001504938 |
| *AL035448.1* | 0.430316352 | 0.170805952 | -1.333039412 | 0.000435264 | 0.001507305 |
| *SERHL* | 0.120980503 | 0.051127381 | -1.242606536 | 0.00043536 | 0.001507427 |
| *TRIM60P17* | 0.247122642 | 0.118867857 | -1.055868546 | 0.000440497 | 0.001523497 |
| *AC120193.1* | 0.07470566 | 0.25317381 | 1.760838704 | 0.00044127 | 0.001525957 |
| *UGT2B7* | 9.767739308 | 4.612088095 | -1.082604627 | 0.000443434 | 0.001532794 |
| *PDZK1* | 1.201707233 | 3.555190476 | 1.564841396 | 0.000443621 | 0.001533224 |
| *CYP2F1* | 0.724012264 | 0.094322619 | -2.940338452 | 0.000443697 | 0.001533271 |
| *IGFL2* | 7.30432956 | 14.93230476 | 1.031613095 | 0.000446636 | 0.001542776 |
| *MT-TV* | 1.838928302 | 0.733495238 | -1.326005727 | 0.000451262 | 0.001557006 |
| *AC107308.1* | 0.902640566 | 1.838292857 | 1.026143098 | 0.000451827 | 0.001558518 |
| *AL358232.1* | 1.043910377 | 0.183440476 | -2.508613948 | 0.000452682 | 0.001561029 |
| *AC006441.1* | 0.554594025 | 0.218971429 | -1.340689435 | 0.000454827 | 0.001567765 |
| *MAPK10-AS1* | 0.161479874 | 0.033279762 | -2.278637351 | 0.000459123 | 0.001581688 |
| *LINC00643* | 0.291459119 | 0.068741667 | -2.084036807 | 0.00046028 | 0.001585007 |
| *TMEM97P1* | 0.285731761 | 0.130060714 | -1.135476158 | 0.000460426 | 0.001585288 |
| *LCE1E* | 0.787772327 | 0.188203571 | -2.065484733 | 0.000461162 | 0.001587156 |

| *OR1L8* | 0.15149434 | 0.060315476 | -1.328663758 | 0.000468462 | 0.001609127 |
| --- | --- | --- | --- | --- | --- |
| *AC008731.1* | 0.748457862 | 0.368307143 | -1.023011718 | 0.000469464 | 0.001611895 |
| *CT62* | 0.194537421 | 1.048589286 | 2.430330105 | 0.000470164 | 0.001613398 |
| *ERVH48-1* | 15.1072956 | 4.786525 | -1.658194873 | 0.000474906 | 0.001626492 |
| *AL353583.1* | 0.339146855 | 0.060808333 | -2.479569163 | 0.000479615 | 0.001640791 |
| *AL035603.1* | 0.093676101 | 0.279575 | 1.57748243 | 0.000480726 | 0.001644134 |
| *PAK5* | 0.260125157 | 0.084408333 | -1.623748589 | 0.000486945 | 0.00166286 |
| *LINC01518* | 6.92772956 | 3.289828571 | -1.074370203 | 0.000492338 | 0.001678785 |
| *UGT1A8* | 7.345255031 | 2.462810714 | -1.576506833 | 0.000498761 | 0.001698261 |
| *IGHV7-27* | 0.294489623 | 0.699167857 | 1.247422065 | 0.000504296 | 0.001714969 |
| *MTND5P1* | 0.337383962 | 0.145894048 | -1.209470372 | 0.00050545 | 0.001718063 |
| *CHORDC2P* | 0.125624528 | 0.060479762 | -1.054593814 | 0.00050836 | 0.001726404 |
| *AP005264.1* | 1.176154403 | 3.166944048 | 1.429013913 | 0.000509073 | 0.001728345 |
| *C1QBPP2* | 0.181681761 | 0.086917857 | -1.063689083 | 0.000510885 | 0.001733303 |
| *SRD5A2* | 1.145267296 | 0.313309524 | -1.870023821 | 0.000515211 | 0.001746775 |
| *AL031719.1* | 0.124195912 | 0.049472619 | -1.327915504 | 0.000515371 | 0.001747076 |
| *WIF1* | 12.41424528 | 1.112880952 | -3.479625379 | 0.000515979 | 0.001748655 |
| *TEPP* | 5.016373899 | 2.047121429 | -1.293048204 | 0.000516993 | 0.001751126 |
| *AL117329.1* | 0.298257547 | 0.084786905 | -1.81464528 | 0.000517782 | 0.001753076 |
| *LTF* | 19.94498522 | 372.6208369 | 4.223610392 | 0.000520627 | 0.001761254 |
| *FAM149A* | 3.446404717 | 1.604344048 | -1.103108572 | 0.000520627 | 0.001761254 |
| *LRRTM3* | 0.377144654 | 0.134633333 | -1.486082331 | 0.000520766 | 0.00176148 |
| *ARHGEF2-AS1* | 0.775006918 | 0.345647619 | -1.164907201 | 0.000529199 | 0.00178583 |
| *SFTA1P* | 0.183600943 | 0.461838095 | 1.330813709 | 0.000530769 | 0.001790637 |
| *IL5RA* | 0.082328302 | 0.175602381 | 1.092852032 | 0.00053286 | 0.001796949 |
| *AP005212.2* | 0.903989623 | 0.448357143 | -1.01165783 | 0.000535829 | 0.001806468 |
| *AC008737.1* | 0.704803774 | 0.313889286 | -1.166965864 | 0.000538648 | 0.001815474 |
| *LRRC37A14P* | 0.257113208 | 0.12282619 | -1.065785499 | 0.000539635 | 0.001817556 |
| *AC022137.3* | 0.89032044 | 0.420504762 | -1.08220254 | 0.000547689 | 0.00184228 |
| *SERPINB11* | 1.315633648 | 0.123852381 | -3.409064303 | 0.000548211 | 0.00184316 |
| *AL135786.1* | 0.406326101 | 0.929919048 | 1.194467091 | 0.000551179 | 0.001852629 |
| *SLC7A14-AS1* | 0.184086164 | 0.057113095 | -1.688487715 | 0.000552833 | 0.001856921 |
| *AC009927.1* | 0.160640252 | 0.071109524 | -1.175718733 | 0.00055783 | 0.001871151 |
| *LUARIS* | 0.162081132 | 0.369313095 | 1.188128263 | 0.000560356 | 0.001878856 |
| *AC110741.1* | 2.216526101 | 0.566832143 | -1.967306876 | 0.000561493 | 0.001882411 |
| *LINC02057* | 0.167906289 | 0.501138095 | 1.577551941 | 0.000562967 | 0.001887095 |
| *OR2A13P* | 0.230814465 | 0.108984524 | -1.08261036 | 0.000563772 | 0.001889277 |
| *AC112206.2* | 0.148327987 | 0.059053571 | -1.328694622 | 0.000564158 | 0.001890168 |
| *AC139453.2* | 0.906521698 | 0.314292857 | -1.528232067 | 0.000568837 | 0.001904435 |
| *AC011297.1* | 3.033270755 | 1.161996429 | -1.384268646 | 0.000582037 | 0.001942281 |
| *AC022463.1* | 0.455589623 | 0.117967857 | -1.949341066 | 0.000585917 | 0.001954699 |
| *ZSCAN5DP* | 0.393338679 | 0.122266667 | -1.685740924 | 0.000587243 | 0.001958326 |
| *AC003092.1* | 0.186706604 | 0.725427381 | 1.958058244 | 0.000589847 | 0.001965438 |
| *SLC5A4-AS1* | 0.181339308 | 0.058619048 | -1.629250252 | 0.000602502 | 0.002002694 |
| *AF106564.1* | 0.33746761 | 0.096457143 | -1.806789058 | 0.000609452 | 0.002022515 |
| *AC092634.3* | 0.202832704 | 0.084179762 | -1.268744955 | 0.000613401 | 0.00203315 |

| *SLC25A39P1* | 0.230281447 | 0.044703571 | -2.36493618 | 0.000615211 | 0.002038051 |
| --- | --- | --- | --- | --- | --- |
| *ASXL3* | 0.397346226 | 0.111857143 | -1.828739258 | 0.000622945 | 0.002058711 |
| *KRR1P1* | 1.124254088 | 0.503766667 | -1.15814056 | 0.000627014 | 0.00207046 |
| *AC105389.2* | 0.512309434 | 0.18657381 | -1.457268978 | 0.000628363 | 0.002073245 |
| *AC104335.2* | 0.099722013 | 0.267142857 | 1.421627538 | 0.000629664 | 0.002076701 |
| *B4GALNT2* | 0.244512579 | 0.89975 | 1.879615513 | 0.000638444 | 0.002103684 |
| *SOX2-OT* | 0.410388994 | 0.059838095 | -2.777855882 | 0.000639004 | 0.002105057 |
| *AMACR* | 1.997465723 | 0.878878571 | -1.18443499 | 0.000641512 | 0.002112095 |
| *SBSPON* | 7.939290566 | 3.704982143 | -1.099543509 | 0.000642758 | 0.002115346 |
| *CYP4F35P* | 1.012060377 | 0.221394048 | -2.192607021 | 0.000662909 | 0.002174681 |
| *IRS4* | 1.002578302 | 0.031827381 | -4.977302662 | 0.000665642 | 0.002182773 |
| *GAS6-DT* | 1.318339937 | 2.802742857 | 1.088116967 | 0.000679799 | 0.00222545 |
| *MTATP6P27* | 0.358163836 | 0.117289286 | -1.610548447 | 0.000679833 | 0.00222545 |
| *AC011447.6* | 0.598600314 | 0.274369048 | -1.125475301 | 0.000681217 | 0.002229387 |
| *TESC* | 48.39939686 | 14.5340369 | -1.735553594 | 0.000689063 | 0.002252067 |
| *AC011451.2* | 0.529926415 | 0.263597619 | -1.007454704 | 0.000696375 | 0.002273551 |
| *AC023090.2* | 0.120740252 | 0.052347619 | -1.205710887 | 0.000706463 | 0.002299964 |
| *DNAH11* | 0.247248742 | 0.554729762 | 1.165821948 | 0.000708181 | 0.002305058 |
| *AC005072.1* | 0.131853774 | 0.030528571 | -2.110706875 | 0.000712876 | 0.002318191 |
| *CRISP2* | 0.680840252 | 0.157670238 | -2.11040597 | 0.000715865 | 0.002326549 |
| *CX3CL1* | 19.63724277 | 41.18224881 | 1.068430235 | 0.000721635 | 0.002341102 |
| *HAPLN2* | 0.273696541 | 0.089377381 | -1.614595526 | 0.000724111 | 0.002348515 |
| *ARC* | 2.44462327 | 0.606279762 | -2.011556585 | 0.000734195 | 0.002374705 |
| *PCDH19* | 1.534196855 | 0.705095238 | -1.121593567 | 0.000735619 | 0.002378329 |
| *AC022092.1* | 0.143027044 | 0.332003571 | 1.214910799 | 0.000737167 | 0.002383004 |
| *CDC42-IT1* | 0.575066667 | 0.202839286 | -1.503392116 | 0.000739613 | 0.002388397 |
| *AL356113.1* | 0.178797484 | 0.030919048 | -2.531758651 | 0.000746238 | 0.002407265 |
| *AC079140.4* | 0.180598428 | 0.08262619 | -1.128114274 | 0.000753469 | 0.002427723 |
| *AC084030.1* | 0.264098428 | 0.094646429 | -1.48045574 | 0.000763954 | 0.002457645 |
| *AC090503.2* | 0.347535849 | 0.171629762 | -1.017862054 | 0.000767323 | 0.002466868 |
| *SKOR1* | 0.61206761 | 0.257483333 | -1.249211973 | 0.00077466 | 0.002487528 |
| *TEKT3* | 0.390760063 | 0.182640476 | -1.0972765 | 0.000779595 | 0.002500438 |
| *AC009185.1* | 0.13691761 | 0.061729762 | -1.149269883 | 0.000780245 | 0.002502197 |
| *AC100774.1* | 0.14171761 | 0.040315476 | -1.813613373 | 0.000795153 | 0.002545026 |
| *BSN* | 0.439589308 | 0.186803571 | -1.234634259 | 0.000800214 | 0.002558256 |
| *AL034351.4* | 0.199078931 | 0.078990476 | -1.333589919 | 0.000804084 | 0.002567933 |
| *MYADML2* | 0.161454403 | 0.059510714 | -1.439905442 | 0.000806772 | 0.002575179 |
| *TMEM191B* | 1.093777358 | 0.438867857 | -1.317460588 | 0.000812144 | 0.002591312 |
| *LCMT1-AS2* | 0.40608522 | 0.029384524 | -3.788654096 | 0.000813924 | 0.00259513 |
| *NPIPB15* | 10.46777987 | 5.043160714 | -1.053555385 | 0.000813966 | 0.00259513 |
| *GPIHBP1* | 2.391798742 | 1.096953571 | -1.124593534 | 0.000815613 | 0.002598697 |
| *MAL* | 117.9666252 | 26.1380119 | -2.174157436 | 0.000815619 | 0.002598697 |
| *THRSP* | 0.370402201 | 13.41855357 | 5.178992685 | 0.000818764 | 0.002607704 |
| *USH2A* | 0.376100314 | 0.023485714 | -4.001262136 | 0.000820147 | 0.002610757 |
| *AL355922.4* | 1.07176761 | 0.222421429 | -2.26862443 | 0.000827277 | 0.002630049 |
| *LRRC18* | 0.187882704 | 0.055410714 | -1.761595395 | 0.000827953 | 0.002631076 |

| *ANXA10* | 72.43402673 | 19.94001667 | -1.861000964 | 0.000841996 | 0.002669938 |
| --- | --- | --- | --- | --- | --- |
| *AC007314.1* | 0.304157547 | 0.14742619 | -1.044825959 | 0.000848938 | 0.002689871 |
| *GDAP1L1* | 0.526996226 | 0.049834524 | -3.402575183 | 0.000850064 | 0.00269128 |
| *AC092658.1* | 0.126139308 | 0.046203571 | -1.448941648 | 0.000860203 | 0.002716828 |
| *DSG4* | 0.964768868 | 0.223635714 | -2.109032751 | 0.000866179 | 0.002733233 |
| *AC108519.1* | 0.456936478 | 0.206294048 | -1.147291425 | 0.000869865 | 0.002743323 |
| *AC023158.2* | 0.965446855 | 0.449930952 | -1.101493227 | 0.000888485 | 0.002793224 |
| *MS4A15* | 5.030522013 | 2.015344048 | -1.319681967 | 0.000892021 | 0.002802191 |
| *HORMAD2-AS1* | 0.130195283 | 0.063940476 | -1.025875787 | 0.000894387 | 0.00280855 |
| *EPGN* | 4.095861006 | 11.09776548 | 1.438030557 | 0.000894716 | 0.002808958 |
| *AL023754.1* | 0.265941195 | 0.883044048 | 1.731378132 | 0.000894928 | 0.002808958 |
| *FO681548.1* | 0.118186164 | 0.036357143 | -1.70075041 | 0.000896996 | 0.002813507 |
| *KCNH8* | 0.945383962 | 0.311563095 | -1.601376035 | 0.000898725 | 0.002818573 |
| *ZSCAN1* | 0.81926761 | 0.217210714 | -1.91523951 | 0.000902055 | 0.002827574 |
| *WFDC21P* | 69.37599623 | 23.91801905 | -1.536338677 | 0.000907047 | 0.002841771 |
| *LINC01910* | 0.159475472 | 0.454252381 | 1.510159531 | 0.000908395 | 0.002845633 |
| *SGSM3-AS1* | 0.193733333 | 0.059571429 | -1.701379741 | 0.000917545 | 0.002871004 |
| *AC104794.4* | 0.713285849 | 0.277496429 | -1.362011148 | 0.000918247 | 0.002872836 |
| *AL139246.4* | 1.57716478 | 0.557445238 | -1.500431408 | 0.00091946 | 0.0028759 |
| *THRA1/BTR* | 0.30815 | 0.035760714 | -3.107185333 | 0.000920828 | 0.002879537 |
| *AC015910.1* | 0.378345283 | 0.053914286 | -2.810963958 | 0.0009262 | 0.002892933 |
| *AC006262.4* | 2.288805346 | 0.600358333 | -1.930699014 | 0.000952124 | 0.00296187 |
| *CFAP57* | 0.598039623 | 1.324796429 | 1.147457711 | 0.000959843 | 0.002981745 |
| *AC026464.5* | 0.174940252 | 0.047728571 | -1.873937213 | 0.000960078 | 0.002982086 |
| *MIR133A1HG* | 0.131235535 | 0.024625 | -2.413962781 | 0.000962288 | 0.002988572 |
| *APOBEC2* | 0.585858805 | 0.171647619 | -1.771103164 | 0.000972541 | 0.003017367 |
| *ATP6V1B1* | 0.226557547 | 0.095342857 | -1.248680787 | 0.000973041 | 0.003018538 |
| *AL157834.1* | 0.284115723 | 0.121883333 | -1.220977813 | 0.000974007 | 0.003021154 |
| *ATP2B3* | 0.188487107 | 0.022435714 | -3.070596821 | 0.000998921 | 0.003089857 |
| *CKMT2* | 1.214042138 | 0.533495238 | -1.186271198 | 0.001007891 | 0.00311409 |
| *LINC01511* | 0.162743396 | 0.041786905 | -1.961476199 | 0.001009313 | 0.003117691 |
| *AL445183.2* | 0.286518553 | 0.126452381 | -1.180034362 | 0.001030577 | 0.003175395 |
| *LINC01505* | 0.133626415 | 0.06545 | -1.029740129 | 0.001031275 | 0.003177148 |
| *AC090950.1* | 0.19715 | 0.034858333 | -2.499718213 | 0.001042047 | 0.003207121 |
| *COL17A1* | 59.33391164 | 150.4535821 | 1.342389654 | 0.001058161 | 0.00325102 |
| *CFAP47* | 0.785074843 | 0.302884524 | -1.374062332 | 0.001061051 | 0.003259085 |
| *B4GALT4-AS1* | 0.223298113 | 0.0855 | -1.384974736 | 0.001068792 | 0.003279584 |
| *AC024592.2* | 0.385123899 | 0.185579762 | -1.053283266 | 0.001074521 | 0.003293467 |
| *NPIPA3* | 0.360957547 | 0.176896429 | -1.028924248 | 0.00108191 | 0.00330884 |
| *EDRF1-AS1* | 0.190142453 | 0.083783333 | -1.182345488 | 0.001082228 | 0.00330884 |
| *AP000344.1* | 0.668103459 | 0.159582143 | -2.065772305 | 0.001127287 | 0.003432948 |
| *TMPRSS11E* | 28.15615346 | 13.32085714 | -1.079763337 | 0.001135591 | 0.003454628 |
| *PPP1R8P1* | 0.119855975 | 0.051766667 | -1.2112065 | 0.001141252 | 0.003467749 |
| *ZNF626* | 9.732058176 | 3.869935714 | -1.330435343 | 0.001152815 | 0.003498128 |
| *LINC01971* | 0.313178616 | 0.133234524 | -1.233017747 | 0.001156849 | 0.003509504 |
| *RPS5P2* | 0.249214151 | 0.095936905 | -1.377228191 | 0.001162546 | 0.003524612 |

| *BRWD1-AS1* | 0.323515094 | 0.131871429 | -1.294701004 | 0.001172914 | 0.003553418 |
| --- | --- | --- | --- | --- | --- |
| *AC092881.1* | 0.431198428 | 0.076605952 | -2.492823517 | 0.001173136 | 0.003553651 |
| *RNU6-1223P* | 2.513880818 | 1.219291667 | -1.043872979 | 0.0011741 | 0.003555295 |
| *MTND2P40* | 0.33339434 | 0.108190476 | -1.623656103 | 0.001178783 | 0.003566283 |
| *INHCAP* | 0.192971384 | 0.080463095 | -1.26198778 | 0.001180006 | 0.003569189 |
| *PSORS1C3* | 14.73214717 | 5.610927381 | -1.392656568 | 0.001186326 | 0.0035861 |
| *SNX18P13* | 0.792612264 | 0.249238095 | -1.669090694 | 0.001190755 | 0.003596394 |
| *UGT1A10* | 29.37951887 | 12.68035 | -1.212216203 | 0.001208292 | 0.003643542 |
| *AC008514.1* | 0.314545597 | 1.391445238 | 2.145243056 | 0.00120943 | 0.003645605 |
| *AC020978.1* | 0.32465 | 0.148314286 | -1.130227644 | 0.001211153 | 0.003649485 |
| *AC051619.10* | 0.119238679 | 0.039242857 | -1.603350312 | 0.001212587 | 0.003652463 |
| *SCUBE3* | 3.142699371 | 1.225504762 | -1.358628181 | 0.001216445 | 0.00366095 |
| *LK* *AEAR1* | 0.827987107 | 0.343758333 | -1.268213615 | 0.001235468 | 0.003711383 |
| *FSTL3* | 46.40569371 | 94.00579167 | 1.018447817 | 0.001236845 | 0.003714614 |
| *AC073648.7* | 0.16412673 | 0.056327381 | -1.542901917 | 0.001239645 | 0.003721204 |
| *PAX7* | 0.70956195 | 0.18864881 | -1.911225651 | 0.001261218 | 0.003777664 |
| *CYP3A43* | 0.236458176 | 0.057046429 | -2.051376554 | 0.001264436 | 0.003785752 |
| *SLC35F1* | 0.565403459 | 0.206345238 | -1.454220564 | 0.001278577 | 0.003822256 |
| *BNIP3P27* | 1.204304717 | 0.582405952 | -1.048103468 | 0.00129749 | 0.003871901 |
| *ORM1* | 2.131913836 | 9.190578571 | 2.108006555 | 0.001303842 | 0.003887814 |
| *AL354751.3* | 0.189722013 | 0.049761905 | -1.930773463 | 0.001307659 | 0.003896833 |
| *AL354714.1* | 0.232284906 | 0.106925 | -1.119296201 | 0.001322575 | 0.003936038 |
| *VCX3B* | 0.748962579 | 0.089034524 | -3.072456872 | 0.001335202 | 0.003967854 |
| *AC011411.1* | 0.171052201 | 0.064713095 | -1.402307082 | 0.001341765 | 0.003982546 |
| *CYP51A1P3* | 0.127678616 | 0.033830952 | -1.916101226 | 0.001351985 | 0.004008526 |
| *AC019257.1* | 0.162046541 | 0.062269048 | -1.379821106 | 0.001357944 | 0.004024739 |
| *AC007666.2* | 0.117516352 | 0.047633333 | -1.302818104 | 0.001359354 | 0.004028431 |
| *AC138904.1* | 1.565714465 | 0.67679881 | -1.210022201 | 0.001377637 | 0.004074266 |
| *CST5* | 3.147740566 | 0.743741667 | -2.081443136 | 0.001383218 | 0.00408684 |
| *AL132765.1* | 0.309961006 | 0.092353571 | -1.746847075 | 0.001384288 | 0.004089512 |
| *BNIP3P24* | 0.993614465 | 0.374788095 | -1.406611049 | 0.001386701 | 0.004095164 |
| *LINC02516* | 0.293092767 | 0.129109524 | -1.182761941 | 0.001413615 | 0.004165143 |
| *AC007743.1* | 0.466901258 | 0.174839286 | -1.417088086 | 0.001419102 | 0.004177306 |
| *SLC10A1* | 3.049465409 | 0.3595 | -3.084492675 | 0.001437662 | 0.004226235 |
| *AL121761.1* | 2.394769497 | 0.677535714 | -1.821517898 | 0.001439281 | 0.004230123 |
| *AL157834.4* | 0.452928616 | 0.215838095 | -1.069334171 | 0.001456497 | 0.004275612 |
| *DUXB* | 0.116432075 | 0.046184524 | -1.334007158 | 0.001472417 | 0.004317709 |
| *AC106038.1* | 0.205991195 | 0.09877619 | -1.060347437 | 0.001487917 | 0.004357966 |
| *AC116021.1* | 0.208707233 | 0.429140476 | 1.039969384 | 0.001488762 | 0.00435992 |
| *CDRT15P9* | 1.304291509 | 0.429240476 | -1.603408318 | 0.001494018 | 0.004372709 |
| *AL109614.1* | 4.107794969 | 1.926727381 | -1.09221172 | 0.0015074 | 0.004404387 |
| *AL133444.1* | 0.191183648 | 0.036091667 | -2.405221461 | 0.001508006 | 0.004405263 |
| *C8orf34-AS1* | 0.137680818 | 0.068484524 | -1.007477661 | 0.001523755 | 0.004446514 |
| *UNC5CL* | 3.135409434 | 1.456747619 | -1.105902895 | 0.001526827 | 0.004454732 |
| *GRIK3* | 0.314786164 | 0.071908333 | -2.130141251 | 0.001532878 | 0.004468366 |
| *PPIAP90* | 0.260298113 | 0.110509524 | -1.235994147 | 0.001561096 | 0.004542546 |

| *FCER1A* | 4.702290881 | 2.240852381 | -1.069316173 | 0.001562994 | 0.004545676 |
| --- | --- | --- | --- | --- | --- |
| *FAT3* | 0.716698428 | 0.27404881 | -1.386933322 | 0.001563199 | 0.004545676 |
| *CELF5* | 0.505765094 | 0.116934524 | -2.112766537 | 0.001587905 | 0.004604756 |
| *AL358075.1* | 0.272722642 | 0.128942857 | -1.080702622 | 0.001612508 | 0.004666749 |
| *AC062028.1* | 0.301191824 | 0.076155952 | -1.9836539 | 0.001646935 | 0.004753518 |
| *AC007684.1* | 1.310327044 | 0.341678571 | -1.939215262 | 0.001652734 | 0.004764771 |
| *STYXL2* | 3.456487421 | 0.759647619 | -2.185904426 | 0.001652771 | 0.004764771 |
| *SCHLAP1* | 1.953575786 | 0.8459 | -1.207558197 | 0.001654491 | 0.004769169 |
| *CRYZP1* | 0.627691509 | 0.29069881 | -1.110530531 | 0.001682375 | 0.004841602 |
| *AL671883.1* | 1.205366667 | 0.533169048 | -1.17680714 | 0.001707217 | 0.00490105 |
| *NNAT* | 116.074528 | 5.16015119 | -4.491494267 | 0.001711073 | 0.004910401 |
| *SOSTDC1* | 4.164743082 | 1.509864286 | -1.463808622 | 0.001716941 | 0.004926666 |
| *AC026111.1* | 0.14951478 | 0.041813095 | -1.838261356 | 0.001741206 | 0.004989307 |
| *AL669831.7* | 0.150621384 | 0.036560714 | -2.042560442 | 0.001745061 | 0.004996861 |
| *MYOCD* | 1.829472013 | 0.850808333 | -1.104521276 | 0.001757845 | 0.005029366 |
| *NDUFA9P1* | 0.40447956 | 0.160571429 | -1.33285159 | 0.001796514 | 0.005129859 |
| *AL442636.1* | 0.244250943 | 0.100942857 | -1.274825307 | 0.001799431 | 0.005135802 |
| *AC006270.1* | 2.263543396 | 0.718770238 | -1.654980388 | 0.001800028 | 0.005136909 |
| *RPL7L1P3* | 0.574222327 | 0.132021429 | -2.120837313 | 0.001809057 | 0.005161479 |
| *AC064807.2* | 1.453140881 | 0.394260714 | -1.881952711 | 0.001830021 | 0.005212222 |
| *PSAT1P1* | 0.152774214 | 0.052266667 | -1.547437998 | 0.001861481 | 0.005290184 |
| *AP003555.2* | 1.297872956 | 0.414216667 | -1.647691661 | 0.001862243 | 0.005291129 |
| *GS1-279B7.1* | 0.147005346 | 0.038854762 | -1.919705294 | 0.001889827 | 0.005360214 |
| *DPH6-DT* | 0.140621069 | 0.318935714 | 1.18145289 | 0.00191897 | 0.005429774 |
| *SMILR* | 3.87403805 | 1.714497619 | -1.176052223 | 0.001924041 | 0.005442819 |
| *TAFA3* | 0.638095283 | 2.111035714 | 1.726107212 | 0.001924652 | 0.00544392 |
| *AC107918.4* | 0.425077673 | 0.18795 | -1.177377569 | 0.001935519 | 0.005468632 |
| *WNT5B* | 10.63353899 | 24.10886786 | 1.180942078 | 0.001953498 | 0.005510304 |
| *CTBP2P8* | 0.420540252 | 0.204947619 | -1.036988663 | 0.001969698 | 0.005551541 |
| *AL354794.2* | 0.150283648 | 0.069507143 | -1.112454892 | 0.001971128 | 0.005553664 |
| *AC018552.3* | 0.294930189 | 0.088144048 | -1.74243845 | 0.001984499 | 0.005584938 |
| *FLJ42393* | 0.319002516 | 0.074115476 | -2.105721071 | 0.00199779 | 0.005618486 |
| *AC083964.2* | 0.217130818 | 0.095272619 | -1.188430949 | 0.002013514 | 0.005656885 |
| *AC002044.3* | 0.574264151 | 0.091458333 | -2.650527969 | 0.0020194 | 0.005669535 |
| *ZACN* | 0.165510692 | 0.077947619 | -1.086347556 | 0.002032776 | 0.00570058 |
| *DDX11L10* | 0.434327358 | 0.136052381 | -1.674620627 | 0.00203374 | 0.005702634 |
| *MARCHF4* | 0.810769497 | 3.278975 | 2.015881185 | 0.00204001 | 0.005718911 |
| *AP001207.2* | 0.23382956 | 0.069570238 | -1.748915158 | 0.002046162 | 0.005732236 |
| *AL138960.1* | 0.45641478 | 0.168320238 | -1.43913686 | 0.002056696 | 0.005757491 |
| *AC025430.1* | 0.392061635 | 0.164992857 | -1.248676907 | 0.002065094 | 0.005777377 |
| *MTCYBP21* | 0.24305283 | 0.074069048 | -1.714327241 | 0.002067652 | 0.005781619 |
| *SST* | 27.70913082 | 0.497852381 | -5.798499617 | 0.002104935 | 0.005869474 |
| *AC098869.2* | 0.750149686 | 0.348932143 | -1.104231999 | 0.002108936 | 0.005879964 |
| *AC009563.1* | 0.131036478 | 0.049920238 | -1.392271765 | 0.002123018 | 0.005912525 |
| *AC021218.1* | 0.539970126 | 0.184475 | -1.549454275 | 0.002136402 | 0.005944413 |
| *MTCYBP35* | 0.119619182 | 0.049463095 | -1.274024335 | 0.002141132 | 0.005955554 |

| *HSD3BP4* | 0.121678616 | 0.052140476 | -1.222599989 | 0.002156968 | 0.005993501 |
| --- | --- | --- | --- | --- | --- |
| *CKB* | 144.893955 | 62.91951071 | -1.20341805 | 0.002165127 | 0.006014813 |
| *AC025918.1* | 0.424034906 | 0.171604762 | -1.305093442 | 0.002182287 | 0.006054277 |
| *UNC93B6* | 0.293795912 | 0.124821429 | -1.234948693 | 0.002228592 | 0.006168823 |
| *DPY19L2P4* | 0.255187421 | 0.122394048 | -1.060023821 | 0.002229222 | 0.006169871 |
| *AC018628.2* | 0.303585535 | 0.047982143 | -2.661533556 | 0.002248993 | 0.006222492 |
| *AC022217.2* | 0.090856604 | 0.191392857 | 1.074873705 | 0.002262641 | 0.006253992 |
| *AC106845.1* | 0.30581761 | 0.151871429 | -1.009821002 | 0.00230217 | 0.006343929 |
| *KRT13* | 1332.653686 | 564.8166726 | -1.238447338 | 0.002323108 | 0.006395178 |
| *CT69* | 0.494110063 | 2.906936905 | 2.556595415 | 0.002330377 | 0.006412316 |
| *AJ011932.1* | 0.589493082 | 1.565803571 | 1.409356452 | 0.002363781 | 0.006485846 |
| *IFITM3P9* | 1.256941509 | 2.923044048 | 1.217554053 | 0.002368128 | 0.006495845 |
| *AC006254.2* | 0.395158491 | 0.177707143 | -1.152929736 | 0.002378895 | 0.006520289 |
| *FGF2* | 1.614501572 | 3.656704762 | 1.179455304 | 0.002380849 | 0.006524194 |
| *Z84478.1* | 0.212508176 | 0.034020238 | -2.643053206 | 0.002388424 | 0.006541259 |
| *PRSS2* | 97.40638459 | 35.74850238 | -1.446133534 | 0.002389004 | 0.006541259 |
| *LINC01048* | 0.184810377 | 0.431153571 | 1.222156062 | 0.002397573 | 0.006561244 |
| *ADRA2C* | 8.210044969 | 2.43584881 | -1.752965535 | 0.002401776 | 0.006571284 |
| *AC005757.1* | 0.306424214 | 0.126508333 | -1.276297883 | 0.002405924 | 0.006577711 |
| *DEFB131E* | 0.242310692 | 0.081435714 | -1.57312452 | 0.002406653 | 0.006578042 |
| *Z99755.1* | 0.508721698 | 0.201590476 | -1.335449147 | 0.002411095 | 0.006587988 |
| *AC018761.3* | 0.245822642 | 0.119664286 | -1.038625163 | 0.002431798 | 0.006634972 |
| *AC069431.2* | 0.219849686 | 0.037778571 | -2.540877416 | 0.002433205 | 0.006638076 |
| *AC010287.1* | 0.312185849 | 0.09912619 | -1.655066951 | 0.002435664 | 0.006644045 |
| *LINC02860* | 0.317643396 | 0.044842857 | -2.824457919 | 0.002454033 | 0.006686736 |
| *AC104041.1* | 0.721922642 | 0.351816667 | -1.037020422 | 0.002462951 | 0.00670732 |
| *AC092117.2* | 0.118250629 | 0.042885714 | -1.463278802 | 0.002496487 | 0.006785876 |
| *MUC21* | 3.067658491 | 0.36319881 | -3.078306504 | 0.002501891 | 0.006799063 |
| *LINC02766* | 0.510270126 | 0.165919048 | -1.620781662 | 0.002502612 | 0.006800269 |
| *RPS15AP6* | 0.523954403 | 0.2042 | -1.3594584 | 0.002528142 | 0.006861303 |
| *LINC01645* | 0.388227358 | 0.139969048 | -1.47179396 | 0.00253389 | 0.006875384 |
| *FGF12* | 3.123307547 | 1.221890476 | -1.353959661 | 0.002562042 | 0.006943365 |
| *TLX1* | 2.026359434 | 0.452921429 | -2.161557398 | 0.00259531 | 0.007024988 |
| *PCSK2* | 0.607088679 | 0.06942381 | -3.128404833 | 0.002653028 | 0.007156019 |
| *AC012354.1* | 0.165909748 | 0.0488 | -1.765445605 | 0.002679167 | 0.007214655 |
| *SLC26A7* | 0.347530818 | 0.138177381 | -1.330619444 | 0.002713332 | 0.007291487 |
| *AL391840.2* | 0.351682075 | 0.1292 | -1.444665736 | 0.002728342 | 0.007325421 |
| *MMP7* | 78.5338066 | 159.358775 | 1.02089273 | 0.002736767 | 0.007344832 |
| *AC036103.1* | 0.328785535 | 0.15350119 | -1.098896982 | 0.00274233 | 0.007357355 |
| *RNU7-3P* | 1.617400943 | 0.773745238 | -1.063746827 | 0.002756621 | 0.007389245 |
| *KRTAP1-5* | 1.216158176 | 0.12920119 | -3.234639613 | 0.002758895 | 0.00739373 |
| *RNA5SP78* | 1.200281447 | 0.579019048 | -1.051690021 | 0.002783981 | 0.007451217 |
| *AC000120.1* | 0.344398742 | 0.164755952 | -1.063749285 | 0.002812913 | 0.007518864 |
| *AC020910.4* | 0.196101572 | 0.062563095 | -1.648217308 | 0.002814388 | 0.007520323 |
| *CHRM2* | 0.992434277 | 0.487419048 | -1.025808933 | 0.002902566 | 0.007722384 |
| *GPRACR* | 0.082756918 | 0.255220238 | 1.624790907 | 0.002924437 | 0.007770484 |

| *NOVA1* | 0.634146541 | 0.269008333 | -1.237165397 | 0.002947547 | 0.007825125 |
| --- | --- | --- | --- | --- | --- |
| *AL357140.4* | 0.27301195 | 0.123732143 | -1.14174377 | 0.002953436 | 0.007839068 |
| *JPH3* | 0.969334277 | 0.353778571 | -1.454147602 | 0.002966971 | 0.007872443 |
| *AC025031.5* | 0.185071069 | 0.042955952 | -2.107149423 | 0.002999202 | 0.00794254 |
| *AC007319.1* | 1.094938365 | 2.362029762 | 1.109177482 | 0.003023194 | 0.007996606 |
| *LINC02234* | 0.23580283 | 0.10824881 | -1.123229875 | 0.003030611 | 0.008011055 |
| *CHIAP3* | 0.156213522 | 0.048736905 | -1.680432805 | 0.003070475 | 0.008099019 |
| *AC087284.1* | 0.430910377 | 0.15275 | -1.496215462 | 0.003071102 | 0.008099805 |
| *POTEKP* | 0.432004088 | 0.122055952 | -1.823502311 | 0.003073226 | 0.00810367 |
| *ABCA6* | 2.40614717 | 1.097921429 | -1.131950073 | 0.003092668 | 0.008147075 |
| *AC011458.1* | 0.330226101 | 0.148130952 | -1.156581026 | 0.003131178 | 0.008226493 |
| *AC061975.1* | 0.837276101 | 0.288280952 | -1.538227929 | 0.003141801 | 0.008248234 |
| *RASSF10* | 6.254281447 | 12.7996131 | 1.033184153 | 0.003151362 | 0.00827245 |
| *AL136018.1* | 0.380273899 | 0.841917857 | 1.146640561 | 0.003163729 | 0.008300486 |
| *RNU6-125P* | 0.826954717 | 0.399775 | -1.048620076 | 0.003164852 | 0.008302547 |
| *POU3F3* | 0.932163208 | 0.033510714 | -4.797888228 | 0.003185684 | 0.008354524 |
| *AC005014.4* | 0.495030189 | 0.241489286 | -1.035557327 | 0.0031928 | 0.008370507 |
| *AC009478.1* | 0.389245597 | 0.175141667 | -1.152158376 | 0.003220095 | 0.008433077 |
| *AC083798.1* | 1.067158805 | 0.478063095 | -1.158501937 | 0.003248443 | 0.008498266 |
| *RPS15AP16* | 0.71871195 | 0.291161905 | -1.303592066 | 0.003250688 | 0.008501427 |
| *AC124862.1* | 0.135706918 | 0.060914286 | -1.155641754 | 0.003308899 | 0.008633467 |
| *GLRX5P2* | 1.15604434 | 0.443596429 | -1.381877077 | 0.003311339 | 0.008638 |
| *ACE2* | 0.234323899 | 0.485357143 | 1.050542617 | 0.003348347 | 0.008716973 |
| *AC022400.3* | 0.630568553 | 0.304363095 | -1.050859785 | 0.0033534 | 0.008725511 |
| *EMP2P1* | 0.445209748 | 0.207486905 | -1.101464897 | 0.003454192 | 0.008940476 |
| *NUTM1* | 0.252844969 | 0.516635714 | 1.030894308 | 0.00348795 | 0.009019309 |
| *PHBP19* | 0.468721384 | 0.23210119 | -1.013976691 | 0.003504111 | 0.009054435 |
| *AC087645.2* | 0.531878302 | 1.078280952 | 1.019565042 | 0.003556308 | 0.009176775 |
| *AC010378.1* | 20.21880723 | 4.705730952 | -2.103207148 | 0.003599247 | 0.009267158 |
| *AL590282.1* | 0.235208176 | 0.106809524 | -1.138897918 | 0.003613747 | 0.009299624 |
| *MYOC* | 0.92493805 | 0.238619048 | -1.954647531 | 0.00363127 | 0.009337877 |
| *AC006947.1* | 0.304795597 | 0.137097619 | -1.152638548 | 0.003633981 | 0.009343872 |
| *FDX1P1* | 0.336981132 | 0.15425119 | -1.127386192 | 0.003639226 | 0.009354422 |
| *RPL35AP5* | 1.071865409 | 0.5186 | -1.047429651 | 0.003661843 | 0.009406659 |
| *RPS6P12* | 0.606655346 | 0.283770238 | -1.096153836 | 0.003687937 | 0.009458873 |
| *BIRC6-AS2* | 0.44096478 | 0.166744048 | -1.40302817 | 0.003692894 | 0.009470597 |
| *DNAJB6P3* | 0.474013522 | 0.150086905 | -1.659130109 | 0.003693845 | 0.00947205 |
| *AL161669.4* | 0.438839308 | 1.138105952 | 1.374870209 | 0.003716656 | 0.0095226 |
| *MADD-AS1* | 0.33208522 | 0.131107143 | -1.340807228 | 0.003717918 | 0.009524842 |
| *TLX2* | 0.848193711 | 0.301430952 | -1.492566218 | 0.003758921 | 0.009616397 |
| *APLN* | 7.829528302 | 16.76901548 | 1.098800691 | 0.003772195 | 0.009645807 |
| *ACADL* | 1.129722956 | 0.507555952 | -1.154330244 | 0.003792913 | 0.009691731 |
| *AC105206.3* | 0.113366667 | 0.255452381 | 1.172057877 | 0.003795393 | 0.009696054 |
| *RNA5SP311* | 0.804994969 | 0.383427381 | -1.070026401 | 0.003796244 | 0.009697219 |
| *AL035252.5* | 0.191360377 | 0.062714286 | -1.609426122 | 0.003802386 | 0.009707867 |
| *AC009163.4* | 0.228816981 | 0.112478571 | -1.024543946 | 0.003813121 | 0.009731234 |

| *AC020661.3* | 0.347264151 | 0.12 | -1.532999079 | 0.003819993 | 0.009746748 |
| --- | --- | --- | --- | --- | --- |
| *AC108050.1* | 0.124974843 | 0.038453571 | -1.70044821 | 0.003835536 | 0.009780319 |
| *AC139769.3* | 1.88283522 | 0.89954881 | -1.065633276 | 0.00384293 | 0.009796124 |
| *PROKR1* | 0.172307862 | 0.032222619 | -2.418842861 | 0.003846568 | 0.009804384 |
| *RNU6-652P* | 1.806786164 | 0.765158333 | -1.239595552 | 0.003864251 | 0.009839258 |
| *RIMS4* | 1.022502201 | 0.121660714 | -3.071168664 | 0.003868963 | 0.009848196 |
| *AC010320.4* | 0.186092138 | 0.060965476 | -1.609952706 | 0.003875682 | 0.009861216 |
| *AC133561.1* | 0.351542453 | 0.067646429 | -2.377613248 | 0.003909263 | 0.009928171 |
| *HHLA2* | 0.096676101 | 0.247146429 | 1.35413487 | 0.003940631 | 0.009993388 |
| *CCDC187* | 0.33063522 | 0.094240476 | -1.810821678 | 0.003949881 | 0.010012715 |
| *RIPOR3-AS1* | 0.23267956 | 0.095113095 | -1.290628588 | 0.003955148 | 0.010021935 |
| *AC098679.4* | 0.117568553 | 0.048271429 | -1.2842608 | 0.003956613 | 0.010023582 |
| *PPP1R14D* | 18.44062862 | 3.996620238 | -2.206035438 | 0.003958764 | 0.010027998 |
| *AL133243.4* | 0.197112893 | 0.054777381 | -1.847369952 | 0.003989773 | 0.010090958 |
| *ERVV-1* | 2.102896226 | 0.815465476 | -1.366681953 | 0.004006582 | 0.010125343 |
| *LDC1P* | 0.229364465 | 0.056933333 | -2.010296423 | 0.004010006 | 0.010131713 |
| *LINC01143* | 0.623799371 | 0.307789286 | -1.019139086 | 0.004024722 | 0.010161587 |
| *MAGEA10* | 13.17874214 | 6.530402381 | -1.012968884 | 0.004025684 | 0.010162973 |
| *AC092844.1* | 0.228900629 | 0.109820238 | -1.059577483 | 0.004029604 | 0.010169737 |
| *AC009407.1* | 0.402505031 | 0.165844048 | -1.279179585 | 0.004039399 | 0.010189227 |
| *LMNTD1* | 0.126563522 | 0.043613095 | -1.537028365 | 0.004072004 | 0.010262001 |
| *AC061975.6* | 2.292741195 | 0.419257143 | -2.451166244 | 0.004076203 | 0.010268374 |
| *AC093525.9* | 0.131316981 | 0.064963095 | -1.015361211 | 0.004081232 | 0.010279452 |
| *KLHL41* | 0.566728931 | 0.281736905 | -1.008310297 | 0.004101644 | 0.01031947 |
| *AC006141.1* | 0.172695283 | 0.035883333 | -2.266842859 | 0.004114359 | 0.010347535 |
| *CLDN20* | 0.537426415 | 0.256452381 | -1.067376267 | 0.004147784 | 0.010416686 |
| *AC103810.5* | 0.400443396 | 0.162369048 | -1.30232169 | 0.004155615 | 0.010433157 |
| *H2BW1* | 0.948508491 | 0.358282143 | -1.404564548 | 0.004170748 | 0.010462051 |
| *CNNM1* | 0.778465094 | 0.370069048 | -1.072837877 | 0.004240691 | 0.010614244 |
| *TBC1D3E* | 0.114637736 | 0.053386905 | -1.102524208 | 0.004245856 | 0.010626089 |
| *MARCKSL1P1* | 0.628869497 | 0.254088095 | -1.307431877 | 0.004254378 | 0.010641774 |
| *ATP1A2* | 3.804580503 | 0.771982143 | -2.301098008 | 0.00427543 | 0.01068814 |
| *MTCO1P53* | 3.098415723 | 0.624053571 | -2.311788941 | 0.004349625 | 0.010846059 |
| *PTPRZ1* | 3.547931447 | 7.191960714 | 1.019407006 | 0.004364712 | 0.010879266 |
| *AL121652.1* | 0.919862893 | 0.305232143 | -1.591511946 | 0.004389699 | 0.010931576 |
| *GNG13* | 0.410462893 | 0.18042619 | -1.185843031 | 0.00439332 | 0.010937273 |
| *SDR42E2* | 0.187828931 | 0.089933333 | -1.062491447 | 0.00439934 | 0.010950042 |
| *AC124069.1* | 0.325554403 | 0.09715119 | -1.744595069 | 0.004404411 | 0.010961554 |
| *LMO3* | 2.817533019 | 1.148061905 | -1.295232082 | 0.004457636 | 0.011072748 |
| *IGSF9B* | 0.67303805 | 0.305834524 | -1.137936797 | 0.004487221 | 0.011132854 |
| *TMEM274P* | 0.249248113 | 0.086921429 | -1.519798793 | 0.00451367 | 0.0111927 |
| *CCNYL6* | 0.270633962 | 0.12127619 | -1.158046555 | 0.004545496 | 0.011260757 |
| *SCTR* | 0.46356478 | 0.181475 | -1.353000143 | 0.004556153 | 0.011282122 |
| *TM4SF4* | 2.286311006 | 0.614805952 | -1.894818628 | 0.004575646 | 0.011321785 |
| *SLC30A10* | 0.252276415 | 0.080982143 | -1.639329614 | 0.004624259 | 0.01141514 |
| *CYP2C18* | 3.179068553 | 0.953614286 | -1.737126374 | 0.004696081 | 0.011557633 |

| *AL096855.2* | 0.483800314 | 0.218345238 | -1.147800639 | 0.004717985 | 0.011601091 |
| --- | --- | --- | --- | --- | --- |
| *Z93943.1* | 0.114972642 | 0.05462619 | -1.073625882 | 0.004761724 | 0.011684974 |
| *RNU6-853P* | 1.465443082 | 0.58717619 | -1.31947156 | 0.004817472 | 0.011800887 |
| *HNRNPA1P14* | 0.570421698 | 0.221713095 | -1.363334877 | 0.004829517 | 0.011825681 |
| *AC084757.3* | 0.161896855 | 0.363119048 | 1.165367646 | 0.004834786 | 0.011832691 |
| *CACNA1G* | 0.347965094 | 0.102470238 | -1.763737643 | 0.004864877 | 0.011901599 |
| *STMN2* | 5.781927358 | 0.823004762 | -2.8125778 | 0.004902453 | 0.011987566 |
| *AP003108.4* | 0.209492453 | 0.087691667 | -1.256386615 | 0.004930666 | 0.012045773 |
| *AP001885.1* | 0.214256918 | 0.058795238 | -1.86557057 | 0.004976256 | 0.012140267 |
| *MPPED1* | 0.187509119 | 0.014057143 | -3.737585464 | 0.005005255 | 0.012197705 |
| *LINC02392* | 0.252663836 | 0.041194048 | -2.616711394 | 0.005010888 | 0.012207805 |
| *AC018616.1* | 0.496180503 | 0.234345238 | -1.082229568 | 0.005013969 | 0.012214101 |
| *AC092574.2* | 0.281287736 | 0.08887619 | -1.662177768 | 0.005025926 | 0.012235956 |
| *AL713922.2* | 0.552963836 | 0.155559524 | -1.829718409 | 0.00503635 | 0.012256411 |
| *SNORA20* | 1.114320755 | 0.37544881 | -1.569476447 | 0.00503682 | 0.012256411 |
| *HMGB3P4* | 0.448589308 | 0.216063095 | -1.053942562 | 0.0051376 | 0.012465878 |
| *SLC38A4* | 16.09763208 | 4.845115476 | -1.732245531 | 0.005143702 | 0.012476549 |
| *KCNJ13* | 0.25217044 | 0.112419048 | -1.165512673 | 0.00516733 | 0.012523188 |
| *AC024451.3* | 0.193635849 | 0.092433333 | -1.066860957 | 0.005180004 | 0.012551432 |
| *COL9A3* | 5.853083019 | 2.605410714 | -1.167685926 | 0.005202899 | 0.012594497 |
| *LRRC4C* | 0.419462893 | 0.157464286 | -1.413518541 | 0.005236073 | 0.012663279 |
| *ADAMTS7P3* | 0.598215723 | 0.286769048 | -1.060776515 | 0.005271068 | 0.012729894 |
| *CLVS2* | 0.183558176 | 0.028711905 | -2.676516429 | 0.005274535 | 0.012736564 |
| *DNALI1* | 5.520731447 | 2.525483333 | -1.128299903 | 0.005366564 | 0.012921998 |
| *AC011287.1* | 0.242261321 | 0.091294048 | -1.407971379 | 0.005430869 | 0.013039811 |
| *AC007204.1* | 0.260157547 | 0.100432143 | -1.373164489 | 0.005435648 | 0.013050011 |
| *KCNH4* | 1.372895597 | 0.602403571 | -1.188419691 | 0.005436837 | 0.013050355 |
| *SPOCK3* | 5.917770126 | 1.812804762 | -1.706830101 | 0.005465277 | 0.013112185 |
| *AC132825.1* | 0.317932704 | 0.02337381 | -3.765756636 | 0.005486152 | 0.013153288 |
| *NPTX2* | 6.137555346 | 2.840770238 | -1.111381978 | 0.00552589 | 0.013240816 |
| *AC083806.2* | 0.152148113 | 0.071572619 | -1.087996765 | 0.00558456 | 0.013363171 |
| *RGS9BP* | 0.940393082 | 0.442592857 | -1.087283757 | 0.005595548 | 0.013385557 |
| *AL031668.2* | 4.882677673 | 1.745958333 | -1.483653412 | 0.005598046 | 0.013388928 |
| *AL021068.1* | 2.869608805 | 0.895771429 | -1.679651521 | 0.005615617 | 0.013424426 |
| *AC127024.8* | 0.532019182 | 0.23632619 | -1.170698742 | 0.005693534 | 0.013581646 |
| *SGCZ* | 0.135541195 | 0.056129762 | -1.271893551 | 0.005700329 | 0.013593901 |
| *FGF9* | 0.585640252 | 0.10485 | -2.481687853 | 0.005819413 | 0.013843532 |
| *CR381670.1* | 0.132091509 | 0.054813095 | -1.268945227 | 0.005849287 | 0.013896576 |
| *NDST4* | 0.474206918 | 0.086058333 | -2.462129905 | 0.006029273 | 0.014267523 |
| *ZNF556* | 1.237369811 | 0.527692857 | -1.229506381 | 0.006047606 | 0.014302802 |
| *FNDC5* | 3.052341509 | 1.45562381 | -1.068278832 | 0.006117695 | 0.014446353 |
| *DAPK1-IT1* | 0.992548428 | 0.047760714 | -4.377241177 | 0.006127652 | 0.014464293 |
| *P2RX3* | 0.247025786 | 0.038782143 | -2.671197223 | 0.006159678 | 0.014527341 |
| *HMGB1P51* | 0.508002516 | 0.117258333 | -2.115145185 | 0.006218819 | 0.014638749 |
| *AF186192.1* | 0.270370755 | 0.125710714 | -1.104831492 | 0.006234957 | 0.014675331 |
| *AC120349.3* | 0.229075786 | 0.076069048 | -1.590443523 | 0.006263707 | 0.014731722 |

| *HSPE1P11* | 0.987005975 | 0.338002381 | -1.546025409 | 0.00631813 | 0.014839853 |
| --- | --- | --- | --- | --- | --- |
| *NPSR1-AS1* | 0.195045912 | 0.036815476 | -2.405429493 | 0.006348005 | 0.014901483 |
| *RN7SL239P* | 0.468244025 | 0.233945238 | -1.001089722 | 0.006448825 | 0.015112188 |
| *AC032044.2* | 1.083573585 | 0.508282143 | -1.092095678 | 0.006503454 | 0.015218454 |
| *HSPE1P5* | 0.252804717 | 0.54244881 | 1.101463616 | 0.006504806 | 0.01522017 |
| *AP000350.6* | 0.516363208 | 0.242511905 | -1.090330638 | 0.006564235 | 0.015341134 |
| *CCDC188* | 1.603750943 | 0.708642857 | -1.17831949 | 0.006659101 | 0.015530957 |
| *LINC01394* | 0.622853459 | 0.301720238 | -1.045681306 | 0.006679349 | 0.015570797 |
| *AC010457.1* | 0.548915409 | 1.277629762 | 1.218814082 | 0.00669962 | 0.015610655 |
| *AL606469.1* | 0.13655566 | 0.277129762 | 1.021072538 | 0.006714753 | 0.015634065 |
| *SLCO4C1* | 2.716774214 | 1.134180952 | -1.260243838 | 0.006722329 | 0.01564898 |
| *MTRNR2L3* | 0.273873585 | 0.089577381 | -1.612303734 | 0.006743248 | 0.015691396 |
| *NYAP2* | 0.267041509 | 0.061217857 | -2.125039564 | 0.006748161 | 0.015698476 |
| *SPAG16-DT* | 0.254155031 | 0.114480952 | -1.150601213 | 0.006792134 | 0.015785838 |
| *TRIM54* | 0.966392138 | 0.457554762 | -1.078664297 | 0.006824393 | 0.015851825 |
| *AL034346.1* | 1.232702516 | 0.467922619 | -1.397482806 | 0.006843476 | 0.015887147 |
| *CA6* | 0.081710377 | 0.34967619 | 2.097428346 | 0.006863063 | 0.015923092 |
| *CLEC18C* | 0.273106289 | 0.027315476 | -3.321672059 | 0.006906647 | 0.016001908 |
| *PVALB* | 38.1559566 | 14.10933333 | -1.435258475 | 0.00692243 | 0.016035626 |
| *AC012254.4* | 0.581029874 | 0.289259524 | -1.006247883 | 0.00693549 | 0.016062854 |
| *AL158824.1* | 0.37380283 | 0.163025 | -1.197184273 | 0.00696598 | 0.016124358 |
| *AL139132.1* | 0.389724528 | 0.13184881 | -1.563570189 | 0.006982399 | 0.016159323 |
| *NKD1* | 3.661407233 | 1.141464286 | -1.681512522 | 0.007095883 | 0.016377279 |
| *PTCHD3P3* | 0.29086195 | 0.107241667 | -1.439469031 | 0.007115747 | 0.016413871 |
| *SNORD3B-2* | 0.417409119 | 0.189647619 | -1.138140861 | 0.007117627 | 0.016416667 |
| *LINC01622* | 0.542005346 | 0.17169881 | -1.658427045 | 0.007248872 | 0.016674032 |
| *CDH4* | 0.281041195 | 0.14050119 | -1.000199261 | 0.007264859 | 0.016706069 |
| *ZSCAN5C* | 0.198037736 | 0.070572619 | -1.488594904 | 0.007282613 | 0.016740684 |
| *RPL7AP2* | 0.341907547 | 0.170227381 | -1.006143157 | 0.007302766 | 0.016774467 |
| *AC009264.1* | 0.426210377 | 0.205784524 | -1.050431233 | 0.00731421 | 0.016796048 |
| *RFTN1P1* | 0.135416038 | 0.316669048 | 1.225577248 | 0.00734503 | 0.016854234 |
| *UCA1* | 52.50409403 | 24.20147143 | -1.117335157 | 0.007370034 | 0.016895969 |
| *AC134775.1* | 0.298161006 | 0.118308333 | -1.333539896 | 0.007421137 | 0.016992408 |
| *ADCY10* | 0.322352516 | 0.133471429 | -1.272108297 | 0.007510104 | 0.017164096 |
| *CCDC85A* | 0.206815409 | 0.103242857 | -1.002301706 | 0.007543654 | 0.017229628 |
| *ARL4AP4* | 0.530069811 | 0.208557143 | -1.345739654 | 0.007617274 | 0.01737004 |
| *AC008397.1* | 0.16322327 | 0.064409524 | -1.341500823 | 0.007622771 | 0.017379663 |
| *AP000424.2* | 1.381299686 | 0.385507143 | -1.841196863 | 0.007668084 | 0.017470019 |
| *RGS11* | 1.341873585 | 0.422754762 | -1.666355854 | 0.007677798 | 0.01748567 |
| *KCNT1* | 0.207881447 | 0.056472619 | -1.880137557 | 0.007689806 | 0.017503684 |
| *AC116562.3* | 0.340077987 | 0.122791667 | -1.469652972 | 0.007732781 | 0.017584837 |
| *RYKP1* | 0.202380818 | 0.089752381 | -1.173050436 | 0.00774487 | 0.017605817 |
| *SNORA12* | 6.152177044 | 0.75609881 | -3.024450333 | 0.00775809 | 0.017632609 |
| *LINC00871* | 0.46921478 | 0.114064286 | -2.040401313 | 0.007833495 | 0.017771145 |
| *AC010343.3* | 0.44337956 | 0.923385714 | 1.058391151 | 0.008087649 | 0.018280272 |
| *LINC02886* | 0.381695283 | 0.151805952 | -1.330192997 | 0.008139677 | 0.01838604 |

| *MPRIP-AS1* | 0.252542138 | 0.10890119 | -1.213504406 | 0.008141177 | 0.01838774 |
| --- | --- | --- | --- | --- | --- |
| *AC023794.1* | 0.511813522 | 0.223009524 | -1.198512941 | 0.00814702 | 0.018399248 |
| *AC226118.1* | 0.157058805 | 0.065833333 | -1.254414673 | 0.008153825 | 0.018409545 |
| *AP001020.3* | 0.26980566 | 0.128007143 | -1.0756963 | 0.008259449 | 0.018612138 |
| *AC015722.2* | 0.150301258 | 0.73802619 | 2.295814931 | 0.008284983 | 0.018664549 |
| *AC022960.1* | 0.33428239 | 0.149965476 | -1.156436942 | 0.00828973 | 0.018668402 |
| *AC140479.5* | 0.397969811 | 0.165152381 | -1.268861228 | 0.008291836 | 0.018671435 |
| *ARG1* | 0.622010063 | 0.208891667 | -1.574182981 | 0.008300306 | 0.018688797 |
| *AC005921.3* | 0.357206289 | 0.073217857 | -2.286490026 | 0.008361938 | 0.018815508 |
| *TCAP* | 10.81818019 | 2.763779762 | -1.968743272 | 0.008458821 | 0.019007422 |
| *AC099795.1* | 0.331528931 | 0.132057143 | -1.327972435 | 0.008469768 | 0.019028316 |
| *TPTEP1* | 1.191775786 | 0.207871429 | -2.519349459 | 0.008536625 | 0.019147264 |
| *NWD2* | 0.24542327 | 0.068383333 | -1.843555395 | 0.008558677 | 0.019186227 |
| *AL596244.1* | 1.690783019 | 3.484892857 | 1.043422773 | 0.008685004 | 0.019425166 |
| *AC012076.1* | 0.337550314 | 0.122833333 | -1.458400444 | 0.008743256 | 0.019543001 |
| *RIIAD1* | 0.431078302 | 0.159780952 | -1.431854513 | 0.008759239 | 0.01957339 |
| *RABGAP1L-AS1* | 0.247950314 | 0.06999881 | -1.824648764 | 0.00876499 | 0.019582683 |
| *CALB2* | 7.966982075 | 19.61829881 | 1.30009471 | 0.008847002 | 0.019744612 |
| *BCAR4* | 1.661000943 | 0.549588095 | -1.595630234 | 0.009014208 | 0.020062935 |
| *C1orf194* | 0.294075472 | 0.110744048 | -1.408957299 | 0.009101184 | 0.020232715 |
| *RPL26P27* | 0.465696226 | 0.168366667 | -1.467782651 | 0.009271178 | 0.02055674 |
| *CPB2* | 0.127796541 | 0.053016667 | -1.269330916 | 0.009301477 | 0.020620203 |
| *GLYCTK-AS1* | 0.39742044 | 0.179219048 | -1.148942099 | 0.009390382 | 0.020783572 |
| *CHN2* | 0.122197484 | 0.055803571 | -1.130785222 | 0.009461066 | 0.020917427 |
| *FAM78B* | 5.494027987 | 1.197621429 | -2.197692319 | 0.009527101 | 0.021040748 |
| *AC010969.1* | 0.39038522 | 0.133733333 | -1.545539329 | 0.009555043 | 0.021090277 |
| *LINC01297* | 0.467849371 | 0.163420238 | -1.517457455 | 0.009628726 | 0.021225139 |
| *AL138847.2* | 0.124110692 | 0.026029762 | -2.253393385 | 0.009675348 | 0.021310721 |
| *LEMD1* | 2.93875 | 6.490636905 | 1.143157418 | 0.00969473 | 0.021345763 |
| *DUX4L34* | 0.16141761 | 0.061261905 | -1.397735851 | 0.009709892 | 0.021371495 |
| *CTNND2* | 1.025734906 | 0.476772619 | -1.105284635 | 0.009724786 | 0.021396616 |
| *LINC00112* | 0.123710377 | 0.050908333 | -1.280992785 | 0.009785272 | 0.021506612 |
| *GSG1* | 0.211110377 | 0.103592857 | -1.027072967 | 0.009808756 | 0.021550523 |
| *AC069236.1* | 0.157195912 | 0.056713095 | -1.470809898 | 0.009843567 | 0.021609631 |
| *AC005828.1* | 0.61115566 | 0.127445238 | -2.26166241 | 0.009850362 | 0.021620687 |
| *UGT2B4* | 0.207417296 | 0.083807143 | -1.307391084 | 0.010022607 | 0.021940009 |
| *AC007878.1* | 1.014087421 | 0.376694048 | -1.428716886 | 0.01003438 | 0.021957534 |
| *AC027329.1* | 0.071896855 | 0.272683333 | 1.923225948 | 0.010049257 | 0.021984649 |
| *SLURP1* | 18.10112296 | 3.872115476 | -2.224885319 | 0.010111203 | 0.022102474 |
| *AC118754.1* | 0.746109748 | 0.298017857 | -1.323989079 | 0.010181445 | 0.022236254 |
| *CTNNA3* | 0.279122642 | 0.114319048 | -1.287833353 | 0.010214636 | 0.022294885 |
| *KISS1R* | 1.397402201 | 0.430359524 | -1.699133017 | 0.010277268 | 0.022409712 |
| *AL391807.1* | 0.239862893 | 0.052458333 | -2.192966112 | 0.010355463 | 0.022560215 |
| *AC018462.1* | 0.214384906 | 0.102015476 | -1.071415301 | 0.010411457 | 0.022658116 |
| *MIR3609* | 16.84650283 | 0.703633333 | -4.581481493 | 0.010495848 | 0.022815528 |
| *PKNOX2* | 1.344280189 | 0.506109524 | -1.409312343 | 0.010552846 | 0.022919172 |

| *SLCO4A1-AS1* | 0.343394969 | 0.858572619 | 1.322071263 | 0.010803815 | 0.023393998 |
| --- | --- | --- | --- | --- | --- |
| *ONECUT2* | 2.338027673 | 0.50757619 | -2.203595704 | 0.010811678 | 0.023404611 |
| *AC027644.2* | 1.205759748 | 0.506432143 | -1.251501595 | 0.010812523 | 0.023404611 |
| *P2RX2* | 0.697258176 | 0.295669048 | -1.237709725 | 0.010861379 | 0.02348556 |
| *AC068189.2* | 0.199044654 | 0.085903571 | -1.212302107 | 0.010961711 | 0.023673368 |
| *FRMD6-AS2* | 0.226272013 | 0.099190476 | -1.189784638 | 0.011178702 | 0.02406802 |
| *LINC01362* | 0.157048428 | 0.041544048 | -1.91849581 | 0.011203678 | 0.024113351 |
| *AL136131.2* | 1.040909434 | 0.513085714 | -1.020572787 | 0.011212771 | 0.02413081 |
| *CPN2* | 0.080102516 | 0.234841667 | 1.551768942 | 0.011270233 | 0.024231148 |
| *PIEZO2* | 1.308799686 | 0.551310714 | -1.247306762 | 0.011364062 | 0.024400925 |
| *AC013470.2* | 0.331738994 | 0.158769048 | -1.063118918 | 0.011434483 | 0.024524259 |
| *AC104819.1* | 0.245259119 | 0.092630952 | -1.40474053 | 0.011618081 | 0.024868154 |
| *AC018797.3* | 1.041304088 | 0.390220238 | -1.416030927 | 0.011663161 | 0.024940769 |
| *SNORA22* | 2.221010377 | 0.849596429 | -1.386366527 | 0.011675937 | 0.024961578 |
| *LIN28A* | 1.096431761 | 0.01337619 | -6.357004919 | 0.011687585 | 0.024982136 |
| *AC097376.2* | 0.212588994 | 0.094225 | -1.173885112 | 0.01169971 | 0.025001535 |
| *IGFL1* | 183.3384025 | 77.52599881 | -1.241756895 | 0.011701879 | 0.025003999 |
| *CXCR1* | 0.703504403 | 1.725965476 | 1.29477225 | 0.011731585 | 0.025056587 |
| *PTX4* | 0.139479874 | 0.058061905 | -1.264393162 | 0.011944434 | 0.025436091 |
| *HSPD1P6* | 3.38659717 | 0.483334524 | -2.808742438 | 0.011958875 | 0.025458378 |
| *AF127577.6* | 0.388771069 | 0.052495238 | -2.888662398 | 0.01199331 | 0.025512596 |
| *AL450992.2* | 0.170187107 | 0.058433333 | -1.54225825 | 0.012023389 | 0.025566595 |
| *ESPNP* | 0.136796226 | 0.063345238 | -1.110720358 | 0.012103313 | 0.025712819 |
| *AL136418.1* | 0.410534591 | 0.136283333 | -1.590894647 | 0.012103656 | 0.025712819 |
| *AL731563.1* | 0.447085849 | 0.186105952 | -1.264427684 | 0.012127472 | 0.025758964 |
| *CCDC178* | 0.207560063 | 0.071405952 | -1.539412631 | 0.012204177 | 0.025908462 |
| *RNU6-1016P* | 4.149342138 | 1.848355952 | -1.166640006 | 0.01224326 | 0.025977977 |
| *AMTN* | 3.523070126 | 17.94494881 | 2.348672711 | 0.012269104 | 0.026023834 |
| *LINC02255* | 0.509757233 | 0.209819048 | -1.280664687 | 0.01231886 | 0.026113029 |
| *CYCSP52* | 0.510071698 | 0.21235119 | -1.264247857 | 0.01246134 | 0.026381526 |
| *BRINP1* | 0.900503459 | 0.240030952 | -1.907511362 | 0.012467364 | 0.026387603 |
| *MIR4639* | 1.513148113 | 0.738519048 | -1.034846175 | 0.012485055 | 0.026417311 |
| *ZNF98* | 0.685621698 | 0.247672619 | -1.468978385 | 0.012488984 | 0.026417311 |
| *FGF17* | 0.29136761 | 0.120720238 | -1.271172952 | 0.012515207 | 0.02646595 |
| *AC139792.1* | 0.286430818 | 0.064257143 | -2.156257984 | 0.01256079 | 0.026544086 |
| *AC015923.1* | 0.544753774 | 0.081425 | -2.742060565 | 0.012564024 | 0.026548639 |
| *MS4A6E* | 0.117849371 | 0.408190476 | 1.792298463 | 0.012604297 | 0.026626875 |
| *AC009495.2* | 0.219040881 | 0.109188095 | -1.004384583 | 0.012627488 | 0.026662126 |
| *F2* | 0.740932075 | 0.294492857 | -1.331108648 | 0.012653127 | 0.026711675 |
| *AF279873.2* | 0.205941195 | 0.097141667 | -1.084070301 | 0.012725618 | 0.026832462 |
| *SH3GL2* | 5.699947484 | 2.57182619 | -1.148155482 | 0.012773338 | 0.026912318 |
| *AC006946.1* | 0.489669811 | 0.199236905 | -1.297324352 | 0.012841303 | 0.027032355 |
| *CYP1A2* | 27.35631352 | 1.798545238 | -3.926971476 | 0.01299235 | 0.027303586 |
| *XIAPP2* | 0.142450943 | 0.02402619 | -2.567785354 | 0.013060433 | 0.027420888 |
| *RPL18P10* | 0.54145283 | 0.173844048 | -1.63904199 | 0.013099843 | 0.027483722 |
| *RBFOX3* | 0.804588679 | 0.244259524 | -1.719836624 | 0.013170682 | 0.027595821 |

| *EMILIN3* | 0.843023899 | 0.27835119 | -1.598667278 | 0.013192026 | 0.02763348 |
| --- | --- | --- | --- | --- | --- |
| *ARPC3P4* | 0.944813522 | 0.367586905 | -1.361944241 | 0.01322195 | 0.027684371 |
| *ARHGDIG* | 0.538869182 | 0.148719048 | -1.857345646 | 0.013395041 | 0.028003879 |
| *AC018557.2* | 0.235685849 | 0.106111905 | -1.151278617 | 0.013436294 | 0.028075805 |
| *ZBTB20-AS4* | 0.212207547 | 0.093947619 | -1.175547462 | 0.01351691 | 0.028220221 |
| *ARGFXP2* | 0.301269811 | 0.129079762 | -1.222793293 | 0.013524029 | 0.028230348 |
| *EPHA8* | 0.199890252 | 0.037769048 | -2.403931805 | 0.013689421 | 0.028505428 |
| *AC024451.1* | 0.204954403 | 0.049402381 | -2.052650501 | 0.013711613 | 0.028546806 |
| *NGFR* | 6.922394025 | 14.82381667 | 1.098573976 | 0.013750054 | 0.028614725 |
| *LRRN4* | 0.463051887 | 1.283392857 | 1.470717092 | 0.013813421 | 0.028719864 |
| *TMEM88B* | 1.518592138 | 0.616364286 | -1.30087927 | 0.014017954 | 0.029068933 |
| *RNF14P3* | 0.230418553 | 0.106632143 | -1.111614503 | 0.014021832 | 0.029074525 |
| *MAGEA8-AS1* | 0.443089623 | 0.189579762 | -1.224793577 | 0.014135309 | 0.029277733 |
| *AC004584.3* | 0.484047799 | 0.108075 | -2.163116682 | 0.014188152 | 0.029359984 |
| *SNORA73B* | 94.05620409 | 10.73393333 | -3.131344276 | 0.014327478 | 0.029600974 |
| *SLC8A1-AS1* | 0.18950283 | 0.069147619 | -1.454467914 | 0.014355292 | 0.029655947 |
| *AC090114.3* | 0.50589717 | 0.24819881 | -1.027347973 | 0.01443897 | 0.029801415 |
| *AL365223.1* | 0.144458805 | 0.042404762 | -1.768359953 | 0.014530871 | 0.029960785 |
| *VCX* | 3.279565409 | 1.223011905 | -1.423066202 | 0.014606629 | 0.030094281 |
| *RNU6ATAC16P* | 0.848516038 | 0.330690476 | -1.359460431 | 0.014912316 | 0.030613816 |
| *LINC00923* | 0.182860377 | 0.074554762 | -1.294370095 | 0.014984403 | 0.030733584 |
| *LINC00161* | 0.23401195 | 0.108883333 | -1.103799063 | 0.01516843 | 0.031038554 |
| *BMP4* | 19.49848962 | 9.593941667 | -1.023166803 | 0.015278102 | 0.031211054 |
| *AC027544.1* | 0.247934591 | 0.122504762 | -1.017121737 | 0.015395361 | 0.031416654 |
| *CLEC18B* | 0.926404088 | 0.281602381 | -1.717982088 | 0.015704056 | 0.031951154 |
| *RPL36AP29* | 0.45083522 | 0.208044048 | -1.115711214 | 0.015707403 | 0.031952678 |
| *LINC02147* | 0.125238365 | 0.03947619 | -1.665621898 | 0.015717323 | 0.03196757 |
| *AC138473.1* | 0.184792138 | 0.091429762 | -1.015167613 | 0.015874418 | 0.03223377 |
| *TSPEAR* | 0.64141195 | 0.174646429 | -1.876814091 | 0.015881731 | 0.03224589 |
| *ASB4* | 0.143434906 | 0.033672619 | -2.090748311 | 0.01601325 | 0.032480815 |
| *HEPACAM2* | 4.454258805 | 1.57992381 | -1.495330398 | 0.016186669 | 0.032769199 |
| *ACP3* | 9.008469811 | 2.866971429 | -1.651754542 | 0.016190063 | 0.032769199 |
| *CRABP1* | 2.209279874 | 0.935132143 | -1.240334042 | 0.01621653 | 0.032814656 |
| *VIP* | 1.170272327 | 0.542090476 | -1.110238724 | 0.016227887 | 0.032834937 |
| *AC008277.1* | 0.155397484 | 0.034647619 | -2.165135028 | 0.016266683 | 0.032899901 |
| *LDHAL6A* | 0.766410377 | 0.322096429 | -1.250624431 | 0.016322718 | 0.032991528 |
| *RPS3AP27* | 0.264462893 | 0.047972619 | -2.462782201 | 0.01646345 | 0.033226824 |
| *RNF113B* | 0.328326415 | 0.064941667 | -2.337914509 | 0.016564178 | 0.033394491 |
| *AC009754.1* | 0.875433962 | 0.40622619 | -1.107715097 | 0.016589264 | 0.033439439 |
| *GABRR3* | 0.181771698 | 0.059709524 | -1.606094621 | 0.016590645 | 0.033439439 |
| *A1CF* | 0.220951887 | 0.023410714 | -3.238491392 | 0.016694334 | 0.03361556 |
| *MUC5AC* | 0.805950314 | 0.378521429 | -1.090315926 | 0.016857144 | 0.033893443 |
| *TBC1D22A-AS1* | 0.201207547 | 0.066046429 | -1.607131965 | 0.016865604 | 0.033907681 |
| *PCDH10* | 1.055916667 | 0.319969048 | -1.722491724 | 0.01687295 | 0.033919678 |
| *RN7SL558P* | 0.664169811 | 0.282253571 | -1.234560313 | 0.016887737 | 0.033946629 |
| *CTSE* | 69.25124057 | 19.10067143 | -1.858216563 | 0.016952618 | 0.034040346 |

| *AP000302.1* | 0.152012893 | 0.074225 | -1.034216599 | 0.017018611 | 0.0341483 |
| --- | --- | --- | --- | --- | --- |
| *AL031768.2* | 0.208473899 | 0.1035 | -1.010236004 | 0.017176317 | 0.034408596 |
| *IGFN1* | 0.18475 | 0.788480952 | 2.093501539 | 0.017210691 | 0.034451578 |
| *AC103810.2* | 0.53548239 | 0.20580119 | -1.379587803 | 0.017211781 | 0.034451578 |
| *AL590550.1* | 1.528778302 | 3.065694048 | 1.003834518 | 0.017359756 | 0.034691301 |
| *ZBTB20* | 0.309509748 | 0.131257143 | -1.237588915 | 0.017901291 | 0.03560899 |
| *PMP2* | 0.308413522 | 0.09100119 | -1.760908696 | 0.017992903 | 0.035770548 |
| *KLHL14* | 0.433922642 | 0.136311905 | -1.670526301 | 0.018128906 | 0.036003094 |
| *AC078880.5* | 1.044540566 | 0.327765476 | -1.672132715 | 0.018157234 | 0.036038591 |
| *AC079915.1* | 0.166080503 | 0.038897619 | -2.094128965 | 0.018327258 | 0.036320756 |
| *AKAP14* | 0.090941195 | 0.185794048 | 1.030698416 | 0.018355265 | 0.036373329 |
| *PARM1-AS1* | 0.129485535 | 0.045857143 | -1.497572562 | 0.018359164 | 0.036378124 |
| *FRG1FP* | 5.426901887 | 1.624769048 | -1.739894165 | 0.018373676 | 0.036395151 |
| *MYLK4* | 4.325818239 | 2.125086905 | -1.025451207 | 0.018471147 | 0.036561754 |
| *SNX6P1* | 0.125877673 | 0.059804762 | -1.073690146 | 0.018607091 | 0.036789697 |
| *AC024337.1* | 0.469194025 | 0.106002381 | -2.146087972 | 0.018607263 | 0.036789697 |
| *AL021391.1* | 0.568901258 | 0.284158333 | -1.001483246 | 0.018644327 | 0.036825244 |
| *SEZ6* | 0.831623899 | 0.054455952 | -3.932769561 | 0.018644708 | 0.036825244 |
| *TRIML2* | 0.519281761 | 2.333820238 | 2.168103983 | 0.018677244 | 0.036874701 |
| *AC037487.1* | 0.251598742 | 0.113833333 | -1.144201632 | 0.018714166 | 0.036929809 |
| *OTOGL* | 0.21857044 | 0.09665 | -1.177256665 | 0.019054398 | 0.037482301 |
| *ZSCAN5B* | 0.343633019 | 0.105409524 | -1.704863447 | 0.019263725 | 0.037823093 |
| *IGHV3-79* | 0.572367296 | 0.284279762 | -1.009629844 | 0.019312235 | 0.037909259 |
| *LINC00605* | 0.870739623 | 0.351495238 | -1.30873623 | 0.019395604 | 0.038045581 |
| *TPH1* | 0.37467327 | 0.183728571 | -1.028057062 | 0.019409876 | 0.038061971 |
| *AL353586.1* | 0.227422642 | 0.088930952 | -1.354618351 | 0.019493393 | 0.038188665 |
| *LINC02723* | 0.076958491 | 0.224772619 | 1.546313895 | 0.019572993 | 0.038314086 |
| *KCNA5* | 0.502566981 | 0.250670238 | -1.003525172 | 0.019813591 | 0.038717066 |
| *Z99127.2* | 0.317357547 | 0.158225 | -1.004131584 | 0.019842134 | 0.038760719 |
| *AL450344.3* | 0.609262579 | 0.295689286 | -1.042982163 | 0.0198693 | 0.038810704 |
| *SNORA19* | 0.795188365 | 0.305972619 | -1.377894093 | 0.0199574 | 0.038945672 |
| *PURG* | 0.213362264 | 0.07104881 | -1.586422658 | 0.019962493 | 0.03894943 |
| *AC012085.2* | 0.610657233 | 0.233520238 | -1.386815223 | 0.020109173 | 0.039204519 |
| *BX571818.1* | 10.39947201 | 2.622220238 | -1.987649517 | 0.020170056 | 0.039307635 |
| *FRG1GP* | 3.905837736 | 1.915625 | -1.027816846 | 0.020219013 | 0.039381897 |
| *RERGL* | 0.951968868 | 0.437254762 | -1.122440298 | 0.020296302 | 0.039506708 |
| *LHX9* | 0.134488994 | 0.337461905 | 1.327236539 | 0.020400697 | 0.039684785 |
| *AC022395.1* | 0.14494434 | 0.039357143 | -1.880801598 | 0.020586738 | 0.039989751 |
| *AC103739.1* | 0.768355346 | 0.359830952 | -1.094454386 | 0.020748651 | 0.040256573 |
| *KRT16P6* | 18.42598239 | 40.97899524 | 1.153143072 | 0.02078095 | 0.0403097 |
| *GDF5* | 0.238898428 | 0.816232143 | 1.772582166 | 0.02079205 | 0.040320469 |
| *LRRC36* | 0.285395597 | 0.127471429 | -1.162789162 | 0.020815833 | 0.040355084 |
| *SNORD53B* | 1.359185849 | 0.659394048 | -1.043529968 | 0.020913724 | 0.040519311 |
| *TMEM179* | 0.599025157 | 0.221769048 | -1.433558571 | 0.02102104 | 0.040704783 |
| *AC079466.1* | 5.688387421 | 0.126402381 | -5.491924183 | 0.021052294 | 0.040758885 |
| *AC010680.5* | 0.232453774 | 0.110465476 | -1.073348292 | 0.021146588 | 0.040918951 |

| *SNORA80E* | 1.06292673 | 0.490735714 | -1.115023976 | 0.021373968 | 0.041284172 |
| --- | --- | --- | --- | --- | --- |
| *HTR3C* | 0.401038679 | 0.104497619 | -1.940271317 | 0.021473357 | 0.041440344 |
| *LHX8* | 0.182633962 | 0.059686905 | -1.613468725 | 0.021861673 | 0.042067257 |
| *AL138963.1* | 0.76694434 | 0.347933333 | -1.140310978 | 0.02201622 | 0.042309852 |
| *PHBP12* | 0.341217925 | 0.152204762 | -1.164679939 | 0.022340422 | 0.042831333 |
| *CLUL1* | 0.289188994 | 0.131407143 | -1.137968948 | 0.022371123 | 0.042873482 |
| *BUD31P2* | 0.335954403 | 0.163167857 | -1.041908551 | 0.022527518 | 0.043146312 |
| *LINC01732* | 0.137266352 | 0.277942857 | 1.017810283 | 0.022542507 | 0.043154608 |
| *AC084262.1* | 0.271101258 | 0.127905952 | -1.083748402 | 0.02255164 | 0.04316226 |
| *LRRC37A7P* | 0.446517296 | 0.175595238 | -1.346462337 | 0.022589301 | 0.043224249 |
| *MYRFL* | 0.794469811 | 0.345960714 | -1.199384179 | 0.022815562 | 0.043589367 |
| *AC008985.1* | 0.135913836 | 0.046192857 | -1.556950646 | 0.022875532 | 0.043690366 |
| *AC002558.3* | 0.203796855 | 0.075908333 | -1.42480161 | 0.02299267 | 0.043859592 |
| *AP000487.2* | 0.279398428 | 0.134642857 | -1.053186206 | 0.023101535 | 0.04404676 |
| *SNORA74A* | 7.023494969 | 0.517138095 | -3.763567619 | 0.023580478 | 0.044769049 |
| *TMEFF2* | 0.35021478 | 0.078791667 | -2.152125013 | 0.023590536 | 0.044784688 |
| *IMPA1P1* | 0.180208176 | 0.073854762 | -1.286901619 | 0.023667173 | 0.044916309 |
| *XKR5* | 0.201916352 | 0.075821429 | -1.413080208 | 0.023689352 | 0.044951464 |
| *GSG1L* | 0.164853774 | 0.019316667 | -3.093268846 | 0.023944434 | 0.0453585 |
| *AL365356.1* | 0.18646478 | 0.042594048 | -2.130179417 | 0.023947956 | 0.045361677 |
| *LINC01422* | 0.138221384 | 0.067259524 | -1.039170356 | 0.024079609 | 0.045568938 |
| *PROX1* | 1.790200943 | 0.796982143 | -1.167502229 | 0.024120327 | 0.045614577 |
| *TUBA3E* | 2.345248428 | 0.402539286 | -2.542539258 | 0.024328956 | 0.045966531 |
| *LINC01694* | 0.667296226 | 0.281883333 | -1.243229166 | 0.024551213 | 0.046354408 |
| *AGXT* | 0.406440252 | 0.085502381 | -2.249006786 | 0.024612851 | 0.04645652 |
| *AC012653.2* | 1.78960283 | 0.437210714 | -2.033238782 | 0.024855572 | 0.046857117 |
| *SNORA53* | 13.74987358 | 1.200571429 | -3.51762521 | 0.024904422 | 0.046931219 |
| *THSD7A* | 1.765039623 | 0.810166667 | -1.123409937 | 0.024960814 | 0.047030344 |
| *SHISA9* | 0.649093396 | 0.170014286 | -1.932770102 | 0.025010678 | 0.047117014 |
| *AP000350.2* | 0.330815094 | 0.161565476 | -1.033906111 | 0.025190437 | 0.047415706 |
| *NKX2-1* | 1.003954088 | 0.267764286 | -1.906657842 | 0.025307919 | 0.04759313 |
| *APOD* | 94.69805409 | 32.68142738 | -1.534863785 | 0.025861681 | 0.048508404 |
| *KCNH2* | 4.919472327 | 1.639004762 | -1.585683531 | 0.025967396 | 0.048684414 |
| *MTRNR2L10* | 0.35368805 | 0.169842857 | -1.058276931 | 0.026131404 | 0.048962041 |
| *GABRQ* | 3.856919497 | 1.092907143 | -1.819278204 | 0.026225787 | 0.049090265 |
| *LINC02065* | 0.165480189 | 0.39344881 | 1.249517435 | 0.026266393 | 0.04915879 |
| *RHCG* | 51.53558176 | 136.9859643 | 1.410387318 | 0.026286773 | 0.049178337 |
| *AC105916.1* | 0.132721384 | 0.061103571 | -1.119072221 | 0.026500737 | 0.049489922 |
| *BALC* | 5.759081761 | 1.956853571 | -1.557302999 | 0.026609629 | 0.049665167 |
| *IQCM* | 0.238643396 | 0.110878571 | -1.10587584 | 0.02665573 | 0.049731294 |
| *AC008750.2* | 0.282992453 | 0.125969048 | -1.167694291 | 0.026659242 | 0.049731294 |
| *STEAP3-AS1* | 0.409097799 | 0.193254762 | -1.081941811 | 0.026694995 | 0.049786661 |
| *EPO* | 0.612961635 | 0.256559524 | -1.256503198 | 0.026780133 | 0.049926515 |
