# Supplementary material for: Development and validation of a model based on immunogenic cell death related genes to predict the prognosis and immune response to bladder urothelial carcinoma
Source: Front Oncol. 2023 Nov 10;13:1291720. doi: 10.3389/fonc.2023.1291720 (PMC10676223; doi:10.3389/fonc.2023.1291720)
Supplement: Supplementary file 7 [file Table_7.docx]

**Supplementary Table 7 The list of 50-upregulated genes in ICD -high and -low subgroups**

| Gene | LowMean | HighMean | LogFC | Pvalue | Fdr |
| --- | --- | --- | --- | --- | --- |
| *IFNG* | 0.409871698 | 7.806232143 | 4.251382087 | 2.91E-45 | 7.15E-41 |
| *FASLG* | 0.605001572 | 5.423960714 | 3.164335932 | 1.41E-41 | 1.73E-37 |
| *NKG7* | 12.03408836 | 114.5427607 | 3.25068752 | 4.37E-41 | 3.58E-37 |
| *GZMH* | 3.733333333 | 44.2944381 | 3.568589324 | 7.09E-41 | 4.36E-37 |
| *GZMB* | 18.62394308 | 85.18884286 | 2.19350594 | 1.81E-40 | 8.89E-37 |
| *LAG3* | 3.83380566 | 34.76748929 | 3.180889775 | 2.92E-40 | 1.20E-36 |
| *TBX21* | 0.580447799 | 4.129179762 | 2.830616994 | 1.66E-39 | 5.85E-36 |
| *LINC02446* | 0.641336164 | 12.00728452 | 4.226685349 | 1.99E-39 | 6.11E-36 |
| *CCL4* | 6.270995283 | 43.39896786 | 2.790894392 | 2.46E-39 | 6.72E-36 |
| *PRF1* | 8.35062044 | 48.16152976 | 2.52792592 | 3.95E-39 | 9.71E-36 |
| *CD8A* | 4.589598113 | 38.89693095 | 3.083216593 | 6.24E-39 | 1.40E-35 |
| *PDCD1* | 1.951063836 | 12.15584762 | 2.639317608 | 2.81E-38 | 5.76E-35 |
| *GBP5* | 4.326373899 | 57.48752143 | 3.732018572 | 5.81E-38 | 1.10E-34 |
| *SLA2* | 1.515792453 | 9.112360714 | 2.587752629 | 6.84E-38 | 1.20E-34 |
| *CXCR6* | 2.264489937 | 11.4611881 | 2.339498572 | 1.13E-37 | 1.84E-34 |
| *GZMA* | 27.66733522 | 94.38215476 | 1.770330411 | 1.20E-37 | 1.84E-34 |
| *LINC01871* | 4.641851887 | 31.7507381 | 2.774017735 | 1.56E-37 | 2.26E-34 |
| *AC022126.1* | 0.179781132 | 1.626683333 | 3.177619905 | 9.31E-37 | 1.27E-33 |
| *CCR5* | 2.62373805 | 15.17773929 | 2.532261322 | 1.24E-36 | 1.61E-33 |
| *CD2* | 10.70428333 | 58.33114286 | 2.446078132 | 1.35E-36 | 1.66E-33 |
| *LINC02195* | 1.708227358 | 14.15116071 | 3.050348481 | 1.71E-36 | 2.00E-33 |
| *ZNF683* | 1.135642453 | 14.7093131 | 3.695149285 | 2.70E-36 | 3.02E-33 |
| *TRGC2* | 1.11122327 | 8.081508333 | 2.862475866 | 3.01E-36 | 3.22E-33 |
| *CXCR3* | 2.028167296 | 12.67797738 | 2.644076035 | 6.52E-36 | 6.68E-33 |
| *CXCL9* | 24.65012453 | 277.15505 | 3.491026455 | 7.54E-36 | 7.42E-33 |
| *TIGIT* | 1.869307547 | 8.05085119 | 2.106637375 | 1.58E-35 | 1.50E-32 |
| *CD7* | 6.584187736 | 34.19516905 | 2.376715144 | 2.48E-35 | 2.26E-32 |
| *CD3E* | 9.113149371 | 44.2194631 | 2.278659889 | 2.94E-35 | 2.59E-32 |
| *CALHM6* | 1.008275157 | 11.49933929 | 3.511589663 | 3.06E-35 | 2.60E-32 |
| *AC243829.4* | 0.099390566 | 0.606011905 | 2.608165309 | 3.19E-35 | 2.61E-32 |
| *IRF1* | 19.43979057 | 65.95345238 | 1.762435504 | 5.61E-35 | 4.45E-32 |
| *CCL5* | 95.53524969 | 359.5874583 | 1.912237655 | 5.83E-35 | 4.48E-32 |
| *FTH1P22* | 0.482734591 | 3.512269048 | 2.863101248 | 1.02E-34 | 7.61E-32 |
| *CXCL10* | 59.39033868 | 642.0553452 | 3.434397498 | 1.17E-34 | 8.44E-32 |
| *IL12RB1* | 1.954029874 | 10.09401429 | 2.368975604 | 1.58E-34 | 1.11E-31 |
| *GNLY* | 4.958233019 | 47.98977857 | 3.274829177 | 1.66E-34 | 1.13E-31 |
| *WARS1* | 63.68210189 | 438.0401667 | 2.782103306 | 1.94E-34 | 1.25E-31 |
| *TRAC* | 35.37382893 | 159.949956 | 2.176866302 | 1.94E-34 | 1.25E-31 |
| *CXCR2P1* | 0.609195597 | 8.156655952 | 3.74300038 | 2.41E-34 | 1.52E-31 |
| *SH2D1A* | 1.117277358 | 5.558272619 | 2.314649225 | 2.61E-34 | 1.61E-31 |
| *PSMB9* | 41.35654308 | 196.4541786 | 2.248005351 | 4.28E-34 | 2.48E-31 |
| *AL031846.1* | 0.380091509 | 2.540121429 | 2.740478762 | 4.29E-34 | 2.48E-31 |
| *CD3G* | 1.298792767 | 6.370629762 | 2.29426474 | 4.33E-34 | 2.48E-31 |
| *KIR2DL4* | 0.567654403 | 4.448365476 | 2.970190559 | 5.90E-34 | 3.30E-31 |
| *SIRPG* | 2.505215409 | 10.25994286 | 2.034016133 | 7.17E-34 | 3.92E-31 |
| *IL2RB* | 6.407859434 | 24.38835833 | 1.928278243 | 7.55E-34 | 3.95E-31 |
| *GBP1* | 38.15193553 | 210.5267798 | 2.464175606 | 7.55E-34 | 3.95E-31 |
| *CD3D* | 15.15251541 | 69.84288095 | 2.20455576 | 8.81E-34 | 4.51E-31 |
| *CASP5* | 0.331630503 | 2.455520238 | 2.888380097 | 1.12E-33 | 5.63E-31 |
| *TAF5LP1* | 1.134250943 | 5.9858 | 2.399804216 | 1.36E-33 | 6.69E-31 |
|  |  |  |  |  |  |
