# Supplementary material for: Development and validation of a model based on immunogenic cell death related genes to predict the prognosis and immune response to bladder urothelial carcinoma
Source: Front Oncol. 2023 Nov 10;13:1291720. doi: 10.3389/fonc.2023.1291720 (PMC10676223; doi:10.3389/fonc.2023.1291720)
Supplement: Supplementary file 8 [file Table_8.docx]

**Supplementary Table 8 The list of HLA genes**

| HLA genes |
| --- |
| *HLA-E*  *HLA-DPB2*  *HLA-C*  *HLA-J*  *HLA-DQB1*  *HLA-DQB2*  *HLA-DQA2*  *HLA-DQA1*  *HLA-A*  *HLA-DMA*  *HLA-DOB*  *HLA-DRB1*  *HLA-H*  *HLA-B*  *HLA-DRB5*  *HLA-DOA*  *HLA-DPB1*  *HLA-DRA*  *HLA-DRB6*  *HLA-L*  *HLA-F*  *HLA-G*  *HLA-DMB*  *HLA-DPA1* |
